# The impact of digital adherence technologies on treatment outcomes, adherence, and patient-reported outcomes in tuberculosis: a systematic review and meta-analysis

**DOI:** 10.1186/s12879-025-11503-3

**Published:** 2025-10-14

**Authors:** Mona S. Mohamed, Miranda Zary, Cedric Kafie, Chimweta I. Chilala, Shruti Bahukudumbi, Nicola Foster, Genevieve Gore, Katherine Fielding, Ramnath Subbaraman, Kevin Schwartzman

**Affiliations:** 1 McGill International Tuberculosis Centre, Research Institute of the McGill University Health Centre, Montréal, Canada; 2 TB Centre, London School of Hygiene and Tropical Medicine, London, UK; 3 Department of Public Health and Community Medicine, Tufts University School of Medicine, Boston, USA; 4 Schulich Library of Physical Sciences, Life Sciences, and Engineering, McGill University, Montréal, Canada; 5 Division of Geographic Medicine and Infectious Diseases, Tufts Medical Center, Boston, USA; 6 Centre for Outcomes Research and Evaluation, Research Institute of the McGill University Health Centre, 5252 Boulevard de Maisonneuve Ouest, Room 3D.63, Montreal, QC H4A 3S5 Canada

**Keywords:** Digital technology, Video-observed treatment, SMS, Digital pillbox, 99DOTS, Smartphone- application, Tuberculosis, Adherence

## Abstract

**Background:**

Incomplete tuberculosis (TB) treatment adherence may lead to unsuccessful treatment and relapse. Digital adherence technologies (DATs) may allow more person-centric approaches for supporting treatment adherence. We conducted a systematic review (PROSPERO- CRD42022313166) to evaluate the impact of DATs on adherence, treatment outcomes and patient-reported outcomes in persons treated for TB.

**Methods:**

We searched MEDLINE, Embase, CENTRAL, CINAHL, Web of Science and preprints from Europe PMC, and clinicaltrials.gov for relevant literature from January 2000 to March 2024. We considered experimental or cohort studies reporting quantitative comparisons of adherence, treatment outcomes and patient-reported outcomes between a DAT and the standard of care in each setting. We excluded studies where the technology was used only to log visit attendance or for “routine telephone calls” to patients. Risk of bias was assessed using the Cochrane risk of bias assessment tool and the Newcastle- Ottawa Scale. Pre-specified subgroup analyses considered study design, specific DAT interventions as well as income levels in the countries where studies were conducted.

**Results:**

Seventy-six studies (total 86,586 participants) were included evaluating SMS-based interventions (k = 18 studies), feature phone-based interventions (k = 8), medication sleeves with phone calls (branded as “99DOTS,” k = 6), video-observed therapy (VOT; k = 18), smartphone apps (k = 7), digital pillboxes (k = 21), ingestible sensors (k = 1), and interventions combining two DATs (k = 2).

Overall, the use of DATs was associated with a modest increase in treatment success in TB disease in both RCTs (OR = 1.14 [0.99, 1.30]; I^2^ = 57%, k = 34, very low certainty evidence) and observational studies (OR = 1.11 [0.94, 1.30]; I^2^ = 74%, k = 22, very low certainty evidence). Additionally, DAT use was linked to a significant increase in reporting of adverse events in RCTs (OR = 1.57 [1.25, 1.97]; I^2^ = 12%, k = 6, moderate certainty) while observational studies showed a similar but non-significant finding (OR = 1.39 [0.93, 2.09]; I^2^ = 0%, k = 3, moderate certainty). VOT was associated with an increased likelihood of treatment completion in TB infection (OR 4.69 [2.08; 10.55]; I^2^ = 0%, k = 2, low certainty evidence). VOT also increased frequency of adverse event reporting, as demonstrated in RCTs (OR = 1.9 [1.27; 2.84]; I^2^ = 0%, k = 3, moderate certainty evidence) and a similar but non-significant effect in observational studies (OR = 1.48 [0.91; 2.42]; I^2^ = 0%, k = 2, low certainty evidence). Other interventions involving smartphone apps were associated with increased treatment success in TB disease, with a significant effect observed in RCTs (OR 2.17 [1.07; 4.4]; I^2^ = 20%, k = 3, low certainty evidence) and a non-significant effect in observational studies (OR 1.51 [0.53; 4.3]; I^2^ = 60%, k = 3, very low certainty evidence). In contrast, interventions with 99DOTS were not associated with improvements in short-term clinical outcomes. There was substantial methodological heterogeneity among studies reporting on adherence. Few studies assessed patient-reported outcomes, though satisfaction was generally higher with DATs.

**Conclusion:**

Some DATs, notably VOT and smartphone apps, have been successfully used to support TB treatment. Although in many cases DATs did not improve clinical outcomes, they may improve efficiency and adherence, and may be preferred to traditional directly observed therapy by persons with TB. However, evidence remains highly variable, and generalizability limited. Higher quality data are needed.

**Trial registration:**

PROSPERO- CRD42022313166

**Supplementary Information:**

The online version contains supplementary material available at 10.1186/s12879-025-11503-3.

## Introduction

Tuberculosis (TB) has once again become the leading infectious cause of death worldwide, with the waning of the COVID-19 pandemic. About one-quarter of the world's population is estimated to have been infected with *Mycobacterium tuberculosis* [1]. In 2023, an estimated 10.8 million people developed TB disease worldwide, of whom 1.25 million died [2].

Incomplete adherence to treatment for TB disease may lead to treatment failure, increased risk of death or relapse. To improve adherence, the World Health Organization in 1994 endorsed directly observed therapy (DOT) where health workers, community volunteers or family members observe and record patients taking each dose [3]. In recent years, patients and health-care providers have taken advantage of improved global access to information and communication technologies, and many digital technologies that support medication adherence have emerged [4].

Digital adherence technologies (DATs) can be defined as interventions that include a digital component with the intention to measure or promote treatment adherence and/or reduce missed visits and/or loss to follow up, thereby improving treatment outcomes [5]. They can be patient-facing only, provider-facing only, or patient and provider-facing. In 2017 the WHO Guidelines for the treatment of drug-susceptible tuberculosis and patient care focused on three DATs: short messaging service (SMS); electronic medication monitors–namely medication boxes and medication sleeves; and video-observed therapy (VOT) [4]. As the field is evolving rapidly, other DATs have been identified in recent systematic reviews. [5–7] While some involve interactive communication with persons with TB, others may be used only to monitor drug ingestion [8–10]. Further description of the digital technologies used to support treatment adherence in TB is provided in Table 1.

**Table 1:** Digital adherence technologies used in TB

Type of digital technology	Description of use in TB
Short messaging service (SMS)	SMS (texting) is a standard feature built into all cellphones globally. It is used to communicate with persons with TB, either through regular, automated reminders to take their medication, refill their medication and/or to attend their appointments or by providing health-related information (one-way SMS). They can also facilitate interactions about care (two-way SMS) [4]
Video-observed therapy (VOT)	VOT uses video communication to enable healthcare providers to remotely verify medication adherence of persons with TB through videos. Persons with TB either record and send videos of every dose ingested through internet-enabled smartphones or tablets with free or customized video communication software to a server (asynchronous) or take their medication under direct vision through videoconferencing software -live videos- at a prearranged time (synchronous). VOT can also allow interactions with healthcare providers for support and advice [4]
Digital pillbox	The digital pillbox is a medication storage container with an electronic device. It records medication intake each time the person with TB opens the pillbox. With more recent versions the pillbox can send real-time electronic signals to a server to inform the healthcare provider of container opening (real time data). The box is sometimes configured to emit audible and/or visual alarms for medication reminders, and for medication refill reminders [4]
Feature-phone based	A feature-phone based intervention consists of live or automated phone calls with or without SMS, or a health application system/platform compatible with feature- phones. It does not require an internet connection and can also utilize Interactive Voice Response System or unstructured supplementary service data (USSD) to send drug reminders, motivational or educational messages. It can also provide interaction with study team members and other persons with TB for support and advice [9, 11, 12]
Phone calls with medication sleeves (99DOTS)	This intervention involves medication blister packs fitted with custom envelopes displaying unique phone numbers revealed only when the pills are removed. Persons with TB are expected to call these toll-free numbers -after they take their medication- in the revealed sequence, creating a person-specific dosing history to verify adherence. This technology is branded as 99DOTS [4]
Smartphone applications	Smartphone application-based interventions involve mobile phone applications or instant messaging service applications with or without subscription to an official account. The app can be used to send drug reminders, motivational and/or educational messages. It can also provide interaction with study team members for support and advice. It can be used to confirm drug intake [13]
Ingestible sensor	This consists of an edible ingestion sensor attached to each pill, an external wearable patch, and a paired mobile device that can detect, digitally record and transmit medication ingestion [10]

DATs may facilitate more person-centric, community rather than facility-based, and less intrusive approaches for monitoring TB medication adherence than existing DOT models [5]. Depending on the setting and care model, some studies have suggested that DAT-based treatment may be more effective than standard care in achieving favorable treatment outcomes [14].

In HIV care, DATs such as the SMS-based system WelTel, have been shown to improve medication adherence and increase rates of viral suppression compared to standard care [15]. While several reviews have investigated the use of DATs in TB, findings regarding health outcomes are inconsistent and may vary by country income level and technology type [6, 7, 14, 16]. A systematic review limited to randomized trials of DATs noted substantial variation in their impact on medication adherence and clinical outcomes [6]. A separate meta-analysis suggested that mobile-phone messaging had a modest effect in improving TB treatment success [17].

We conducted an inclusive systematic review and meta-analysis to evaluate the impact of different types of DATs on adherence, treatment outcomes and patient-reported outcomes in persons treated for TB disease and TB infection.

## Methods

Our systematic review and meta-analysis were conducted and reported using the Preferred Reporting Items for Systematic Reviews and Meta-Analyses (PRISMA) guidelines [18]. (S1 checklist), and followed a protocol registered as PROSPERO-CRD42022313166.

### Search strategy and study selection

On March 4th, 2024 (updated from April 25th, 2023), we searched MEDLINE(Ovid), Embase (Ovid), CENTRAL (Wiley), CINAHL, Web of Science Core Collection, Europe PMC preprints (including MedRxiv) and clinicaltrials.gov (for unpublished clinical trials). The search was conducted by a health sciences librarian (GG) to ensure that the most suitable search terms were used. Key elements included terms that denoted tuberculosis (disease or infection); digital technologies and tools (including mobile phone, smartphone, video observation, medication monitors, text messaging, etc.) and health outcomes (microbiologic conversion, treatment success, microbiologic cure, treatment failure, death, loss to follow-up, relapse/recurrence, subsequent TB disease after treatment for latent TB infection, medication adherence, and patient-reported outcomes). More detailed information about the search strategy and results is provided in S1 Table.

We also hand-searched conference abstracts from the Union World Conference on Lung Health from 2004 to 2022 and cross-checked reference lists and citations of included studies as a supplementary approach to identify further data sources.

We followed the PICOS format (participants, interventions, comparator, outcomes, and study design) to formulate the clinical question used for including studies in the systematic review. 

#### Study design

We included randomized controlled trials (RCTs), quasi-experimental studies, and cohort studies.

#### Population

The population of interest was individuals diagnosed and treated for TB disease or infection, including persons at risk of unfavorable outcomes (e.g., those with drug-resistant disease, those living with HIV, children).

#### Interventions

We considered studies that addressed DATs intended to monitor and support TB treatment adherence, including medication dosing but also visit attendance and continued follow-up. This included reminding patients to take their medications, facilitating digital observation of pill-taking, compiling patient dosing histories and identifying adherence patterns, which facilitate patient-centric approaches for monitoring TB medication adherence. We included all such interventions whether they engaged persons treated for TB, providers, or both. We excluded studies where the technology was an electronic health record used only to log visit attendance or if the intervention consisted only of non-automated “routine telephone calls” to patients. A detailed definition for eligible DAT interventions was developed before study selection and is provided in S2 Table.

#### Comparator

To be eligible for inclusion, studies had to report a comparator arm involving standard care in the same settings. This included various forms of directly observed therapy (DOT), self- administered therapy (SAT) or a combination of the two. Approaches to DOT used as the standard of care varied substantially and could include facility-based DOT (where people with TB had to visit clinics or other health facilities for in-person observation of dosing), field-based DOT (where a healthcare provider or other treatment supporter visited people with TB at their home or another location for observation of dosing), or family DOT (where a family member or other local supporter observed dosing).

#### Outcomes

We considered short- and long-term clinical outcomes, medication adherence, and patient-reported outcomes. Short-term clinical outcomes were treated as binary (yes/no) and included: treatment success, treatment completion, cure, loss to follow up, treatment failure, death during treatment, adverse events, emergence of anti-tuberculous drug resistance, microbiological conversion of sputum smear, completion of intensive phase treatment and composite outcomes of favorable or unfavorable outcomes. Short-term clinical outcomes are defined in S3 Table.

Long-term clinical outcomes were also treated as binary, and included recurrence or relapse (TB disease), defined as a repeat diagnosis of TB disease within 2 years of successful treatment; post-treatment death, defined as death within 2 years of successful treatment for TB disease; and subsequent risk of TB disease, defined as development of TB disease within 5 years of treatment for TB infection.

Adherence was considered as a binary, ordinal, or continuous outcome depending on the measures used in specific studies: These included indirect measures of medication adherence such as pharmacy refill visits; or more direct measures of medication ingestion as indicated by persons with TB themselves, DAT and DOT records, or biologic testing (e.g. drug metabolites in urine). Patient-reported outcomes were based on suitably validated instruments, administered to persons using or not using DATs, at comparable time points during/after treatment. They included health-related quality of life (HRQoL), functional status, perceived stigma and satisfaction.

To be included, studies had to report quantitative comparisons of adherence, treatment outcomes or patient-reported outcomes between a DAT and standard care with at least 20 participants using the DAT. Clinical outcomes could include treatment outcomes, other health outcomes and/or patient reported outcomes. We included data from full-text publications, preprints, or abstracts without any language restriction. We excluded review articles and studies that reported purely qualitative information.

After deduplication of items initially retrieved from our search, all remaining items were imported into Rayyan software (Rayyan, Cambridge USA). Selection of studies involved two levels of screening conducted in parallel by two independent reviewers (among MM, MZ, CK, CC, SB, NF and KS). In the first-level screen, all titles and abstracts were screened: all studies addressing TB and DATs were included. A third reviewer (KS, KF, or RS) resolved conflicts. In the second-level screen, two independent reviewers (MM, MZ, CK, SB, NF and KS) screened the full text of studies retained from the title and abstract review. Articles were excluded if they did not meet the inclusion criteria. In the event of discordant judgment regarding suitability, the conflicts were resolved by discussion, or a third reviewer (KS) resolved them. Excluded full texts were stored and the reasons for their exclusion were documented.

### Data extraction

Data extraction was conducted independently by two reviewers (among MM, MZ, CK, CC) into a standardised template. Disagreements between reviewers were resolved by consensus and discussion with a third reviewer (KS) when needed. Data extracted included study characteristics (design, year of study, country, setting, sample size, inclusion and exclusion criteria, limitations, strengths and conclusion), population characteristics (age, sex, number of participants in each group, TB condition, drug resistance, HIV co-infection), DAT intervention and comparator (type, duration, frequency of the intervention, approach if suboptimal adherence was detected), and outcomes (number of events in each group, measures of effect (means ratios, risk ratios, or odds ratios with respective confidence intervals).

### Quality assessment

Each eligible study was evaluated by two reviewers (MM, MZ, CK, CC) independently to assess methodological quality. We judged randomized trials to be at low, high, or unclear risk of bias in seven categories using Cochrane’s Assessment of Risk of Bias (S4 Table).

Similarly, two reviewers (MM, MZ, CK, CC) independently evaluated each cohort study using the Newcastle Ottawa Scale (S5 Table). Disagreements were resolved through discussion between the two reviewers, and with a third reviewer (KS) when needed.

### Data synthesis

For each included study, data were summarized in tabular format to highlight study and participant characteristics, as well as key features of the DAT and comparator interventions reported. Results for short-term clinical outcomes were pooled and summarized quantitatively. When treatment success (which includes both successful completion and microbiologic cure) and treatment completion were reported in the same study, we focused on treatment success for purposes of meta-analysis. When only treatment completion was reported in a study, we used those data when pooling the results for treatment success.

Results were pooled using an inverse-variance weighted random effects model to estimate overall odds ratios and 95% confidence intervals. For short-term clinical outcomes, the odds ratios in individual studies were calculated from the number of events in the intervention and control groups. When the direct estimate required for the analysis – an odds ratio with its 95% confidence intervals- was provided by the authors, it was used when pooling the data. Risk ratios in individual studies were used when odds ratios were not reported. The reciprocal of the odds ratio for unfavorable outcomes was used to calculate the odds ratio for treatment success in studies that addressed unfavorable outcomes only and did not report treatment success. For cluster-randomized trials and stepped-wedge trials, we used the reported effect estimates from analyses that properly accounted for the cluster design. When an effect measure was not reported, available crude data were used to estimate odds ratios and 95% confidence intervals. A continuity correction of 0.5 was applied for measures with zero cells.

Between-study variance was estimated by the DerSimonian-Laird method [19]. The meta-analysis was conducted using R version 4.2.3, R Foundation for Statistical Computing, Vienna, Austria, using the *metafor* [20] and *meta* [21] packages. When substantial heterogeneity was present (I^2^ > 50%) we conducted subgroup analyses to explore potential sources.

Meta-analyses were conducted separately for RCTs and observational studies. Pre-specified subgroup analyses considered specific DAT interventions as well as income levels in the countries where studies were conducted. Country income level was classified as high, upper-middle, lower-middle or low according to the classification of the World Bank [22]. When the number of pooled studies exceeded 10, publication bias was assessed qualitatively by visual inspection of funnel plot symmetry and quantitatively using Egger’s linear regression [23]. A sensitivity analysis was completed with and without data extracted from conference abstracts to confirm the robustness of the results.

Treatment adherence was summarized in tabular form and described narratively. Only odds ratios for adherence results with sufficiently homogeneous assessment measures were presented in forest plots. When adherence was reported in binary terms, odds ratios or risk ratios provided by the authors were used, or the odds ratio was estimated from the numbers of events in the intervention and comparison groups. For continuous adherence measurements, the standardized mean difference was calculated and transformed to the log odds using the Hasselblad and Hedges method [24]. As reports were much sparser, results for long-term clinical outcomes, and patient reported outcomes were summarized in tabular form and/or described narratively following PRISMA guidelines for best practices.

### Grading of evidence

Finally, MM rated the quality of evidence for each outcome we meta-analyzed, based on the Grading of Recommendations, Assessment, Development and Evaluations (GRADE) approach which considers study limitations, inconsistency of results, indirectness of evidence, imprecision, and publication bias.

## Results

**Fig. 1:**
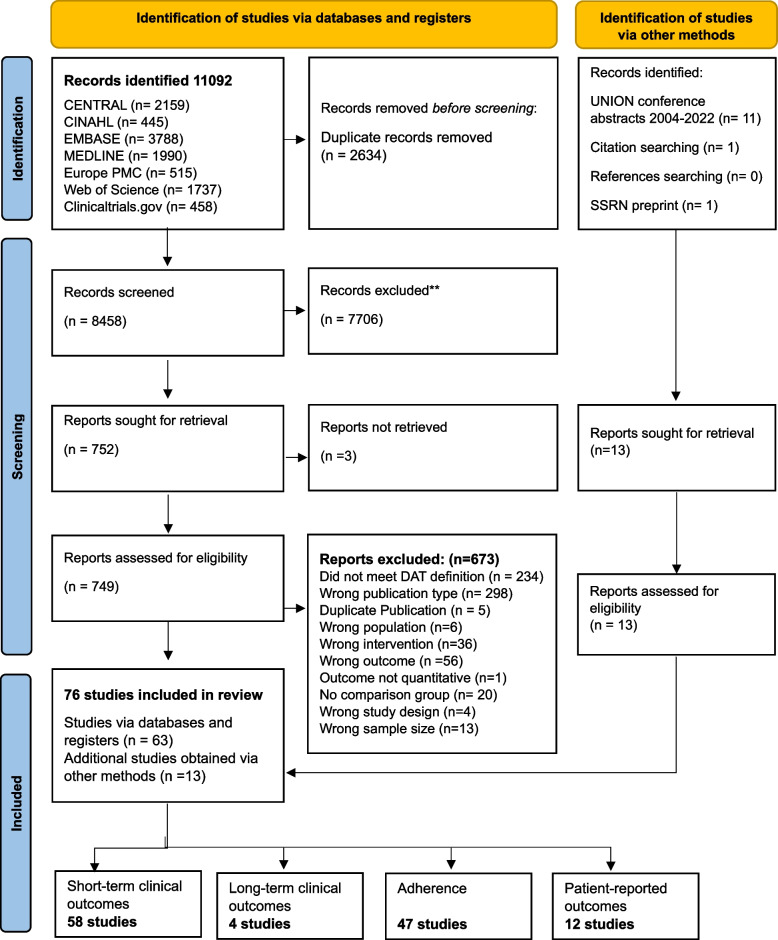
The PRISMA flow diagram explaining the flow of information through the different phases of the systematic review

Our search yielded 11,092 records as shown in Fig. 1. After removing 2,634 duplicate records, a further 7,706 items were excluded after title and abstract review, as they did not address TB and/or DATs; 749 full texts were therefore retained for further eligibility screening, of which 63 studies met inclusion criteria. Our search of the Union Conference abstracts identified 11 additional studies. One additional preprint was identified by a co-author [25] and one further study [26] was identified after reviewing citations of all included studies, for a total of 76 included studies. These 76 studies reported on 72 unique study cohorts. (Fig. 1).

### Characteristics of the included studies

Of the 76 included studies, 41 were randomized controlled trials (RCTs) (including one cross-over trial), six were quasi-experimental studies, and 29 were cohort studies. In the included studies, a total of 42,461 participants used DATs while 44,125 others received standard care. Reports addressed eight types of DAT intervention: SMS-based interventions (k = 18), feature phone-based interventions (k = 8), medication sleeves with phone calls (hereafter referred to by its brand name “99DOTS” (k = 6), video-observed therapy (VOT; synchronous and/or asynchronous) (k = 18), smartphone apps (k = 7), digital pillboxes (k = 21), ingestible sensors (k = 1) and interventions involving a combination of DATs (k = 2).

VOT, smartphone apps and ingestible sensors were exclusively studied in high income countries (HICs) and upper-middle income countries (UMICs), while the DATs reported in low-income countries (LICs) included SMS, feature phone-based interventions, digital pillboxes and 99DOTS.

Of the 76 included studies, nine used facility-based DOT as the comparator; four gave persons on treatment the choice between facility- or field-based DOT; 10 used field-based DOT provided by a health worker, of which three allowed observation at home; two used DOT provided by a family member or friend; and 14 used DOT without further details. Eleven used self-administered treatment (SAT), nine used a combination of DAT and SAT, and 16 did not clearly define the comparator. The standard of care differed according to the specific country setting in one multicenter cluster-randomized study [25]. In that study, some sites allowed self-administered treatment or observation of treatment administration by a suitable treatment partner, while other sites used home or clinic-based DOT (S6 Table).

**Fig. 2:**
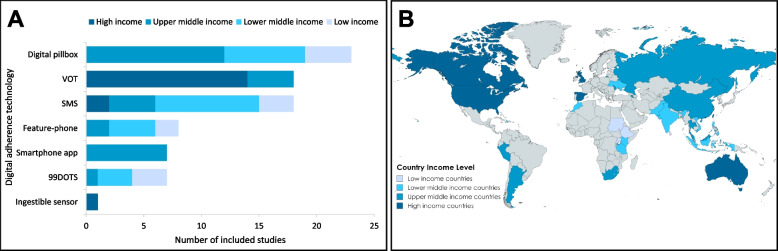
**A** Distribution of digital adherence technologies (DATs) by country income level in included studies. **B** Geographical distribution of included studies. VOT Video observed therapy, SMS Short service messaging. Four studies included several intervention arms with different DATs: Liu et al. 2015 included 3 different intervention arms (SMS, MERM and SMS + MERM), Wu et al. 2023 and Wang et al. 2024 included two intervention arms (MERM and smartphone app), Jerene et al., (MERM and 99DOTS) was conducted in lower and upper middle-income countries. Belknap et al. (SMS) was conducted in several upper-middle and high-income countries and is shown in the high-income category

Among participants in the DAT arms of the included studies, 99DOTS was provided to the largest number (41.9%). A summary of study and participant characteristics is provided in Tables 2 and 3, while Fig. 2 highlights DATs reported according to country income level. Detailed characteristics of each study, participants, interventions used, and outcomes reported are listed in (S6-S8 Tables).

**Table 2:** Summary of characteristics of included studies and participants

Subgroup	Number of studies	Number of DAT participants	Number of participants in SOC
All studies	76	42,461	44,125
Publication type			
Full-text paper	61	24,088	24,306
Preprint	3	14,497	13,501
Abstract	11	3307	5783
Research letter	1	569	535
Study design			
RCT	41	24,728	21,891
Quasi-experimental	6	1051	1496
Cohort study^a^	29	16,682	20,738
Type of DAT			
SMS	18^b^	4515	4788^b^
VOT	18	1741	2605
Digital pillbox	21^b,c,d^	13,470	22422^b,c,d^
Feature-phone	8	2036	2287
99DOTS	6^d^	17,792	21036^d^
Smartphone app	7^c^	1757	3998^c^
Ingestible sensor	1	41	20
SMS + digital pillbox	2^b^	1109	1125^b^
WHO region			
African Region (AFR)	23^d^	13,428	12,223
Eastern Mediterranean Region (EMR)	4	1464	1386
European Region (EUR)	6^d^	2077	2493
Region of the Americas (AMR)	16	1523	1909
South-East Asian Region (SEAR)	10	10,540	12,991
Western Pacific Region (WPR)	20^d^	13,429	13,123
Country income level			
Low	12	4192	3730
Lower-Middle	22^d^	23,244	25,277
Upper-Middle	25^d^	13,351	12,899
Upper-Middle and High	1	328	337
High	16	1346	1882
TB disease vs. infection			
TB disease	71	41,786	43,091
TB infection	4	628	987
Both	1	47	47
Drug resistance (TB disease)			
Drug-sensitive	46	35,721	36,542
Multi-drug resistant	2	151	211
Both drug-sensitive and drug-resistant	3	560	2215
Unknown	20	5354	4123

^a^One study was a comparative cross-sectional study with retrospective data, ^b^Liu et al. [27] included 3 DAT arms (SMS only, digital pillbox only and SMS and digital pillbox (combined) and one control arm, ^c^Wang et al. [28] and Wu et al. [29] included 2 DAT arms (digital pillbox and smartphone app) and one control arm, ^d^Jerene et al. [25] included two different interventions (digital pillbox or 99DOTS) in four countries (Philippines, Ukraine, South Africa and Tanzania)

**Table 3:** Characteristics of the included studies

DAT	Author	Year	Participants	Intervention	Time period	Setting	DAT (N)	SoC (N)	Comparator	Method	Outcomes assessed
**SMS- based intervention**	Ali et al. [30]	2019	PTB ^b^	One-way SMS	2017- 2018	Sudan	74	74	DOT- clinic	Prospective cohort	T
	Bediang et al. [31]	2018	SS + PTB^a,c^ (newly diagnosed)	One-way SMS	2013- 2014	Cameroon	137	142	SAT (a)	RCT	A,T,P
	Belknap et al. [32]	2017	LTBI	One-way SMS	2012- 2014	United States, Spain, Hong Kong, China	328	337	SAT (h)	RCT noninferiority trial	T
	Dewi et al. [33]	2019	Active TB (newly diagnosed)^b^	One-way SMS	2014	Indonesia	60	60	DOT- no details	Quasi-experimental	A,T
	Fang et al. [34]	2017	PTB^b,c^	One-way SMS	2014- 2015	China	160	190	DOT- no details	RCT	A,T
	Farooqi et al. [35]	2017	PTB^b^ (newly diagnosed)	One-way SMS + graphics-based messages	2014- 2015	Pakistan	74	74	not clear	RCT	T
	Gashu et al. [36]	2021	DS TB^a^(newly diagnosed)	One-way SMS + graphics-based messages	2019	Ethiopia	152	154	DOT- home (b)	RCT	A,T,P
	Hermans et al. [37]	2017	HIV + with active TB^b^	Two-way SMS	2010- 2011	Uganda	183	302	SAT (c)	Quasi-experimental	T
	Hirsch-Moverman et al. [38]	2017	HIV + with active TB	One-way SMS automated and coded	2013- 2015	Lesotho	183	166	SAT or DOT- family	Cluster RCT (The START study)	A
	Johnston et al. [39]	2018	LTBI	Two-way SMS	2012- 2015	Canada	170	188	SAT	RCT	T,P
	Kibu et al. [40]	2022	Active TB- no details	Two-way SMS and one-way SMS	Not specified	Cameroon	28	28	not clear	RCT (3 arms)	A
	Kumboyono [41]	2016	Active TB- no details	One-way SMS	Not specified	Indonesia	45	45	DOT- field	RCT post-test-only control-group design	A
	Liu et al. [27]	2015	Active TB ^b,c^	Two-way SMS	2011- 2012	China	1008	1104	Choice from SATor DOT- family or field (h)	Cluster RCT^	A,T
	Louwagie et al. [42]	2022	DS PTB^b^ literate, Tobacco smokers	One-way SMS	2018- 2019	South Africa	283	291	Not clear (d)	Multicenter RCT	A,T
	Mohammed et al. [43]	2016	Active TB^b^ (newly diagnosed)	Two-way SMS automated & coded	2011- 2014	Pakistan	1110	1097	DOT- no details	RCT	A,T
	Nguyen et al. [44]	2014	PTB (newly diagnosed)	One-way SMS	2012- 2013	Vietnam	136	270	Not clear	Retrospective cohort; HC	A
	Owiti et al. [45]	2012	Active TB- no details	One-way SMS	2011	Kenya	150	37	NR	Prospective cohort	A
	Peng et al. [46]	2014	Active TB- no details	One-way SMS	2011- 2012	China	234	229	DOT- no details	Cluster RCT	A,T
**Video-observed therapy**	Bachina et al. [47]	2022	DS PTB and EPTB	Asynchronous VOT	2019- 2021	United States	23	26	DOT-home or field	Prospective cohort	A
	Burzynski et al. [48]	2022	DSTB	Patient’s choice between synchronous or asynchronous VOT	2017- 2019	United States	113	103	DOT- clinic or field	Two-period crossover, noninferiority trial	A
	Chen et al. [49]	2020	LTBI	Synchronous VOT	2014- 2017	Taiwan	80	160	DOT- field	Retrospective cohort	A,T,P
	Chuck et al. [50]	2016	DS or DR, PTB or EPTB	Synchronous VOT pre-arranged schedule	2013- 2014	United States	61	329	DOT- variable €	Prospective cohort	T
	Doltu et al. [51]	2021	Active TB- no details	Asynchronous VOT	2016- 2017	Moldova	83	478	DOT- clinic	Retrospective cohort α	A,T
	Garfein et al. [52]	2018	DS PTB	Asynchronous VOT	2014- 2015	United States	272	159	DOT- field (f)	Prospective cohort; HC	A
	Guo et al. (a) [53]	2020	DS PTB	Asynchronous VOT	2017- 2018	China	235	158	DOT- clinic	Retrospective cohort; HC	A,T
	Guo et al. (b) [54]	2020	DS PTB (bacteriologically confirmed)	Synchronous VOT	2018	China	203	202	DOT- no details	RCT	T,P
	Lam et al. [55]	2018	LTBI	Synchronous VOT	2015	United States	50	302	DOT- clinic	Prospective cohort; HC	T
	Lippincott [56]	2022	PTB and EPTB	Asynchronous VOT (2 way)	2019- 2021	United States	30	22	DOT-home or field	Retrospective cohort	A
	Perry et al. [57]	2021	DS or DR, PTB or EPTB	Asynchronous VOT	2018- 2019	United States	94	69	DOT (g)	Prospective cohort	A,T
	Ravenscroft et al. [58]	2020	DS or DR, PTB or EPTB but not MDR-TB patients	Asynchronous VOT (2way)	2016- 2017	Moldova	85	93	DOT- clinic	RCT	A,T,P
	Salcedo et al. [59]	2021	DS PTB	Asynchronous videos on an AI platform that uses computer vision and machine learning accessed by a smartphone app	2015- 2017	United States	43	71	DOT- clinic or field	Prospective cohort historical control	A,T
	Salerno et al. [60]	2023	DS TB	Choice between Synchronous or asynchronous VOT	2017–2019	United States	113	103	DOT- clinic or field	Two-period crossover trial	T
	Siddiqui et al. [26]	2019	Active TB and LTBI	Asynchronous VOT	2014- 2015	United States	47	47	DOT-field (m)	Retrospective cohort	A
	Story et al. [61]	2019	DS, PTB or EPTB	Asynchronous VOT	2014- 2016	United Kingdom	112	114	DOT- field	Multicenter RCT superiority trial	A,T,P
	Wade et al. [62]	2012	Not specified	Synchronous VOT	2003- 2010	Australia	58	70	DOT- field	Retrospective cohort	A,T
	Yu et al. [63]	2013	MDR TB	Synchronous VOT	2007- 2011	Taiwan	39	99	DOT- field	Prospective cohort	T
**Digital pillbox**	Acosta et al. [64]	2021	DS PTB	Real-time monitoring	2018- 2020	Peru	53	53	DOT- clinic	RCT	A,T
	Broomhead and Mars [65]	2012	DS PTB	Real-time monitoring	data from a 2005 pilot	South Africa	24	96	DOT- no details	Retrospective cohort	T
	Charalambous et al. [66]	2022	DS TB	Real-time monitoring	Not specified	South Africa	1306	1278	Not clear (h)	Cluster RCT	A
	Guo et al. [67]	2023	PTB	Real-time monitoring	2016	China	93	74	DOT- no details	RCT	A
	Kurada et al. [68]	2019	DS PTB		2018	India	385	3019	DOT- no details SAT or field DOT SAT (p) SAT or field DOT	Prospective cohort	T
	Jerene et al. [25]	2024	DS PTB	Real-time monitoring	2021–2022	Philippines	2236	4188	Clinic or home DOT	Cluster RCT	T
			DS TB			South Africa	1281	1880			
			DS TB			Tanzania	1778	3656			
			DS TB			Ukraine	1526	1589			
	Liu et al. [27]	2015	Active TB^c^	Real-time monitoring	2011- 2012	China	997	1104	Choice from SATor DOT- family or field (h)	Cluster RCT^	A,T
	Liu et al. [69]	2023	DS PTB	No real-time monitoring (assumed)	2017- 2019	China	1298	1388	Choice from SATor DOT- family or field	Cluster RCT (superiority trial)	A,T
	Manyazewal et al. [70]	2022	DS PTB	No real-time monitoring	2020- 2021	Ethiopia	57	57	DOT- clinic (n)	Multicenter RCT	A,T
	Manyazewal et al. [71]	2023	DS PTB	No real-time monitoring	2020- 2021	Ethiopia	57			Multicenter RCT	T,P
	Manyazewal et al. (b) [72]	2022	DS PTB	No real-time monitoring	2020- 2021	Ethiopia	57			Multicenter RCT	P
	Moulding & Caymittes [73]	2002	DS PTB	Real-time monitoring	Not specified	Haiti	64	59	SAT	RCT	A,T
	Park et al. [74]	2019	DS PTB	Real-time monitoring	2014- 2015	Morocco	206	141	DOT & SAT (i)	Retrospective cohort	A,T
	Pima et al. [75]	2021	Active TB- no details	Real-time monitoring	Not specified	Tanzania	297	228	Not clear	RCT	A
	Ratchakit-Nedsuwan et al. [76]	2020	DS PTB or EPTB	Real-time monitoring	2014- 2015	Thailand	50	50	SAT or DOT- family	Mixed methods pilot RCT	A,T
	Saha et al. [77]	2022	DS TB	Real-time monitoring	2020- 2021	India	200	200	Not clear “SoC according NTEP guidelines”	Quasi-experimental	A,T,P
	Velen et al. [78]	2022	DS PTB	Real-time monitoring	2017- 2020	Vietnam	124	126	Not clear (h)	RCT	A,T
	Wang et al. [79]	2020	DS PTB	No real-time monitoring	2018	China	1047	763	SAT (j)	Prospective cohort	T
	Wang et al. [28]	2024	PTB	Real time-monitoring	2022	China	124	2136	SAT(o)	Prospective ohort	A
	Wei et al. [80]	2024	DS PTB	real time monitoring	2018- 2021	China	143	135	SAT (h)	Multicentre RCT	A,T
	Wu et al. [29]	2023	DS PTB	Real-time monitoring	2019	China	90	88	SAT or DOT- family	Prospective cohort	A,T
**Feature-phone based**	Bassett et al. [81]	2016	Active TB- no details	SMS and phone calls	2010- 2012	South Africa	293	230	Not clear	Multicenter RCT	T
	Das Gupta et al. [12]	2020	TB (newly diagnosed)	SMS, live calls and pre-recorded calls (group A) or SMS & phone calls (group B)	2015	India	111	111	DOT- clinic (k)	Quasi-experimental	T,P
	Hope et al. [82]	2022	Active TB- no details	interactive voice response software- The CallforLife tool	2020- 2021	Uganda	210	248	Not clear	Prospective cohort	T
	Hope et al. [83]	2022	Active TB- no details	interactive voice response software- The CallforLife tool	2020 −2021	Uganda	129	131	Not clear	RCT	T
	Khachadourian et al. [84]	2020	DS PTB	SMS and phone calls	2014	Armenia	227	209	DOT- clinic	Cluster RCT (noninferiority trial)	T,P
	Santra et al. [85]	2021	Active TB^c^	SMS and phone calls	2018- 2019	India	110	110	DOT- no details	Quasi-experimental	A
	Sodhi et al. [11]	2023	DS TB	Connect for Life; Interactive Voice Response System and SMS	2019–2020	India	713	276	Not clear	Quasi-experimental	T
	Yoeli et al. [9]	2019	DS TB	Keheala: Mobile phone platform and SMS	2016- 2017	Kenya	569	535	Not clear	RCT	T
**99DOTS**	Cattamanchi et al. [86]	2021	DS PTB	99DOTS	2018- 2019	Uganda	891	1022	DOT- field or family	Stepped-wedge cluster RCT	T
	Chen et al. [87]	2022	DS PTB and EPTB	99DOTS Real-time monitoring digital pillbox	Pre-intervention 2017 and post-intervention group 2018- 2019	India	8322	7722	DOT DOT- field SAT or field DOT	Retrospective cohort (pre- post study)	T
	Crowder et al. [88]	2024	DS PTB	99DOTS	2019- 2021	Uganda	2051	1475	SAT (p)	Retrospective cohort (pre- post study)	T
	Jerene et al. [25]	2024	DS PTB	99DOTS	2021–2022	Philippines	2368	4188	SAT or field DOT	Cluster RCT	T
			DS TB			South Africa	1213	1880			
			DS TB			Tanzania	1768	3656			
	Thekkur et al. [89]	2019	HIV + with DS TB	99DOTS	2016	India	870	961	DOT- field	Mixed-methods study with quantitative cohort study	T
	Wambi et al. [90]	2022	DS PTB	99DOTS	2020- 2021	Uganda	309	132	DOT	not specified- assumed retrospective cohort study	T
**Ingestible sensor**	Browne et al. [10]	2019	SS- TB	Ingestible sensors^d^ (WOT)	2013- 2016	United States	41	20	DOT- clinic or field	Stage 1: Prospective cohort Stage 2: RCT	A
**Smartphone apps**	Haslinda & Juni [91]	2019	DS PTB (newly diagnosed)	TB@Clicks educational module delivered through Whatsapp	2017- 2018	Malaysia	55	55	DOT- clinic	RCT	A,T
	Iribarren et al. [13]	2022	DS PTB	Mobile application	2019- 2020	Argentina	21	21	SAT (l)	RCT (mixed methods)	T
	Wang et al. [28]	2024	PTB	WeChat application and subscription to ‘‘E taking medication” account	2022	China	124	2136	SAT(o)	Prospective cohort	A
	Wu et al. [29]	2023	DS PTB	A reminder app	2019	China	82	88	SAT or DOT- family	Prospective cohort	A,T
	Zhadnova et al. [92]	2020	HIV + with PTB with a history of psychoactive substances abuse	A specially developed smartphone app	Not specified	Russian Federation	44	10	Those who refused to use the app	Prospective cohort	T
	Zhang [93]	2023	DS PTB	Mobile phone application, or WeChat (an instant messaging service)	e-PSS group 2021 TCIS group 2020	China	1145	1576	Not clear	comparative cross-sectional study using retrospective data	A,T
	Zhou et al. [94]	2018	MDR TB	Messaging smartphone app	2013- 2015	China	112	112	Not clear	RCT	A,T
**Interventions combining two DATs**	Liu et al. [27]	2015	Active TB^c^	Real-time monitoring digital pillbox and SMS	2011- 2012	China	997	1104	Choice from SATor DOT- family or field (h)	Cluster RCT^	A,T
	Musiimenta et al. [95]	2023	DS TB	Real-time monitoring digital pillbox and SMS	2019–2020	Uganda	45	21	SAT with pillbox	Mixed methods pilot RCT	A

DAT Digital adherence technology, Soc Standard of care, Outcomes: A = adherence, T = Treatment outcomes (short-term or long-term clinical outcomes), P = patient-reported outcomes

*PTB* pulmonary TB, *EPTB* extra-pulmonary TB, *DS* drug susceptible, *DR* drug resistant, *MDR* multidrug resistant, *LTBI* latent tuberculosis infection, *SAT* self-administered therapy, *DOT* directly observed treatment, *MR* medication reminder, *AR* appointment reminder, *MM* motivational message, *EM* educational message, *XR* examination reminder, *RR* refill reminder, *AF* adherence feedback, *CTS* communicate with treatment supporter, *CP* communicate with other patients, *WOT* wireless observed therapy

^a^participants enrolled own a mobile phone,

^b^participants enrolled have access to a mobile phone,

^c^patient or family member can read

^d^a sensor made of minerals that is swallowed with TB medication and subsequently records the ingestion on a cellphone

In South Africa and Tanzania the DAT intervention started 1 week after treatment initiation, while in Ukraine it started after 2 months of treatment initiation [25]

(a) Routine care was referred to as selective DOT. Selective DOT included free treatment, appointments for drug refill, 3 smear control tests at 2, 5, 6 months, education coupled with counselling throughout the process

(b) Routine care in continuation phase TB treatment means patients take their daily medication at home with the help of community health worker), family member, neighbour, workmate

(c) DOT was not practiced, although “community-based DOT with treatment buddies” was advocated

(d) The routine care includes health education, dietetic input, social support, point of care biochemical testing, and HIV testing with pretest and post-test HIV test counselling

(e) “Patients on in person DOT were defined as those who had at least one dose of medication observed at a health department or hospital clinic or in the community and underwent no VDOT observation”

(f) San Francisco also offered clinic-based DOT. Nonclinical personnel conducted most DOT visits; nurses also provided some DOT based on clinical needs and staffing considerations

(g) Patients with no VOT include some patients who received exclusively SAT

(h) All participants in the control group were provided with e-monitors with reminder feature turned off

(i) DOT for the initial one to two weeks and SAT for the remaining Period

(j) The village doctors visit patients every ten days during the first two months of treatment followed by once a month

(k) DOTS motivators also conducted follow‐up home visits after patients had failed to report to DOTS centers for 1 week

(l) Monthly visits but patient may return earlier if they experience any problems with treatment

(m) Assumed from the cost analysis

(n) Some take home doses were allowed

### Risk of bias in included studies

Performance bias was common in almost all RCTs, as it was not feasible to mask participants to the interventions used. We rated RCT studies that lacked sufficient information about participant blinding as of “some concern”. A high risk of detection bias was also present in 30% of the studies, where outcome assessors were not blinded. Other biases in some studies included inclusion or exclusion of specific patient groups, or recruitment bias. Some studies restricted participation to individuals who had a low probability of being lost to follow-up and low risk of poor adherence to treatment [48, 64, 86]. All studies had either low or unclear risk of selection bias (S1 and S2 Figs). For the treatment success, LTFU, death and treatment failure outcomes, very few studies showed no detailed tracking of participant attrition; one study indicated that outcomes were unknown for 11% of participants. All RCTs that reported on adverse events reporting showed low or unclear selection bias, attrition bias or reporting bias.

More than 60% of the included observational studies had a high risk of bias related to participant representativeness. Many studies required participants to be literate, have access to a mobile phone, demonstrate digital literacy, and/or have a reliable internet connection to participate (S3 Fig, S9 and S10 Tables).

### Short-term clinical outcomes in TB disease

**Fig. 3:**
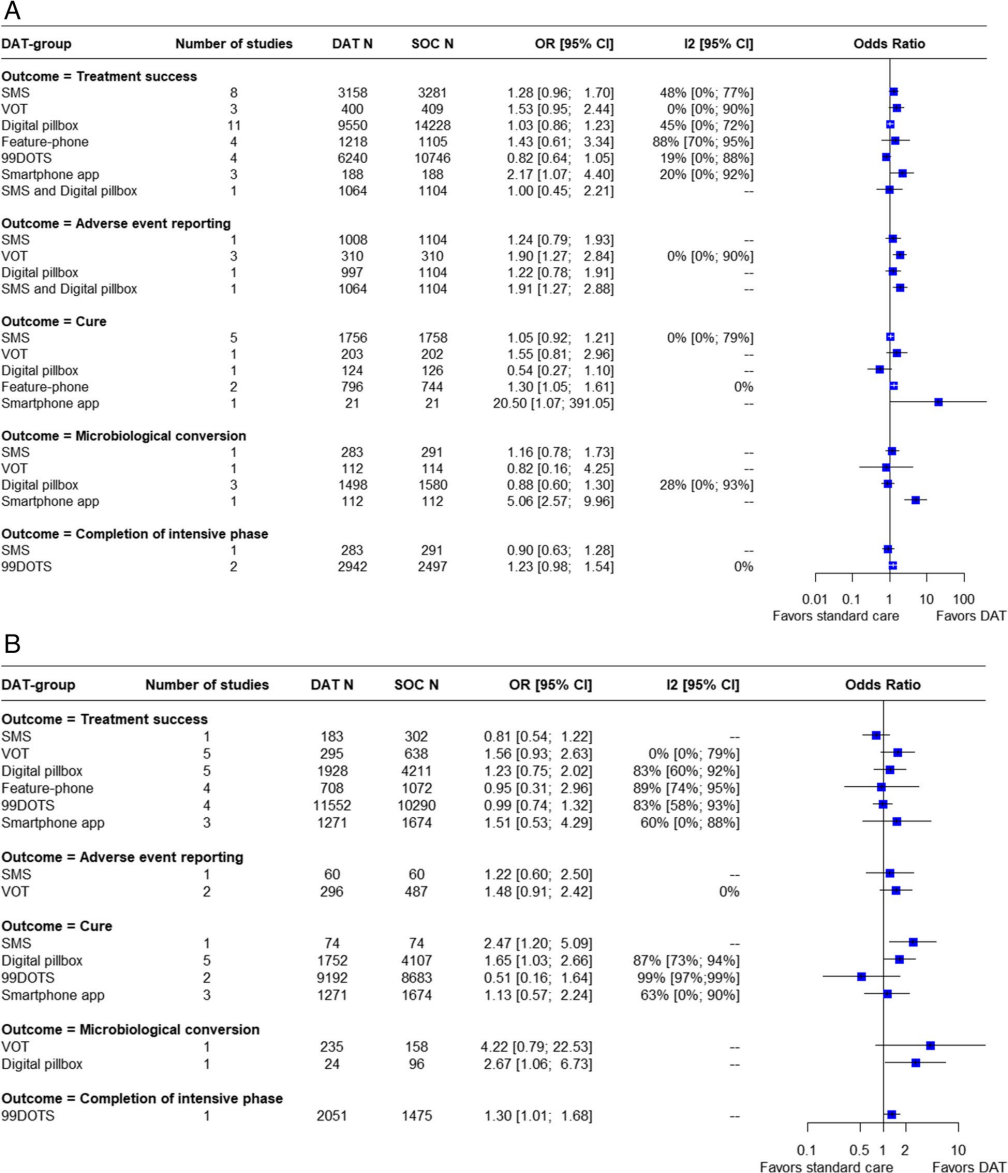
Pooled estimates for favorable short-term clinical outcomes in TB disease stratified by type of DAT a) in RCTs b) in observational studies

**Fig. 4:**
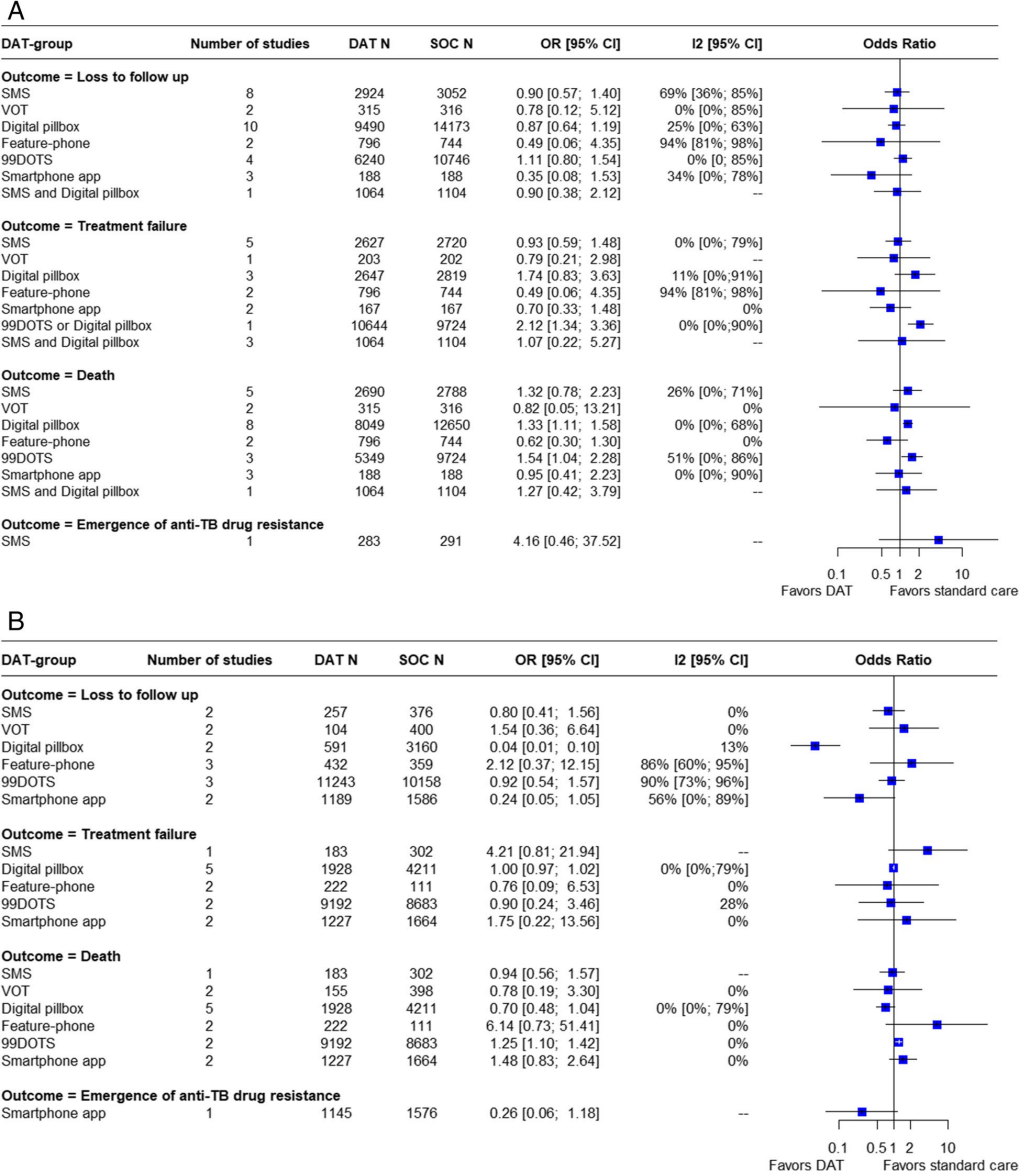
Pooled estimates for unfavorable short-term clinical outcomes in TB disease stratified by type of DAT a) in RCTs b) in observational studies

A summary of findings for the effect of digital technologies on short-term clinical outcomes in TB disease is provided in Figs. 3 and 4 and S11 Table.

#### Treatment success

Overall, when all studies were considered together, the use of digital technologies was associated with a small increase in treatment success, with substantial heterogeneity across studies (OR = 1.18 [1.06, 1.33]; I^2^ = 68%, k = 56, [very low certainty evidence]). When RCTs and cohort studies were considered separately, the use of DATs did not significantly increase treatment success in RCTs alone, nor in cohort studies alone, although point estimates of effect were similar to the combined results. (S4a Fig) In RCTs video-observed therapy (OR 1.53 [0.95; 2.44]; I^2^ = 0%, k = 3, [low certainty evidence]) and smartphone apps (OR 2.14 [1.07; 4.4]; I^2^ = 20%, k = 3, [low certainty evidence]) were each associated with an increased likelihood of treatment success for persons with TB disease. In a meta-regression analysis, we found that treatment success with SMS-based interventions and digital pillboxes did not vary significantly according to country income level or by study design (S1 and S2).

#### Loss to follow up

Similarly, DATs overall were associated with a significant decrease in the proportion of persons lost to follow up, again with substantial heterogeneity (OR = 0.77 [0.61, 0.98; I^2^ = 77%, k = 44, [low certainty evidence]). However, the use of DATs did not significantly decrease the number of persons lost to follow up in RCTs alone nor in cohort studies alone, again with similar point estimates (S5a Fig). Among the specific DATs, the use of digital pillboxes was associated with decreased likelihood of loss to follow up in RCTs (OR = 0.87 [0.64; 1.19]; I^2^ = 25%, k = 10, [moderate certainty evidence]) and in observational studies (OR = 0.04 [0.01; 0.1]; I^2^ = 13%, k = 2, [low certainty evidence]) (S13 Table, S5 Fig). A subgroup analysis revealed no significant differences in loss to follow-up across different country income levels (S17 Fig).

#### Treatment failure and death

None of the digital technology interventions was associated with reductions in treatment failure. (S14 Table, S8 Fig). However, the use of DATs was consistently associated with a small increase in the likelihood of death in RCTs (OR = 1.34 [1.18–1.53]; I^2^ = 0%, k = 24, [low certainty evidence]) and in cohort studies (OR = 1.16 [1.01–1.33]; I^2^ = 5%, k = 14, [low certainty evidence]) when results were stratified by study design. (S15 Table, S7 Fig) Among specific DAT types, the use of medication sleeves with phone call reminders (99DOTS) was also associated with increased mortality, both in RCTs (OR = 1.54 [1.04, 2.28; I^2^ = 51%, k = 3, [low certainty evidence]) and in observational studies (OR = 1.25 [1.1, 1.42; I^2^ = 0%, k = 2, [low certainty evidence]). Notably, clustering of participants could not be accounted for in this specific analysis.

#### Adverse events

The use of digital technologies was associated with an increase in the number of persons reporting adverse events (OR = 1.52 [1.26; 1.84]; I^2^ = 0%, k = 9, [moderate certainty evidence]). However, only RCTs (OR = 1.57 [1.25;1.97]; I^2^ = 12%; k = 6, [moderate certainty evidence]) suggested a significant increase in adverse event reporting, when considered separately. (S8a Fig) Subgroup analysis in RCTs indicated that VOT (OR = 1.90 [1.27; 2.84]; I^2^ = 0%, k = 3, [moderate certainty evidence]) was associated with an increased frequency of adverse event reporting. The combination of SMS and digital pillbox used together (OR = 1.91 [1.27; 2.88]; I^2^ = N/A, k = 1) also significantly increased the number of adverse events reported. (S16 Table, S8 Fig). While in most studies the number of adverse events corresponded to the number of persons who reported any adverse event [33, 53, 58, 61], in one study it referred specifically to persons for whom adverse events led to longer TB treatment duration [27], and in another study it referred to persons for whom adverse events led to treatment interruption [50]. Abdominal pain, nausea, and vomiting were the most common adverse events reported [58, 61].

#### Cure

Twenty-one studies reported on microbiologic cure: overall, DATs were not associated with an increase in cure. A small subgroup analysis suggested that feature-phone based interventions might be associated with more frequent microbiologic cure (OR = 1.30 [1.05; 1.62]; I^2^ = 0%, k = 2, [low certainty evidence]) (S9 Fig).

#### Microbiologic conversion

Ten studies assessed microbiologic conversion of sputum smears or cultures in persons with TB disease. Overall, there was no significant difference between the DAT intervention and comparison groups in RCTs. While nine studies addressed the proportion of persons with microbiologic conversion in the DAT group compared to standard care, one study [57] reported the mean time to culture conversion in the VOT group (47 days) vs. the standard of care group (48 days). (S17 Table, S10 Fig).

#### Completion of intensive phase treatment

Three studies reported on completion of intensive phase treatment for TB disease. None showed a significant association with DAT use (S11 Fig).

#### Emergence of antibiotic resistance

Two studies reported on emergence of anti-TB antibiotic resistance during treatment. Neither showed a significant association with DAT use. (S12 Fig).

### Publication bias

**Fig. 5:**
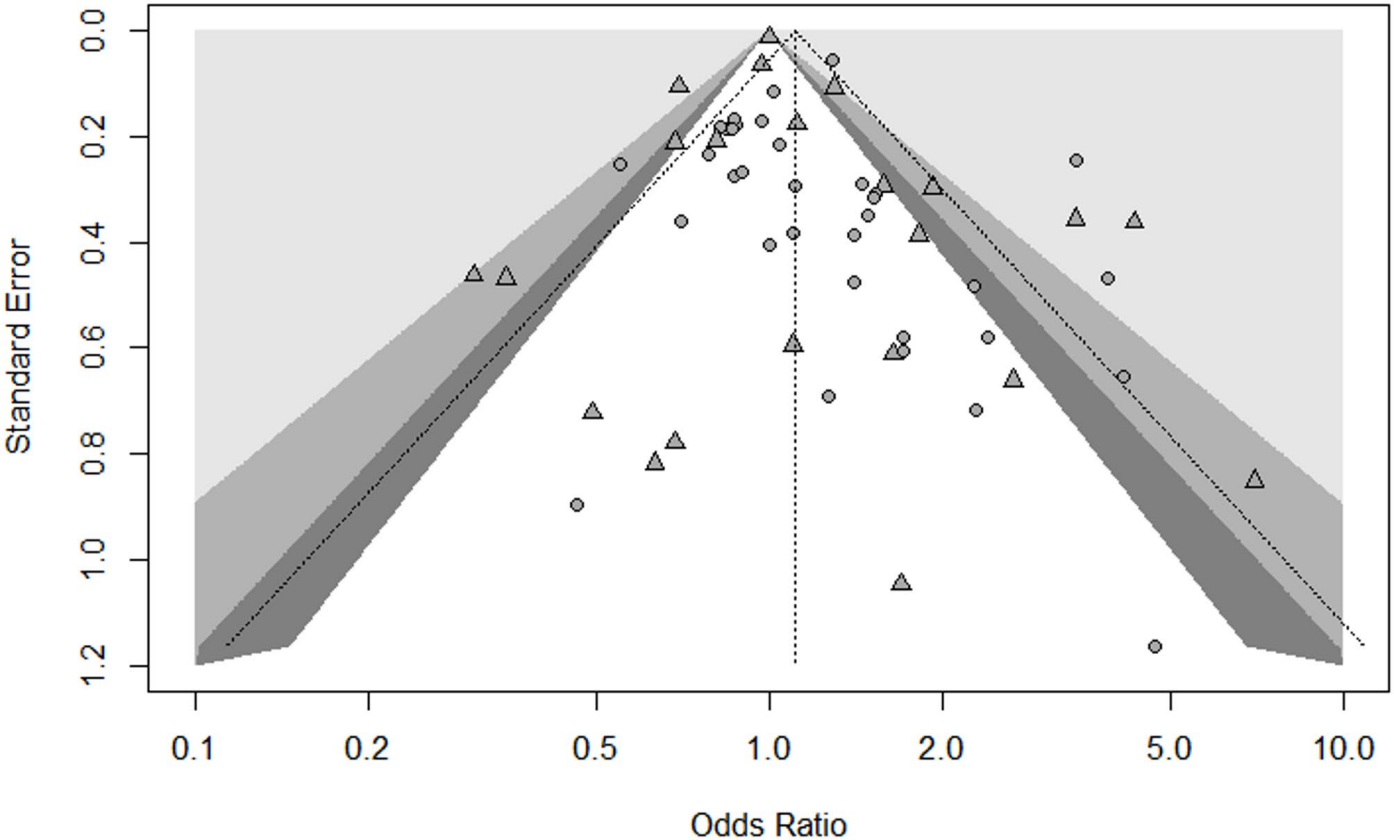
Funnel plot of included studies for the assessment of publication bias using Egger’s test. It shows studies comparing treatment success in patients using DATs versus standard care. Each point represents an individual study (● randomized controlled trials ▲ observational study). Varying levels of statistical significance of the studies are indicated by the shading; the unshaded region in the middle corresponds to p-values greater than 0.10, the dark gray-shaded region corresponds to p-values between 0.05 and 0.10, the medium gray-shaded region corresponds to p-values between 0.01 and 0.05, and the region outside of the funnel corresponds to p-values below 0.01

Publication bias was assessed using funnel plots for treatment success, loss to follow up, treatment failure and death. Egger’s test indicated asymmetry for treatment success (Egger’s β_0_ = 0.66 [0.16 to 1.16]; t_(44)_ = 2.58; *p* = 0.01) suggesting potential publication bias reflected by the relative paucity of smaller studies with negative findings. (Fig. 5) No publication bias was detected for loss to follow-up, treatment failure or death. (S13-15 Figs).

### Sensitivity analysis

A sensitivity analysis was conducted after excluding studies published as conference abstracts. Findings for the specific DATs were generally similar.

#### Short-term clinical outcomes in TB infection

##### Treatment completion

**Fig. 6:**
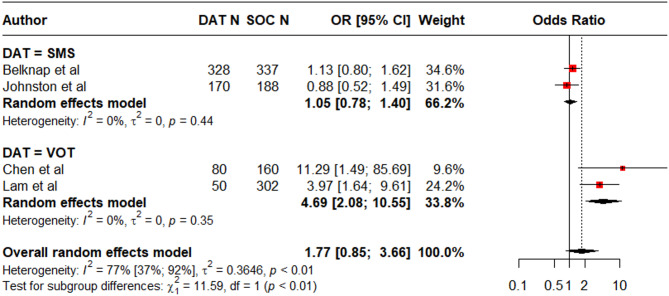
Forest plot showing treatment completion in DAT groups compared to standard of care among persons with TB infection

Four studies (1615 participants) addressed the use of DATs (2 SMS [32, 39], 2 VOT [49, 55]) in TB infection. Three were conducted exclusively in HIC and one included outpatient tuberculosis clinics in three HIC and one UMIC [32]. The definition of treatment completion varied. Some studies involving weekly isoniazid-rifapentine treatment defined it as ingestion of 11 doses within 16 weeks [32, 55], while another study required ingestion of at least 80% or 90% of planned medication doses within specified time intervals, i.e. ⩾80% or 90% of prescribed INH doses within 12 months, or ⩾80% or 90% of prescribed rifampin doses within 6 months [39]. Overall, DAT use was not associated with a significant increase in treatment completion. (S18 Table, Fig. 6). A subgroup analysis suggested that VOT was the only DAT associated with improved treatment completion. (OR = 4.69 [2.08, 10.55; I^2^ = 0%, k = 2, [low certainty evidence]).

##### Loss to follow up and adverse events

Results for loss to follow-up and adverse event reporting were inconsistent: one study suggested that VOT was associated with an increase in loss to follow-up [49], while another suggested that a two-way SMS-based intervention increased the number of adverse events reported [39].

### Long-term clinical outcomes

Two cohort studies [51, 53] and one RCT [69] examined the frequency of recurrence after treatment for TB disease. One cohort study suggested a lower rate of recurrence in the DAT group. The other two studies found no difference [51].

No study assessed death after treatment completion, or subsequent risk of TB disease among persons treated for TB infection.

### Adherence

There was substantial methodological heterogeneity among studies reporting on adherence. Key differences included the measurements used to assess adherence (S20 Fig), the interval over which adherence was measured, and the timing of the assessment. (S19 Table).

**Fig. 7:**
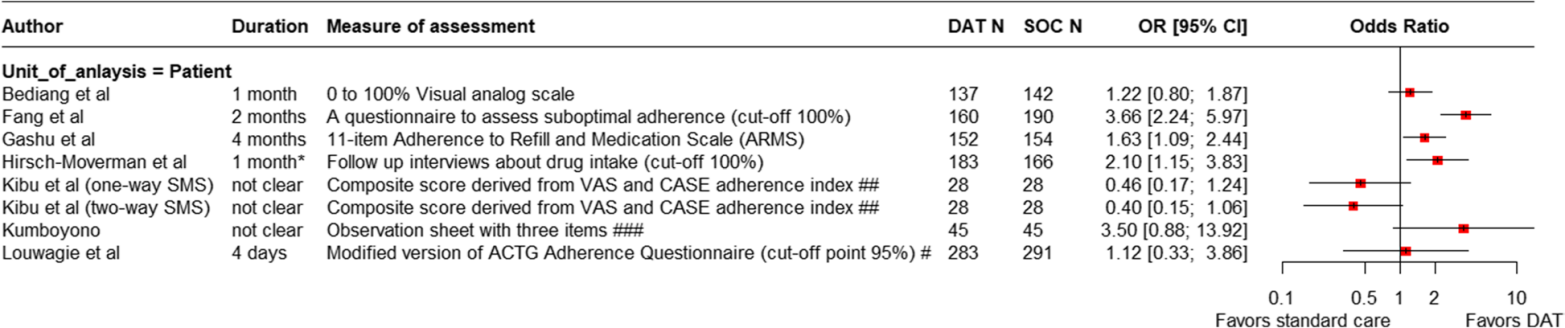
Forest plot showing self-reported adherence to TB treatment in SMS groups versus standard of care. * Adherence was assessed monthly in follow-up visits, # ACTG AIDS Clinical Trials Group. An adherence index was calculated using the 4-day recall formula [doses taken/doses prescribed], ## VAS Visual Analogue Scale; (CASE) Center for Adherence Support Evaluation, ### observation sheet including three items: the number of drugs consumed; the type of drugs; the time of drug consumption. Patients who satisfy all three subjects are considered adherent

Of 19 studies examining SMS interventions, 13 (68%) reported adherence measures. In eight, these reflected self-reports by persons on treatment. Three studies assessed adherence in terms of (timely) clinic attendance and one used pill counts. When compared to simultaneous urine tests for isoniazid metabolites, self-reports over-reported medication ingestion [43]. The association of SMS interventions with treatment adherence was inconsistent. For studies that used patients’ self-reports to measure adherence, the use of SMS-based interventions was not associated with a significant increase in adherence to treatment (Fig. 7).

**Fig. 8:**
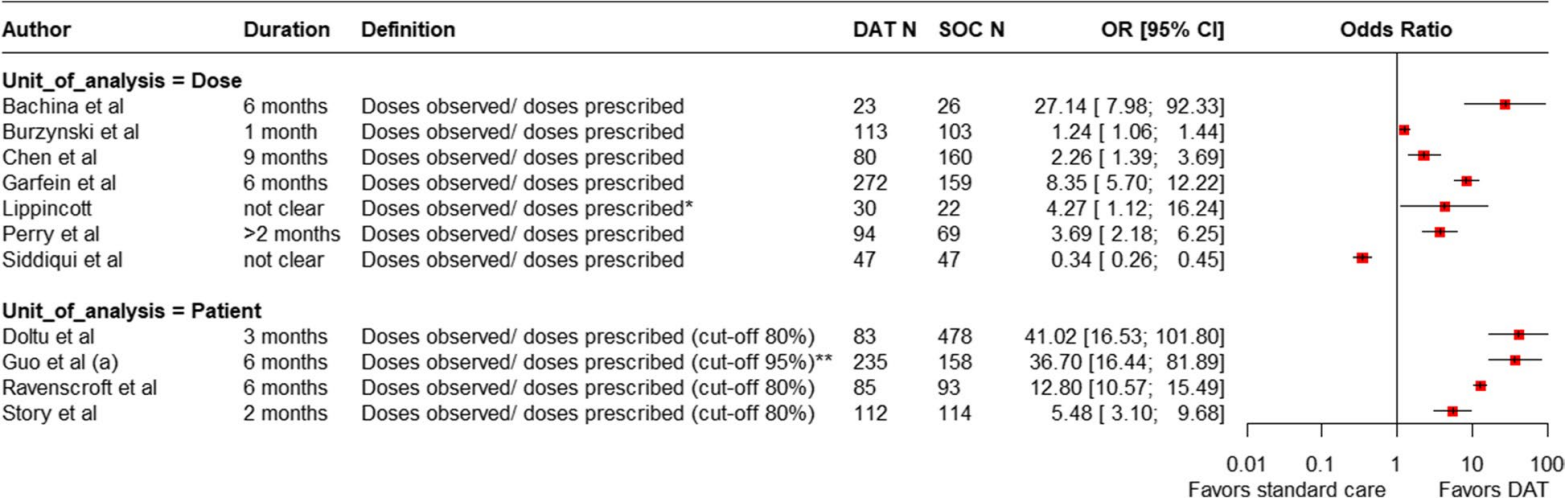
Forest plot showing comparing dose observation in VOT groups versus standard of care. * Prescribed doses that could not be confirmed as ingested were recorded to be ‘missed’ or ‘self-administered’ based on chart documentation, ** The numerator includes either directly observed or video-observed doses, excluding those obtained by self-report. The denominator includes the self-administered medications; it does not include periods when the treatment was suspended based on medical advice because of side effects

Of 17 studies reporting VOT interventions, 12 (71%) explicitly assessed treatment adherence, based on the videos reviewed, and usually described by the ratio of observed to prescribed doses. Some studies reported the proportion of doses, while others used categorical cut-offs of 80% or 95% to define adherence. Most associated VOT use with improved adherence (Fig. 8).

**Fig. 9:**
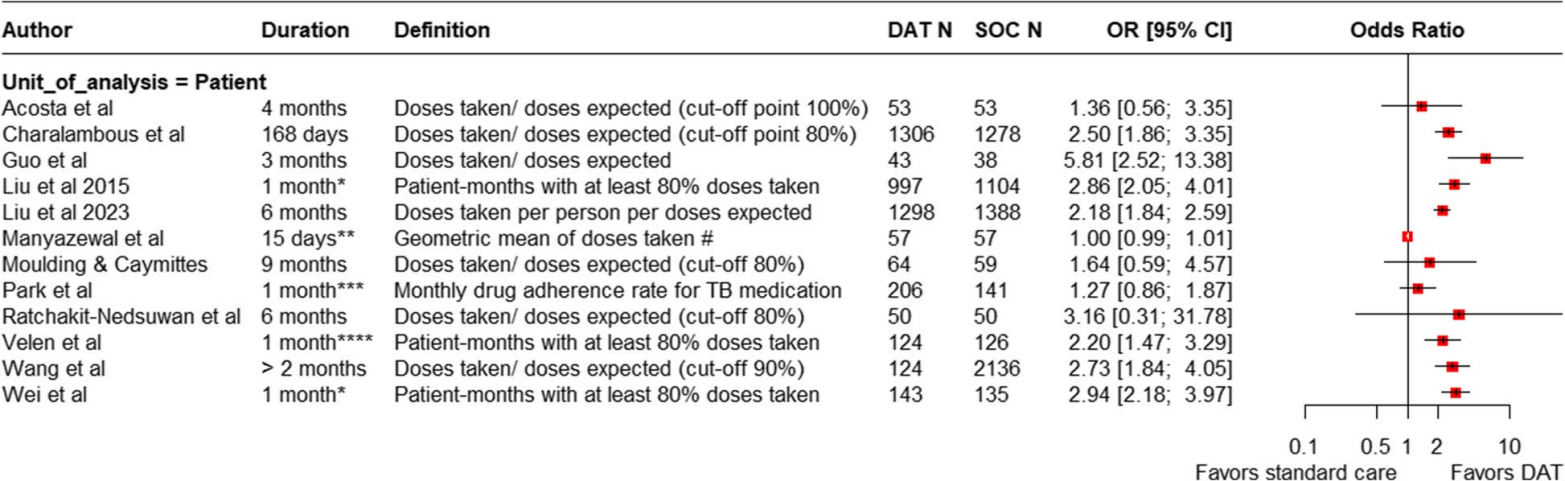
Forest plot showing adherence to TB medication dosing from digital pillbox data in digital pillbox group versus standard of care. * The study provided the percentage of patient-months on TB treatment where at least 20% of doses were missed. Adherence was measured for 6 months, ** Four 15-day appointments (intensive phase), *** The study provided the mean monthly drug adherence. Adherence was measured monthly for 6 months, **** Adherence was measured monthly for 6 months, # individual-level % adherence averaged over the 2-month intensive phase

Similarly, among 21 studies involving digital pillboxes, 15 (71%) assessed adherence of which 12 (57%) studies used the pillbox data (Fig. 9). Some studies also used complementary measures such as pill counts, self report by persons on treatment, or urine tests [27, 69, 70]. Most suggested that the use of the pillboxes was associated with improved adherence.

Feature-phone based interventions were generally associated with improved adherence [84, 85]. For interventions that used smartphone apps, results were inconsistent. While one study [94] suggested that the technology was associated with improved adherence among persons with multi-drug resistant TB, the two other studies found conflicting results among persons with drug-sensitive TB [91, 93].

### Patient-reported outcomes

#### Satisfaction

Satisfaction of people with TB with their care was assessed in two SMS studies [31, 36]. One assessed satisfaction with a Likert scale at the end of the sixth month on treatment [31]. In this study, satisfaction appeared high and similar between SMS and standard care groups based on two general questions. Another SMS study [36] assessed satisfaction with the provider–patient relationship using a more detailed 7-item questionnaire; satisfaction was significantly higher in the intervention group than in the control group. (S20 Table).

Three VOT studies [54, 58, 61] used surveys to measure satisfaction among persons with TB; all reported greater satisfaction among participants in the VOT groups.

Only one study [71] reported satisfaction of persons with TB with the digital pillbox, using the Treatment Satisfaction Questionnaire for Medication (TSQM). It includes 14 questions across four domains: Effectiveness, Convenience, Side Effects, and Global Satisfaction. Satisfaction was significantly greater in the DAT group than in the control group in all domains assessed. (S20 Table).

#### Health related quality of life

Four studies (one SMS, one VOT, and two digital pillbox studies) assessed health related quality of life (HRQOL) among persons with TB disease [61, 72, 77, 84], while one SMS study evaluated HRQOL in persons with TB infection [39]. All five studies used versions of the EuroQol questionnaires and found no differences between participants in the DAT and standard care groups.

#### Stigma

One study measured TB-related stigma from the patient and family perspectives using the Van Rie stigma scale [84]. It did not identify any significant difference in stigma reported by persons with TB and their families supported by a feature-phone intervention vs. those who received standard care.

#### Special populations

Other prespecified stratified analyses involving children, persons living with HIV, those with drug resistant TB disease, and those with extrapulmonary TB disease were not conducted as no sufficiently homogeneous studies were found for each subgroup.

#### Non-inferiority studies

We identified four studies employing non-inferiority designs that evaluated different DATs compared to standard of care. One study found that phone-based interventions and a people-centred treatment approach can be less expensive, more flexible and as effective as DOT within the specified non-inferiority margin [84]. Another SMS-based intervention study, the iAdhere study, reported high treatment completion rates in TB infection using self-administered INH and rifapentine in the U.S., Spain, and Hong Kong, suggesting feasibility where DOT is impractical. A randomized crossover trial conducted in New York City from 2017 to 2019, where patients were given the choice between using synchronous or asynchronous VOT compared to DOT, showed that VOT was as effective as DOT in ensuring adherence, which led to adoption of VOT by the NYC TB program during the COVID-19 pandemic [48]. The evriMED500 study demonstrated that managing patients using digital pillboxes for 15 days led to superior treatment satisfaction and usability compared to daily in-clinic- DOT [71].

## Discussion

Our systematic review assessed the impact of digital technologies on adherence, treatment outcomes and patient-reported outcomes among persons treated for TB disease and infection. We included recent randomized and quasi-experimental studies, as well as cohort studies, from diverse country settings.

Our review suggests that digital adherence technologies, taken together, were associated with modest improvements or were as effective as standard of care in some TB disease outcomes. There was also some evidence of enhanced adverse event reporting. These findings are concordant with previous reviews [8, 14, 96]. For some clinical outcomes, there was generally no significant impact of DATs. They may improve efficiency and may be preferred by persons with TB as they can offer reasonable alternatives to traditional DOT. This is particularly relevant given that certain DATs demonstrated more consistent cost savings when compared to clinic- or field-based DOT, primarily due to reduced travel and labor costs for healthcare workers, as well as less productivity loss for treated individuals [97]. From the program standpoint, cost savings allow treatment support for more persons with TB.

The digital technologies we reviewed are diverse, and can potentially support adherence in different ways. While some technologies only relayed medication reminders, others were combined with differentiated care pathways, based on patient engagement with the DAT as a proxy for treatment adherence. Subgroup analyses suggested that some digital technologies, notably those involving video observation and smartphone apps, were more likely associated with small improvements in treatment success. They may also enhance adverse event reporting. Similarly, VOT was associated with improved adherence to expected dosing. This differs from two recent reviews which found no effect of VOT on treatment completion [6, 16] although one suggested that VOT was associated with improved dosing adherence [16]. Of note, these reviews included fewer studies than ours.

We emphasize that VOT and smartphone apps were overwhelmingly evaluated in high- and upper-middle-income countries. Hence the generalizability of these findings to low- and lower-middle-income country settings, where most people with TB live, may be limited. Low- and lower-middle-income countries may face greater challenges in mobile coverage and accessibility, and in health system capacity to implement DATs [98].

Indeed, SMS-based interventions and medication sleeves with phone calls (99DOTS), which were largely implemented in low -and middle income countries settings, were not consistently associated with improved adherence or clinical outcomes. In fact, 99DOTs may have been associated with an increased frequency of death, raising the possibility of gaps in patient supervision and support [89]. However, this result should be interpreted with caution as the effect of confounders was not adjusted for. Clustering was not considered in one of the 99DOTS studies. It is also possible that deaths appeared more frequent if there were fewer losses to follow-up, as the latter are a key competing risk.

Digital pillbox-based interventions, which were also mostly implemented in LMIC settings, also had variable associations with clinical outcomes. While digital pillboxes were associated with increased medication adherence, these findings were often reported from studies that compared adherence data from a “silent” pillbox (i.e., without reminders) in the control arm to a pillbox that provided audiovisual reminders in the intervention arm. This approach may increase risk of detection bias, as people receiving audiovisual reminders may be more inclined to keep their medication blister packs within the device, thereby increasing engagement with the DAT. Earlier studies and a systematic review have highlighted limits to the accuracy of dose reports with 99DOTS and reveal considerable variability in the accuracy of dose reports from digital pillboxes [99–102].

The increased reporting of adverse events is likely a positive consequence of more consistent opportunities for contact with providers. In many VOT studies, persons with TB were routinely encouraged to report any side effects before swallowing the pills [50]. This was sometimes done as a safety precaution, to address concerns that reduced face-to-face contact might lead to side effects being undetected [61, 103]. The increased reporting of adverse events could also be a reflection of the satisfaction of the individuals with the way their treatment is observed [49, 61]. People with TB using DATs have specifically highlighted their satisfaction with their ability to report adverse events to providers [61]. Reporting of adverse events is important to manage potential complications, and to address concerns that may affect adherence to treatment [58].

Importantly, many of the VOT and digital pillbox studies also included co-interventions to enhance patient support, beyond dose monitoring. Examples include medication reminders [47, 52–57, 61, 80], encouraging adverse event reporting [50, 52–54, 58, 61] and/or escalation via SMS, phone calls, or home visits if expected video observation was missed. Some VOT interventions were coupled with motivation, al SMS, reimbursement of mobile data costs, payment for visit attendance, or nutritional allowances [48, 49, 52, 58, 61]. Similarly, for interventions that involved smartphone apps, most studies described various combinations of associated measures e.g., medication reminders, adverse event reporting, instant messaging, chat groups with providers and/or other patients [13, 92, 93].

The diversity of comparison strategies reported as the “standard of care” further complicates interpretation and inference. While most studies indicated daily provider-administered DOT (facility-or field-based) as the comparator, others used family DOT or self-administered treatment, a combination of approaches, or simply did not specify.

Unfortunately, no study evaluated deaths post-treatment, and most did not report on recurrent TB disease or emergence of drug resistance at any time.

Strengths of this review include a comprehensive and updated search by a health librarian, which ultimately yielded a large number of included studies (n = 76), with many participants (total 86,586) in diverse settings, permitting more detailed, stratified analyses. Our comprehensive search included gray literature, non–English language studies, and studies indexed in other databases. We also assessed the potential association of DATs with a variety of outcomes. Meta-analyses were conducted and possible reasons for heterogeneity were investigated.

However, our review has several limitations. As emphasized previously, the generalizability of findings regarding VOT and smartphone apps is limited, with respect to lower-income settings where TB burden is highest, yet where these technologies and the necessary technical and human resource infrastructure may be least accessible. We do report other robust findings from studies in LMICs: the DAT types implemented in these settings (e.g., SMS, 99DOTS, digital pillboxes) had variable or limited impact on treatment outcomes, which has important policy implications.

There was also substantial heterogeneity in study findings, which is not surprising given the diversity of study design, study settings, methodologies, technologies and comparators considered. This limited our ability to formally pool and meta-analyze data and made some quantitative findings less robust. Furthermore, performance bias was present in most randomized controlled trials as it was not feasible to mask participants due to the nature of the DAT intervention.

We could not assess treatment success and treatment completion as separate outcomes: some studies used the two terms interchangeably. While the treatment success category includes both persons with microbiologic cure and those who completed treatment without known microbiologic failure, most studies did not report microbiologic cure.

We estimated odds ratios for some short-term clinical outcomes from crude event numbers reported in cluster randomized trials, as no adjusted estimates were provided. This may have resulted in artificially narrow confidence intervals when clustering was ignored.

We focused on evidence from RCTs, quasi-experimental studies and cohort studies. For this reason, cross-sectional studies which assessed patient-reported outcomes—often without a comparison group—were excluded. More generally, there was very limited evidence around the use of DATs in persons with drug-resistant TB, in children, or in persons living with HIV.

Finally, our review and analysis provide a comprehensive view of the literature through March 2024. Unfortunately, for logistical reasons, we were unable to update our search beyond that date.

## Conclusion

While the evidence base remains incomplete, and generalizability of studies often limited, this systematic review nonetheless synthesizes relevant information and identifies further evidence gaps. It provides insights into how some DATs can be successfully used to support curative and preventive TB treatment. VOT and smartphone apps appear to be associated with improvement in treatment success in high-income settings and could be considered as alternatives to DOT when appropriate. SMS, and other feature-phone based interventions did not improve treatment success or had highly variable findings, with most studies conducted in LMIC settings. Although in many cases DATs did not improve clinical outcomes, they may improve efficiency and may be preferred by persons with TB as they can offer reasonable alternatives to traditional DOT. This finding has important policy implications and raises questions regarding investments in the DATs as currently designed and implemented. With the rapid growth of technology and mobile usage worldwide, programs, patients, and providers will increasingly look to digital technologies for support. This raises the question of whether DATs perform as well as DOT. While our pooled results suggest similar treatment success rates, this review does not establish non-inferiority.

The combination and fine-tuning of different support interventions, with a more personalized delivery approach, is promising. However, there remain substantial knowledge gaps with respect to key populations and to longer-term health outcomes. Beyond issues of generalizability to settings and populations which bear the highest TB burden, there also remain important questions related to implementation and logistics. Further research is urgently needed to refine DATs, to inform non-DAT-based approaches to improving TB treatment outcomes in LMICs, and to use non-inferiority designs in comparisons of DATs with the standard of care. More studies on technologies like SMS, and other feature-phone-based interventions -which have shown highly variable results—are necessary to better assess their effectiveness. Investigating VOT in previously less feasible settings or further examining its impact on TB preventive treatment support could provide valuable insights. Qualitative studies are also crucial to understand why some technologies are associated with increased adverse event reporting compared to others. Additionally, research on patient satisfaction and other patient-reported outcomes should be conducted using validated tools to ensure the reliability of these measures.

## Supplementary Information

Supplementary Material 1

## Data Availability

All data not shared in the main manuscript and its supplement will be available in a public repository at https://borealisdata.ca/dataverse/mcgill

## Abbreviations

AFR: African Region
AMR: Region of the Americas
DATs: Digital adherence technologies
DOT: Directly observed therapy
EMR: Eastern Mediterranean Region
EUR: European Region
GRADE: Grades of Recommendation, Assessment, Development, and Evaluation
HIC: High income countries
HRQoL: Health-related quality of life
LIC: Low-income countries
LMIC: Lower-middle income countries
LTFU: Loss to follow-up
OR: Odds ratio
PRISMA: Preferred Reporting Items for Systematic Reviews and Meta-Analyses
RCT: Randomized controlled trial
RR: Risk ratio
SAT: Self- administered therapy
SEAR: South-East Asian Region
SMS: Short messaging service
TSQM: Treatment Satisfaction Questionnaire for Medication
UMIC: Upper-middle income countries
VOT: Video-observed therapy
WPR: Western Pacific Region
