# Supplementary material for: The impact of digital adherence technologies on treatment outcomes, adherence, and patient-reported outcomes in tuberculosis: a systematic review and meta-analysis
Source: BMC Infect Dis. 2025 Oct 14;25:1314. doi: 10.1186/s12879-025-11503-3 (PMC12523041; doi:10.1186/s12879-025-11503-3)
Supplement: Supplementary file 1 — Supplementary Material 1 [file 12879_2025_11503_MOESM1_ESM.pdf]

Supplementary material

Contents

Search strategy 5
MEDLINE (Ovid) 5
Embase (Ovid) 6
CINAHL (EBSCOhost) 6
CENTRAL (Cochrane Library/Wiley) 8
WOS.SCI,WOS.ISTP,WOS.ESCI (Web of Science Core Collection) 9
MedRxiv and other preprints via Europe PMC 10
Working definition 12
Definitions of short-term clinical outcomes 13
Quality assessment tools 14
Risk of bias assessment tool- randomized controlled trials 14
Newcastle - Ottawa quality assessment scale- cohort studies 16
Summary of quality assessment evaluation 34
Grading evidence 38
Quantitative synthesis of evidence- TB disease 48
Treatment success 48
Loss to follow up 51
Treatment failure 54
Death 57
Adverse events 60
Cure 61
Microbiologic conversion 64
Completion of intensive phase 65
Emergence of anti-TB drug resistance 66
Quantitative synthesis of evidence- TB infection 74
Subgroup analysis based on country income level 75
S1 Meta-regression (country income level) 82
S2 Meta-regression (Study design) 83
Evidence on patient-reported outcomes 94
References 97

List of Tables

Table S1 Search strategy 5

Table S2 Case definition of digital adherence technology (DAT) 12

Table S3 Definitions of short-term clinical outcomes 13

Table S4 Cochrane Risk of bias assessment tool for randomized controlled trials[2] 14

Table S5 Cochrane Risk of bias assessment tool for randomized controlled trials 16

Table S6 Characteristics of included studies 17

Table S7 Characteristics of study participants and intervention in included studies 22

Table S8 Reported outcomes in included studies 31

Table S9 Risk of bias in included observational studies with estimated overall risk of bias 35

Table S10 Risk of bias in included observational studies 36

Table S11a Grading the evidence from RCTs 38

Table S11b Grading the evidence from observational studies 43

Table S12 Pooled estimates for treatment success in TB disease stratified by type of DAT, subtype of DAT and country-income-level. Evidence is pooled from RCTs and observational studies 67

Table S13 Pooled estimates for loss to follow up stratified by type of DAT, subtype of DAT and country-income-level Evidence is pooled from RCTs and observational studies 68

Table S14 Pooled estimates for treatment failure in TB disease stratified by type of DAT, subtype of DAT and country-income-level Evidence is pooled from RCTs and observational studies 69

Table S15 Pooled estimates for death in TB disease stratified by type of DAT, subtype of DAT and country-income-level. Evidence is pooled from RCTs and observational studies 70

Table S16 Pooled estimates for reporting of adverse events in TB disease stratified by type of DAT, subtype of DAT and country-income-level. Evidence is pooled from RCTs and observational studies 71

Table S17 Pooled estimates for microbiologic conversion stratified by type of DAT. Evidence is pooled from RCTs and observational studies 71

Table S18 Pooled estimates for treatment completion in l LTBI 74

Table S19 Qualitative summary of measures of adherence and estimated adherence in individual studies 85

Table S20 Qualitative summary of patient-reported outcomes in DAT-groups and standard of care groups 94

List of Figures

Figure S1 Risk of bias summary: review authors' judgements about each risk of bias item for each included study 35

Figure S2 Risk of bias graph: review authors' judgements about each risk of bias item presented as percentages across all included studies. 35

Figure S3 Risk of bias graph: review authors' judgements about each risk of bias item presented as percentages across included observational studies 37

Figure S4a Forest plot showing treatment success in DAT groups compared to standard of care stratified by study design 48

Figure S4b Forest plot showing treatment success in DAT groups compared to standard of care (RCTs) 49

Figure S4c Forest plot showing treatment success in DAT groups compared to standard of care (observational studies) 50

Figure S5a Forest plot showing loss to follow up in DAT groups compared to standard of care stratified by study design 51

Figure S5b Forest plot showing loss to follow up in DAT groups compared to standard of care (RCTs) 52

Figure S5c Forest plot showing loss to follow up in DAT groups compared to standard of care (observational studies) 53

Figure S6a Forest plot showing treatment failure in DAT groups compared to standard of care stratified by study design 54

Figure S6b Forest plot showing treatment failure in DAT groups compared to standard of care (RCTs) 55

Figure S6c Forest plot showing treatment failure in DAT groups compared to standard of care (observational studies) 56

Figure S7a Forest plot showing death during treatment in DAT groups compared to standard of care stratified by study design 57

Figure S7b Forest plot showing death during treatment in DAT groups compared to standard of care (RCTs) 58

Figure S7c Forest plot showing death during treatment in DAT groups compared to standard of care (observational studies) 59

Figure S8a Forest plot showing adverse events reported in DAT groups compared to standard of care stratified by study design 60

Figure S8b Forest plot showing adverse events reported in DAT groups compared to standard of care (RCTs) 60

Figure S8c Forest plot showing adverse events reported in DAT groups compared to standard of care (observational studies) 61

Figure S9a Forest plot showing cure rate in DAT groups compared to standard of care stratified by study design 61

Figure S9b Forest plot showing cure rate in DAT groups compared to standard of care stratified by study design (RCTs) 62

Figure S9c Forest plot showing cure rates in DAT groups compared to standard of care (observational studies) 63

Figure S10a Forest plot showing microbiologic conversion of sputum smear in DAT groups compared to standard of care stratified by study design 64

Figure S10b Forest plot showing microbiologic conversion of sputum smear in DAT groups compared to standard of care (RCTs) 64

Figure S10c Forest plot showing microbiologic conversion of sputum smear in DAT groups compared to standard of care (observational studies) 65

Figure S11a Forest plot showing completion of intensive phase treatment in DAT groups compared to standard of care stratified by study design 65

Figure S11b Forest plot showing completion of intensive phase treatment in DAT groups compared to standard of care (RCTs)........65

Figure S11c Forest plot showing completion of intensive phase treatment in DAT groups compared to standard of care (observational studies) 66

Figure S12 Forest plot showing emergence of anti-TB drug resistance in DAT groups compared to standard of care 66

Figure S13 Funnel plot of included studies for the qualitative assessment of publication bias using Egger's test. It shows studies comparing LTFU in patients using DATs versus standard care. Each point represents an individual study.................................72

Figure S14 Funnel plot of included studies for the qualitative assessment of publication bias using Egger's test. It shows studies comparing treatment failure in patients using DATs versus standard care. Each point represents an individual study 72

Figure S15 Funnel plot of included studies for the qualitative assessment of publication bias using Egger's test. It shows studies comparing death during treatment in patients using DATs versus standard care. Each point represents an individual study.....73

Figure S16a Forest plot showing treatment success with DATs compared to standard of care stratified by country income level (RCTs) 75

Figure S16 Forest plot showing treatment success with DATs compared to standard of care stratified by country income level (observational studies) 76

Figure S17a Forest plot showing losses to follow-up with DATs compared to standard of care stratified by country income level (RCTs)...77

Figure S17b Forest plot showing losses to follow-up with DATs compared to standard of care stratified by country income level (observational studies) 78

Figure S18a Forest plot showing treatment failure with DATs compared to standard of care stratified by country income level (RCTs) 79

Figure S18b Forest plot showing treatment failure with DATs compared to standard of care stratified by country income level (observational studies) 80

Figure S19a Forest plot showing death during treatment with DATs compared to standard of care stratified by country income level (RCTs)...81

Figure S19b Forest plot showing death during treatment with DATs compared to standard of care stratified by country income level (observational studies) 82

Figure S20 Measure of assessment of adherence to TB treatment stratified by DAT-type...84

Search strategy

Table S1 Search strategy

MEDLINE (Ovid)

Ovid MEDLINE(R) ALL <1946 to March 01, 2024>

1 exp tuberculosis/ or mycobacterium tuberculosis/ 231446
2 Antitubercular Agents/ 42051
3 (Tuberculosis or Kochs disease or Phthisis or TB or MTB or MDRTB or XDRTB or DRTB or LTBI or directly observed treatment short course).mp. 309723
4 1 or 2 or 3 [TB CONCEPT] 315180
5 ((antitubercular agents/ or directly observed therapy/ or medication adherence/ or patient compliance/ or "treatment adherence and compliance"/) and technology/) or mobile applications/ or internet/ or cell phone/ or smartphone/ or text messaging/ or computer, handheld/ or telemedicine/ or therapy, computer-assisted/ or medical informatics applications/ 153813
6 (((digital* or electronic* or mobile or wireless* or virtual*) adj2 (adherence or medication monitor* or medication package* or observ*)) or digital technolog* or technology-based or Digital health or eHealth or e health or mhealth or m health or SMS or reminder* or short messag* service* or text messag* or MMS or multimedia messag* or MEMS or (monitor* adj2 (electronic* or sensor* or device*)) or Webcam* or web cam* or smartphone* or smart phone* or web based or health IT or health ICT or ((cell* or mobile) adj1 (device* or health or phone* or technolog*)) or video* or cellphone* or feature phone* or vdot or vmalt or tele* or dat or wot or 99dots or 99 dots or monitoring system* or ingestible sensor* or merm or artificial intelligence or ai or vot or ((digital or smart) adj (pill box* or pillbox*)) or VST).mp. 763889
7 5 or 6 [DAT CONCEPT] 832284
8 4 and 7 [TB CONCEPT AND DAT CONCEPT] 3516
9 limit 8 to yr="2000 -Current" 3088
10 health behavior/ or exp patient acceptance of health care/ or exp Outcome Assessment, Health Care/ or exp "Treatment Adherence and Compliance"/ or "Value of Life"/ or Quality-Adjusted Life Years/ or exp health status indicators/ 1921252
11 (adher* or adverse or complet* or complian* or conversion* or cure* or death* or default or dose* or drug resistan* or fail* or fatal* or mortality or morbidity or outcome* or relaps* or recur* or (risk adj5 disease) or scor* or severity or side effect* or status or surviv*).mp. 13231674
12 (loss to follow up or loss to followup or lost to follow up or lost to followup).mp. 29727
13 activities of daily living/ or functional status/ or Patient Reported Outcome Measures/ or quality of life/ or social stigma/ or (health state* or hrql or hrqol or patient experience* or patient reported or preference* or pro$1 or prom$1 or qol or quality of life or respiratory questionnaire* or satisf* or SGRQ or stigma* or visual analog* scale*).mp. 1671880
14 (((disability or quality) adj1 adjusted life) or qald* or qale* or qtime* or life year* or daly* or sf 36 or sf36 or short form 36 or shortform 36 or short form36 or shortform36 or sf thirtysix or sfthirtysix or sfthirty six or sf thirty six or shortform thirtysix or shortform thirty six or short form thirtysix or short form thirty six or sf6 or sf 6 or short form 6 or shortform 6 or sf six or sfsix or shortform six or short form six or shortform6 or short form6 or sf8 or sf 8 or sf eight or sfeight or shortform 8 or shortform 8 or shortform8 or short form8 or shortform eight or short form eight or sf12 or sf 12 or short form 12 or shortform 12 or short form12 or shortform12 or sf twelve or sftwelve or shortform twelve or short form twelve or sf16 or sf 16 or short form 16 or shortform 16 or short form16 or shortform16 or sf sixteen or sfsixteen or shortform sixteen or short form sixteen or sf20 or sf 20 or short form 20 or shortform 20 or short form20 or shortform20 or sf twenty or sftwenty or shortform twenty or short form twenty or hql or hqol or h qol or hr qol or hye or hyes or (health* adj2 year* adj2 equivalent*) or pqol or qls or quality of wellbeing or quality of well being or index of wellbeing or index of well being or qwb or nottingham

health profile* or sickness impact profile* or (health adj3 utilit*) or (utilit* adj3 (valu* or measur* or health or life or estimat* or elicit* or disease or weight)) or (preference* adj3 (valu* or measur* or health or life or estimat* or elicit* or disease or instrument or instruments)) or disutilit* or rosser or willingness to pay or standard gamble* or (time adj (trade off* or tradeoff*)) or tto or hui or hui1 or hui2 or hui3 or eq or euroqol or euro qol or eq5d or eq 5d or euroqual or euro qual or duke health profile or (health adj3 assess*)).mp. 314839
15 10 or 11 or 12 or 13 or 14 [HEALTH OUTCOMES CONCEPT] 13949792
16 9 and 15 [TB CONCEPT AND DAT CONCEPT AND HEALTH OUTCOMES CONCEPT] 1990

https://proxy.library.mcgill.ca/login?url=https://ovidsp.ovid.com/ovidweb.cgi?T=JS&NEWS=N&PAGE=main&SHAREDSEARCHID=zWr2m4jx2QZxRivVP8txKa8pupKfKY7yTj2Hox2KUYhQaMmmTBBMXwqOtn3W0ScD

Embase (Ovid)

CINAHL (EBSCOhost)

#	Query	Limiters/Expanders	Results
S15	S9 AND S14	Expanders - Apply equivalent subjects Search modes - Boolean/Phrase	445
S14	S10 OR S11 OR S12 OR S13	Expanders - Apply equivalent subjects Search modes - Boolean/Phrase	3,114,513
S13	(((disability OR quality) N1 "adjusted life") OR qald* OR qale* OR qtime* OR "life year*" OR daly* OR "sf 36" OR sf36 OR "short form 36" OR "shortform 36" OR "short form36" OR shortform36 OR "sf thirtysix" OR sfthirtysix OR "sfthirty six" OR "sf thirty six" OR "shortform thirtysix" OR "shortform thirty six" OR "short form thirtysix" OR "short form thirty six" OR sf6 OR "sf 6" OR "short form 6" OR "shortform 6" OR "sf six" OR sfsix OR "shortform six" OR "short form six" OR shortform6 OR "short form6" OR sf8 OR "sf 8" OR "sf eight" OR sfeight OR "shortform 8" OR "shortform 8" OR shortform8 OR "short form8" OR "shortform eight" OR "short form eight" OR sf12 OR "sf 12" OR "short form 12" OR "shortform 12" OR "short form12" OR shortform12 OR "sf twelve" OR sftwelve OR "shortform twelve" OR "short form twelve" OR sf16 OR "sf 16" OR "short form 16" OR "shortform 16" OR "short form16" OR shortform16 OR "sf sixteen" OR sfsixteen OR "shortform sixteen" OR "short form sixteen" OR sf20 OR "sf 20" OR "short form 20" OR "shortform 20" OR "short form20" OR shortform20 OR "sf twenty" OR sftwenty OR "shortform twenty" OR "short form twenty" OR hql OR hqol OR "h qol" OR "hr qol" OR hye OR hyes OR (health* N2 year* N2 equivalent*) OR pqol OR qls OR "quality of wellbeing" OR "quality of well being" OR "index of wellbeing" OR "index of well being" OR qwb OR "nottingham health profile*" OR "sickness impact profile*" OR (health N3 utilit*) OR (utilit* N3 (valu* OR measur* OR health OR life OR estimat* OR elicit* OR disease OR weight)) OR (preference* N3 (valu* OR measur* OR health OR life OR estimat* OR elicit* OR disease OR instrument OR instruments)) OR disutilit* OR rosser OR "willingness to pay" OR "standard gamble*" OR (time W1 ("trade off*" OR tradeoff*)) OR tto OR hui OR hui1 OR hui2 OR hui3 OR eq OR euroqol OR "euro qol" OR eq5d OR "eq 5d" OR euroqual OR "euro qual" OR "duke health profile" OR (health N3 assess*))	Limiters - Publication Date: 20000101-20240331 Expanders - Apply equivalent subjects Search modes - Boolean/Phrase	75,800
S12	("loss to follow up" OR "loss to followup" OR "lost to follow up" OR "lost to followup")	Limiters - Publication Date: 20000101-	6,926

		20240331 Expanders - Apply equivalent subjects Search modes - Boolean/Phrase	
S11	(adher* OR adverse OR complet* OR complian* OR conversion* OR cure* OR death* OR default OR dose* OR "drug resistan*" OR fail* OR fatal* OR mortality OR morbidity OR outcome* OR relaps* OR recur* OR (risk N5 disease) OR scor* OR severity OR "side effect*" OR status OR surviv*)	Limiters - Publication Date: 20000101-20240331 Expanders - Apply equivalent subjects Search modes - Boolean/Phrase	3,064,077
S10	(MH "Health Behavior") OR (MH "Patient Compliance+") OR (MH "Treatment Refusal") OR (MH "Outcomes (Health Care)") OR (MH "Outcome Assessment") OR (MH "Patient-Reported Outcomes+") OR (MH "Treatment Outcomes+") OR (MH "Disability-Adjusted Life Years") OR (MH "Quality-Adjusted Life Years") OR (MH "Health Status Indicators+")	Limiters - Publication Date: 20000101-20240331 Expanders - Apply equivalent subjects Search modes - Boolean/Phrase	675,497
S9	S4 AND S7	Limiters - Publication Date: 20000101-20240331 Expanders - Apply equivalent subjects Search modes - Boolean/Phrase	699
S8	S4 AND S7	Expanders - Apply equivalent subjects Search modes - Boolean/Phrase	739
S7	S5 OR S6	Expanders - Apply equivalent subjects Search modes - Boolean/Phrase	342,220
S6	(((digital* OR electronic* OR mobile OR wireless* OR virtual*) N2 (adherence OR "medication monitor*" OR "medication package*" OR observ*)) OR "digital technolog*" OR technology-based OR "Digital health" OR eHealth OR "e health" OR mhealth OR "m health" OR SMS OR reminder* OR "short messag* service*" OR "text messag*" OR MMS OR "multimedia messag*" OR MEMS OR (monitor* N2 (electronic* OR sensor* OR device*)) OR Webcam* OR "web cam*" OR smartphone* OR "smart phone*" OR "web based" OR "health IT" OR "health ICT" OR ((cell* OR mobile) N1 (device* OR health OR phone* OR technolog*)) OR video* OR cellphone* OR "feature phone*" OR vdot OR vmalt OR tele* OR dat OR wot OR 99dots OR "99 dots" OR "monitoring system*" OR "ingestible sensor*" OR merm OR "artificial intelligence" OR ai OR vot OR ((digital OR smart) W1 ("pill box*" OR pillbox*)) OR VST)	Expanders - Apply equivalent subjects Search modes - Boolean/Phrase	273,561
S5	((MH "Antitubercular Agents") OR (MH "Directly Observed Therapy") OR (MH "Medication Compliance") OR (MH "Patient Compliance")) AND (MH "Technology")) OR (MH "Wireless Communications") OR (MH "Mobile Applications") OR (MH "Internet") OR (MH "Internet-Based Intervention") OR (MH "Cellular Phone+") OR (MH "Text Messaging+") OR (MH "Instant Messaging") OR (MH "Interactive Voice Response Systems") OR (MH "Videoconferencing+") OR (MH "Computers, Hand-Held+") OR (MH "Telehealth+") OR (MH "Digital Technology") OR (MH "Therapy, Computer Assisted") OR (MH "Drug Therapy, Computer Assisted") OR (MH "Medical Informatics")	Expanders - Apply equivalent subjects Search modes - Boolean/Phrase	137,327

S4	S1 OR S2 OR S3	Expanders - Apply equivalent subjects<br>Search modes - Boolean/Phrase	36,305
S3	(Tuberculosis OR "Kochs disease" OR Phthisis OR TB OR MTB OR MDRTB OR XDRTB OR DRTB OR LTBI OR "directly observed treatment short course")	Expanders - Apply equivalent subjects<br>Search modes - Boolean/Phrase	35,973
S2	(MH "Antitubercular Agents")	Expanders - Apply equivalent subjects<br>Search modes - Boolean/Phrase	4,928
S1	(MH "Tuberculosis+") OR (MH "Mycobacterium Tuberculosis")	Expanders - Apply equivalent subjects<br>Search modes - Boolean/Phrase	26,786

CENTRAL (Cochrane Library/Wiley)

Search Name:
Date Run: 04/03/2024 19:23:47
Comment:

ID Search Hits
#1 (Tuberculosis or "Kochs disease" or Phthisis or TB or MTB or MDRTB or XDRTB or DRTB or LTBI or "directly observed treatment short course"):ti,ab,kw 9695
#2 (((digital* or electronic* or mobile or wireless* or virtual*) NEAR/2 (adherence or medication NEXT monitor* or medication NEXT package* or observ*)) or digital NEXT technolog* or technology-based or "Digital health" or eHealth or "e health" or mhealth or "m health" or SMS or reminder* or short NEXT messag* NEXT service* or text NEXT messag* or MMS or multimedia NEXT messag* or MEMS or (monitor* NEAR/2 (electronic* or sensor* or device*)) or Webcam* or web NEXT cam* or smartphone* or smart NEXT phone* or "web based" or "health IT" or "health ICT" or ((cell* or mobile) NEXT (device* or health or phone* or technolog*)) or video* or cellphone* or feature NEXT phone* or vdot or vmalt or tele* or dat or wot or 99dots or 99 NEXT dots or monitoring NEXT system* or ingestible NEXT sensor* or merm or artificial NEXT intelligence or ai or vot or ((digital or smart) NEXT (pill box* or pillbox*)) or VST):ti,ab,kw 393921
#3 #1 AND #2 with Publication Year from 2000 to 2024, in Trials 2308
#4 (adher* or adverse or complet* or complian* or conversion* or cure* or death* or default or dose* or drug resistan* or fail* or fatal* or mortality or morbidity or outcome* or relaps* or recur* or (risk NEAR/5 disease) or scor* or severity or side effect* or status or surviv*):ti,ab,kw 1521179
#5 (loss to follow up or loss to followup or lost to follow up or lost to followup):ti,ab,kw 31880
#6 (health state* or hrql or hrqol or patient experience* or patient reported or preference* or pro or pros or prom or proms or qol or quality of life or respiratory questionnaire* or satisf* or SGRQ or stigma* or visual analog* scale*):ti,ab,kw 434412
#7 (((disability or quality) NEXT adjusted life) or qald* or qale* or qtime* or life year* or daly* or sf 36 or sf36 or short form 36 or shortform 36 or short form36 or shortform36 or sf thirtysix or sfthirtysix or sfthirty six or sf thirty six or shortform thirtysix or shortform thirty six or short form thirtysix or short form thirty six or sf6 or sf 6 or short form 6 or shortform 6 or sf six or sfsix or shortform six or short form six or shortform6 or short form6 or sf8 or sf 8 or sf eight or sfeight or shortform 8 or shortform 8 or shortform8 or short form8 or shortform eight or short form eight or sf12 or sf 12 or short form 12 or shortform 12 or short form12 or shortform12 or sf twelve or sftwelve or shortform twelve or short form twelve or sf16 or sf 16 or short form 16 or shortform 16 or short form16 or shortform16 or sf sixteen or sfsixteen or shortform sixteen or short form sixteen or sf20 or sf 20 or short form 20 or shortform 20 or short form20 or shortform20 or sf twenty or sftwenty or shortform twenty or short form twenty or hql or

hqol or h qol or hr qol or hye or hyes or (health* NEAR/2 year* NEAR/2 equivalent*) or pqol or qls or quality of wellbeing or quality of well being or index of wellbeing or index of well being or qwb or nottingham health profile* or sickness impact profile* or (health NEAR/3 utilit*) or (utilit* NEAR/3 (valu* or measur* or health or life or estimat* or elicit* or disease or weight)) or (preference* NEAR/3 (valu* or measur* or health or life or estimat* or elicit* or disease or instrument or instruments)) or disutilit* or rosser or willingness to pay or standard gamble* or (time NEXT (trade off* or tradeoff*)) or tto or hui or hui1 or hui2 or hui3 or eq or euroqol or euro qol or eq5d or eq 5d or euroqual or euro qual or duke health profile or (health NEAR/3 assess*)):ti,ab,kw 170867
#8 #4 OR #5 OR #6 OR #7 1569957
#9 #3 AND #8 2159

WOS.SCI,WOS.ISTP,WOS.ESCI (Web of Science Core Collection)

Search:
TS=(((digital* OR electronic* OR mobile OR wireless* OR virtual*) NEAR/2 (adherence OR "medication monitor*" OR "medication package*" OR observ*)) OR "digital technolog*" OR technology-based OR "Digital health" OR eHealth OR "e health" OR mhealth OR "m health" OR SMS OR reminder* OR "short messag* service*" OR "text messag*" OR MMS OR "multimedia messag*" OR MEMS OR (monitor* NEAR/2 (electronic* OR sensor* OR device*)) OR Webcam* OR "web cam*" OR smartphone* OR "smart phone*" OR "web based" OR "health IT" OR "health ICT" OR ((cell* OR mobile) NEAR/1 (device* OR health OR phone* OR technolog*)) OR video* OR cellphone* OR "feature phone*" OR vdot OR vmalt OR tele* OR dat OR wot OR 99dots OR "99 dots" OR "monitoring system*" OR "ingestible sensor*" OR merm OR "artificial intelligence" OR ai OR vot OR ((digital OR smart) NEAR/0 ("pill box*" OR pillbox*)) OR VST)
Editions: WOS.SCI,WOS.ISTP,WOS.ESCI
Date Run: Mon Mar 04 2024 13:36:22 GMT-0500 (Eastern Standard Time)
Results: 1761900

Search:
(
TS=(adher* OR adverse OR complet* OR complian* OR conversion* OR cure* OR death* OR default OR dose* OR "drug resistan*" OR fail* OR fatal* OR mortality OR morbidity OR outcome* OR relaps* OR recur* OR (risk N5 disease) OR scor* OR severity OR "side effect*" OR status OR surviv*)
OR
TS=("loss to follow up" OR "loss to followup" OR "lost to follow up" OR "lost to followup")
OR
TS=("health state*" OR hrql OR hrqol OR "patient experience*" OR "patient reported" OR preference* OR pro$ OR prom$ OR qol OR "quality of life" OR "respiratory questionnaire*" OR isutil* OR SGRQ OR stigma* OR "visual analog* scale*")
OR
TS=(((disability OR quality) N1 "adjusted life") OR qald* OR qale* OR qtime* OR "life year*" OR daly* OR "sf 36" OR sf36 OR "short form 36" OR "shortform 36" OR "short form36" OR shortform36 OR "sf thirtysix" OR sfthirtysix OR "sfthirty six" OR "sf thirty six" OR "shortform thirtysix" OR "shortform thirty six" OR "short form thirtysix" OR "short form thirty six" OR sf6 OR "sf 6" OR "short form 6" OR "shortform 6" OR "sf six" OR sfsix OR "shortform six" OR "short form six" OR shortform6 OR "short form6" OR sf8 OR "sf 8" OR "sf eight" OR sfeight OR "shortform 8" OR "shortform 8" OR shortform8 OR "short form8" OR "shortform eight" OR "short form eight" OR sf12 OR "sf 12" OR "short form 12" OR "shortform 12" OR "short form12" OR shortform12 OR "sf twelve" OR sftwelve OR "shortform twelve" OR "short form twelve" OR sf16 OR "sf 16" OR "short form 16" OR "shortform 16" OR "short form16" OR shortform16 OR "sf sixteen" OR sfsixteen OR "shortform sixteen" OR "short form sixteen" OR sf20 OR "sf 20" OR "short form 20" OR "shortform 20" OR "short form20" OR shortform20 OR "sf twenty" OR sftwenty OR "shortform twenty" OR "short form twenty" OR hql OR hqol OR "h qol" OR "hr qol" OR hye OR hyes OR (health* N2 year* N2 equivalent*) OR pqol OR qls OR "quality of wellbeing" OR "quality of well being" OR "index of wellbeing" OR "index of well being" OR qwb OR "isutility health profile*" OR "sickness impact profile*" OR (health N3 utilit*) OR (isutil* N3 (valu* OR

measur* OR health OR life OR isutili* OR elicit* OR disease OR weight)) OR (preference* N3 (valu* OR measur* OR health OR life OR isutili* OR elicit* OR disease OR instrument OR instruments)) OR isutility* OR rosser OR "willingness to pay" OR "standard gamble*" OR (time W1 ("trade off*" OR tradeoff*)) OR tto OR hui OR hui1 OR hui2 OR hui3 OR eq OR euroqol OR "euro qol" OR eq5d OR "eq 5d" OR euroqual OR "euro qual" OR "duke health profile" OR (health N3 assess*))
)
Editions: WOS.SCI,WOS.ISTP,WOS.ESCI
Date Run: Mon Mar 04 2024 13:36:48 GMT-0500 (Eastern Standard Time)
Results: 16222573

Search:
#3 AND #2 AND #1
Editions: WOS.SCI,WOS.ISTP,WOS.ESCI
Date Run: Mon
Mar 04 2024 13:37:03 GMT-0500 (Eastern Standard Time)
Results: 1801

Database: Web of Science Core Collection

Entitlements:

- WOS.IC: 1993 to 2024
- WOS.CCR: 1985 to 2024
- WOS.SCI: 1900 to 2024
- WOS.AHCI: 1975 to 2024
- WOS.BHCI: 2005 to 2024
- WOS.BSCI: 2005 to 2024
- WOS.ESCI: 2005 to 2024
- WOS.ISTP: 1990 to 2024
- WOS.SSCI: 1900 to 2024
- WOS.ISSHP: 1990 to 2024

Searches:
Search: #3 AND #2 AND #1
Editions: WOS.SCI,WOS.ISTP,WOS.ESCI
Timespan: 2000-01-01 to 2024-03-04
Date Run: Mon Mar 04 2024 13:37:35 GMT-0500 (Eastern Standard Time)
Results: 1737

MedRxiv and other preprints via Europe PMC

(TITLE:Tuberculosis OR TITLE:"Kochs disease" OR TITLE:Phthisis OR TITLE:TB OR TITLE:MTB OR TITLE:MDRTB OR TITLE:XDRTB OR TITLE:DRTB OR TITLE:LTBI OR TITLE:"directly observed treatment short course" OR TITLE:"antituberculosis" OR TITLE:"antituberculous") AND (Adher* OR "directly observed" OR Digital OR electronic OR internet OR mobile OR wireless OR virtual OR TITLE:technology OR "technology based" OR tele* OR ehealth OR "e health" OR mhealth OR "m health" OR SMS OR reminder* OR messaging OR message* OR MEMS OR web OR webcam* OR smartphone* OR "health IT" OR "health ICT" OR video* OR cellphone* OR phone* OR vdot OR vmalt OR dat OR wot OR 99dots OR "99 dots" OR "monitoring system" OR "monitoring systems" OR "ingestible sensor" OR "ingestible sensors" OR merm OR "artificial intelligence" OR AI OR VOT OR "smart pillbox" OR "smart pill box" OR VST OR "computer assisted") AND (SRC:PPR)

Clinicaltrials.gov basic search

Translated and exported to EndNote/RIS and documented

384 records on April 25, 2023

384 Studies found for: **Adherence OR directly observed OR computer OR Digital OR electronic OR internet OR mobile OR virtual OR technology OR video OR mhealth OR artificial intelligence OR AI OR cellphones OR SMS OR reminders OR monitoring OR MEMS OR DAT OR sensors | Tuberculosis OR Kochs disease OR Phthisis OR TB OR MTB OR MDRTB OR XDRTB OR DRTB OR LTBI OR directly observed treatment short course**

The search was updated on March 04, 2024, using "Tuberculosis" as a search term. Studies published between April 25, 2023 and March 04, 2024 were

Translated and exported to EndNote/RIS and documented
74 records on April 25, 2023

Working definition

Table S2 Case definition of digital adherence technology (DAT)

Intervention can be patient-facing only, provider-facing only, or patient and provider-facing.

Intervention includes:

- a digital component (which could be part of a multi-component intervention)
- with the intention to measure or promote treatment adherence and/or reducing missed visits and/or reducing LTFU (and thereby improving successful treatment outcomes)

Examples of "digital adherence component" include (though not limited to):

- SMS daily reminders (1- or 2-way) to patient to take treatment
- SMS reminders (1- or 2-way) to patient to attend dispensing visit/routine follow-up appt
- Automated phone calls to remind patient for visit/daily dose
- Smart pillbox daily reminders to patient to take treatment
- Smart pillbox reminders to patient attend dispensing visit/routine follow-up appt
- Chatbot/telemedicine accessed by TB patients that provides information about "treatment adherence"
- Automated feedback (such as electronic health record) to alert HCW to a missed visit (eg., dispensing, routine follow-up appt for patients on TB/LTBI treatment) by a patient with an intended action of "promote treatment adherence and/or reducing LTFU"
- Digital calendar generated (from electronic health record/smart pill box) showing missed visits/doses by patient at consultation with HCW
- Telehealth – video-call initiated by HCW
- WhatsApp group (with patients/HCWs)- educational messages sent etc..
- Specialist Healthcare provider Apps (– not automated) – used by patient and HCW
- electronic pillbox (or suchlike) used to measure adherence only (ie., not used to promote adherence/improving outcomes) -note, not of interest for outcomes Systematic review

These need to be used in conjunction with the intention to measure* or promote treatment adherence and/or reducing missed visits and/or reducing LTFU (and thereby improving successful treatment outcomes).

Examples to exclude (not limited to)

- electronic health record to document (monitor/record/summarise) visit attendance only - with NO intended action of "promote treatment adherence and/or reducing LTFU"
- non-automated "routine telephone calls" to patient (Note previous SRs such as Liu's Cochrane review, 2014, looked at this. Given our search starts from Jan 2000, we are unlikely to have capture this older-style intervention.
- Phone calls or SMS or WhatsApp (by human) & not automated - with no other digital component ("older-style")
- Papers of hypothetical scenarios of DAT use except if they include any information on cell-phone access# (incl. cell-phone ownership)

Definitions of short-term clinical outcomes

Table S3 Definitions of short-term clinical outcomes

Short-term clinical outcome	Definition
Treatment success	**Treatment success** is a composite outcome including cured and completed.
Cured	**Cured** is a pulmonary TB patient with bacteriologically confirmed TB at the beginning of treatment who completed treatment with evidence of bacteriological response and no evidence of failure.
Treatment completion	**Completed** are all participants who finished treatment and whose outcome does not meet the definition for cure or treatment failure.
Treatment failure	**Treatment failure** is considered when a patient's sputum smear or culture remains positive at 5 months or later after the initiation of treatment or when treatment regimen needs to be terminated or permanently changed to a new regimen or treatment strategy.[1]
Loss to follow up	**Loss to follow up** participants are all who did not start treatment or whose treatment was interrupted for 2 consecutive months or more.
Death	**Death** before starting treatment or during treatment.
Adverse event	An **adverse event i**s any undesirable experience was associated with the use of a medical product in a patient.
Emergence of anti-tuberculous drug resistance	**Emergence of anti-tuberculous drug resistance** renders the bacteria resistant to the most used anti-TB drugs. Among the reasons for this is the non-compliance with the treatment regimens.
Microbiologic conversion of sputum smear or culture	A smear or culture is considered to have converted to negative when two consecutive smears or cultures, taken at least 30 days apart, are negative. In such a case, the specimen collection date of the first negative smear or culture is used as the date of conversion.
Completion of intensive phase	**Completion of treatment of intensive phase** is finishing the first two months of treatment (intensive phase)

Quality assessment tools

Risk of bias assessment tool- randomized controlled trials

Table S4 Cochrane Risk of bias assessment tool for randomized controlled trials[2]

Risk of Bias Assessment Domain	Description	High Risk of Bias	Low Risk of Bias	Unclear Risk of Bias	Reviewer Assessment
Selection bias<br><br>*Random sequence generation*	Described the method used to generate the allocation sequence in sufficient detail to allow an assessment of whether it should produce comparable groups	Selection bias (biased allocation to interventions) due to inadequate generation of a randomized sequence	Random sequence generation method should produce comparable groups	Not described in sufficient detail	High<br>Low<br>Unclear
Selection bias<br><br>*Allocation concealment*	Described the method used to conceal the allocation sequence in sufficient detail to determine whether intervention allocations could have been foreseen before or during enrollment	Selection bias (biased allocation to interventions) due to inadequate concealment of allocations prior to assignment	Intervention allocations likely could not have been foreseen in before or during enrollment	Not described in sufficient detail	High<br>Low<br>Unclear
Performance bias Blinding<br><br>*(Participants and personnel)*	Described all measures used, if any, to blind study participants and personnel from knowledge of which intervention a participant received. Provided any information relating to whether the intended blinding was effective.	Performance bias due to knowledge of the allocated interventions by participants and personnel during the study.	Blinding was likely effective.	Not described in sufficient detail	High<br>Low<br>Unclear
Detection bias Blinding<br><br>*(Outcome assessment)*	Described all measures used, if any, to blind outcome assessors from knowledge of which intervention a participant received. Provided any information relating to whether the intended blinding was effective.	Detection bias due to knowledge of the allocated interventions by outcome assessors.	Blinding was likely effective.	Not described in sufficient detail	High<br>Low<br>Unclear
Attrition bias<br><br>*Incomplete outcome data*	Described the completeness of outcome data for each main outcome, including attrition and exclusions from the analysis. Stated whether attrition and exclusions were reported, the numbers in each intervention group (compared with total randomized participants), reasons for attrition/exclusions where reported.	Attrition bias due to amount, nature or handling of incomplete outcome data.	Handling of incomplete outcome data was complete and unlikely to have produced bias	Insufficient reporting of attrition/exclusions to permit judgment (e.g., number randomized not stated, no reasons for missing data provided)	High<br>Low<br>Unclear

Reporting bias<br><br>*Selective reporting*	Stated how the possibility of selective outcome reporting was examined by the authors and what was found	Reporting bias due to selective outcome reporting	Selective outcome reporting bias not detected	Insufficient information to permit judgment†	High<br>Low<br>Unclear
Other bias<br><br>*Other sources of bias*	Any important concerns about bias not addressed above	Bias due to problems not covered elsewhere in the table	No other bias detected	There may be a risk of bias, but there is either insufficient information to assess whether an important risk of bias exists or insufficient rationale or evidence that an identified problem will introduce bias	High<br>Low<br>Unclear

Newcastle- Ottawa quality assessment scale- cohort studies

Table S5 Cochrane Risk of bias assessment tool for randomized controlled trials

Selection

1) Are patients in the DAT exposed group representative of the average TB patient?
    1= Truly or somewhat representative
    0= selected group of patients or no description of the derivation of the cohort

2) Is the selection of the control group from the same community as the exposed group?
    1= drawn from the same community as the exposed cohort
    0= drawn from a different source or no description of the derivation of the non exposed cohort

3) Is there low risk of bias for the ascertainment of the exposure in the exposed group?
    1= secure record (eg surgical records or structured interview)
    0= written self report or no description

4) Demonstration that outcome of interest was not present at start of study
    1= yes
    0= no

Comparability

1) Exposed and non-exposed groups must be matched in the design and/or confounders must be adjusted for in the analysis. Was the context, management, and processes for care for patients in the exposed group and the comparator group highly comparable except for the exposure?
    2= study controls for age/ sex and for any additional factor
    1= study controls for age/sex only
    0= study does not control for confounders
    N.B. Statements of no differences between groups or that differences were not statistically significant are not sufficient for establishing comparability.

Outcome

1) Blind assessment of outcome
    1= Independent or blind assessment of the outcome, stated in the paper/ or confirmation of the outcome by reference to secure records or Record linkage (e.g. identified through ICD codes on database records)
    0= no blind assessment, no description, or self-report

2) Was follow-up long enough for outcomes to occur
    1= yes (select an adequate follow up period for outcome of interest)
    0= no

3) Was the loss to follow up not related to either the exposure or the outcome?
    1= No attrition bias, complete follow up, all subjects accounted for
    or subjects lost to follow up are unlikely to introduce bias (<10%) and description of those lost.
    0= No description

The tool includes eight items, categorized into three groups: the selection of the study groups, the comparability of the groups, and the ascertainment of the outcome. Each item is given either 0 or 1 – with exception of comparability 0-2 -depending on the methods reported. Note: A study can be awarded a maximum of one star for each numbered item within the Selection and Outcome categories. A maximum of two stars can be given for Comparability.

Table S6 Characteristics of included studies

DAT	Author	Publication year	Type of publication	Time period	Country	World Bank classification of country income level	Study Setting	Study design
**SMS- based intervention**	Ali et al[3]	2019	Full-text paper	2017- 2018	Sudan	Low	8 TB treatment units	Prospective cohort
	Bediang et al[4]	2018	Full-text paper	2013- 2014	Cameroon	Lower-Middle	16 treatment and diagnostic centres in Yaoundé – all centers except the one in prison	RCT
	Belknap et al[5]	2017	Full-text paper	2012- 2014	United States, Spain, Hong Kong, China	Upper-Middle and High	Outpatients TB clinics: 9 sites in the United States, 1 in Spain, and 1 in Hong Kong as part of the PREVENT TB study, and 1 site in South Africa	RCT noninferiority trial
	Dewi et al[6]	2019	Full-text paper	2014	Indonesia	Lower-Middle	Government owned primary health cares, the Sleman District Hospital, and the Community Pulmonary Health Service Unit in Sleman	Quasi-experimental
	Fang et al[7]	2017	Full-text paper	2014- 2015	China	Upper-Middle	6 counties (districts) in Anhui province	RCT
	Farooqi et al[8]	2017	Full-text paper	2014- 2015	Pakistan	Lower-Middle	Khyber Teaching Hospital Peshawar and Emergency Satellite Hospital Nahaqi	RCT
	Gashu et al[9]	2021	Full-text paper	2019	Ethiopia	Low	22 urban and rural health facilities (15 health centres and 7 hospitals)	RCT
	Hermans et al[10]	2017	Full-text paper	2010- 2011	Uganda	Low	The Infectious Diseases Institute in Kampala and its integrated TB-HIV clinic	Quasi-experimental
	Hirsch-Moverman et al[11]	2017	Full-text paper	2013- 2015	Lesotho	Lower-Middle	12 facilities in Berea district: 2 hospitals and 10 health centers	Cluster RCT (The START study)
	Johnston et al[12]	2018	Full-text paper	2012- 2015	Canada	High	2 publicly funded, specialized TB clinics in Vancouver and New Westminster, British Columbia	RCT
	Kibu et al[13]	2022	Full-text paper	Not specified	Cameroon	Lower-Middle	TB treatment centers at the Buea Regional and Kumba District Hospitals, state-owned hospitals at the Southwest Region of Cameroon	RCT (3 arms)
	Kumboyono[14]	2016	Full-text paper	Not specified	Indonesia	Lower-Middle	Dinoyo Community Health Center Malang in EastJava	RCT post-test-only control-group design
	Liu et al[15]	2015	Full-text paper	2011- 2012	China	Upper-Middle	9 clusters, with a rural to urban ratio of 2:1 selected from two cities in each province (Heilongjiang, Jiangsu, Hunan, and Chongqing—located in northern, eastern, central, and western China). Each cluster had > 300 active PTB patients	Cluster RCT^
	Louwagie et al[16]	2022	Full-text paper	2018- 2019	South Africa	Upper-Middle	27 primary care clinics in three districts	Multicenter RCT
	Mohammed et al[17]	2016	Full-text paper	2011- 2014	Pakistan	Lower-Middle	Public and private sector TB clinics in Karachi (a large tertiary center, 9 public facilities, and a network of private General Practitioner clinics and private laboratories)	RCT
	Nguyen et al[18]	2014	Abstract	2012- 2013	Vietnam	Lower-Middle	17 commune health centers of Vung Tau City in Vietnam	Retrospective cohort; HC
	Owiti et al[19]	2012	Abstract	2011	Kenya	Lower-Middle	Moi Teaching and Referral Hospital Chest clinic	Prospective cohort
	Peng et al[20]	2014	Abstract	2011- 2012	China	Upper-Middle	4 counties (districts) in Jiangsu province	Cluster RCT

**Video-observe d therapy**	Bachina et al[21]	2022	Full-text paper	2019- 2021	United States	High	Hennepin County Public Health Clinic's Tuberculosis Program in Minneapolis Minnesota- a setting without prior vDOT use or experience	Prospective cohort
	Burzynski et al[22]	2022	Full-text paper	2017- 2019	United States	High	4 clinics operated by the New York City Health Department	Two-period crossover, noninferiority trial
	Chen et al[23]	2020	Full-text paper	2014- 2017	Taiwan	High	Taipei City	Retrospective cohort
	Chuck et al[24]	2016	Full-text paper	2013- 2014	United States	High	The NYC Department of Health and Mental Hygiene	Prospective cohort
	Doltu et al[25]	2021	Full-text paper	2016- 2017	Moldova	Upper-Middle	15 TB sites for outpatient TB treatment in 5 city districts in Chisinau,	Retrospective cohort α
	Garfein et al[26]	2018	Full-text paper	2014- 2015	United States	High	3 urban (San Diego, San Francisco, Santa Clara) and 2 rural (San Joaquin, Imperial) California health jurisdictions	Prospective cohort; HC
	Guo et al (a)[27]	2020	Full-text paper	2017- 2018	China	Upper-Middle	Center for Chronic Disease Control of the Nanshan district of Shenzhen	Retrospective cohort; HC
	Guo et al (b)[28]	2020	Full-text paper	2018	China	Upper-Middle	Shandong Provincial Chest Hospital Affiliated to Shandong University - Jinan City	RCT
	Lam et al[29]	2018	Full-text paper	2015	United States	High	4 New York City Health Department TB clinics	Prospective cohort; HC
	Lippincott[30]	2022	Full-text paper	2019- 2021	United States	High	the Baltimore City Health Department TB Program in Baltimore, Maryland	Retrospective cohort
	Perry et al[31]	2021	Full-text paper	2018- 2019	United States	High	Large, urban public health program- TB program at the Alameda County Public Health Department in California	Prospective cohort
	Ravenscroft et al[32]	2020	Full-text paper	2016- 2017	Moldova	Upper-Middle	15 clinics in the capital Chişinău	RCT
	Salcedo et al[33]	2021	Full-text paper	2015- 2017	United States	High	1 of the LACDPH clinics at Pacoima, Los Angeles	Prospective cohort historical control
	Salerno et al[34]	2023	Full-text paper	2017-2019	United States	High	4 clinics operated by the New York City Health Department	Two-period crossover trial
	Siddiqui et al[35]	2019	Full-text paper	2014- 2015	United States	High	Harris County Public Health facility in Texas	Retrospective cohort
	Story et al[36]	2019	Full-text paper	2014- 2016	United Kingdom	High	22 clinics in England (London [17 sites], Birmingham [three], Coventry [one], and Leicester [one])	Multicenter RCT superiority trial
	Wade et al[37]	2012	Full-text paper	2003- 2010	Australia	High	The Royal District Nursing Service of South Australia, a community nursing service and the Royal Adelaide Hospital Chest Clinic	Retrospective cohort
	Yu et al[38]	2013	Abstract	2007- 2011	Taiwan	High	Hospital, nursing home group and outpatient clinics	Prospective cohort
**Digital pillbox**	Acosta et al[39]	2021	Full-text paper	2018- 2020	Peru	Upper-Middle	19 Ministry of Health primary healthcare centres with the highest incidence of TB in Callao	RCT
	Broomhead and Mars[40]	2012	Full-text paper	data from a 2005 pilot	South Africa	Upper-Middle	The Betty Gaetsewe Clinic in the Northern Cape Province	Retrospective cohort
	Charalambous et al[41]	2022	Abstract	Not specified	South Africa	Upper-Middle	18 primary health clinics	Cluster RCT
	Guo et al[42]	2023	Full-text paper	2016	China	Upper-Middle	14 villages in Yinjisha County of Kashgar Prefecture in southern Xinjiang, and 2 districts in Urumqi city area in northern Xinjiang	RCT
	Kurada et al[43]	2019	Abstract	2018	India	Lower-Middle	3 TB Units of Hyderabad District in Telangana State	Prospective cohort
	Jerene et al[44]	2024	Preprint	2021-2022	Philippines	Lower middle		Cluster RCT

					South Africa<br>Tanzania<br>Ukraine	Upper middle<br>Lower middle<br>Lower middle	In consultation with the National TB Program (NTP) in each country, districts (Ukraine), regions (Tanzania) and provinces (South Africa and the Philippines) were identified, and facilities purposively selected based on having previously notified TB cases, willingness, and capacity to participate, and a reasonable balance of urban/rural and large/small facilities.	
	Liu et al[15]	2015	Full-text paper	2011- 2012	China	Upper-Middle	9 clusters, with a rural to urban ratio of 2:1 selected from two cities in each province (Heilongjiang, Jiangsu, Hunan, and Chongqing—located in northern, eastern, central, and western China). Each cluster had > 300 active PTB patients	Cluster RCT^
	Liu et al[45]	2023	Full-text paper	2017- 2019	China	Upper-Middle	4 prefectures (administrative subdivisions of provinces) in China. Geographical areas served by a tuberculosis dispensary or designated hospital were the unit of randomisation (clusters)	Cluster RCT (superiority trial)
	Manyazewal et al[46]	2022	Full-text paper	2020- 2021	Ethiopia	Low	10 health care facilities	Multicenter RCT
	Manyazewal et al[47]	2023	Full-text paper	2020- 2021	Ethiopia	Low	10 health care facilities in Ethiopia.	Multicenter RCT
	Manyazewal et al (b)[48]	2022	Full-text paper	2020- 2021	Ethiopia	Low	10 health care facilities	Multicenter RCT
	Moulding & Caymittes[49]	2002	Full-text paper	Not specified	Haiti	Lower-Middle	TB clinic using self administered medication in Port-Au-Prince, Haiti	RCT
	Park et al[51]	2019	Full-text paper	2014- 2015	Morocco	Lower-Middle	5 health centers (Bab Khmiss, Sidi Mousa, Hay Rahma, Laayayda, and Hay Salam II) in the Sale area of the Rabat–Sale–Kenitra region.	Retrospective cohort
	Pima et al[52]	2021	Abstract	Not specified	Tanzania	Lower-Middle	In Kilimanjaro (no further details provided)	RCT
	Ratchakit-Nedsuwan et al[53]	2020	Full-text paper	2014- 2015	Thailand	Upper-Middle	TB clinic at Chiangrai Prachanukroh Hospital, a tertiary- care hospital located in Thailand's northernmost province.	Mixed methods pilot RCT
	Saha et al[54]	2022	Full-text paper	2020- 2021	India	Lower-Middle	5 TB units in Urban Nasik (considered a district unit)<br>The TB units were assigned into two arms, ensuring that they are geographically apart	Quasi-experimental
	Velen et al[55]	2022	Full-text paper	2017- 2020	Vietnam	Lower-Middle	2 clinics in Thanh Hoa Province	RCT
	Wang et al[56]	2020	Full-text paper	2018	China	Upper-Middle	30 counties (districts) from 3 provinces: 9 from Zhejiang province (eastern region), 16 from Jilin province (middle region) and 5 from Ningxia Autonomous Region (west region)	Prospective cohort
	Wang et al[57]	2024	Full-text paper	2022	China	Upper-Middle	Patients were diagnosed at 10 TB-designated hospitals and came from 220 community health service centers or township health centers in Wuhanurban setting	Prospective cohort
	Wei et al[58]	2024	Full-text paper	2018- 2021	China	Upper-Middle	6 settings in countries/ districts in Shigtase, Tibet	Multicentre RCT
	Wu et al[59]	2023	Full-text paper	2019	China	Upper-Middle	Sixteen CHCs in Songjiang District Center for Diseases Control and Prevention (CDC) in Shanghai	Prospective cohort
**Featu re-**	Bassett et al[60]	2016	Full-text paper	2010- 2012	South Africa	Upper-Middle	2 hospital-affiliated outpatient departments and 2 municipal clinics (nurse-driven primary health care sites) in the greater Durban area	Multicenter RCT

	Das Gupta et al[61]	2020	Full-text paper	2015	India	Lower-Middle	3 of 6 tuberculosis units (TU) in Ahmedabad	Quasi-experimental
	Hope et al[62]	2022	Abstract	2020- 2021	Uganda	Low	5 selected health facilities in the Karamoja subregion (a nomadic population in the Karamoja sub region-Northeastern Uganda)	Prospective cohort
	Hope et al[63]	2022	Abstract	2020 -2021	Uganda	Low	5 public health facilities	RCT
	Khachadourian et al[64]	2020	Full-text paper	2014	Armenia	Upper-Middle	Outpatient TB centres (52 out of 60 clusters) stratified by patient load and treatment success	Cluster RCT (noninferiority trial)
	Santra et al[65]	2021	Full-text paper	2018- 2019	India	Lower-Middle	6 DOTS centers under the jurisdiction of a major hospital in Central Delhi	Quasi-experimental
	Sodhi et al[66]	2023	Preprint	2019-2020	India	Lower-Middle	3 private care facilities in New Delhi, India, namely, Vinod Karhana Hospital, Ganga Ram Hospital, and St. Stephens hospital	Quasi-experimental
	Yoeli et al[67]	2019	Research letter	2016- 2017	Kenya	Lower-Middle	17 health clinics that selected by the Kenya Ministry of Health	RCT
**99DOTS**	Cattamanchi et al[68]	2021	Full-text paper	2018- 2019	Uganda	Low	18 health facilities with National TB and Leprosy Program–affiliated TB treatment units:(= 5 regional referral hospitals, 10 general hospitals, and 3 district health centers) *	Stepped-wedge cluster RCT
	Chen et al[69]	2022	Full-text paper	Pre-intervention 2017 and post-intervention group 2018- 2019	India	Lower-Middle	74 TB Units in 12 districts in Himachal Pradesh, in northern India, mostly rural with several urban centers	Retrospective cohort (pre- post study)
	Crowder et al[70]	2024	Full-text paper	2019- 2021	Uganda	Low	18 99DOTS experienced facilities and 12 99DOTS naïve facilities	Retrospective cohort (pre- post study)
	Jerene et al[44]	2024	Preprint	2021-2022	Philippines<br>South Africa<br>Tanzania	Lower middle<br>Upper middle<br>Lower middle	In consultation with the National TB Program (NTP) in each country, regions (Tanzania) and provinces (South Africa and the Philippines) were identified, and facilities purposively selected based on having previously notified TB cases, willingness, and capacity to participate, and a reasonable balance of urban/rural and large/small facilities.	Cluster RCT
	Thekkur et al[71]	2019	Full-text paper	2016	India	Lower-Middle	21 ART centres situated in four districts of Karnataka State (Belgaum, Gulbarga, Bagalkot and Bangalore city)	Mixed-methods study with quantitative cohort study
	Wambi et al[72]	2022	Abstract	2020- 2021	Uganda	Low	30 health facilities	not specified- assumed retrospective cohort study
**Inges tible**	Browne et al[73]	2019	Full-text paper	2013- 2016	United States	High	2 sites in San Diego Country and Orange County Divisions of TB Control and Refugee Health (Public health treatment programs in southern California)	Stage 1: Prospective cohort Stage 2: RCT
**Smartphone apps**	Haslinda & Juni[74]	2019	Full-text paper	2017- 2018	Malaysia	Upper-Middle	Health Clinics in the Seremban District; district has the highest number of reported TB cases and low treatment success	RCT
	Iribarren et al[75]	2022	Full-text paper	2019- 2020	Argentina	Upper-Middle	Hospital Cetrangolo: specialized in respiratory medicine in the Province of Buenos Aires	RCT (mixed methods)
	Wang et al[57]	2024	Full-text paper	2022	China	Upper-Middle	Patients were diagnosed at 10 TB-designated hospitals and came from 220 community health service centers or township health centers in Wuhanurban setting	Prospective cohort

	Wu et al[59]	2023	Full-text paper	2019	China	Upper-Middle	Sixteen CHCs in Songjiang District Center for Diseases Control and Prevention (CDC) in Shanghai	Prospective cohort
	Zhadnova et al[76]	2020	Full-text paper	Not specified	Russian Federation	Upper-Middle	The Irkutsk Regional Clinical Tuberculosis Hospital	Prospective cohort
	Zhang[77]	2023	Full-text paper	e-PSS group 2021 TCIS group 2020	China	Upper-Middle	17 (all) districts in Wuhan with a TB program.	comparative cross-sectional study using retrospective data.
	Zhou et al[78]	2018	Abstract	2013- 2015	China	Upper-Middle	Wuhan Pulmonary Hospital	RCT
**SMS+ Digital pillbox**	Liu et al[15]	2015	Full-text paper	2011- 2012	China	Upper-Middle	9 clusters, with a rural to urban ratio of 2:1 selected from two cities in each province (Heilongjiang, Jiangsu, Hunan, and Chongqing—located in northern, eastern, central, and western China). Each cluster had > 300 active PTB patients	Cluster RCT^
	Musiimenta et al[50]	2023	Full-text paper	2019-2020	Uganda	Low	The TB clinic at Mbarara Regional Referral Hospital	Mixed methods pilot RCT

RCT randomized controlled trial; HC historical control

* Eligible health facilities treat >10 TB patients/month in 2017, are located within 225km of Kampala but not within Kampala District, and had a TB treatment success rate < 80% in 2017

α combined from two data sources: the RCT conducted in 2016-2017 and the national TB register (SIME-TB)

Table S7 Characteristics of study participants and intervention in included studies

	Study ID	DAT Participants				DAT Intervention				Comparator		
		TB type	N	Age Mean (SD)	HIV (%)	Type	Duration	Frequency	Details	Type	Frequency	N
SMS- based intervention	Ali et al	PTB §	74	37.8 (17.9)	0.0	One-way SMS	6 months	Weekly	MR (sent every 2 days in the first 2 months then weekly)	DOT-clinic	N/A	74
	Bediang et al	SS+ PTB # §§ (newly diagnosed)	137	Median in age category 26-40	19.7	One-way SMS	6 months	Daily	MR; MM (message content changes every 2 weeks)	SAT (a)	N/A	142
	Belknap et al	LTBI	328	Median 38 IQR (27-49)	1.5	One-way SMS	16 weeks	Weekly	MR; medication dispensed in a digital pillbox (used only to record adherence monthly)	SAT (h)	N/A	337
	Dewi et al	Active TB (newly diagnosed) §	60	Median in age category 18-55	N/A	One-way SMS	56 days-intensive phase	Daily	MR; MM; EM	DOT- no details	N/A	60
	Fang et al	PTB # §	160	47.6 (13.4)	N/A	One-way SMS	6 months	Daily	MR; XR; EM	DOT- no details	N/A	190
	Farooqi et al	PTB§ (newly diagnosed)	74	29.4 (17.6)	N/A	One-way SMS+ graphics-based messages	2 months-intensive phase	Daily	MR	not clear	N/A	74
	Gashu et al	DS TB §§ (newly diagnosed)	152	Median in age category 18-29	12.5	One-way SMS+ graphics-based messages	4 months-continuation phase	Daily	MR; weekly RR	DOT-home (b)	N/A	154
	Hermans et al	HIV+ with active TB §	183	35.0 (9.4)	100.0	Two-way SMS	2 months	2-3x/ week	After the most recent appointment adherence reminder on days 2, 7, and 11; interactive educational quizzes on days 3, 6, 9, and 12; AR every 2 weeks; a toll-free call back for support with any queries	SAT (c)	N/A	302
	Hirsch-Moverman et al	HIV+ with active TB	183	37.6 (10.4)	100.0	One-way SMS automated and coded	6 months	Daily or weekly	MR (sent daily in the first 6 weeks); AR at prescheduled time sent to the patients and/ or treatment supporters	SAT or DOT-family	N/A	166
	Johnston et al	LTBI	170	Median 45 IQR (34-55)	N/A	Two-way SMS	9INH regimen: < 12 months, 4RIF < 6 months	Weekly	participants respond to the weekly "check in" SMS to identify any concern; a second SMS within 48h in case of a missed reply; a phone call after 48h in case of a missed reply	SAT	N/A	188
	Kibu et al	Active TB- no details	28	37.9 (13.2)	45.2	Two-way SMS and 1-way SMS	3 months	3x/ week	1 way SMS: MR<br>2-way SMS: MR; patients confirm drug intake via SMS	not clear	N/A	28
	Kumboyono	Active TB- no details	45	Median in age category 18-39	N/A	One-way SMS	Throughout the treatment (assuming)	Daily	MR	DOT-field	N/A	45

	Liu et al	Active TB # §	1008	Median 43 IQR (29-56)	N/A	Two-way SMS	6 months	Every 2 days	MR at prescheduled time (SMS); AR 4, 3, 2, and 1 day before the scheduled monthly follow-up visit; patient confirms drug intake via SMS; 2 additional MR (SMS) in case of a missed reply	Choice from SATor DOT-family or field (h)	N/A	1104
	Louwagie et al	DS PTB § literate, Tobacco smokers	283	38.6 (11.2)	50.2	One-way SMS	12 weeks	2x/week (SMS)	3 motivational counselling sessions; SMS: 10 TB-related and 7 smoking or alcohol reduction related	Not clear (d)	N/A	291
	Mohammed et al	Active TB § (newly diagnosed)	1110	33.0 (16.0)	N/A	Two-way SMS automated & coded	6 months	Daily	MR at prescheduled time; patient confirms drug intake via SMS or a missed call; 2 additional MR (SMS) in case of a missed reply; a phone call in case of a missed response for 7 days	DOT- no details	N/A	1097
	Nguyen et al	PTB (newly diagnosed)	136	N/A	N/A	One-way SMS	6-7 months	N/A	MR; AR; real-time access to patient adherence data	Not clear	N/A	270
	Owiti et al	Active TB- no details	150	30.9	31.1	One-way SMS			AR	NR	N/A	37
	Peng et al	Active TB- no details	234	Median in age category 15-59	N/A	One-way SMS	6 months	Daily	MR	DOT- no details	N/A	229
**Video-observed therapy**	Bachina et al	DS PTB and EPTB	23	35.0 (17.0)	N/A	Asynchronous VOT	>= 2 months	daily	MR (SMS) 2x/day; chat allowed between patient and health care team in case of questions; videos reviewed the next business day	DOT-home or field	5x/ week	26
	Burzynski et al	DSTB	113	Median 40 range (16-86)	4.0	Choice between Synchronous or asynchronous VOT	4 weeks	5x/week	patients loaned a smartphone if they did not own one; monthly reimbursement of data usage costs; reimbursement for completing enrollment visit and questionnaire	DOT-clinic or field	N/A	103
	Chen et al	LTBI	80	Median in age category 15-29	N/A	Synchronous VOT	9 months	N/A	nutritional allowance according to adherence to treatment for both groups	DOT-field	N/A	160
	Chuck et al	DS or DR, PTB or EPTB	61	Median 36 range (18-85)	10.0	Synchronous VOT pre-arranged schedule	5.3months	N/A	reporting of side effects; a physician is contacted in case of reported side effects; a phone call in case of a missed video, if unsuccessful, a home visit	DOT-variable €	N/A	329
	Doltu et al	Active TB- no details	83	35.5 (13.9)	5.3	Asynchronous VOT	3 months	Daily	patients loaned a tablet if no personal resources available, reporting of side effects; a video containing MM sent back to the patients after they send the video; a phone call in case of a missed video	DOT-clinic	5x/ week	478
	Garfein et al	DS PTB	272	Median 44 range (18-87)	N/A	Asynchronous VOT	5.4 months	Daily	MR (SMS or email) daily; all patients loaned smartphones with cellular data	DOT-field (f)	N/A	159

									plans; advice to call or visit healthcare provider before taking medications if side effects occur; patients contacted in case of missed videos			
	Guo et al (a)	DS PTB	235	Median in age category 25-44	N/A	Asynchronous VOT	6 months	Daily	MR (app); reporting of side effects, biweekly visits to TB specialist for the first 2 months then once monthly; phone call in case of a missed video	DOT-clinic	daily	158
	Guo et al (b)	DS bacteriologically confirmed PTB	203	40.2 (16.1)	N/A	Synchronous VOT	1-12 months	N/A	MR (SMS); reporting of any problem with the treatment; phone call and/or home visits in case of a missed video	DOT- no details	every 2 days	202
	Lam et al	LTBI	50	Median 33.5 IQR (25-46)	0.3	Synchronous VOT	16 weeks	Daily	MR (SMS) after patients' approval; phone call in case patients were 5min late to log in; an SMS in case of a missed video	DOT-clinic	N/A	302
	Lippincott	PTB and EPTB	30	Median 43 IQR 30-57	2	Asynchronous VOT (2 way)	N/A	Daily	MR (app); videos sent to a web-based dashboard used by the TB clinic	DOT-home or field	5x/ week	22
	Perry et al	DS or DR, PTB or EPTB	94	46.1 (17.7)	4.4	Asynchronous VOT	27 weeks	5x/ week §	patients encouraged to report side effects; 2x/daily reminders (SMS) in case of a missed video	DOT (g)	5x/ week	69
	Ravenscroft et al	DS or DR, PTB or EPTB but not MDR-TB patients	85	38.7 (14)	N/A	Asynchronous VOT (2way)	4 months	Daily	patients loaned a tablet if no personal resources available, reporting of side effects; a video containing MM sent back to the patients after they send the video; a phone call in case of a missed video	DOT-clinic	5x/ week	93
	Salcedo et al	DS PTB	43	48.4 (19.8)	0.0	AiCure, an AI platform that uses computer vision and machine learning accessed by patients using a smartphone app	4 months (continuation phase)	Daily or 2x/week	AiCure automatically detects medication ingestion and flags complications or doses not completed correctly for nurse review, allowing nurses to focus on cases that need more attention.<br>1-2 clinic appointment/month	DOT-clinic or field	N/A	71
	Salerno et al	DS TB	113	Median 40 range (16-86)	4.0	Choice between Synchronous or asynchronous VOT	4 weeks	5x/week	patients loaned a smartphone if they did not own one; monthly reimbursement of data usage costs; reimbursement for completing enrollment visit and questionnaire	DOT-clinic or field	N/A	103
	Siddiqui et al	Active TB and LTBI	47	Median in age category 15-39	N/A	Asynchronous VOT	N/A	Active TB 3X/ week LTBI 2X/ week	in-person DOT in intensive phase; recorded videos are submitted through a mobile device with a mobile application	DOT-field (m)	3x/ week TBD 2x/ week LTBI	47
	Story et al	DS, PTB or EPTB	112	Median in age category	N/A	Asynchronous VOT	2-6 months	Daily	MR (SMS) daily according to patients' choice; reporting of side effects;	DOT-field	3-5x/ week	114

				16-34					videos regularly acknowledged with a personalised MM or email; a phone call in case of a missed video			
	Wade et al	Not specified	58	Median in age category 30-39	N/A	Synchronous VOT	5.5 months	Daily	a minority 3x/week or 2x/ daily; nurses attempt to call patients up to 3 times, if unsuccessful a message is sent to reschedule the video call	DOT-field	N/A	70
	Yu et al	MDR TB	39	37.8	N/A	Synchronous VOT	24 months-assumed	N/A	VOT at prescheduled time	DOT-field	N/A	99
**Digital pillbox**	Acosta et al	DS PTB	53	Median in age category 18-35	N/A	Real-time monitoring	4 months	3x/ week	Up to 3 SMS/ day in case of a missed dose	DOT-clinic	N/A	53
	Broomhead and Mars	DS PTB	24	37.9 (16.2)	N/A	Real-time monitoring	6 months	N/A	SMS in case of a missed dose	DOT- no details	N/A	96
	Charalambous et al	DS TB	1306	Median 36	52	Real-time monitoring	N/A	N/A	MR (MERM- audio and visual); weekly adherence reports and patients' intensified support (text, phone call, home visit, motivational counselling) depending on number of missed doses	Not clear (h)	N/A	1278
	Guo et al	PTB	43	Village 36.7 (17.7),	0.0	Real-time monitoring	~ 6.5 months	Daily	MR at prescheduled time	DOT- no details	N/A	38
			50	Downtown 33.9 (18.1)	0.0							36
	Kurada et al	DS PTB	385	N/A	N/A	Real-time monitoring	7 months	Daily	MR (daily); RR	DOT- no details	N/A	3019
	Jerene et al - Philippines	DS PTB	2236	Median 47 IQR 33-58	N/A	Real-time monitoring	6 months	Daily	MR (automated SMS), SMS, phone call and home visits in case of 1,2 and 3 missed doses.Missed doses marked in Everwell Hub app. Interventions were combined with differentiated care pathway, based on patient engagement with a DAT as a proxy for treatment adherence.	SAT or field DOT	daily	4188
	Jerene et al - South Africa	DS TB	1281	Median 41 IQR 33-50	0.06	Real-time monitoring	6 months	Daily		SAT (p)	daily	1880
	Jerene et al - Tanzania	DS TB	1778	Median 45 IQR 33-60	0.08	Real-time monitoring	6 months	Daily		SAT or field DOT	daily	3656
	Jerene et al - Ukraine	DS TB	1526	Median 44 IQR 36-54	0.01	Real-time monitoring	4 months	Daily		Clinic or home DOT	daily	1589
	Liu et al (2015)	Active TB #	997	Median 43 IQR (29-56)	N/A	No real-time monitoring (assumed)	6 months	Every 2 days	MR at prescheduled time (SMS); AR 4, 3, 2, and 1 day before the scheduled monthly follow-up visit; patient confirms drug intake via SMS; 2 additional MR (SMS) in case of a missed reply	Choice from SATor DOT-family or field (h)	N/A	1104
	Liu et al (2023)	DS PTB	1298	Median 42 IQR 29-57	0.0	No real-time monitoring	6 months with follow-up visits at 12 and 18 months	Daily	MR 3x/day at prescheduled time (MERM-audio and visual) 3x/day; monthly AR (MERM); monthly review of adherence data and differentiated	Choice from SATor DOT-	daily	1388

									care for patients with adherence issues.	family or field		
	Manyazewal et al (2022)	DS PTB	57	32.9 (11.1)	17.5	No real-time monitoring	2 months (intensive phase)	Daily	MR; RR (audio and visual); pill count is compared with data sent from MERM every 15 days at the clinic; intensified support (text, phone call, home visit, motivational counselling) depending on number of missed doses	DOT-clinic (n)	daily	57
	Manyazewal et al (2023)	DS PTB	57	33.2 (11.1)	15.4							
	Manyazewal et al (b)	DS PTB	57	33.2 (11.1)	15.4							
	Moulding & Caymittes	DS PTB	64	N/A	N/A	No real-time monitoring	1 year	N/A	Group A MERM + counseling, Group B & C no feedback with and without MERM	SAT	N/A	59
	Park et al	DS PTB	206	36.7 (14.9)	N/A	Real-time monitoring	6 months	6x/ week	MR; a phone call or a home visit in case of a missed dose	DOT & SAT (i)	N/A	141
	Pima et al	Active TB- no details	297	N/A	N/A	Real-time monitoring	6 months	Not clear	MR (SMS)	Not clear	N/A	228
	Ratchakit-Nedsuwan et al	DS PTB or EPTB	50	50.0 (16.0)	0.0	Real-time monitoring	6 months	Daily	MR (pillbox-audio) at prescheduled time; a phone call in case of a missed dose; access (through the pillbox) to health staff in case of immediate need to consultation	SAT or DOT-family	N/A	50
	Saha et al	DS TB	200	37 range (18-92)	N/A	Real-time monitoring	7 months, (up to 8 months in poor adherence)	Daily	MR (Tuberculosis Monitoring Encouragement Adherence Drive (TMEAD)); daily updates and patient analytics to peripheral health institutions through an application; weekly calls to document experience with treatment	Not clear "SoC according NTEP guidelines"	N/A	200
	Velen et al	DS PTB	124	Median 52 IQR 35-61	66.1% unknown	Real-time monitoring	4-7 months	daily and monthly	daily MR (MERM) audible alert; AF to the healthcare worker and to patients for the previous month; reasons for the missed doses discussed; treatment regimens were reviewed and changed in accordance with routine care in case non-adherence was linked to adverse events; medication dispensed in MERM for both groups	Not clear (h)	daily	126
	Wang et al	DS PTB	1047	Median in age category 45-60	N/A	No real-time monitoring	6-12 months	Daily	MR; AF based on their treatment card in monthly follow up visits.	SAT (j)	N/A	763
	Wang et al	PTB	124	Median in age category 40-59	N/A	Real time-monitoring	Not clear	Daily	MR (audio and visio), phone call after several missed doses	SAT(o)	daily	2136
	Wei et al	DS PTB	143	Median 57 IQR 40-65	N/A	real time monitoring	6-7 months	Daily	Audio MR, real-time AF, CTS through WeChat app, data plan provided to all and smartphones to few patients, patients select their treatment	SAT (h)	daily	135

									supporter, real-time audio/video-based DOT by the village doctor for 3 days in case of suboptimal adherence			
	Wu et al	DS PTB	90	Median 44 IQR 31-65	0.00	Real-time monitoring	6 months	Daily	MR (audio and visio), AF and CTS through management app	SAT or DOT-family	daily	88
Feature phone-based	Bassett et al	Active TB- no details	293	35.0 (10.0)	100.0	SMS and phone calls	6 months	5x/ 16 weeks	5 scheduled phone calls in weeks 1, 4, 8, 12, and 16 after enrollment);4 SMS reminders to retrieve test results and AR; phone call, to reassess perceived barriers; CTS for questions.	Not clear	N/A	230
	Das Gupta et al	TB (newly diagnosed)	111	Group A: 36.1, Group B: 36.7	N/A	SMS, live calls, pre-recorded calls (group A) or SMS & phone calls (group B)	4 months	N/A	Group A: choice from (i) SMS, (ii) live calls, (iii) pre-recorded calls, and (iv) a combination of SMS and live and pre-recorded calls.Group B: Live call (reminder cues)Treatment-completion rewards	DOT-clinic (k)	N/A	111
	Hope et al	Active TB- no details	210	N/A	N/A	interactive voice response software- The CallforLife tool	N/A	N/A	CallForLife-TB is an interactive voice response software providing daily MR (phone call), health tips, AR, and allows remote symptom reporting and records patients' adherence to TB medicines and subsequent treatment outcomes	Not clear	N/A	248
	Hope et al	Active TB- no details	129	Median 34.4 IQR 26.6-45.3	34.6	interactive voice response software- The CallforLife tool	6 months	Daily	CallForLife-TB is an interactive voice response software providing daily MR (phone call), health tips, AR, and allows remote symptom reporting and records patients' adherence to TB medicines and subsequent treatment outcomes	Not clear	N/A	131
	Khachadourian et al	DS PTB	227	45.2 (15.7)	8.4	SMS and phone calls	4-5 months	Daily	an educational/counseling session; weekly visits to TB centres for drug refill; daily MR (SMS); phone calls to track adherence and record side effects	DOT-clinic	N/A	209
	Santra et al	Active TB #	110	44.8 (10.8)	N/A	SMS and phone calls	90 days	SMS: daily, Phone calls: weekly	MM; MR; phone calls addressing concerns regarding potential adverse effects	DOT- no details	N/A	110
	Sodhi et al	DS TB	276	Median 38 IQR 24-56	N/A	Connect for Life; Interactive Voice Response System and SMS	Not clear	Daily	MR, AR and EM, treatment coordinators utilize increase follow ups when the patients were found to lag in medication adherence.	Not clear	N/A	713

	Yoeli et al	DS TB	569	30.6	32.8	Keheala: Mobile phone platform and SMS	2-12 months	Daily	daily MR (SMS); daily verification of adherence using the USSD interface; weekly MM (SMS); CP- "Adherence contest" in which patients can compare their reported adherence with others to win; information about TB through the platform; interaction with study team members for support and advice; a wristband inscribed with a MM	Not clear	N/A	535
99DOTS	Cattamanchi et al	DS PTB	891	39.7 (14.6)	46.2	99DOTS	6 months	Daily	daily automated MR (SMS); patients confirm drug intake by making daily toll-free phone calls to the number printed on the envelope of the blister pack; EM and MM sent to patients after they confirm drug consumption	DOT-field or family	N/A	1022
	Chen et al	DS PTB and EPTB	8322	42.4 (18.3)	0.8	99DOTS	24 weeks	Daily	No MR; direct benefit transfers to patients (₹500/ monthly for nutritional support); providers could see an auto-updated list of patients with low adherence as reported by 99DOTS	DOT	daily	7722
	Crowder et al	DS PTB	2051	Median 35 IQR 28-43	43.7	99DOTS	N/A	Daily	Provision of low-cost phones to patients who lack access, task-shifting of adherence monitoring and patient follow-up to community health worker; automated task lists to facilitate follow-up	DOT-field	N/A	1475
	Jerene et al - Philippines	DS PTB	2368	Median 47 IQR 33-58	N/A	Real-time monitoring	6 months	Daily	MR (automated SMS), SMS, phone call and home visits in case of 1,2 and 3 missed doses.Missed doses marked in Everwell Hub app. Interventions were combined with differentiated care pathway, based on patient engagement with a DAT as a proxy for treatment adherence.	SAT or field DOT	daily	4188
	Jerene et al - South Africa	DS TB	1213	Median 41 IQR 33-50	0.06	Real-time monitoring	6 months	Daily		SAT (p)	daily	1880
	Jerene et al - Tanzania	DS TB	1768	Median 45 IQR 33-60	0.08	Real-time monitoring	6 months	Daily		SAT or field DOT	daily	3656
	Thekkur et al	HIV+ with DS TB	870	Median in age category 30-44	100.0	99DOTS	6 months	Daily	patients confirm drug intake by making daily toll-free phone calls to the number printed on the envelope of the blister pack; an alert message to the supervisor in case of a missed dose to visit the patient; drug dispensed for 28 days during routine visits	DOT-field	3x/ week	961
	Wambi et al	DS PTB	309	Median 17 IQR 15-18	20.1	99DOTS	N/A	N/A	** Adolescents (15-19 years) with confirmed TB and a treatment	DOT	N/A	132

									supporter or were treated at lower-level health centers were more likely to enroll on 99DOTS			
Ingestible [illegible]	Browne et al	SS- TB	41	41.0 (16.0)	N/A	Ingestiblesensors * (WOT)	< 12 months	5x/week	Stage 1 = 2-3 weeks to assess accuracy of WOT, Stage 2 = WOT: to the end of treatment complete < 12 months	DOT-clinic or field	5x/ week	20
Smartphone apps	Haslinda & Juni	DS PTB (newly diagnosed)	55	38.3 (13.7)	0.8	TB@Clicks educational module delivered through Whatsapp.	6 months	Daily	EM sent once during intensive phase and 3 times during maintenance phase; CTS- ask questions related to TB through WhatsApp; both groups received DOT (clinic) and MR in the intensive phase	DOT-clinic	N/A	55
	Iribarren et al	DS PTB	21	41.4 (17.6)	N/A	Mobile application	6 months	Daily; 3x/week adherence check with urine strip	Daily reporting of drug intake, reporting of side-effects, uploading a photo of the urine test to verify adherence; access to information about TB, a personal calendar view of the treatment progress; CTS or CP anonymously in a group discussion forum	SAT (l)	daily single-pill	21
	Wang et al	PTB	124	Median in age category 40-59	N/A	WeChat application and subscription to ‘‘E taking medication” account	Not clear	Daily	MR through the app and confirmation of drug intake through the account	SAT(o)	Daily	2136
	Wu et al	DS PTB	82	Median 26 IQR 24-33	0.00	A reminder app	6 months	Daily	MR, AF and CTS through the app	SAT or DOT-family	Daily	88
	Zhadnova et al	HIV+ with PTB with a history of psychoactive substances abuse	44	37.8 (5.8)	100.0	A specially developed smartphone app	6 months	Messages: daily, quizzes: weekly	daily self-monitoring questions (mood, stress and treatment adherence); AR; anonymous chat (CTS medical consultations, CP); weekly quizzes.	Those who refused to use the app	N/A	10
	Zhang	DS PTB	1145	47.9 (19.3)	N/A	Mobile phone application, or WeChat (an instant messaging service)	6 months	daily	a website, mobile phone application, or WeChat (an instant messaging service). The e-PSS patient treatment management module: manage and follow up patients via mobile phone, (2) MR and AR sent to community doctors and patients, (3) AF in real time	Not clear	N/A	1576
	Zhou et al	MDR TB	112	N/A	N/A	Messaging smartphone app	24 months	N/A	WeChat, QQ, and telephone communication after hospital	Not clear	N/A	112

									discharge and health education from dedicated nursing staff and standard treatment			
SMS+ Digital pillbox	Liu et al (2015)	Active TB #	997	Median 43 IQR (29-56)	N/A	No real-time monitoring (assumed)	6 months	Every 2 days	MR at prescheduled time (SMS); AR 4, 3, 2, and 1 day before the scheduled monthly follow-up visit; patient confirms drug intake via SMS; 2 additional MR (SMS) in case of a missed reply	Choice from SATor DOT-family or field (h)	N/A	1104
	Musiimenta et al	DS TB	22	Median 37.5 IQR 32-54	86%	Real-time monitoring	6 months	Daily SMS	MR (SMS) daily for the first 3 months, for the next 3 months MR (SMS) if the pillbox was not opened	SAT with pillbox	daily	21
			23	Median 31 IQR 25.5-41	85%	Real-time monitoring	6 months	Weekly SMS	MR (SMS) weekly for the first 3 months, for the next 3 months MR (SMS) if the pillbox was not opened			

PTB pulmonary TB, EPTB extra-pulmonary TB, DS drug susceptible, DR drug resistant, MDR multidrug resistant, LTBI latent tuberculosis infection,
SAT self-administered therapy, DOT directly observed treatment, MR medication reminder, AR appointment reminder, MM motivational message, EM educational message, XR examination reminder, RR refill reminder, AF adherence feedback; CTS communicate with treatment supporter; CP communicate with other patients; WOT wireless observed therapy.
§§ participants enrolled own a mobile phone,
§ participants enrolled have access to a mobile phone,
# patient or family member can read
* a sensor made of minerals that is swallowed with TB medication and subsequently records the ingestion on a cellphone
In South Africa and Tanzania the DAT intervention started 1 week after treatment initiation, while in Ukraine it started after 2 months of treatment initiation.[44]
(a) Routine care was referred to as selective DOT. Selective DOT included free treatment, appointments for drug refill, 3 smear control tests at 2, 5, 6 months, education coupled with counselling throughout the process.
(b) Routine care in continuation phase TB treatment means patients take their daily medication at home with the help of community health worker), family member, neighbour, workmate.
(c) DOT was not practiced, although “community-based DOT with treatment buddies” was advocated.
(d) The routine care includes health education, dietetic input, social support, point of care biochemical testing, and HIV testing with pretest and post-test HIV test counselling.
(e) “Patients on in person DOT were defined as those who had at least one dose of medication observed at a health department or hospital clinic or in the community and underwent no VDOT observation.”
(f) San Francisco also offered clinic-based DOT. Nonclinical personnel conducted most DOT visits; nurses also provided some DOT based on clinical needs and staffing considerations.
(g) Patients with no VOT include some patients who received exclusively SAT.
(h) all participants in the control group were provided with e-monitors with reminder feature turned off.
(i) DOT for the initial one to two weeks and SAT for the remaining Period
(j) the village doctors visit patients every ten days during the first two months of treatment followed by once a month.
(k) DOTS motivators also conducted follow-up home visits after patients had failed to report to DOTS centers for 1 week.
(l) Monthly visits but patient may return earlier if they experience any problems with treatment.
(m) Assumed from the cost analysis
(n) some take home doses were allowed
(o) community health workers visit TB patients once every 10 days during the intensive phase, and once a month during the continuation phase
(p) missed doses may trigger home or clinic DOT

Table S8 Reported outcomes in included studies

DAT	Author	Treatment Success	Treatment Completion	Cure	LTFU	Death	Treatment Failure	AE	Micro-biological Conversion	Emergence of anti-TB drug resistance	Completion of intensive phase	Recurrence or relapse	Adherence	Satis-faction	HRQOL	Stigma
SMS- based intervention	Ali et al			X	X											
	Bediang et al	X		X	X	X							X	X		
	Belknap et al		X			X		X								
	Dewi et al							X					X			
	Fang et al		X		X								X			
	Farooqi et al	X	X	X	X		X									
	Gashu et al	X	X	X	X	X	X						X	X		
	Hermans et al		X		X	X	X									
	Hirsch-Moverman et al												X			
	Johnston et al		X		X	X		X			X				X	
	Kibu et al												X			
	Kumboyono												X			
	Liu et al	X*			X	X	X	X					X			
	Louwagie et al	X	X	X	X	X	X		X	X	X		X			
	Mohammed et al	X	X	X	X	X	X						X			
	Nguyen et al												X			
	Owiti et al												X			
	Peng et al	X											X			
Video- observed therapy	Bachina et al												X			
	Burzynski et al												X			
	Chen et al		X		X			X					X	X		
	Chuck et al		X		X	X		X								
	Doltu et al	X**										X	X			
	Garfein et al												X			
	Guo et al (a)							X	X			X	X			
	Guo et al (b)	X	X	X	X	X	X							X		
	Lam et al		X										****			
	Lippincott												X			
	Perry et al		X			X			X				X			
	Ravenscroft et al	X						X					X	X		
	Salcedo et al		X		X								X			
	Salerno et al							X								
	Siddiqui et al												X			
	Story et al		X		X	X		X	X				X	X	X	
	Wade et al		X										X			
	Yu et al	X														
Di git	Acosta et al	X											X			
	Broomhead and Mars			X					X							

	Charalambous et al												X			
	Guo et al												X			
	Jerene et al	X			X	X	X									
	Kurada et al	X	X	X	X	X	X									
	Liu et al 2015	X*			X	X	X	X					X			
	Liu et al 2023	X*			X				X			X	X			
	Manyazewal et al 2022				X	X			X				X			
	Manyazewal et al 2023													X		
	Manyazewal et al (b)														X	
	Moulding & Caymittes		X										X			
	Park et al	X	X	X	X	X	X						X			
	Pima et al												X			
	Ratchakit-Nedsuwan et al	X			X	X							X			
	Saha et al		X			X	X						X		X	
	Velen et al	X		X	X	X	X						X			
	Wang et al	X	X	X		X	X									
	Wang et al 2024												X			
	Wei et al	X			X				X				X			
	Wu et al	X	X	X		X	X					X	X			
**Feature-phone based**	Bassett et al		X													
	Das Gupta et al		X		X	X	X								X	
	Hope et al	X			X											
	Hope et al	X											X			
	Khachadourian et al	X	X	X	X	X	X								X	X
	Santra et al												X			
	Sodhi et al	X														
	Yoeli et al	X	X	X	X	X	X									
**99DOTS**	Cattamanchi et al	X			X						X					
	Chen et al	X	X	X	X	X	X									
	Crowder et al	X			X						X					
	Jerene et al	X			X	X	X									
	Thekkur et al	X	X	X	X	X	X									
	Wambi et al		X													
**Ingestible Sensor**	Browne et al							X***					X			
**Smartphone apps**	Haslinda & Juni	X			X	X	X						X			
	Iribarren et al	X	X	X	X	X										
	Wang et al												X			
	Wu et al	X	X	X		X	X					X	X			
	Zhadnova et al		X	X	X											
	Zhang	X	X	X	X	X	X			X			X			
	Zhou et al	X			X	X	X		X				X			

**SMS+Digital pillbox**	Liu et al 2015	X*			X	X	X	X					X			
	Musiimenta et al												X			

*Treatment success was calculated using the combined unfavorable treatment outcome reported; ** Treatment success in VOT group is included in another RCT, *** adverse events reported only in the intervention group, **** Adherence was reported only for the VOT group

Summary of quality assessment evaluation

Study	Random sequence generation (selection bias)	Allocation concealment (selection bias)	Blinding of participants and personnel (performance bias)	Blinding of outcome assessment (detection bias)	Incomplete outcome data (attrition bias)	Selective reporting (reporting bias)	Other bias
1 Bediang et al 2018	+	+	−	−	+	+	+
1 Belknap et al 2017	+	+	?	?	+	+	−
1 Fang et al 2017	?	?	?	?	+	+	−
1 Farooqi et al 2017	+	?	?	?	+	+	−
1 Gashu et al 2021	+	+	−	+	+	−	−
1 Johnston et al 2018	+	+	−	?	+	+	−
1 Kibu et al 2022	+	+	?	+	−	+	+
1 Liu et al 2015	+	?	−	−	+	+	+
1 Louwagie et al 2022	+	+	−	+	−	−	−
1 Mohammed et al 2016	+	+	?	+	+	−	−
2 Burzynski et al 2012	+	+	−	+	?	+	−
2 Guo et al 2020	+	+	−	−	+	+	−
2 Guo et al 2020 (a)	?	?	?	?	?	−	−
2 Ravenscroft et al 2020	+	?	−	−	+	+	−
2 Salerno et al 2023	+	+	−	+	?	+	−
2 Story et al 2019	+	+	−	+	+	+	+
3 Acosta et al 2021	+	+	?	−	+	−	−
3 Jerene et al 2024	+	+	−	+	+	−	+
3 Liu et al 2023	+	?	−	−	+	+	+
3 Manyazewal et al 2022	+	+	−	+	+	+	+
3 Manyazewal et al 2022 (b)	+	+	−	+	+	+	+
3 Manyazewal et al 2023	+	+	−	+	+	+	+
3 Moulding & Caymittes 2002	+	+	−	−	−	+	−
3 Musiimenta et al 2023	+	?	−	+	+	+	−
3 Ratchakit-Nedsuwan et al 2020	?	?	−	?	−	+	+
3 Velen et al 2022	+	+	−	−	+	+	+
3 Wei et al 2023	+	+	−	+	+	+	+
4 Bassett et al 2016	+	+	?	?	+	+	+
4 Khachadourian et al 2020	+	?	−	−	−	+	−
4 Yoeli et al 2019	+	?	−	−	−	+	+
5 Browne et al 2019	+	+	−	?	+	+	−
6 Cattamanchi et al 2021	+	+	−	+	+	+	−
7 Haslinda et Juni 2019	+	+	−	?	+	+	−
7 Iribarren et al 2022	+	+	−	?	+	+	+

1 SMS- based intervention
2 VOT
3 Digital pillbox
4 Feature phone-based
5 Ingestible sensors
6 99DOTS
7 Smartphone app.

Figure S1 Risk of bias summary: review authors' judgements about each risk of bias item for each included study

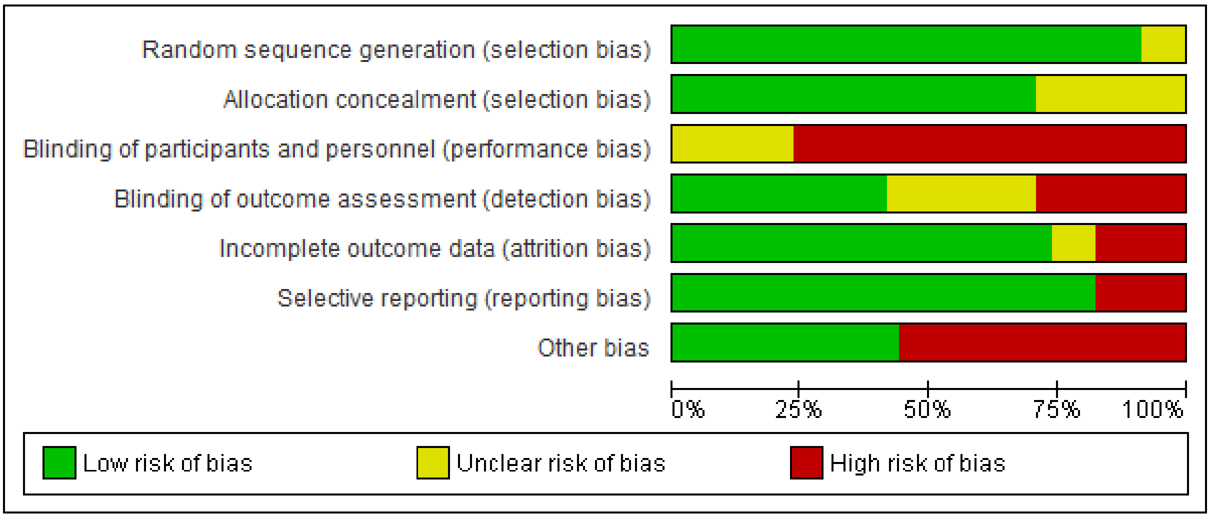

Figure S2 Risk of bias graph: review authors' judgements about each risk of bias item presented as percentages across all included studies.

Table S9 Risk of bias in included observational studies with estimated overall risk of bias

	Selection				Comparability	Outcome			Score	Risk of bias
Santra et al	0	0	0	1	0	0	1	1	3	Poor
Thekkur et al	1	0	1	1	2	0	1	1	7	Good
Dewi et al	1	0	1	1	2	1	1	1	8	Good
Chuck et al	0	1	1	1	0	0	1	1	5	Poor
Ali et al	0	0	1	1	1	0	1	1	5	Fair
Park et al	0	1	1	0	2	0	1	0	5	Poor
Wang et al	1	1	1	1	1	0	1	1	7	Good
Broomhead and Mars	0	1	1	1	2	0	1	0	6	Poor
Perry et al	0	1	1	1	0	0	1	0	4	Poor
Wade et al	1	1	1	1	2	0	1	0	7	Poor
Chen et al	1	1	1	1	2	0	1	1	8	Good
Das Gupta et al	0	1	1	1	0	0	1	1	5	Poor
Guo et al	0	0	1	1	0	0	1	1	4	Poor
Zhadnova et al	0	1	1	1	2	0	1	0	6	Poor
Doltu et al	0	0	1	1	2	0	1	1	6	Fair
Kumboyono et al	1	1	1	1	2	0	0	0	6	Poor
Garfein et al	1	1	1	1	2	0	1	1	8	Good
Hermans et al	1	0	1	1	2	0	1	1	7	Good
Hirsch-Moverman et al	1	0	1	1	0	0	1	0	4	Poor
Lam et al	0	0	1	1	2	0	1	1	6	Fair
Salcedo et al	0	0	1	1	2	0	1	1	6	Fair
Lippincott et al	0	1	1	1	0	0	0	1	4	Poor
Chen et al	1	1	1	1	2	0	1	0	7	Poor
Bachina et al	0	1	1	1	0	0	1	1	5	Poor
Siddiqui	0	1	1	1	1	0	1	1	6	Good
Zhang et al	1	1	1	1	2	0	0	0	6	Poor
Saha et al	1	1	1	1	0	0	0	1	5	Poor
Crowder et al	1	0	1	1	1	0	1	1	6	Good

Wu et al	1	1	1	1	1	0	0	0	5	Poor
Wang et al	1	1	1	1	0	1	0	0	5	Poor
Sodhi et al	1	1	1	1	1	1	1	0	4	Fair

The Thresholds for converting the Newcastle-Ottawa scales to AHRQ standards (good, fair, and poor) was used: **Good quality:** 3 or 4 stars in selection domain AND 1 or 2 stars in comparability domain AND 2 or 3 stars in outcome/exposure domain. **Fair quality:** 2 stars in selection domain AND 1 or 2 stars in comparability domain AND 2 or 3 stars in outcome/exposure domain. **Poor quality:** 0 or 1 star in selection domain OR 0 stars in comparability domain OR 0 or 1 stars in outcome/exposure domain.

Table S10 Risk of bias in included observational studies

	Selection	Comparability of cohorts	Outcome
	****	**	***
Santra et al	*	-	**
Thekkur et al	***	**	**
Dewi et al	***	**	***
Chuck et al	***	-	**
Ali et al	**	*	**
Park et al	**	**	*
Wang et al	****	*	**
Broomhead and Mars	***	**	*
Perry et al	***	-	*
Wade et al	****	**	*
Chen et al	****	**	**
Das Gupta et al	***	-	**
Guo et al	**	-	**
Zhadnova et al	***	**	*
Doltu et al	**	**	**
Kumboyono et al	****	**	-
Garfein et al	****	**	**
Hermans et al	***	**	**
Hirsch-Moverman et al	***	-	*
Lam et al	**	**	**
Salcedo et al	**	**	**
Lippincott et al	***	-	*
Chen et al	****	**	*
Bachina et al	***	-	**
Siddiqui	***	*	**
Zhang et al	****	**	-
Saha et al	****	-	*
Crowder et al	***	*	**
Wu et al	****	*	-
Wang et al	****	-	*
Sodhi et al	****	*	**

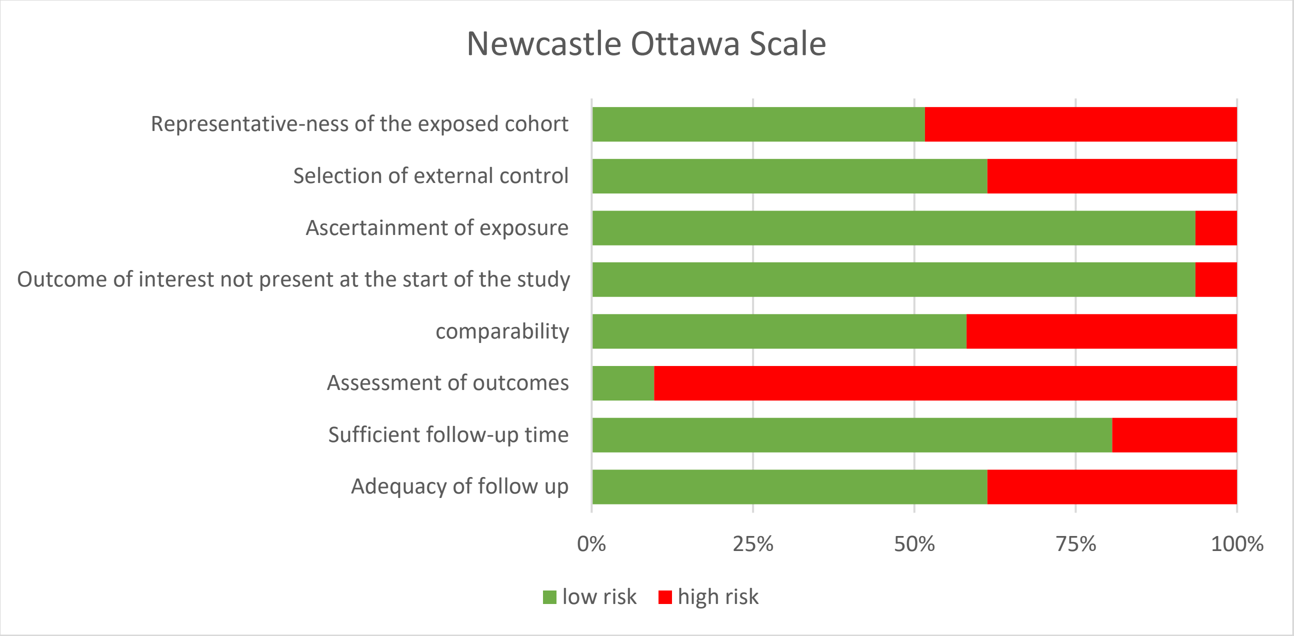

Figure S3 Risk of bias graph: review authors' judgements about each risk of bias item presented as percentages across included observational studies

Grading evidence

Table S11 Grading the evidence from RCTs

What is the impact of digital adherence technologies versus standard care on health outcomes in the treatment of active tuberculosis? (RCTs)

Patient or population: Patients with active TB; **Setting:** Clinics, hospitals or other; **Intervention:** DAT; **Comparison:** Standard care (DOT or SAT)

Outcomes	№ of studies	№ of participants	Certainty of the evidence (GRADE)	Relative effect (95% CI)	Anticipated absolute effects	
					Risk with standard care	Risk difference with DAT
Treatment success - All	34	52879	⨁⊖⊖⊖ Very low [a, b, c]	1.14 (0.99 to 1.3)	819 per 1,000	19 more per 1,000 (1 fewer to 36 more)
Treatment success - SMS	8	16986	⨁⨁⊖⊖ low [a, d]	1.28 (0.96 to 1.7)	836 per 1,000	31 more per 1,000 (5 fewer to 61 more)
Treatment success - VOT	3	6439	⨁⨁⊖⊖ low [a, d]	1.53 (0.95 to 2.44)	875 per 1,000	39 more per 1,000 (5 fewer to 70 more)
Treatment success - Digital pillbox	11	809	⨁⨁⊖⊖ low [a, b]	1.03 (0.86 to 1.23)	781 per 1,000	5 more per 1,000 (27 fewer to 33 more)
Treatment success - Feature phone-based	4	23778	⨁⊖⊖⊖ very low [a, b, d]	1.43 (0.61 to 3.34)	787 per 1,000	54 more per 1,000 (94 fewer to 138 more)
Treatment success - 99DOTS	4	2323	⨁⨁⊖⊖ low [a, b]	0.82 (0.64 to 1.05)	859 per 1,000	26 fewer per 1,000 (63 fewer to 6 more)
Treatment success - Smartphone apps	3	376	⨁⨁⊖⊖ low [a, d]	2.17 (1.07 to 4.4)	718 per 1,000	129 more per 1,000 (13 more to 200 more)
Treatment success - SMS and Digital pillbox	1	2168	-	1 (0.45 to 2.21)	886 per 1,000	0 more per 1,000 (107 fewer to 59 more)
Loss to follow up - All	30	51340	⨁⨁⊖⊖ low [a, b]	0.84 (0.66 to 1.06)	80 per 1,000	12 fewer per 1,000 (26 fewer to 4 more)
Loss to follow up - SMS	8	5976	⨁⨁⊖⊖ low [a, b]	0.9 (0.57 to 1.4)	114 per 1,000	11 fewer per 1,000 (45 fewer to 39 more)
Loss to follow up - VOT	2	631	⨁⨁⊖⊖ low [a, d]	0.78 (0.12 to 5.12)	22 per 1,000	5 fewer per 1,000 (19 fewer to 81 more)

What is the impact of digital adherence technologies versus standard care on health outcomes in the treatment of active tuberculosis? (RCTs)						
**Patient or population:** Patients with active TB; **Setting:** Clinics, hospitals or other; **Intervention:** DAT; **Comparison:** Standard care (DOT or SAT)						
Outcomes	№ of studies	№ of participants	Certainty of the evidence (GRADE)	Relative effect (95% CI)	Anticipated absolute effects	
					Risk with standard care	Risk difference with DAT
Loss to follow up - Digital pillbox	10	23663	⨁⨁⨁◯ moderate [a]	0.87 (0.64 to 1.19)	79 per 1,000	9 fewer per 1,000 (27 fewer to 14 more)
Loss to follow up -Feature phone-based	2	1540	⨁◯◯◯ very low [a, b, d]	0.49 (0.06 to 4.35)	85 per 1,000	41 fewer per 1,000 (80 fewer to 203 more)
Loss to follow up - 99DOTS	4	16986	⨁◯◯◯ very low [a, b, d]	1.11 (0.8 to 1.54)	67 per 1,000	7 more per 1,000 (13 fewer to 33 more)
Loss to follow up - Smartphone apps	3	376	⨁⨁⨁◯ moderate [a]	0.35 (0.08 to 1.53)	112 per 1,000	70 fewer per 1,000 (102 fewer to 50 more)
Loss to follow up - SMS and Digital pillbox	1	2168	-	0.9 (0.38 to 2.11)	106 per 1,000	10 fewer per 1,000 (63 fewer to 94 more)
Treatment failure - All	17	35628	⨁⨁⨁◯ moderate [a]	1.05 (0.66 to 1.65)	12 per 1,000	1 more per 1,000 (4 fewer to 8 more)
Treatment failure - SMS	5	5347	⨁⨁◯◯ low [a, d]	0.93 (0.59 to 1.48)	15 per 1,000	1 fewer per 1,000 (6 fewer to 7 more)
Treatment failure - VOT	1	405	-	0.79 (0.21 to 2.99)	25 per 1,000	5 fewer per 1,000 (20 fewer to 46 more)
Treatment failure - Digital pillbox	3	5466	⨁⨁◯◯ low [a, d]	1.74 (0.83 to 3.63)	12 per 1,000	9 more per 1,000 (2 fewer to 30 more)
Treatment failure - Feature phone-based	2	1540	⨁◯◯◯ very low [a, b, d]	0.49 (0.06 to 4.35)	85 per 1,000	41 fewer per 1,000 (80 fewer to 203 more)
Treatment failure - Smartphone apps	2	334	⨁⨁◯◯ low [a, d]	0.7 (0.33 to 1.48)	108 per 1,000	30 fewer per 1,000 (70 fewer to 44 more)
Treatment failure - SMS and Digital pillbox	1	2168	-	1.07 (0.22 to 5.34)	3 per 1,000	0 more per 1,000 (2 fewer to 13 more)

What is the impact of digital adherence technologies versus standard care on health outcomes in the treatment of active tuberculosis? (RCTs)						
**Patient or population:** Patients with active TB; **Setting:** Clinics, hospitals or other; **Intervention:** DAT; **Comparison:** Standard care (DOT or SAT)						
**Outcomes**	**№ of studies**	**№ of participants**	**Certainty of the evidence (GRADE)**	**Relative effect (95% CI)**	**Anticipated absolute effects**	
					**Risk with standard care**	**Risk difference with DAT**
Treatment failure - Digital pillbox or 99DOTS	3	20368	⊕⊕⊕⊖ **moderate** [a]	2.12 (1.34 to 3.37)	3 per 1,000	3 more per 1,000 (1 more to 7 more)
Death – All	24	45965	⊕⊕⊖⊖ **low** [a, d]	1.34 (1.18 to 1.53)	29 per 1,000	9 more per 1,000 (5 more to 15 more)
Death – SMS	5	5478	⊕⊕⊖⊖ **low** [a, d]	1.32 (0.78 to 2.23)	16 per 1,000	5 more per 1,000 (4 fewer to 19 more)
Death – VOT	2	631	⊕⊕⊖⊖ **low** [a, d]	0.82 (0.05 to 13.22)	3 per 1,000	1 fewer per 1,000 (3 fewer to 35 more)
Death - Digital pillbox	8	20699	⊕⊕⊖⊖ **low** [a, d]	1.33 (1.11 to 1.58)	57 per 1,000	17 more per 1,000 (6 more to 27 more)
Death - Feature phone-based	2	1540	⊕⊕⊖⊖ **low** [a, d]	0.62 (0.3 to 1.3)	24 per 1,000	9 fewer per 1,000 (17 fewer to 7 more)
Death - 99DOTS	3	15073	⊕⊕⊖⊖ **low** [a, b]	1.54 (1.04 to 2.28)	59 per 1,000	29 more per 1,000 (2 more to 66 more)
Death - Smartphone apps	3	376	⊕⊕⊖⊖ **low** [a, d]	0.95 (0.41 to 2.23)	64 per 1,000	3 fewer per 1,000 (37 fewer to 68 more)
Death - SMS and Digital pillbox	1	2168	-	1.27 (0.42 to 3.79)	6 per 1,000	2 more per 1,000 (3 fewer to 16 more)
Reporting of adverse events - All	6	7062	⊕⊕⊕⊖ **moderate** [a]	1.57 (1.25 to 1.97)	49 per 1,000	26 more per 1,000 (12 more to 43 more)
Reporting of adverse events - SMS	1	2112	-	1.24 (0.79 to 1.93)	34 per 1,000	8 more per 1,000 (7 fewer to 30 more)
Reporting of adverse events - VOT	3	620	⊕⊕⊕⊖ **moderate** [a]	1.9 (1.27 to 2.84)	230 per 1,000	132 more per 1,000 (45 more to 229 more)

What is the impact of digital adherence technologies versus standard care on health outcomes in the treatment of active tuberculosis? (RCTs)						
**Patient or population:** Patients with active TB; **Setting:** Clinics, hospitals or other; **Intervention:** DAT; **Comparison:** Standard care (DOT or SAT)						
**Outcomes**	**№ of studies**	**№ of participants**	**Certainty of the evidence (GRADE)**	**Relative effect (95% CI)**	**Anticipated absolute effects**	
					**Risk with standard care**	**Risk difference with DAT**
Reporting of adverse events - Digital pillbox	1	2101	-	1.22 (0.78 to 1.91)	34 per 1,000	7 more per 1,000 (7 fewer to 29 more)
Reporting of adverse events - SMS and Digital pillbox	1	2168	-	1.91 (1.27 to 2.88)	34 per 1,000	29 more per 1,000 (9 more to 58 more)
Cure - All	10	5751	⨁⨁⨁⊖ **moderate** [a]	1.15 (0.97 to 1.36)	447 per 1,000	35 more per 1,000 (8 fewer to 77 more)
Cure - SMS	5	3514	⨁⨁⊖⊖ **low** [a, d]	1.05 (0.92 to 1.21)	475 per 1,000	13 more per 1,000 (21 fewer to 48 more)
Cure - VOT	1	405	-	1.55 (0.81 to 2.96)	876 per 1,000	40 more per 1,000 (25 fewer to 78 more)
Cure - Digital pillbox	1	250	-	0.54 (0.27 to 1.1)	190 per 1,000	77 fewer per 1,000 (131 fewer to 15 more)
Cure - Feature phone-based	2	1540	⨁⨁⊖⊖ **low** [a, d]	1.3 (1.05 to 1.62)	323 per 1,000	60 more per 1,000 (10 more to 114 more)
Cure - Smartphone apps	1	42	-	20.5 (1.07 to 391.07)	24 per 1,000	311 more per 1,000 (2 more to 882 more)
Microbiologic conversion - All	6	4102	⨁⨁⊖⊖ **low** [a, b]	1.36 (0.75 to 2.47)	789 per 1,000	47 more per 1,000 (52 fewer to 113 more)
Microbiologic conversion - SMS	1	574	-	1.16 (0.78 to 1.73)	399 per 1,000	37 more per 1,000 (57 fewer to 136 more)
Microbiologic conversion - VOT	1	226	-	0.82 (0.16 to 4.25)	954 per 1,000	9 fewer per 1,000 (187 fewer to 35 more)
Microbiologic conversion - Digital pillbox	3	3078	⨁⨁⊖⊖ **low** [a, b]	0.88 (0.6 to 1.3)	907 per 1,000	11 fewer per 1,000 (53 fewer to 20 more)

What is the impact of digital adherence technologies versus standard care on health outcomes in the treatment of active tuberculosis? (RCTs)						
**Patient or population:** Patients with active TB; **Setting:** Clinics, hospitals or other; **Intervention:** DAT; **Comparison:** Standard care (DOT or SAT)						
**Outcomes**	**№ of studies**	**№ of participants**	**Certainty of the evidence (GRADE)**	**Relative effect (95% CI)**	**Anticipated absolute effects**	
					**Risk with standard care**	**Risk difference with DAT**
Microbiologic conversion - Smartphone apps	1	224	-	5.06 (2.57 to 9.96)	418 per 1,000	366 more per 1,000 (231 more to 459 more)

*The number of total participants may include repeated control groups
The risk in the intervention group (and its 95% confidence interval) is based on the assumed risk in the comparison group and the **relative effect** of the intervention (and its 95% CI).
CI: confidence interval; **OR:** odds ratio
a) High risk of bias
b) Inconsistency due unexplained heterogeneity of results I^2> 50%
c) Publication bias
d) Imprecision: some studies include relatively few patients and few events and thus have a wide confidence interval (CI) around the estimate or sample size is lower than the optimal information size (OIS)

GRADE Working Group grades of evidence
High certainty: we are very confident that the true effect lies close to that of the estimate of the effect.
Moderate certainty: we are moderately confident in the effect estimate: the true effect is likely to be close to the estimate of the effect, but there is a possibility that it is substantially different.
Low certainty: our confidence in the effect estimate is limited: the true effect may be substantially different from the estimate of the effect.
Very low certainty: we have very little confidence in the effect estimate: the true effect is likely to be substantially different from the estimate of effect.

Table S12 Grading the evidence from observational studies

What is the impact of digital adherence technologies versus standard care on health outcomes in the treatment of active tuberculosis? (observational studies)						
**Patient or population:** Patients with active TB; **Setting:** Clinics, hospitals or other; **Intervention:** DAT; **Comparison:** Standard care (DOT or SAT)						
**Outcomes**	**№ of studies**	**№ of participants**	**Certainty of the evidence (GRADE)**	**Relative effect (95% CI)**	**Anticipated absolute effects**	
					**Risk with standard care**	**Risk difference with DAT**
Treatment success - All	22	34124	⊕⊖⊖⊖ **Very low** [a, b, c]	1.11 (0.94 to 1.3)	898 per 1,000	9 more per 1,000 (6 fewer to 22 more)
Treatment success - SMS	1	485	-	0.81 (0.54 to 1.22)	742 per 1,000	42 fewer per 1,000 (134 fewer to 36 more)
Treatment success - VOT	5	933	⊕⊕⊖⊖ **low** [a, d]	1.56 (0.93 to 2.63)	813 per 1,000	59 more per 1,000 (12 fewer to 106 more)
Treatment success - Digital pillbox	5	6139	⊕⊕⊖⊖ **low** [a, b]	1.23 (0.75 to 2.02)	912 per 1,000	15 more per 1,000 (25 fewer to 42 more)
Treatment success - Feature phone-based	4	1780	⊕⊖⊖⊖ **very low** [a, b, d]	0.95 (0.31 to 2.96)	866 per 1,000	6 fewer per 1,000 (201 fewer to 84 more)
Treatment success - 99DOTS	4	21842	⊕⊕⊖⊖ **low** [a, b]	0.99 (0.74 to 1.32)	906 per 1,000	1 fewer per 1,000 (30 fewer to 21 more)
Treatment success - Smartphone apps	3	2945	⊕⊖⊖⊖ **very low** [a, b, d]	1.51 (0.53 to 4.3)	535 per 1,000	99 more per 1,000 (157 fewer to 297 more)
Loss to follow up - All	14	29855	⊕⊕⊖⊖ **low** [a, b]	0.66 (0.38 to 1.16)	44 per 1,000	14 fewer per 1,000 (27 fewer to 7 more)
Loss to follow up - SMS	2	633	⊕⊕⊖⊖ **low** [a, b]	0.8 (0.41 to 1.56)	56 per 1,000	11 fewer per 1,000 (32 fewer to 29 more)
Loss to follow up - VOT	2	504	⊕⊕⊖⊖ **low** [a, d]	1.54 (0.36 to 6.64)	15 per 1,000	8 more per 1,000 (10 fewer to 77 more)
Loss to follow up - Digital pillbox	2	3751	⊕⊕⊖⊖ **low** [a, d]	0.04 (0.01 to 0.1)	17 per 1,000	16 fewer per 1,000 (17 fewer to -15 more)

What is the impact of digital adherence technologies versus standard care on health outcomes in the treatment of active tuberculosis? (observational studies)						
**Patient or population:** Patients with active TB; **Setting:** Clinics, hospitals or other; **Intervention:** DAT; **Comparison:** Standard care (DOT or SAT)						
Outcomes	№ of studies	№ of participants	Certainty of the evidence (GRADE)	Relative effect (95% CI)	Anticipated absolute effects	
					Risk with standard care	Risk difference with DAT
Loss to follow up - Feature-Feature phone-based	3	791	⨁⊖⊖⊖ **very low** [a, b, d]	2.12 (0.37 to 12.15)	55 per 1,000	55 more per 1,000 (34 fewer to 359 more)
Loss to follow up - 99DOTS	3	21401	⨁⊖⊖⊖ **very low** [a, b, d]	0.92 (0.54 to 1.57)	30 per 1,000	2 fewer per 1,000 (13 fewer to 16 more)
Loss to follow up - Smartphone apps	2	2775	⨁⨁⨁⊖ **moderate** [a]	0.24 (0.05 to 1.05)	14 per 1,000	11 fewer per 1,000 (13 fewer to 1 more)
Treatment failure - All	12	27723	⨁⨁⨁⊖ **moderate** [a]	1 (0.97 to 1.02)	35 per 1,000	0 more per 1,000 (1 fewer to 1 more)
Treatment failure - SMS	1	485	-	4.21 (0.81 to 21.95)	7 per 1,000	22 more per 1,000 (1 fewer to 127 more)
Treatment failure - Digital pillbox	5	6139	⨁⨁⊖⊖ **low** [a, d]	1 (0.98 to 1.02)	8 per 1,000	0 more per 1,000 (0 fewer to 0 more)
Treatment failure - Feature-Feature phone-based	2	333	⨁⊖⊖⊖ **very low** [a, b, d]	0.76 (0.09 to 6.53)	9 per 1,000	2 fewer per 1,000 (8 fewer to 47 more)
Treatment failure - 99DOTS	2	17875	⨁⨁⊖⊖ **low** [a, d]	0.9 (0.24 to 3.46)	5 per 1,000	0 more per 1,000 (4 fewer to 12 more)
Treatment failure - Smartphone app	2	2891	⨁⨁⊖⊖ **low** [a, d]	1.75 (0.22 to 13.56)	1 per 1,000	1 more per 1,000 (1 fewer to 12 more)
Death - All	14	28276	⨁⨁⊖⊖ **low** [a, d]	1.16 (1.01 to 1.33)	47 per 1,000	7 more per 1,000 (0 fewer to 15 more)
Death - SMS	1	485	-	0.94 (0.56 to 1.57)	156 per 1,000	8 fewer per 1,000 (62 fewer to 69 more)
Death - VOT	2	553	⨁⨁⊖⊖ **low** [a, d]	0.78 (0.19 to 3.3)	46 per 1,000	10 fewer per 1,000 (37 fewer to 91 more)

What is the impact of digital adherence technologies versus standard care on health outcomes in the treatment of active tuberculosis? (observational studies)						
**Patient or population:** Patients with active TB; **Setting:** Clinics, hospitals or other; **Intervention:** DAT; **Comparison:** Standard care (DOT or SAT)						
**Outcomes**	**№ of studies**	**№ of participants**	**Certainty of the evidence (GRADE)**	**Relative effect (95% CI)**	**Anticipated absolute effects**	
					**Risk with standard care**	**Risk difference with DAT**
Death - Digital pillbox	5	6139	⨁⨁◯◯ **low** [a, d]	0.7 (0.48 to 1.04)	38 per 1,000	11 fewer per 1,000 (20 fewer to 2 more)
Death - Feature-Feature phone-based	2	333	⨁⨁◯◯ **low** [a, d]	6.14 (0.73 to 51.42)	5 per 1,000	25 more per 1,000 (1 fewer to 200 more)
Death - 99DOTS	2	17875	⨁⨁◯◯ **low** [a, d]	1.25 (1.1 to 1.42)	52 per 1,000	12 more per 1,000 (5 more to 20 more)
Death - Smartphone apps	2	2891	⨁⨁◯◯ **low** [a, d]	1.48 (0.83 to 2.64)	18 per 1,000	8 more per 1,000 (3 fewer to 28 more)
Reporting of adverse events - All	3	903	⨁⨁⨁◯ **moderate** [a]	1.39 (0.93 to 2.09)	126 per 1,000	41 more per 1,000 (8 fewer to 105 more)
Reporting of adverse events - SMS	1	120	-	1.22 (0.6 to 2.5)	500 per 1,000	50 more per 1,000 (126 fewer to 215 more)
Reporting of adverse events - VOT	2	487	⨁⨁◯◯ **low** [a, d]	1.48 (0.91 to 2.42)	73 per 1,000	32 more per 1,000 (6 fewer to 87 more)
Cure - All	11	26827	⨁⨁⨁◯ **moderate** [a]	1.17 (0.86 to 1.59)	489 per 1,000	39 more per 1,000 (38 fewer to 114 more)
Cure - SMS	1	148	-	2.47 (1.2 to 5.09)	595 per 1,000	189 more per 1,000 (43 more to 287 more)
Cure - MERM	5	5859	⨁⨁◯◯ **low** [a, b]	1.65 (1.03 to 2.66)	425 per 1,000	125 more per 1,000 (6 more to 238 more)
Cure - 99DOTS	2	17875	⨁⨁◯◯ **low** [a, b]	0.51 (0.16 to 1.64)	425 per 1,000	151 fewer per 1,000 (320 fewer to 123 more)
Cure - Smartphone app	3	2945	⨁⨁◯◯ **low** [a, d]	1.13 (0.57 to 2.24)	401 per 1,000	29 more per 1,000 (126 fewer to 199 more)

What is the impact of digital adherence technologies versus standard care on health outcomes in the treatment of active tuberculosis? (observational studies)						
**Patient or population:** Patients with active TB; **Setting:** Clinics, hospitals or other; **Intervention:** DAT; **Comparison:** Standard care (DOT or SAT)						
Outcomes	№ of studies	№ of participants	Certainty of the evidence (GRADE)	Relative effect (95% CI)	Anticipated absolute effects	
					Risk with standard care	Risk difference with DAT
Microbiologic conversion - VOT	1	393	-	2.97 (1.33 to 6.67)	818 per 1,000	132 more per 1,000 (37 fewer to 172 more)
Microbiologic conversion - Digital pillbox	1	120	-	4.22 (0.79 to 22.53)	384 per 1,000	241 more per 1,000 (14 more to 424 more)

*The number of total participants may include repeated control groups
The risk in the intervention group (and its 95% confidence interval) is based on the assumed risk in the comparison group and the **relative effect** of the intervention (and its 95% CI).
CI: confidence interval; **OR:** odds ratio
a) Risk of bias
b) Inconsistency due unexplained heterogeneity of results I2> 50%
c) Publication bias
d) Imprecision: some studies include relatively few patients and few events and thus have a wide confidence interval (CI) around the estimate or sample size is lower than the optimal information size (OIS)

GRADE Working Group grades of evidence
High certainty: we are very confident that the true effect lies close to that of the estimate of the effect.
Moderate certainty: we are moderately confident in the effect estimate: the true effect is likely to be close to the estimate of the effect, but there is a possibility that it is substantially different.
Low certainty: our confidence in the effect estimate is limited: the true effect may be substantially different from the estimate of the effect.
Very low certainty: we have very little confidence in the effect estimate: the true effect is likely to be substantially different from the estimate of effect.

What is the impact of digital adherence technologies versus standard care on health outcomes in the treatment of latent tuberculosis?					
**Patient or population:** Patients infected with TB<br>**Setting:** Clinics, hospitals or other<br>**Intervention:** DAT<br>**Comparison:** Standard care (DOT or SAT)					
**Outcomes**	**№ of participants (studies) Follow-up**	**Certainty of the evidence (GRADE)**	**Relative effect (95% CI)**	**Anticipated absolute effects**	
				**Risk with standard care**	**Risk difference with DAT**
LTBI- Treatment completion – SMS (RCT)	1023<br>(2 studies)	⨁⨁⊖⊖<br>**low** [a, b]	**OR 1.05**<br>(0.78 to 1.40)	767 per 1,000	**9 more per 1,000**<br>(47 fewer to 55 more)
LTBI- Treatment completion – VOT (observational)	592<br>(2 studies)	⨁⨁⊖⊖<br>**low** [a, c]	**OR 4.69**<br>(2.08 to 10.55)	727 per 1,000	**199 more per 1,000**<br>(120 more to 238 more)

*__The risk in the intervention group__ (and its 95% confidence interval) is based on the assumed risk in the comparison group and the **relative effect** of the intervention (and its 95% CI).
CI: confidence interval; **OR:** odds ratio
a) Risk of bias
b) Inconsistency due unexplained heterogeneity of results I^2> 50%
c) imprecision

GRADE Working Group grades of evidence
High certainty: we are very confident that the true effect lies close to that of the estimate of the effect.
Moderate certainty: we are moderately confident in the effect estimate: the true effect is likely to be close to the estimate of the effect, but there is a possibility that it is substantially different.
Low certainty: our confidence in the effect estimate is limited: the true effect may be substantially different from the estimate of the effect.
Very low certainty: we have very little confidence in the effect estimate: the true effect is likely to be substantially different from the estimate of effect.

Quantitative synthesis of evidence- TB disease

Treatment success

Study	DAT	CIL	N DAT	N Control	OR [95% CI]	Weight
Study_Design = RCT						
Cattamanchi et al	99DOTS	Low	891	1022	1.04 [0.68; 1.59]	2.6%
Jerene et al - Philippines	99DOTS	Lower middle	2368	4188	0.87 [0.51; 1.48]	2.0%
Jerene et al - South Africa	99DOTS	Upper middle	1213	1880	0.84 [0.59; 1.21]	3.0%
Jerene et al - Tanzania	99DOTS	Lower middle	1768	3656	0.55 [0.34; 0.91]	2.2%
Acosta et al	Digital pillbox	Upper middle	53	53	1.71 [0.52; 5.60]	0.6%
Jerene et al - Philippines	Digital pillbox	Lower middle	2236	4188	0.90 [0.53; 1.52]	2.0%
Jerene et al - South Africa	Digital pillbox	Upper middle	1281	1880	0.82 [0.57; 1.18]	3.0%
Jerene et al - Tanzania	Digital pillbox	Lower middle	1778	3656	0.79 [0.50; 1.25]	2.4%
Jerene et al - Ukraine	Digital pillbox	Lower middle	1526	1589	0.87 [0.63; 1.20]	3.2%
Liu et al (2015)	Digital pillbox	Upper middle	997	1104	1.41 [0.66; 3.01]	1.2%
Liu et al (2023)	Digital pillbox	Upper middle	1298	1388	0.97 [0.69; 1.36]	3.2%
Moulding & Caymittes	Digital pillbox	Lower middle	64	59	2.41 [0.77; 7.52]	0.6%
Ratchakit-Nedsuwan et al	Digital pillbox	Upper middle	50	50	0.46 [0.08; 2.67]	0.3%
Velen et al	Digital pillbox	Lower middle	124	126	1.11 [0.63; 1.97]	1.8%
Wei et al	Digital pillbox	Upper middle	143	135	1.29 [1.15; 1.44]	4.8%
Bassett et al	Feature-phone	Upper middle	293	230	0.87 [0.62; 1.24]	3.1%
Hope et al	Feature-phone	Low	129	131	2.29 [0.56; 9.34]	0.4%
Khachadourian et al	Feature-phone	Upper middle	227	209	0.70 [0.35; 1.42]	1.4%
Yoeli et al	Feature-phone	Lower middle	569	535	3.42 [2.12; 5.52]	2.3%
Haslinda & Juni	Smartphone app	Upper middle	55	55	4.14 [1.15; 14.89]	0.5%
Iribarren et al	Smartphone app	Upper middle	21	21	4.71 [0.48; 46.22]	0.2%
Zhou et al	Smartphone app	Upper middle	112	112	1.53 [0.83; 2.81]	1.7%
Bediang et al	SMS	Lower middle	137	142	1.45 [0.82; 2.56]	1.8%
Fang et al	SMS	Upper middle	160	190	3.89 [1.55; 9.74]	0.9%
Farooqi et al	SMS	Lower middle	74	74	1.27 [0.33; 4.92]	0.5%
Gashu et al	SMS	Low	152	154	1.49 [0.75; 2.95]	1.4%
Liu et al	SMS	Upper middle	1008	1104	2.27 [0.88; 5.86]	0.9%
Louwagie et al	SMS	Upper middle	283	291	0.86 [0.60; 1.24]	3.0%
Mohammed et al	SMS	Lower middle	1110	1097	1.02 [0.81; 1.28]	4.0%
Peng et al	SMS	Upper middle	234	229	1.10 [0.52; 2.34]	1.3%
Liu et al	SMS+ Digital pillbox	Upper middle	1064	1104	1.00 [0.45; 2.21]	1.2%
Guo et al (b)	VOT	Upper middle	203	202	1.40 [0.55; 3.57]	0.9%
Ravenscroft et al	VOT	Upper middle	85	93	1.71 [0.55; 5.33]	0.6%
Story et al	VOT	High	112	114	1.53 [0.82; 2.85]	1.6%
Total (95% CI)					1.14 [0.99; 1.30]	60.6%
Prediction interval					[0.66; 1.97]	--
Heterogeneity: $Tau^2 = 0.0679$; $Chi^2 = 76.68$, df = 33 ($P < 0.01$); $I^2 = 57\%$ [37%; 71%]						
Study_Design = observational						
Chen et al.	99DOTS	Lower middle	8322	7722	0.97 [0.85; 1.10]	4.7%
Crowder et al	99DOTS	Low	2051	1475	1.30 [1.06; 1.61]	4.1%
Thekkur et al	99DOTS	Lower middle	870	961	0.70 [0.57; 0.86]	4.2%
Wambi et al	99DOTS	Low	309	132	1.65 [0.50; 5.47]	0.6%
Kurada et al	Digital pillbox	Lower middle	385	3019	1.59 [0.89; 2.81]	1.8%
Park et al	Digital pillbox	Lower middle	206	141	4.33 [2.13; 8.81]	1.4%
Saha et al	Digital pillbox	Lower middle	200	200	0.69 [0.45; 1.04]	2.6%
Wang et al (2020)	Digital pillbox	Upper middle	1047	763	1.00 [0.98; 1.03]	5.1%
Wu et al	Digital pillbox	Upper middle	90	88	0.49 [0.12; 2.04]	0.4%
Das Gupta et al	Feature-phone	Lower middle	111	111	0.35 [0.14; 0.87]	0.9%
Das Gupta et al	Feature-phone	Lower middle	111	111	0.31 [0.12; 0.76]	0.9%
Hope et al	Feature-phone	Low	210	248	1.93 [1.08; 3.45]	1.8%
Sodhi et al	Feature-phone	Lower middle	276	713	3.41 [1.70; 6.86]	1.4%
Wu et al	Smartphone app	Upper middle	82	88	0.69 [0.15; 3.17]	0.4%
Zhadnova et al	Smartphone app	Upper middle	44	10	7.00 [1.32; 37.07]	0.3%
Zhang et al	Smartphone app	Upper middle	1145	1576	1.12 [0.79; 1.58]	3.1%
Hermans et al	SMS	Low	183	302	0.81 [0.54; 1.22]	2.7%
Chuck et al	VOT	High	61	329	0.63 [0.13; 3.14]	0.3%
Perry et al	VOT	High	94	69	2.67 [0.73; 9.76]	0.5%
Salcedo et al	VOT	High	43	71	1.10 [0.34; 3.54]	0.6%
Wade et al	VOT	High	58	70	1.82 [0.85; 3.89]	1.2%
Yu et al	VOT	High	39	99	1.70 [0.22; 13.22]	0.2%
Total (95% CI)					1.11 [0.94; 1.30]	39.4%
Prediction interval					[0.65; 1.88]	--
Heterogeneity: $Tau^2 = 0.0574$; $Chi^2 = 82.18$, df = 21 ($P < 0.01$); $I^2 = 74\%$ [61%; 83%]						
**Total (95% CI)**					**1.11 [1.01; 1.22]**	**100.0%**
**Prediction interval**					**[0.71; 1.74]**	
Heterogeneity: $Tau^2 = 0.0474$; $Chi^2 = 169.93$, df = 55 ($P < 0.01$); $I^2 = 68\%$ [57%; 75%]						
Test for subgroup differences: $Chi^2 = 0.06$, df = 1 ($P = 0.81$)						

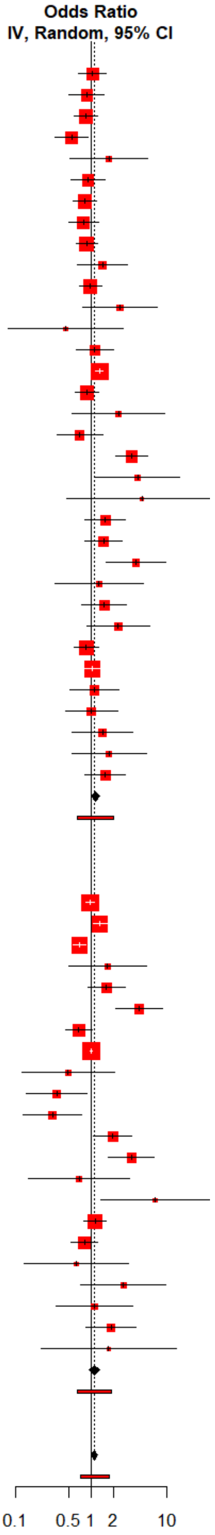

Figure S4a Forest plot showing treatment success in DAT groups compared to standard of care stratified by study design

Study	N DAT	N Control	OR [95% CI]	Weight
DAT = 99DOTS				
Cattamanchi et al	891	1022	1.04 [0.68; 1.59]	4.2%
Jerene et al - Philippines	2368	4188	0.87 [0.51; 1.48]	3.4%
Jerene et al - South Africa	1213	1880	0.84 [0.59; 1.21]	4.7%
Jerene et al - Tanzania	1768	3656	0.55 [0.34; 0.91]	3.7%
Total (95% CI)			**0.82 [0.64; 1.05]**	**16.0%**
Heterogeneity: Tau^2 = 0.0124; Chi^2 = 3.72, df = 3 (P = 0.29); I^2 = 19% [0%; 88%]				
DAT = Feature-phone				
Bassett et al	293	230	0.87 [0.62; 1.24]	4.8%
Hope et al	129	131	2.29 [0.56; 9.34]	0.8%
Khachadourian et al	227	209	0.70 [0.35; 1.42]	2.5%
Yoeli et al	569	535	3.42 [2.12; 5.52]	3.8%
Total (95% CI)			**1.43 [0.61; 3.34]**	**11.9%**
Heterogeneity: Tau^2 = 0.6063; Chi^2 = 24.1, df = 3 (P < 0.01); I^2 = 88% [70%; 95%]				
DAT = Digital pillbox				
Acosta et al	53	53	1.71 [0.52; 5.60]	1.1%
Jerene et al - Philippines	2236	4188	0.90 [0.53; 1.52]	3.5%
Jerene et al - South Africa	1281	1880	0.82 [0.57; 1.18]	4.8%
Jerene et al - Tanzania	1778	3656	0.79 [0.50; 1.25]	3.9%
Jerene et al - Ukraine	1526	1589	0.87 [0.63; 1.20]	5.1%
Liu et al (2015)	997	1104	1.41 [0.66; 3.01]	2.2%
Liu et al (2023)	1298	1388	0.97 [0.69; 1.36]	5.0%
Moulding & Caymittes	64	59	2.41 [0.77; 7.52]	1.2%
Ratchakit-Nedsuwan et al	50	50	0.46 [0.08; 2.67]	0.6%
Velen et al	124	126	1.11 [0.63; 1.97]	3.2%
Wei et al	143	135	1.29 [1.15; 1.44]	6.8%
Total (95% CI)			**1.03 [0.86; 1.23]**	**37.2%**
Heterogeneity: Tau^2 = 0.0317; Chi^2 = 18.02, df = 10 (P = 0.05); I^2 = 45% [0%; 72%]				
DAT = Smartphone app				
Haslinda & Juni	55	55	4.14 [1.15; 14.89]	1.0%
Iribarren et al	21	21	4.71 [0.48; 46.22]	0.3%
Zhou et al	112	112	1.53 [0.83; 2.81]	2.9%
Total (95% CI)			**2.17 [1.07; 4.40]**	**4.3%**
Heterogeneity: Tau^2 = 0.1012; Chi^2 = 2.51, df = 2 (P = 0.29); I^2 = 20% [0%; 92%]				
DAT = SMS				
Bediang et al	137	142	1.45 [0.82; 2.56]	3.2%
Fang et al	160	190	3.89 [1.55; 9.74]	1.7%
Farooqi et al	74	74	1.27 [0.33; 4.92]	0.9%
Gashu et al	152	154	1.49 [0.75; 2.95]	2.6%
Liu et al	1008	1104	2.27 [0.88; 5.86]	1.6%
Louwagie et al	283	291	0.86 [0.60; 1.24]	4.7%
Mohammed et al	1110	1097	1.02 [0.81; 1.28]	6.0%
Peng et al	234	229	1.10 [0.52; 2.34]	2.2%
Total (95% CI)			**1.28 [0.96; 1.70]**	**22.8%**
Heterogeneity: Tau^2 = 0.0680; Chi^2 = 13.43, df = 7 (P = 0.06); I^2 = 48% [0%; 77%]				
DAT = SMS+ Digital pillbox				
Liu et al	1064	1104	1.00 [0.45; 2.21]	2.1%
DAT = VOT				
Guo et al (b)	203	202	1.40 [0.55; 3.57]	1.6%
Ravenscroft et al	85	93	1.71 [0.55; 5.33]	1.2%
Story et al	112	114	1.53 [0.82; 2.85]	2.9%
Total (95% CI)			**1.53 [0.95; 2.44]**	**5.7%**
Heterogeneity: Tau^2 = 0; Chi^2 = 0.07, df = 2 (P = 0.97); I^2 = 0% [0%; 90%]				
**Total (95% CI)**			**1.14 [0.99; 1.30]**	**100.0%**
Heterogeneity: Tau^2 = 0.0679; Chi^2 = 76.68, df = 33 (P < 0.01); I^2 = 57% [37%; 71%]				
Test for subgroup differences: Chi^2 = 12.58, df = 6 (P = 0.05)				

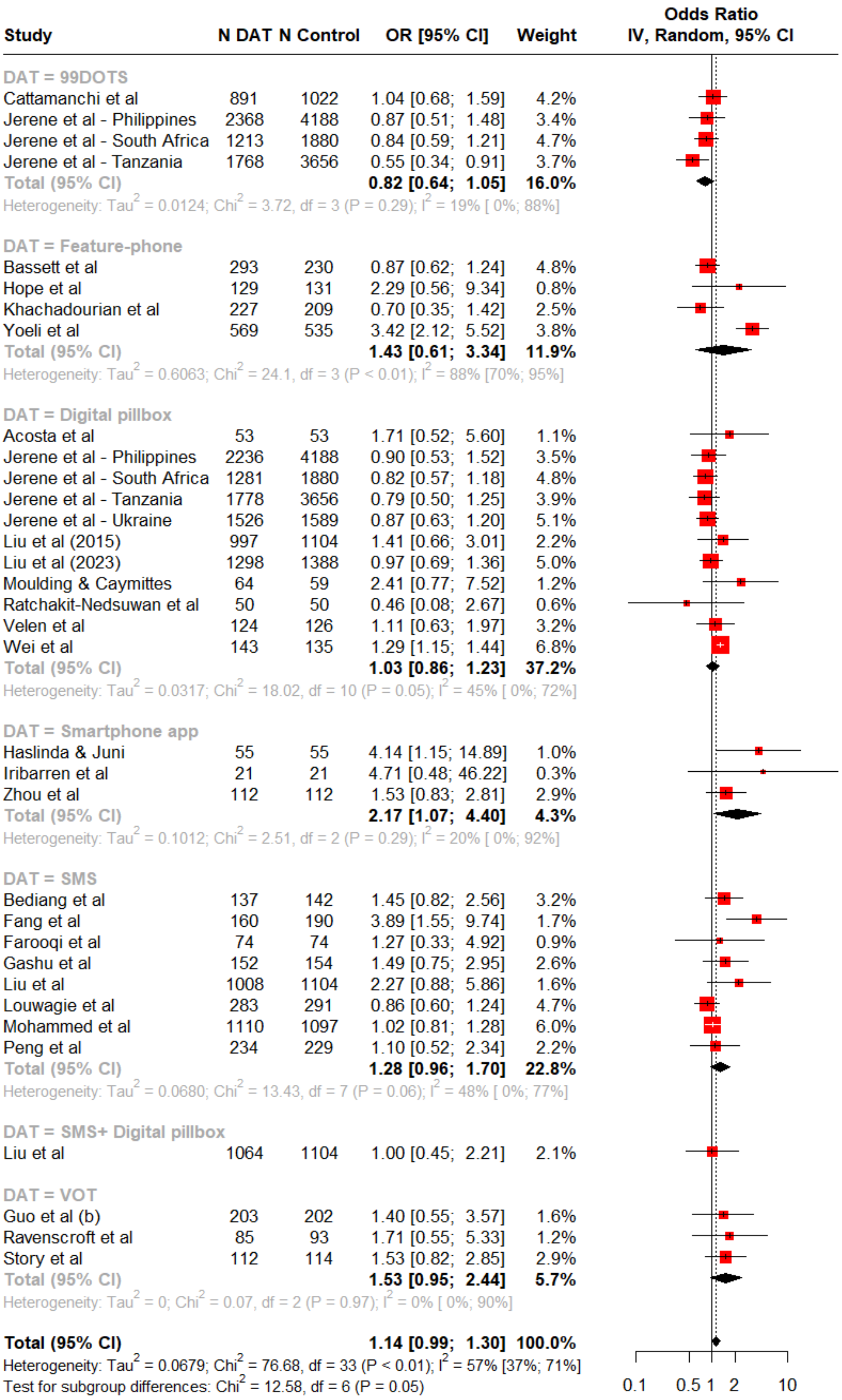

Figure S5b Forest plot showing treatment success in DAT groups compared to standard of care (RCTs)

Study	N DAT	N Control	OR [95% CI]	Weight
**DAT = 99DOTS**				
Chen et al.	8322	7722	0.97 [0.85; 1.10]	11.3%
Crowder et al	2051	1475	1.30 [1.06; 1.61]	10.1%
Thekkur et al	870	961	0.70 [0.57; 0.86]	10.1%
Wambi et al	309	132	1.65 [0.50; 5.47]	1.6%
Total (95% CI)			**0.99 [0.74; 1.32]**	**33.1%**
Heterogeneity: Tau^2 = 0.0616; Chi^2 = 18.03, df = 3 (P < 0.01); I^2 = 83% [58%; 93%]				
**DAT = Feature-phone**				
Das Gupta et al	111	111	0.35 [0.14; 0.87]	2.5%
Das Gupta et al	111	111	0.31 [0.12; 0.76]	2.6%
Hope et al	210	248	1.93 [1.08; 3.45]	4.8%
Sodhi et al	276	713	3.41 [1.70; 6.86]	3.8%
Total (95% CI)			**0.95 [0.31; 2.96]**	**13.6%**
Heterogeneity: Tau^2 = 1.1756; Chi^2 = 26.9, df = 3 (P < 0.01); I^2 = 89% [74%; 95%]				
**DAT = Digital pillbox**				
Kurada et al	385	3019	1.59 [0.89; 2.81]	4.9%
Park et al	206	141	4.33 [2.13; 8.81]	3.7%
Saha et al	200	200	0.69 [0.45; 1.04]	6.8%
Wang et al (2020)	1047	763	1.00 [0.98; 1.03]	12.1%
Wu et al	90	88	0.49 [0.12; 2.04]	1.2%
Total (95% CI)			**1.23 [0.75; 2.02]**	**28.6%**
Heterogeneity: Tau^2 = 0.2185; Chi^2 = 22.97, df = 4 (P < 0.01); I^2 = 83% [60%; 92%]				
**DAT = Smartphone app**				
Wu et al	82	88	0.69 [0.15; 3.17]	1.0%
Zhadnova et al	44	10	7.00 [1.32; 37.07]	0.9%
Zhang et al	1145	1576	1.12 [0.79; 1.58]	7.9%
Total (95% CI)			**1.51 [0.53; 4.30]**	**9.8%**
Heterogeneity: Tau^2 = 0.5203; Chi^2 = 4.95, df = 2 (P = 0.08); I^2 = 60% [0%; 88%]				
**DAT = SMS**				
Hermans et al	183	302	0.81 [0.54; 1.22]	6.9%
**DAT = VOT**				
Chuck et al	61	329	0.63 [0.13; 3.14]	1.0%
Perry et al	94	69	2.67 [0.73; 9.76]	1.4%
Salcedo et al	43	71	1.10 [0.34; 3.54]	1.7%
Wade et al	58	70	1.82 [0.85; 3.89]	3.4%
Yu et al	39	99	1.70 [0.22; 13.22]	0.6%
Total (95% CI)			**1.56 [0.93; 2.63]**	**8.0%**
Heterogeneity: Tau^2 = 0; Chi^2 = 2.38, df = 4 (P = 0.67); I^2 = 0% [0%; 79%]				
**Total (95% CI)**			**1.11 [0.94; 1.30]**	**100.0%**
Heterogeneity: Tau^2 = 0.0574; Chi^2 = 82.18, df = 21 (P < 0.01); I^2 = 74% [61%; 83%]				
Test for subgroup differences: Chi^2 = 4.86, df = 5 (P = 0.43)				

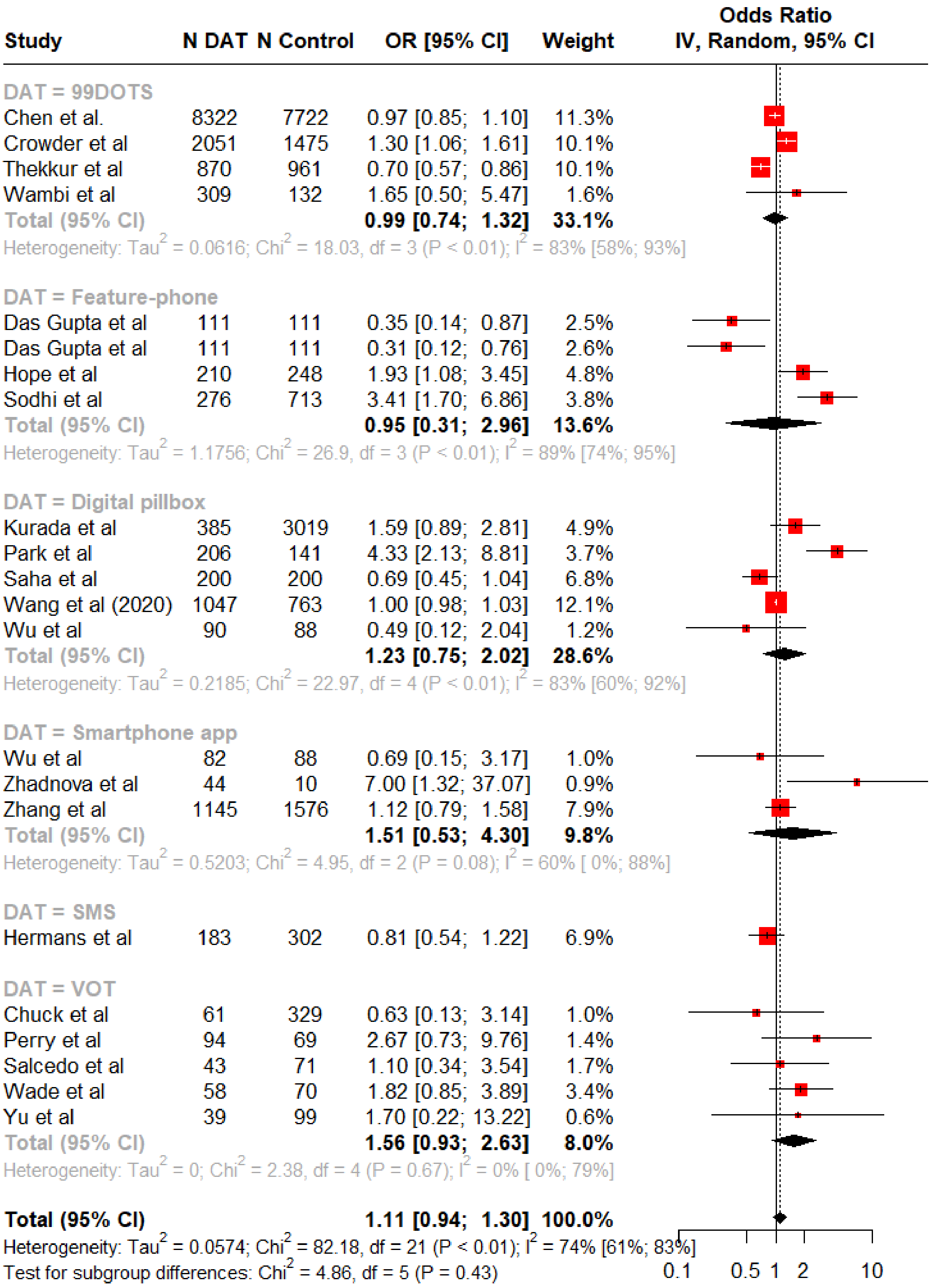

Figure S6c Forest plot showing treatment success in DAT groups compared to standard of care (observational studies)

Loss to follow up

Study	DAT	CIL	N DAT	N Control	OR [95% CI]	Weight
Study_Design = RCT						
Cattamanchi et al	99DOTS	Low	891	1022	1.11 [0.64; 1.93]	3.2%
Jerene et al - Philippines	99DOTS	Lower middle	2368	4188	1.02 [0.42; 2.51]	2.5%
Jerene et al - South Africa	99DOTS	Upper middle	1213	1880	1.09 [0.62; 1.90]	3.2%
Jerene et al - Tanzania	99DOTS	Lower middle	1768	3656	1.23 [0.53; 2.87]	2.6%
Manyazewal et al	Digital pillbox	Low	57	57	2.02 [0.07; 61.37]	0.4%
Velen et al	Digital pillbox	Lower middle	124	126	1.20 [0.39; 3.67]	2.1%
Jerene et al - Philippines	Digital pillbox	Lower middle	2236	4188	0.88 [0.37; 2.10]	2.6%
Jerene et al - Tanzania	Digital pillbox	Lower middle	1778	3656	0.65 [0.29; 1.47]	2.7%
Liu et al	Digital pillbox	Upper middle	997	1104	0.61 [0.25; 1.50]	2.5%
Ratchakit-Nedsuwan et al	Digital pillbox	Upper middle	50	50	1.03 [0.06; 16.98]	0.6%
Liu et al	Digital pillbox	Upper middle	1298	1388	0.92 [0.59; 1.44]	3.4%
Wei et al	Digital pillbox	Upper middle	143	135	0.25 [0.10; 0.65]	2.4%
Jerene et al - South Africa	Digital pillbox	Upper middle	1281	1880	1.25 [0.73; 2.14]	3.3%
Jerene et al - Ukraine	Digital pillbox	Lower middle	1526	1589	1.65 [0.68; 4.00]	2.6%
Yoeli et al	Feature-phone	Lower middle	569	535	0.16 [0.08; 0.32]	3.0%
Khachadourian et al	Feature-phone	Upper middle	227	209	1.51 [0.67; 3.40]	2.7%
Haslinda & Juni	Smartphone app	Upper middle	55	55	0.05 [0.00; 0.96]	0.6%
Zhou et al	Smartphone app	Upper middle	112	112	0.71 [0.27; 1.83]	2.4%
Iribarren et al	Smartphone app	Upper middle	21	21	0.23 [0.01; 5.47]	0.5%
Gashu et al	SMS	Low	152	154	2.05 [0.37; 11.38]	1.3%
Bediang et al	SMS	Lower middle	137	142	1.13 [0.67; 1.89]	3.3%
Gashu et al	SMS	Low	152	154	2.05 [0.37; 11.38]	1.3%
Louwagie et al	SMS	Upper middle	283	291	2.10 [1.10; 4.01]	3.0%
Mohammed et al	SMS	Lower middle	1110	1097	1.04 [0.78; 1.38]	3.7%
Liu et al	SMS	Upper middle	1008	1104	0.42 [0.18; 0.98]	2.6%
Fang et al	SMS	Upper middle	160	190	0.44 [0.26; 0.73]	3.3%
Farooqi et al	SMS	Lower middle	74	74	0.35 [0.09; 1.37]	1.7%
Liu et al	SMS+ Digital pillbox	Upper middle	1064	1104	0.90 [0.38; 2.11]	2.6%
Guo et al (b)	VOT	Upper middle	203	202	1.73 [0.40; 7.41]	1.6%
Story et al	VOT	High	112	114	0.25 [0.03; 2.21]	0.9%
Total (95% CI)					0.84 [0.66; 1.06]	68.9%
Prediction interval					[0.33; 2.15]	--
Heterogeneity: $Tau^2 = 0.1961$; $Chi^2 = 68.45$, df = 29 ($P < 0.01$); $I^2 = 58\%$ [36%; 72%]						
Study_Design = observational						
Crowder et al	99DOTS	Low	2051	1475	0.58 [0.44; 0.78]	3.7%
Thekkur et al	99DOTS	Lower middle	870	961	1.65 [1.15; 2.35]	3.6%
Chen et al	99DOTS	Lower middle	8322	7722	0.84 [0.66; 1.08]	3.7%
Park et al	Digital pillbox	Lower middle	206	141	0.03 [0.01; 0.07]	2.8%
Kurada et al	Digital pillbox	Lower middle	385	3019	0.15 [0.01; 2.39]	0.6%
Hope et al	Feature-phone	Low	210	248	0.45 [0.19; 1.05]	2.6%
Das Gupta et al	Feature-phone	Lower middle	111	111	4.78 [1.32; 17.26]	1.9%
Das Gupta et al	Feature-phone	Lower middle	111	111	5.20 [1.45; 18.63]	1.9%
Zhadnova et al	Smartphone app	Upper middle	44	10	0.10 [0.02; 0.50]	1.4%
Zhang et al	Smartphone app	Upper middle	1145	1576	0.46 [0.14; 1.51]	2.0%
Ali et al	SMS	Low	74	74	0.60 [0.22; 1.61]	2.4%
Hermans et al	SMS	Low	183	302	1.02 [0.41; 2.50]	2.5%
Chuck et al	VOT	High	61	329	2.76 [0.25; 31.05]	0.8%
Salcedo et al	VOT	High	43	71	1.11 [0.18; 6.90]	1.2%
Total (95% CI)					0.66 [0.38; 1.16]	31.1%
Prediction interval					[0.09; 5.05]	--
Heterogeneity: $Tau^2 = 0.7859$; $Chi^2 = 115.15$, df = 13 ($P < 0.01$); $I^2 = 89\%$ [83%; 93%]						
**Total (95% CI)**					**0.77 [0.61; 0.98]**	**100.0%**
**Prediction interval**					**[0.21; 2.81]**	
Heterogeneity: $Tau^2 = 0.3936$; $Chi^2 = 185.90$, df = 43 ($P < 0.01$); $I^2 = 77\%$ [69%; 83%]						
Test for subgroup differences: $Chi^2 = 0.58$, df = 1 ($P = 0.45$)						

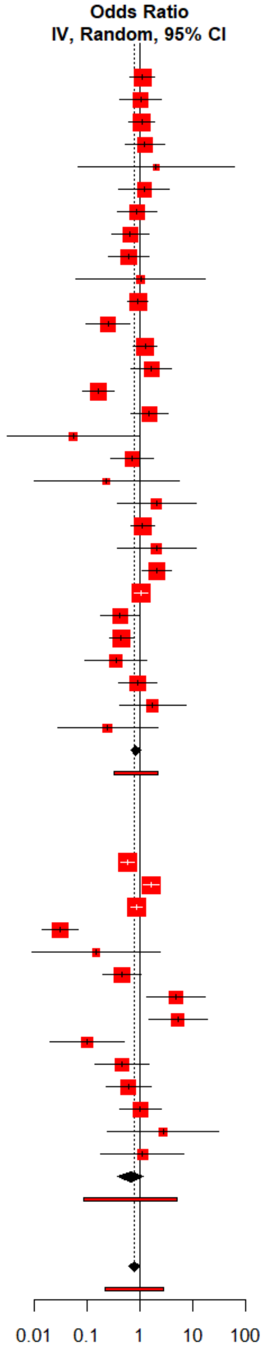

Figure S7a Forest plot showing loss to follow up in DAT groups compared to standard of care stratified by study design

Study	N DAT	N Control	OR [95% CI]	Weight
**DAT = 99DOTS**				
Cattamanchi et al	891	1022	1.11 [0.64; 1.93]	5.2%
Jerene et al - Philippines	2368	4188	1.02 [0.42; 2.51]	3.5%
Jerene et al - South Africa	1213	1880	1.09 [0.62; 1.90]	5.1%
Jerene et al - Tanzania	1768	3656	1.23 [0.53; 2.87]	3.7%
**Total (95% CI)**			**1.11 [0.80; 1.54]**	**17.5%**
Heterogeneity: $Tau^2 = 0$; $Chi^2 = 0.1$, df = 3 (P = 0.99); $I^2 = 0\%$ [0%; 85%]				
**DAT = Feature-phone**				
Yoeli et al	569	535	0.16 [0.08; 0.32]	4.5%
Khachadourian et al	227	209	1.51 [0.67; 3.40]	3.9%
**Total (95% CI)**			**0.49 [0.06; 4.35]**	**8.3%**
Heterogeneity: $Tau^2 = 2.3331$; $Chi^2 = 16.82$, df = 1 (P < 0.01); $I^2 = 94\%$ [81%; 98%]				
**DAT = Digital pillbox**				
Manyazewal et al	57	57	2.02 [0.07; 61.37]	0.4%
Velen et al	124	126	1.20 [0.39; 3.67]	2.7%
Jerene et al - Philippines	2236	4188	0.88 [0.37; 2.10]	3.6%
Jerene et al - Tanzania	1778	3656	0.65 [0.29; 1.47]	3.9%
Liu et al	997	1104	0.61 [0.25; 1.50]	3.5%
Ratchakit-Nedsuwan et al	50	50	1.03 [0.06; 16.98]	0.6%
Liu et al	1298	1388	0.92 [0.59; 1.44]	5.7%
Wei et al	143	135	0.25 [0.10; 0.65]	3.3%
Jerene et al - South Africa	1281	1880	1.25 [0.73; 2.14]	5.2%
Jerene et al - Ukraine	1526	1589	1.65 [0.68; 4.00]	3.5%
**Total (95% CI)**			**0.87 [0.64; 1.19]**	**32.5%**
Heterogeneity: $Tau^2 = 0.0585$; $Chi^2 = 11.93$, df = 9 (P = 0.22); $I^2 = 25\%$ [0%; 63%]				
**DAT = Smartphone app**				
Haslinda & Juni	55	55	0.05 [0.00; 0.96]	0.6%
Zhou et al	112	112	0.71 [0.27; 1.83]	3.3%
Iribarren et al	21	21	0.23 [0.01; 5.47]	0.5%
**Total (95% CI)**			**0.35 [0.08; 1.53]**	**4.4%**
Heterogeneity: $Tau^2 = 0.6903$; $Chi^2 = 3.02$, df = 2 (P = 0.22); $I^2 = 34\%$ [0%; 78%]				
**DAT = SMS**				
Gashu et al	152	154	2.05 [0.37; 11.38]	1.5%
Bediang et al	137	142	1.13 [0.67; 1.89]	5.4%
Gashu et al	152	154	2.05 [0.37; 11.38]	1.5%
Louwagie et al	283	291	2.10 [1.10; 4.01]	4.7%
Mohammed et al	1110	1097	1.04 [0.78; 1.38]	6.5%
Liu et al	1008	1104	0.42 [0.18; 0.98]	3.7%
Fang et al	160	190	0.44 [0.26; 0.73]	5.4%
Farooqi et al	74	74	0.35 [0.09; 1.37]	2.1%
**Total (95% CI)**			**0.90 [0.57; 1.40]**	**30.7%**
Heterogeneity: $Tau^2 = 0.2347$; $Chi^2 = 22.7$, df = 7 (P < 0.01); $I^2 = 69\%$ [36%; 85%]				
**DAT = SMS+ Digital pillbox**				
Liu et al	1064	1104	0.90 [0.38; 2.11]	3.7%
**DAT = VOT**				
Guo et al (b)	203	202	1.73 [0.40; 7.41]	1.9%
Story et al	112	114	0.25 [0.03; 2.21]	1.0%
**Total (95% CI)**			**0.78 [0.12; 5.12]**	**2.9%**
Heterogeneity: $Tau^2 = 1.0029$; $Chi^2 = 2.11$, df = 1 (P = 0.15); $I^2 = 53\%$ [0%; 88%]				
**Total (95% CI)**			**0.84 [0.66; 1.06]**	**100.0%**

Heterogeneity: $Tau^2 = 0.1961$; $Chi^2 = 68.45$, df = 29 (P < 0.01); $I^2 = 58\%$ [36%; 72%]

Test for subgroup differences: $Chi^2 = 3.33$, df = 6 (P = 0.77)

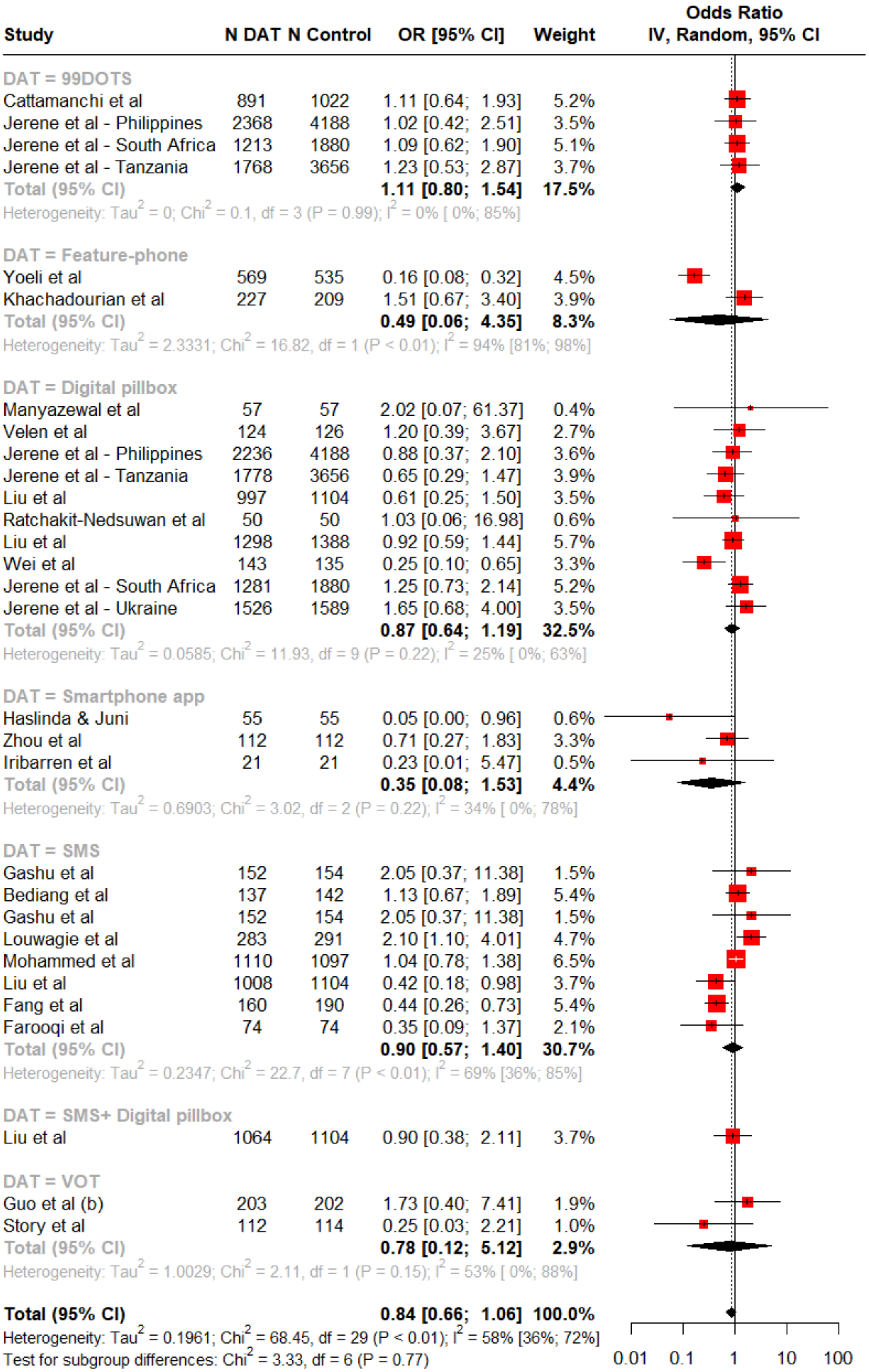

Figure S8b Forest plot showing loss to follow up in DAT groups compared to standard of care (RCTs)

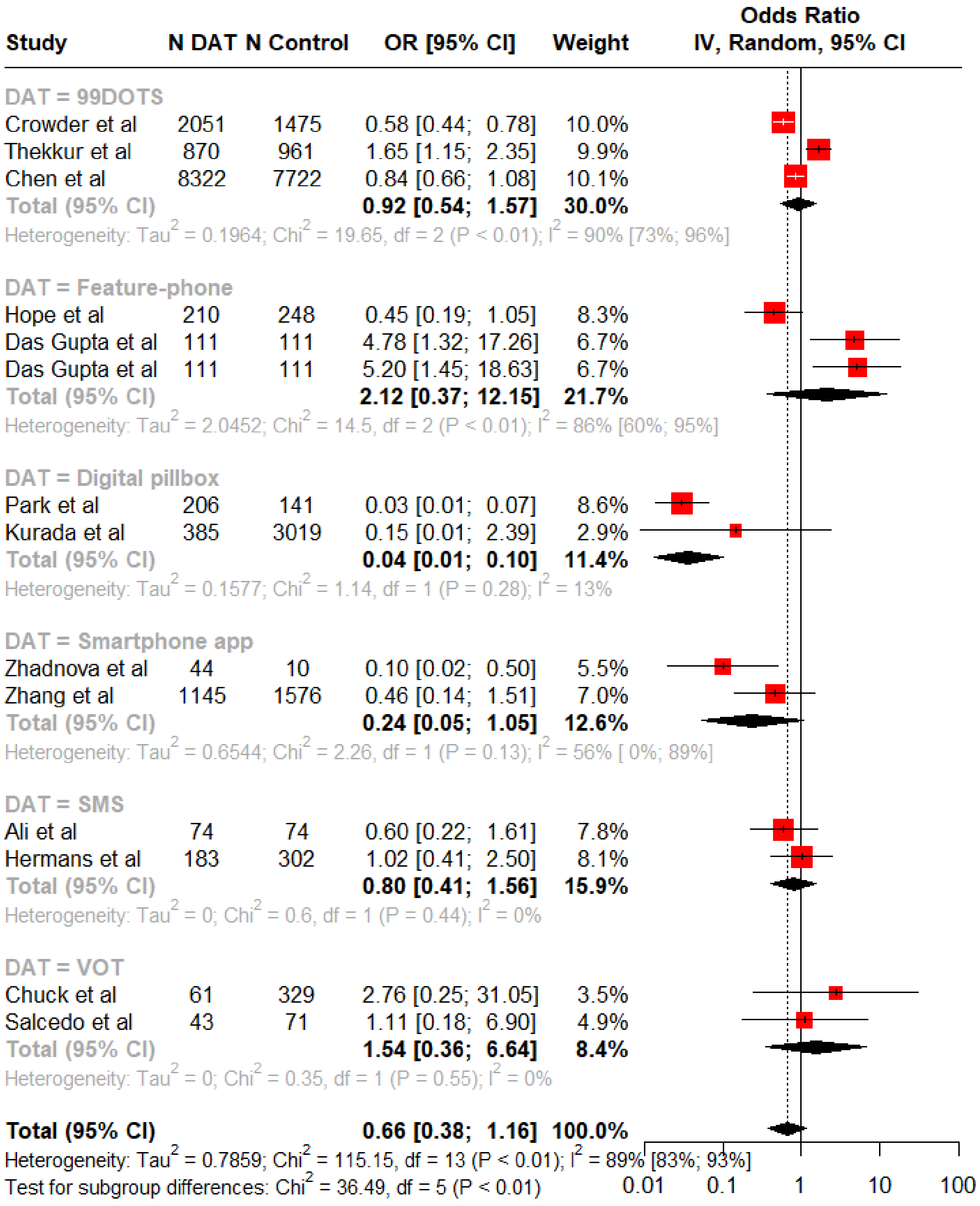

Figure S9c Forest plot showing loss to follow up in DAT groups compared to standard of care (observational studies)

Treatment failure

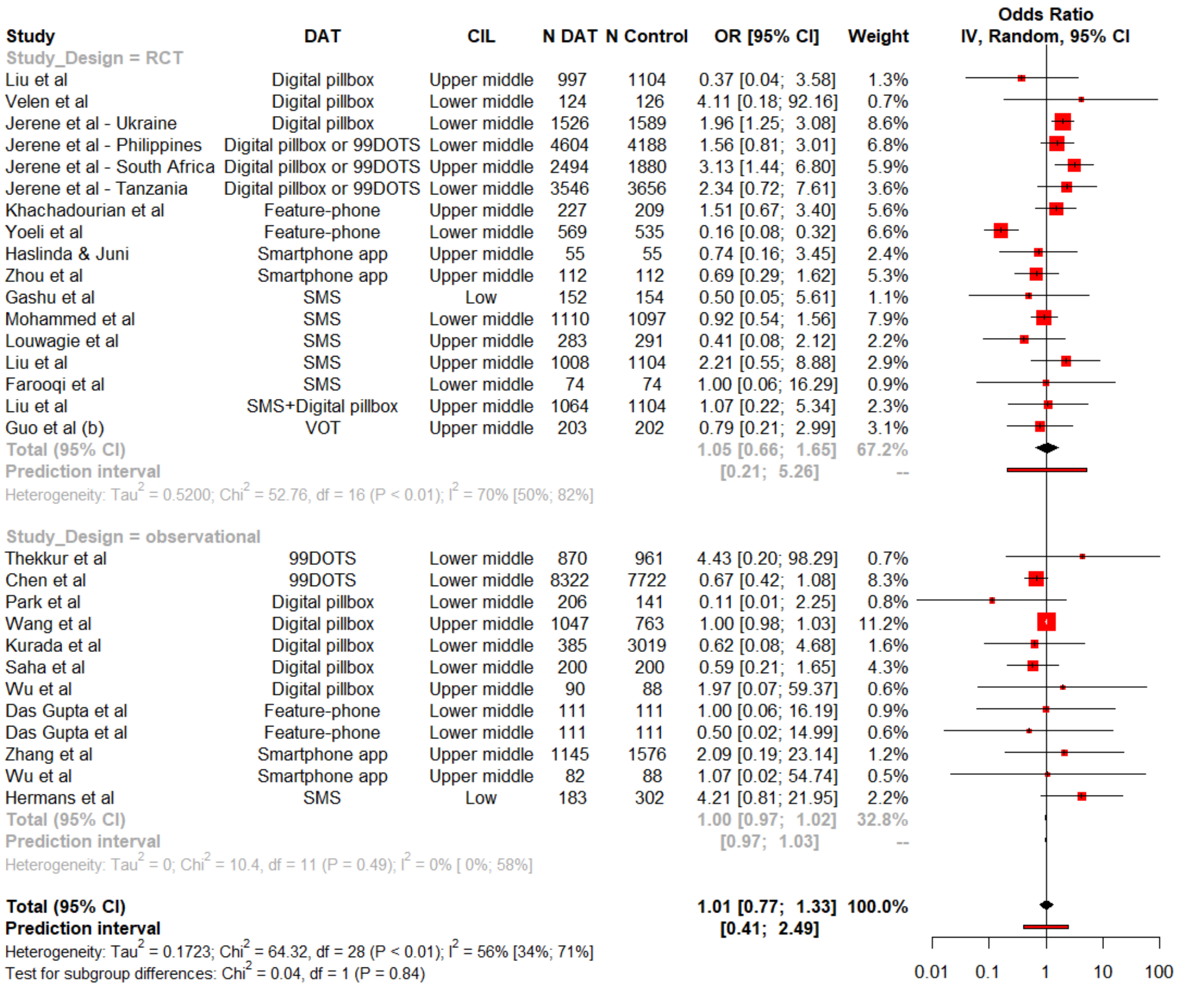

Figure S10a Forest plot showing treatment failure in DAT groups compared to standard of care stratified by study design

Study	N DAT	N Control	OR [95% CI]	Weight
DAT = SMS				
Gashu et al	152	154	0.50 [0.05; 5.61]	2.7%
Mohammed et al	1110	1097	0.92 [0.54; 1.56]	9.2%
Louwagie et al	283	291	0.41 [0.08; 2.12]	4.4%
Liu et al	1008	1104	2.21 [0.55; 8.88]	5.3%
Farooqi et al	74	74	1.00 [0.06; 16.29]	2.1%
Total (95% CI)			**0.93 [0.59; 1.48]**	**23.8%**
Heterogeneity: Tau^2 = 0; Chi^2 = 2.72, df = 4 (P = 0.61); I^2 = 0% [0%; 79%]				
DAT = Feature-phone				
Khachadourian et al	227	209	1.51 [0.67; 3.40]	7.9%
Yoeli et al	569	535	0.16 [0.08; 0.32]	8.5%
Total (95% CI)			**0.49 [0.06; 4.35]**	**16.4%**
Heterogeneity: Tau^2 = 2.3331; Chi^2 = 16.82, df = 1 (P < 0.01); I^2 = 94% [81%; 98%]				
DAT = Digital pillbox				
Liu et al	997	1104	0.37 [0.04; 3.58]	2.9%
Velen et al	124	126	4.11 [0.18; 92.16]	1.8%
Jerene et al - Ukraine	1526	1589	1.96 [1.25; 3.08]	9.5%
Total (95% CI)			**1.74 [0.83; 3.63]**	**14.3%**
Heterogeneity: Tau^2 = 0.1133; Chi^2 = 2.25, df = 2 (P = 0.32); I^2 = 11% [0%; 91%]				
DAT = Digital pillbox or 99DOTS				
Jerene et al - Philippines	4604	4188	1.56 [0.81; 3.01]	8.6%
Jerene et al - South Africa	2494	1880	3.13 [1.44; 6.80]	8.1%
Jerene et al - Tanzania	3546	3656	2.34 [0.72; 7.61]	6.2%
Total (95% CI)			**2.12 [1.34; 3.37]**	**22.9%**
Heterogeneity: Tau^2 = 0; Chi^2 = 1.84, df = 2 (P = 0.40); I^2 = 0% [0%; 90%]				
DAT = Smartphone app				
Haslinda & Juni	55	55	0.74 [0.16; 3.45]	4.8%
Zhou et al	112	112	0.69 [0.29; 1.62]	7.7%
Total (95% CI)			**0.70 [0.33; 1.48]**	**12.5%**
Heterogeneity: Tau^2 = 0; Chi^2 = 0.01, df = 1 (P = 0.94); I^2 = 0%				
DAT = SMS+Digital pillbox				
Liu et al	1064	1104	1.07 [0.22; 5.34]	4.6%
DAT = VOT				
Guo et al (b)	203	202	0.79 [0.21; 2.99]	5.6%
**Total (95% CI)**			**1.05 [0.66; 1.65]**	**100.0%**

Heterogeneity: Tau^2 = 0.5200; Chi^2 = 52.76, df = 16 (P < 0.01); I^2 = 70% [50%; 82%]
Test for subgroup differences: Chi^2 = 10.93, df = 6 (P = 0.09)

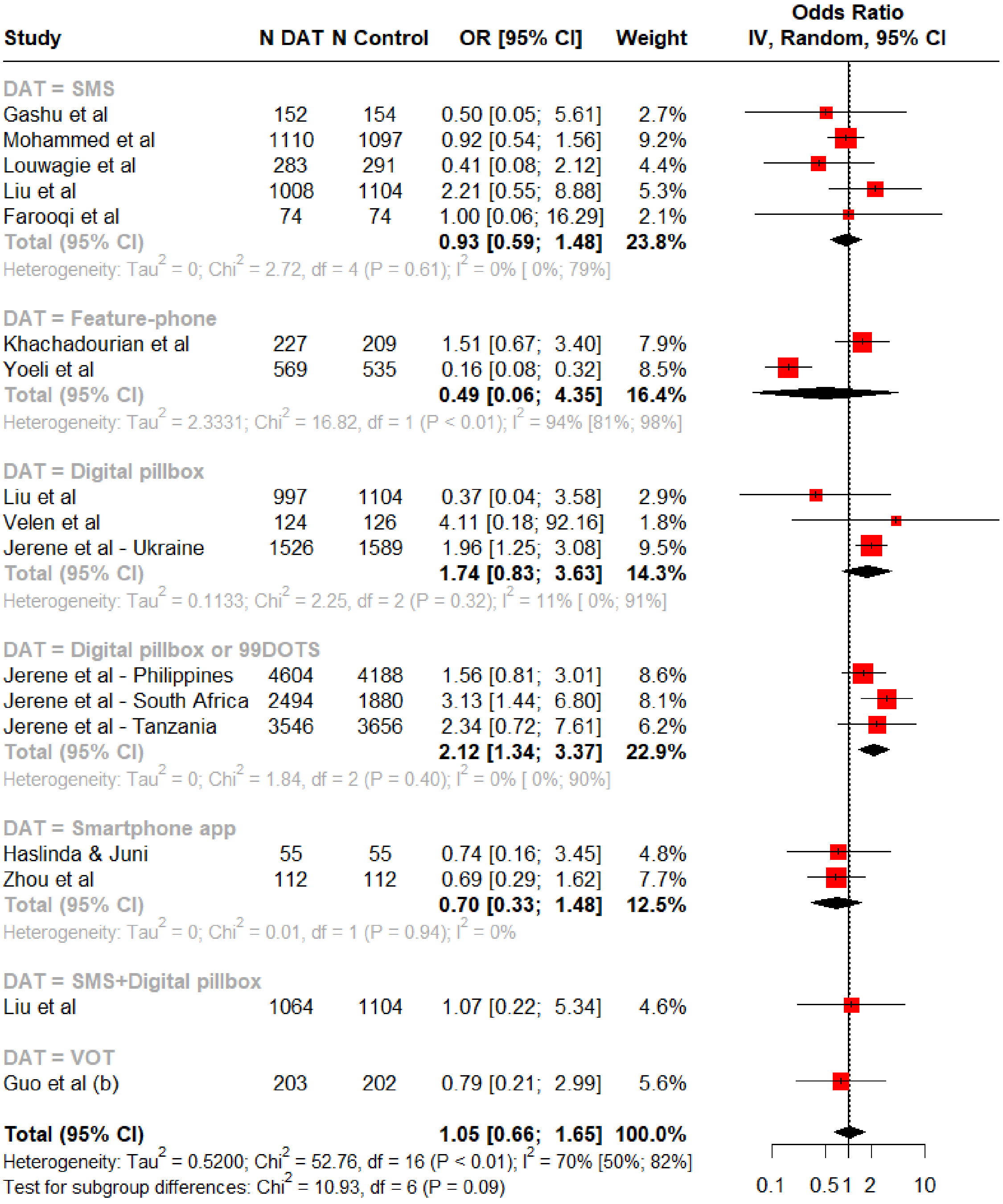

Figure S11b Forest plot showing treatment failure in DAT groups compared to standard of care (RCTs)

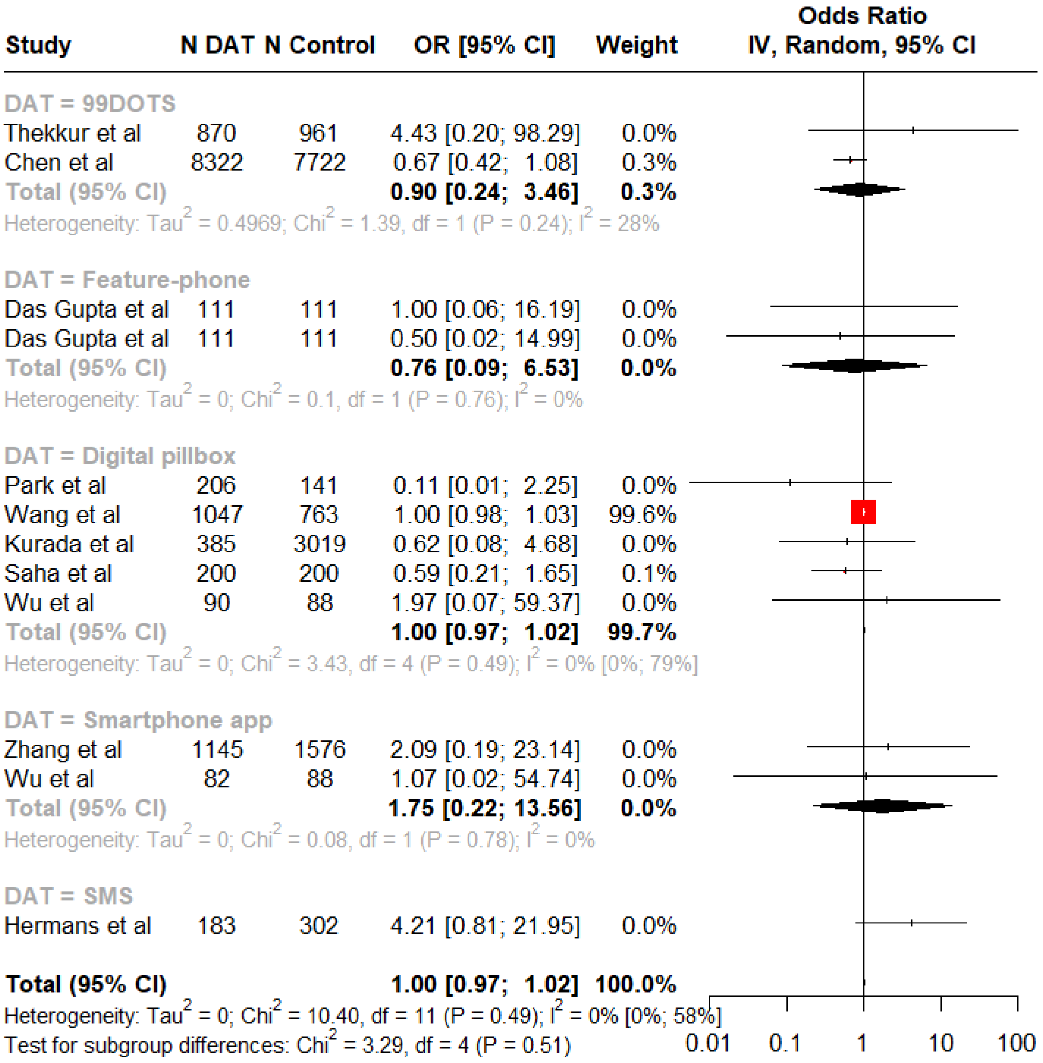

Figure S12c Forest plot showing treatment failure in DAT groups compared to standard of care (observational studies)

Death

Study	DAT	CIL	N DAT	N Control	OR [95% CI]	Weight
Study_Design = RCT						
Jerene et al - Philippines	99DOTS	Lower middle	2368	4188	1.74 [1.03; 2.94]	3.2%
Jerene et al - South Africa	99DOTS	Upper middle	1213	1880	1.05 [0.66; 1.68]	4.0%
Jerene et al - Tanzania	99DOTS	Lower middle	1768	3656	1.99 [1.29; 3.07]	4.7%
Manyazewal et al	Digital pillbox	Low	57	57	1.00 [0.02; 51.28]	0.1%
Liu et al	Digital pillbox	Upper middle	997	1104	1.51 [0.52; 4.36]	0.8%
Ratchakit-Nedsuwan et al	Digital pillbox	Upper middle	50	50	3.24 [0.32; 32.57]	0.2%
Velen et al	Digital pillbox	Lower middle	124	126	6.36 [0.75; 53.59]	0.2%
Jerene et al - Philippines	Digital pillbox	Lower middle	2236	4188	1.57 [0.92; 2.69]	3.1%
Jerene et al - South Africa	Digital pillbox	Upper middle	1281	1880	1.00 [0.63; 1.58]	4.1%
Jerene et al - Tanzania	Digital pillbox	Lower middle	1778	3656	1.57 [1.06; 2.33]	5.5%
Jerene et al - Ukraine	Digital pillbox	Lower middle	1526	1589	1.26 [0.99; 1.60]	13.2%
Khachadourian et al	Feature-phone	Upper middle	227	209	0.92 [0.18; 4.61]	0.4%
Yoeli et al	Feature-phone	Lower middle	569	535	0.56 [0.24; 1.28]	1.3%
Haslinda & Juni	Smartphone app	Upper middle	55	55	1.73 [0.39; 7.64]	0.4%
Zhou et al	Smartphone app	Upper middle	112	112	0.74 [0.25; 2.19]	0.8%
Iribarren et al	Smartphone app	Upper middle	21	21	0.49 [0.02; 15.37]	0.1%
Gashu et al	SMS	Low	152	154	0.50 [0.05; 5.61]	0.2%
Bediang et al	SMS	Lower middle	137	142	0.34 [0.07; 1.69]	0.4%
Louwagie et al	SMS	Upper middle	283	291	1.42 [0.64; 3.16]	1.4%
Mohammed et al	SMS	Lower middle	1110	1097	2.02 [1.15; 3.52]	2.9%
Liu et al	SMS	Upper middle	1008	1104	1.12 [0.36; 3.48]	0.7%
Liu et al	SMS+Digital pillbox	Upper middle	1064	1104	1.27 [0.42; 3.79]	0.8%
Story et al	VOT	High	112	114	1.02 [0.02; 51.75]	0.1%
Guo et al (b)	VOT	Upper middle	203	202	0.67 [0.01; 33.71]	0.1%
Total (95% CI)					1.34 [1.18; 1.53]	48.5%
Prediction interval					[1.17; 1.55]	--
Heterogeneity: Tau^2 = 0; Chi^2 = 22.56, df = 23 (P = 0.49); I^2 = 0% [0%; 45%]						
Study_Design = observational						
Thekkur et al	99DOTS	Lower middle	870	961	1.33 [1.05; 1.69]	13.2%
Chen et al	99DOTS	Lower middle	8322	7722	1.22 [1.04; 1.42]	25.8%
Park et al	Digital pillbox	Lower middle	206	141	0.34 [0.03; 3.78]	0.2%
Wang et al	Digital pillbox	Upper middle	1047	763	0.74 [0.44; 1.25]	3.2%
Kurada et al	Digital pillbox	Lower middle	385	3019	0.64 [0.30; 1.39]	1.5%
Saha et al	Digital pillbox	Lower middle	200	200	0.66 [0.23; 1.88]	0.8%
Wu et al	Digital pillbox	Upper middle	90	88	1.98 [0.18; 22.21]	0.2%
Das Gupta et al	Feature-phone	Lower middle	111	111	6.14 [0.30; 124.00]	0.1%
Das Gupta et al	Feature-phone	Lower middle	111	111	6.14 [0.30; 124.00]	0.1%
Zhang et al	Smartphone app	Upper middle	1145	1576	1.50 [0.83; 2.72]	2.5%
Wu et al	Smartphone app	Upper middle	82	88	1.07 [0.07; 17.46]	0.1%
Hermans et al	SMS	Low	183	302	0.94 [0.56; 1.57]	3.4%
Chuck et al	VOT	High	61	329	1.79 [0.17; 18.80]	0.2%
Perry et al	VOT	High	94	69	0.48 [0.08; 2.94]	0.3%
Total (95% CI)					1.16 [1.01; 1.33]	51.5%
Prediction interval					[0.94; 1.43]	--
Heterogeneity: Tau^2 = 0.0043; Chi^2 = 13.73, df = 13 (P = 0.39); I^2 = 5% [0%; 57%]						
**Total (95% CI)**					**1.25 [1.13; 1.38]**	**100.0%**
**Prediction interval**					**[1.07; 1.46]**	
Heterogeneity: Tau^2 = 0.0032; Chi^2 = 38.38, df = 37 (P = 0.41); I^2 = 4% [0%; 31%]						
Test for subgroup differences: Chi^2 = 2.25, df = 1 (P = 0.13)						

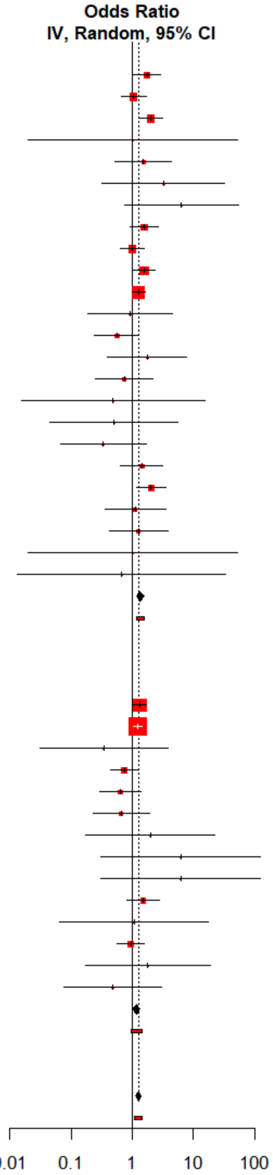

Figure S13a Forest plot showing death during treatment in DAT groups compared to standard of care stratified by study design

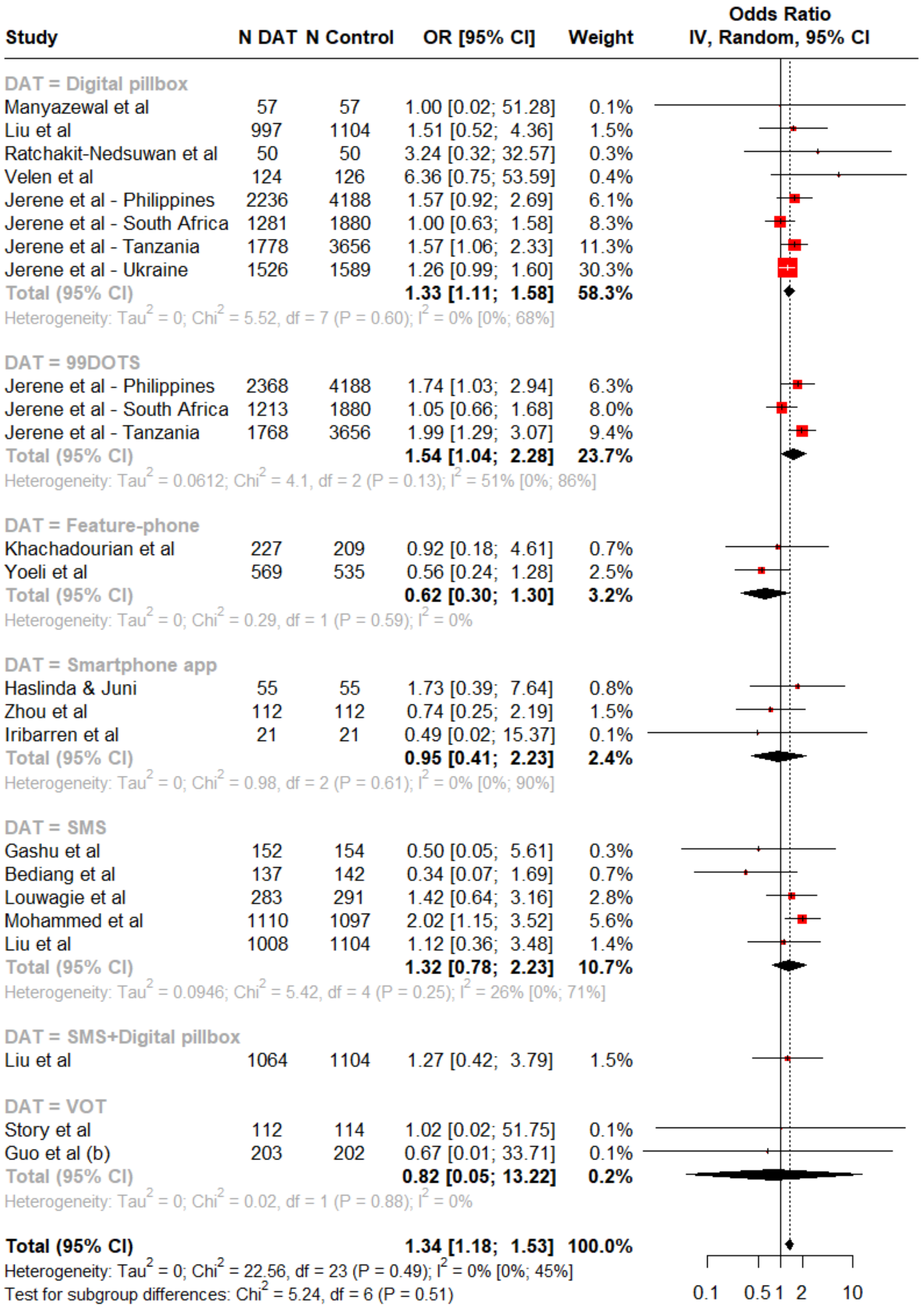

Figure S14b Forest plot showing death during treatment in DAT groups compared to standard of care (RCTs)

Study	N DAT	N Control	OR [95% CI]	Weight
**DAT = 99DOTS**				
Thekkur et al	870	961	1.33 [1.05; 1.69]	26.0%
Chen et al	8322	7722	1.22 [1.04; 1.42]	48.3%
**Total (95% CI)**			**1.25 [1.10; 1.42]**	**74.3%**
Heterogeneity: $Tau^2 = 0$; $Chi^2 = 0.37$, df = 1 (P = 0.54); $I^2 = 0\%$				
**DAT = Feature-phone**				
Das Gupta et al	111	111	6.14 [0.30; 124.00]	0.2%
Das Gupta et al	111	111	6.14 [0.30; 124.00]	0.2%
**Total (95% CI)**			**6.14 [0.73; 51.42]**	**0.4%**
Heterogeneity: $Tau^2 = 0$; $Chi^2 = 0$, df = 1 (P = 1.00); $I^2 = 0\%$				
**DAT = Digital pillbox**				
Park et al	206	141	0.34 [0.03; 3.78]	0.3%
Wang et al	1047	763	0.74 [0.44; 1.25]	6.6%
Kurada et al	385	3019	0.64 [0.30; 1.39]	3.1%
Saha et al	200	200	0.66 [0.23; 1.88]	1.7%
Wu et al	90	88	1.98 [0.18; 22.21]	0.3%
**Total (95% CI)**			**0.70 [0.48; 1.04]**	**12.1%**
Heterogeneity: $Tau^2 = 0$; $Chi^2 = 1.16$, df = 4 (P = 0.88); $I^2 = 0\%$ [0%; 79%]				
**DAT = Smartphone app**				
Zhang et al	1145	1576	1.50 [0.83; 2.72]	5.2%
Wu et al	82	88	1.07 [0.07; 17.46]	0.2%
**Total (95% CI)**			**1.48 [0.83; 2.64]**	**5.5%**
Heterogeneity: $Tau^2 = 0$; $Chi^2 = 0.05$, df = 1 (P = 0.82); $I^2 = 0\%$				
**DAT = SMS**				
Hermans et al	183	302	0.94 [0.56; 1.57]	6.9%
**DAT = VOT**				
Chuck et al	61	329	1.79 [0.17; 18.80]	0.3%
Perry et al	94	69	0.48 [0.08; 2.94]	0.6%
**Total (95% CI)**			**0.78 [0.19; 3.30]**	**0.9%**
Heterogeneity: $Tau^2 = 0$; $Chi^2 = 0.76$, df = 1 (P = 0.38); $I^2 = 0\%$				
**Total (95% CI)**			**1.16 [1.01; 1.33]**	**100.0%**
Heterogeneity: $Tau^2 = 0.0043$; $Chi^2 = 13.73$, df = 13 (P = 0.39); $I^2 = 5\%$ [0%; 57%]				
Test for subgroup differences: $Chi^2 = 11.39$, df = 5 (P = 0.04)				

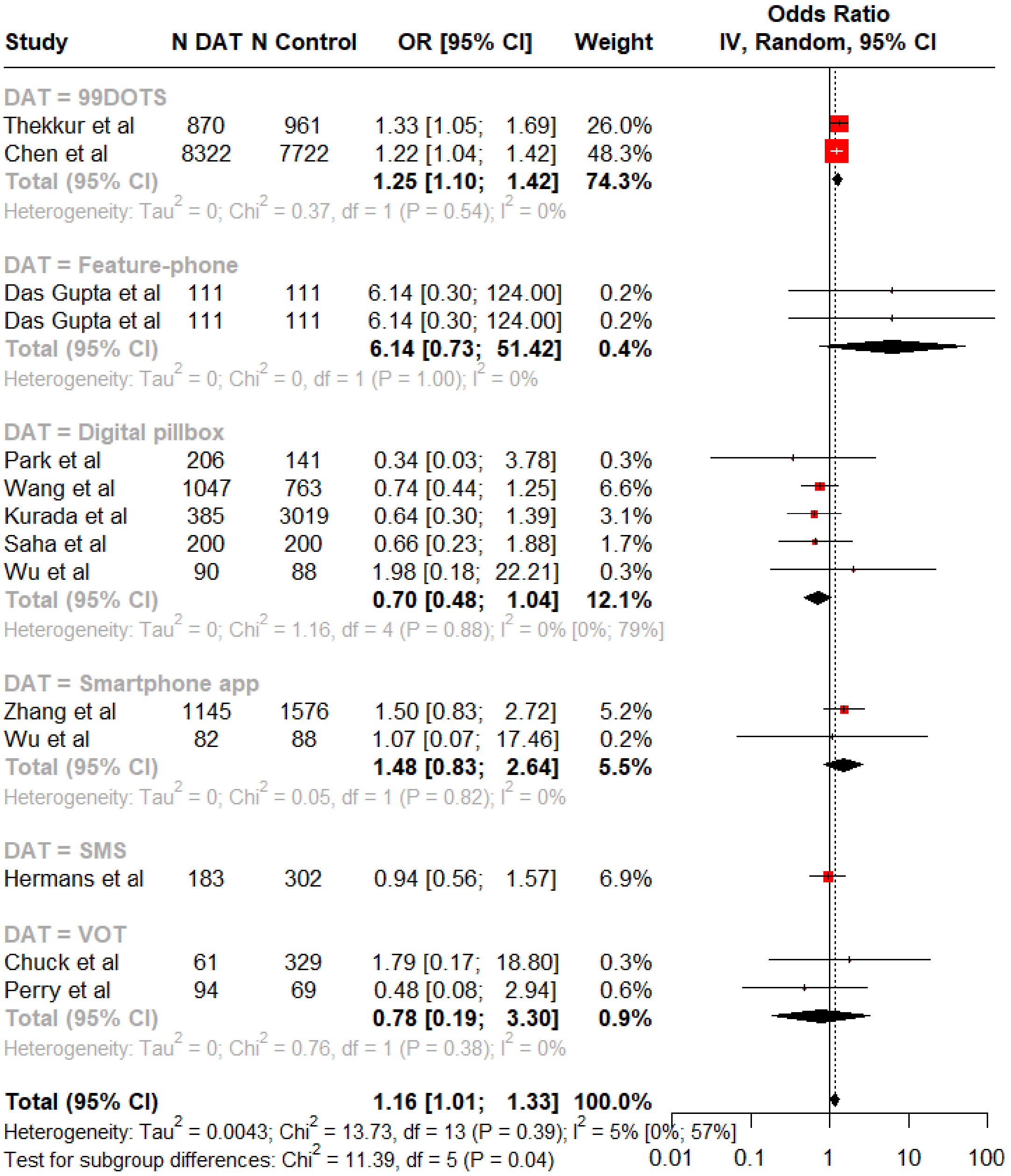

Figure S15c Forest plot showing death during treatment in DAT groups compared to standard of care (observational studies)

Adverse events

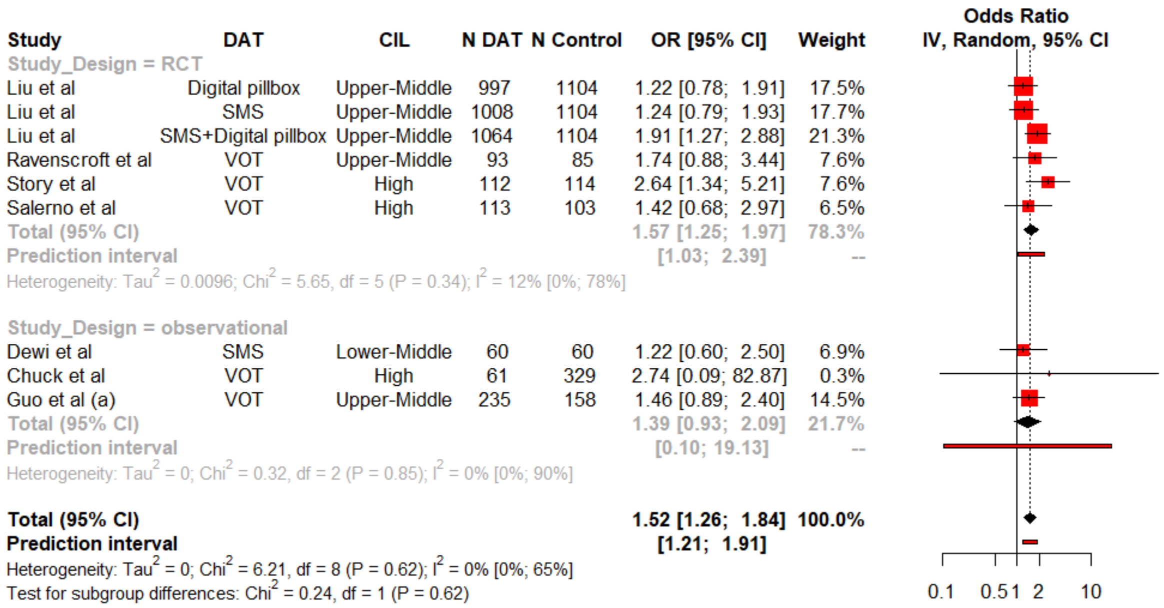

Figure S16a Forest plot showing adverse events reported in DAT groups compared to standard of care stratified by study design

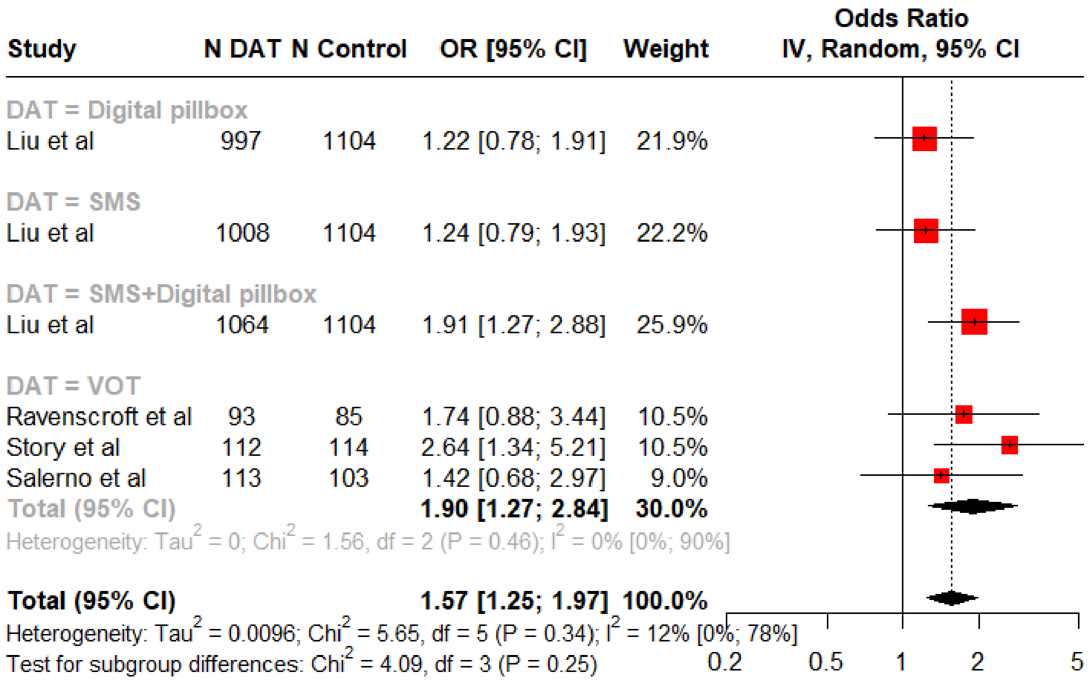

Figure S17b Forest plot showing adverse events reported in DAT groups compared to standard of care (RCTs)

Study	N DAT	N Control	OR [95% CI]	Weight
DAT = SMS				
Dewi et al	60	60	1.22 [0.60; 2.50]	31.7%
DAT = VOT				
Chuck et al	61	329	2.74 [0.09; 82.87]	1.4%
Guo et al (a)	235	158	1.46 [0.89; 2.40]	66.9%
Total (95% CI)			**1.48 [0.91; 2.42]**	**68.3%**
Heterogeneity: Tau^2 = 0; Chi^2 = 0.13, df = 1 (P = 0.72); I^2 = 0%				
**Total (95% CI)**			**1.39 [0.93; 2.09]**	**100.0%**
Heterogeneity: Tau^2 = 0; Chi^2 = 0.32, df = 2 (P = 0.85); I^2 = 0% [0%; 90%]				
Test for subgroup differences: Chi^2 = 0.19, df = 1 (P = 0.66)				

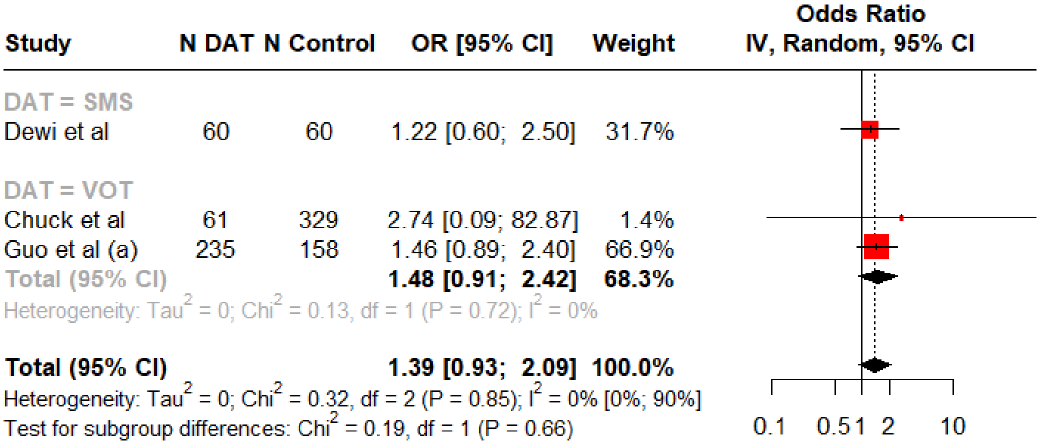

Figure S18c Forest plot showing adverse events reported in DAT groups compared to standard of care (observational studies)

Cure

Study	DAT	CIL	N DAT	N Control	OR [95% CI]	Weight
Study_Design = RCT						
Velen et al	Digital pillbox	Lower middle	124	126	0.54 [0.27; 1.10]	3.7%
Khachadourian et al	Feature-phone	Upper middle	227	209	1.37 [0.78; 2.39]	4.6%
Yoeli et al	Feature-phone	Lower middle	569	535	1.29 [1.02; 1.64]	6.7%
Iribarren et al	Smartphone app	Upper middle	21	21	20.50 [1.07; 391.07]	0.4%
Gashu et al	SMS	Low	152	154	1.50 [0.84; 2.69]	4.5%
Bediang et al	SMS	Lower middle	137	142	1.07 [0.66; 1.74]	5.1%
Louwagie et al	SMS	Upper middle	283	291	1.16 [0.78; 1.73]	5.7%
Mohammed et al	SMS	Lower middle	1110	1097	1.00 [0.85; 1.19]	7.0%
Farooqi et al	SMS	Lower middle	74	74	1.07 [0.52; 2.20]	3.7%
Guo et al (b)	VOT	Upper middle	203	202	1.55 [0.81; 2.96]	4.1%
Total (95% CI)					1.15 [0.97; 1.36]	45.4%
Prediction interval					[0.78; 1.69]	--
Heterogeneity: Tau^2 = 0.0203; Chi^2 = 13.23, df = 9 (P = 0.15); I^2 = 32% [0%; 68%]						
Study_Design = observational						
Thekkur et al	99DOTS	Lower middle	870	961	0.28 [0.22; 0.36]	6.6%
Chen et al	99DOTS	Lower middle	8322	7722	0.92 [0.86; 0.98]	7.3%
Wang et al	Digital pillbox	Upper middle	1047	763	1.07 [0.89; 1.29]	6.9%
Park et al	Digital pillbox	Lower middle	206	141	2.15 [1.39; 3.33]	5.4%
Broomhead and Mars	Digital pillbox	Upper middle	24	96	6.29 [2.27; 17.41]	2.5%
Kurada et al	Digital pillbox	Lower middle	385	3019	0.81 [0.62; 1.04]	6.6%
Wu et al	Digital pillbox	Upper middle	90	88	2.42 [1.25; 4.68]	4.0%
Zhadnova et al	Smartphone app	Upper middle	44	10	6.35 [1.20; 33.55]	1.2%
Zhang et al	Smartphone app	Upper middle	1145	1576	0.86 [0.72; 1.03]	7.0%
Wu et al	Smartphone app	Upper middle	82	88	0.88 [0.42; 1.86]	3.6%
Ali et al	SMS	Low	74	74	2.47 [1.20; 5.09]	3.7%
Total (95% CI)					1.17 [0.86; 1.59]	54.6%
Prediction interval					[0.41; 3.38]	--
Heterogeneity: Tau^2 = 0.1950; Chi^2 = 137.32, df = 10 (P < 0.01); I^2 = 93% [89%; 95%]						
**Total (95% CI)**					**1.14 [0.94; 1.39]**	**100.0%**
**Prediction interval**					**[0.52; 2.53]**	
Heterogeneity: Tau^2 = 0.1337; Chi^2 = 161.48, df = 20 (P < 0.01); I^2 = 88% [82%; 91%]						
Test for subgroup differences: Chi^2 = 0.01, df = 1 (P = 0.91)						

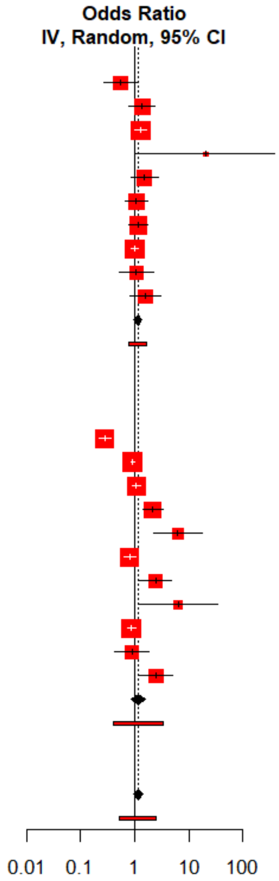

Figure S19a Forest plot showing cure rate in DAT groups compared to standard of care stratified by study design

Study	N DAT	N Control	OR [95% CI]	Weight
DAT = SMS				
Gashu et al	152	154	1.50 [0.84; 2.69]	6.9%
Bediang et al	137	142	1.07 [0.66; 1.74]	9.2%
Louwagie et al	283	291	1.16 [0.78; 1.73]	12.3%
Mohammed et al	1110	1097	1.00 [0.85; 1.19]	27.2%
Farooqi et al	74	74	1.07 [0.52; 2.20]	4.8%
Total (95% CI)			**1.05 [0.92; 1.21]**	**60.4%**
Heterogeneity: $Tau^2 = 0$; $Chi^2 = 1.99$, df = 4 (P = 0.74); $I^2 = 0\%$ [0%; 79%]				
DAT = Feature-phone				
Khachadourian et al	227	209	1.37 [0.78; 2.39]	7.3%
Yoeli et al	569	535	1.29 [1.02; 1.64]	21.3%
Total (95% CI)			**1.30 [1.05; 1.62]**	**28.6%**
Heterogeneity: $Tau^2 = 0$; $Chi^2 = 0.03$, df = 1 (P = 0.86); $I^2 = 0\%$				
DAT = Digital pillbox				
Velen et al	124	126	0.54 [0.27; 1.10]	4.9%
DAT = Smartphone app				
Iribarren et al	21	21	20.50 [1.07; 391.07]	0.3%
DAT = VOT				
Guo et al (b)	203	202	1.55 [0.81; 2.96]	5.8%
**Total (95% CI)**			**1.15 [0.97; 1.36]**	**100.0%**
Heterogeneity: $Tau^2 = 0.0203$; $Chi^2 = 13.23$, df = 9 (P = 0.15); $I^2 = 32\%$ [0%; 68%]				
Test for subgroup differences: $Chi^2 = 11.21$, df = 4 (P = 0.02)				

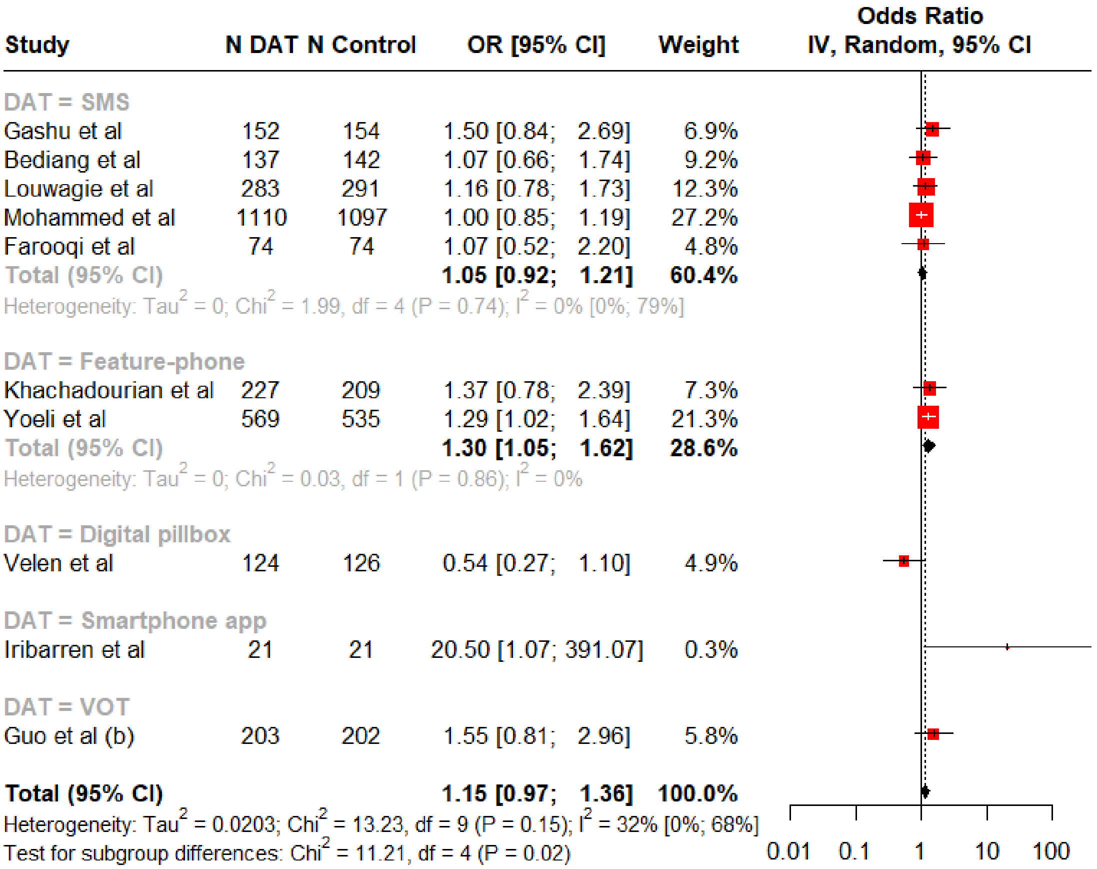

Figure S20b Forest plot showing cure rate in DAT groups compared to standard of care stratified by study design (RCTs)

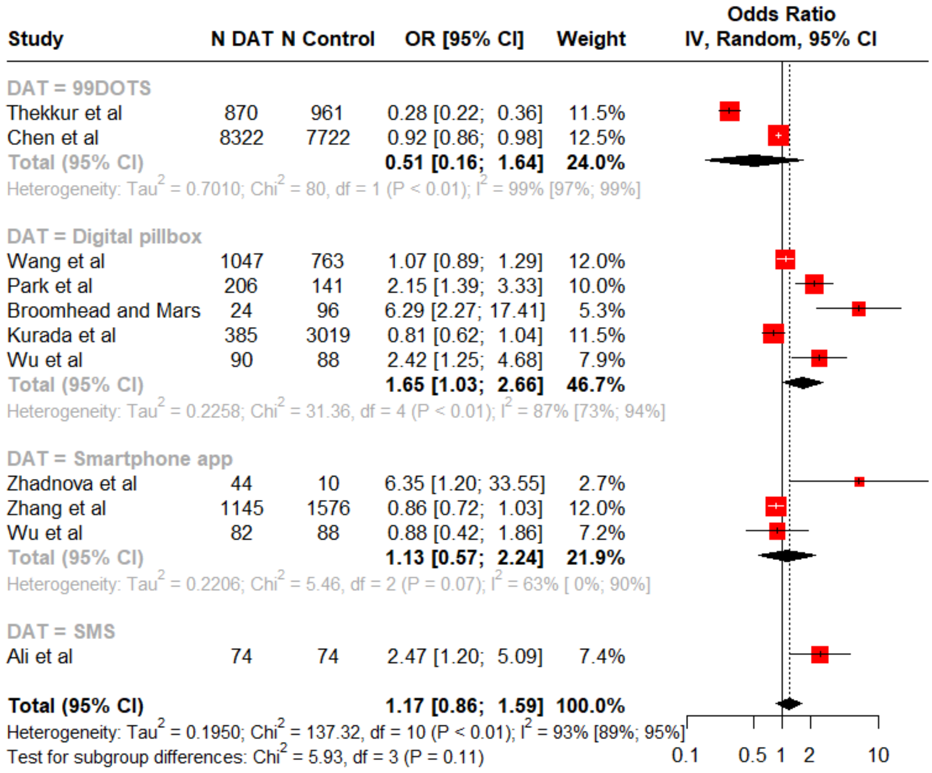

Figure S21c Forest plot showing cure rates in DAT groups compared to standard of care (observational studies)

Microbiologic conversion

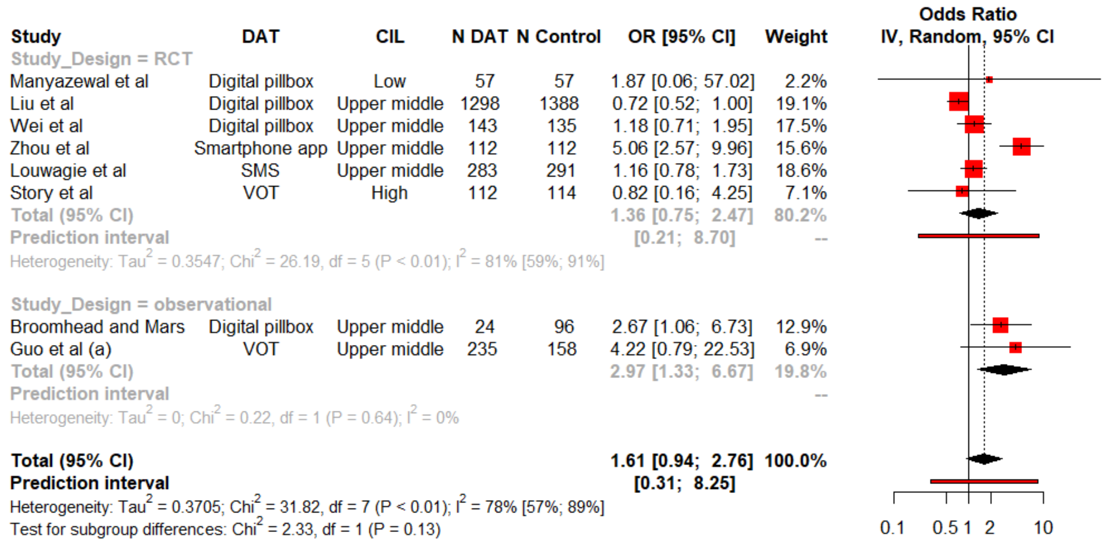

Figure S22a Forest plot showing microbiologic conversion of sputum smear in DAT groups compared to standard of care stratified by study design

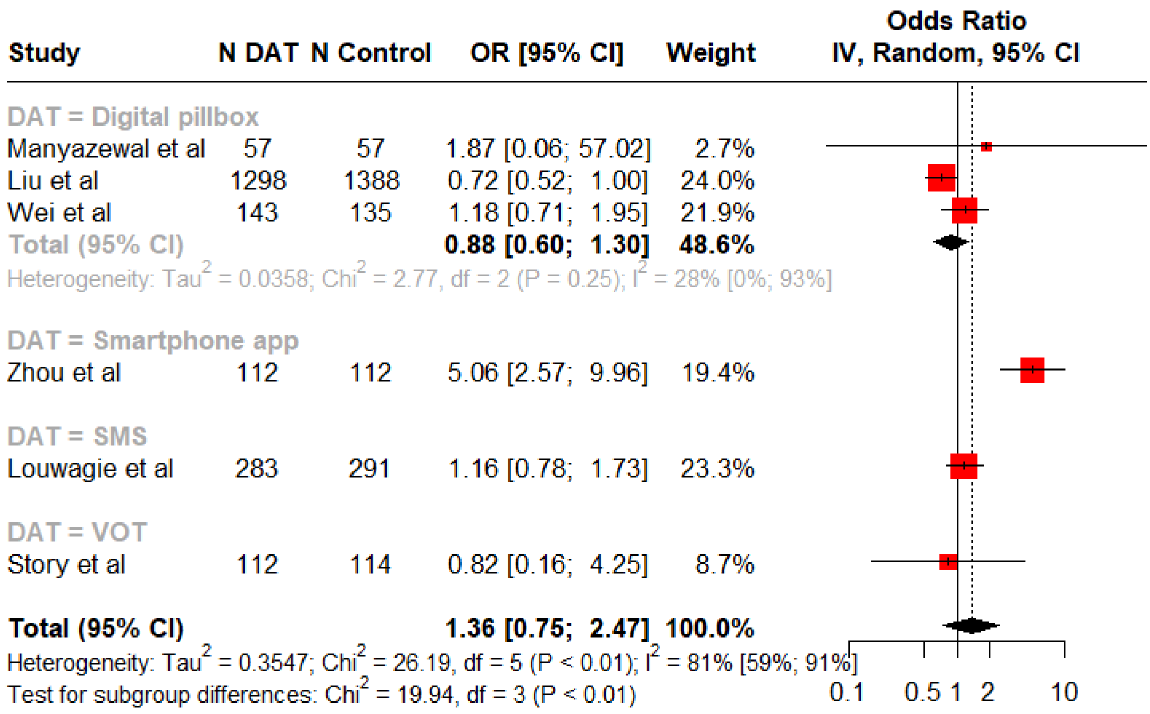

Figure S23b Forest plot showing microbiologic conversion of sputum smear in DAT groups compared to standard of care (RCTs)

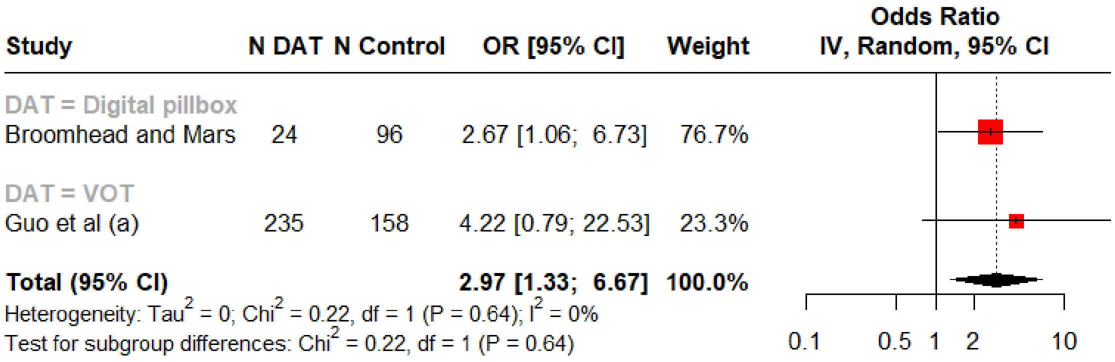

Figure S24c Forest plot showing microbiologic conversion of sputum smear in DAT groups compared to standard of care (observational studies)

Completion of intensive phase

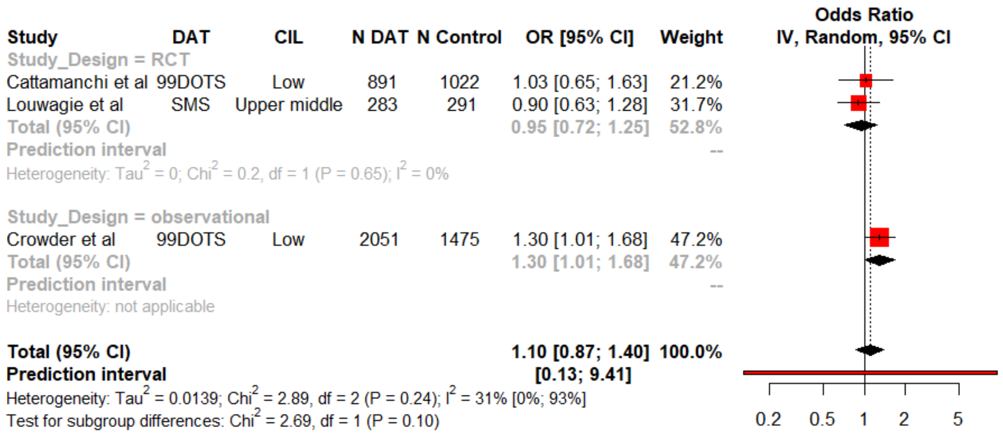

Figure S25a Forest plot showing completion of intensive phase treatment in DAT groups compared to standard of care stratified by study design

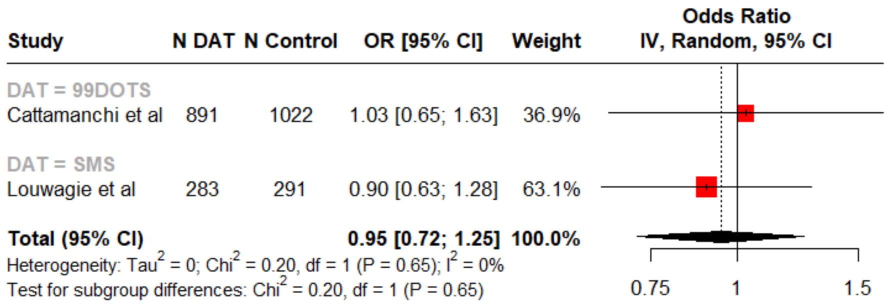

Figure S26b Forest plot showing completion of intensive phase treatment in DAT groups compared to standard of care (RCTs)

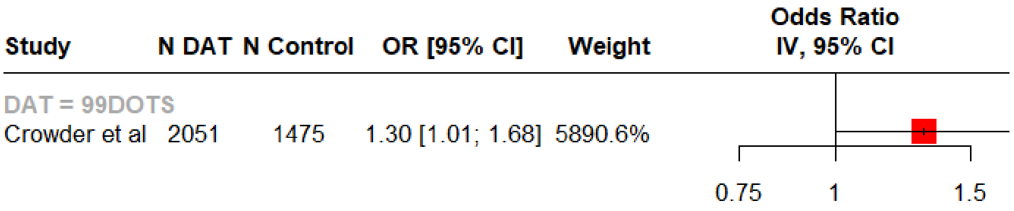

Figure S27c Forest plot showing completion of intensive phase treatment in DAT groups compared to standard of care (observational studies)

Emergence of anti-TB drug resistance

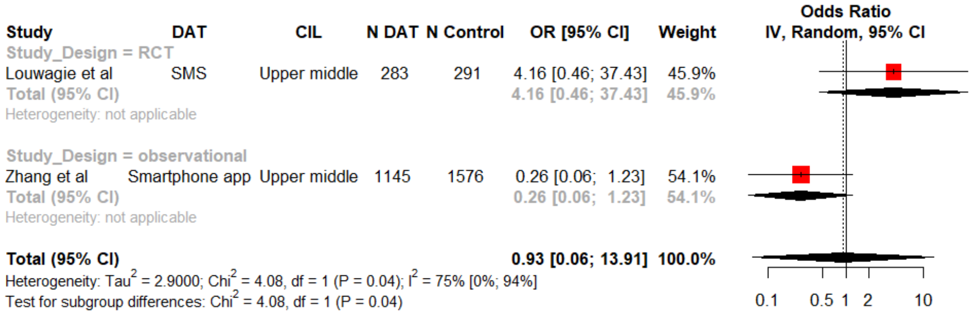

Figure S28 Forest plot showing emergence of anti-TB drug resistance in DAT groups compared to standard of care

Table S13 Pooled estimates for treatment success in TB disease stratified by type of DAT, subtype of DAT and country-income-level. Evidence is pooled from RCTs and observational studies

		Primary analysis					Sensitivity analysis		
	**Subgroup**	**Studies k**	**Participants N DAT/N SoC**	**OR [95% CI]**	**95% Prediction Interval**	**I^2 (%)**	**Studies k**	**OR [95% CI]**	**I^2 (%)**
**SMS**	**All**	**9**	**3341/3583**	**1.18 [0.92; 1.52]**	**[0.61; 2.29]**	**48.2**	**8**	**1.21 [0.91; 1.60]**	**54.7**
	One-way SMS	6	1040/1080	1.36 [0.91; 2.03]	[0.44; 4.16]	51.9	5	1.44 [0.88; 2.35]	61.4
	Two-way SMS	3	2301/2503	1.03 [0.73; 1.46]	[0.03; 35.29]	49.2	3	1.03 [0.73; 1.46]	49.2
	Low income	2	335/456	1.03 [0.57; 1.85]	-	55.9	2	1.03 [0.57; 1.85]	55.9
	Lower middle income	3	1321/1313	1.07 [0.87; 1.32]	[0.28; 4.10]	0.0	3	1.07 [0.87; 1.32]	0.0
	Upper middle income	4	1685/1814	1.56 [0.79; 3.09]	[0.08; 29.45]	73.3	3	1.83 [0.67; 4.98]	82.2
**VOT**	**All**	**8**	**695/1047**	**1.54 [1.09; 2.19]**	**[1.00; 2.38]**	**0.0**	**7**	**1.54 [1.08; 2.19]**	**0.0**
	Asynchronous	4	334/347	1.59 [1.00; 2.52]	[0.58; 4.38]	0.0	4	1.59 [1.00; 2.52]	0.0
	Synchronous	4	361/700	1.48 [0.87; 2.53]	[0.46; 4.77]	0.0	3	1.47 [0.84; 2.55]	0.0
	Upper-middle income	2	288/295	1.52 [0.74; 3.13]	-	0.0	2	1.52 [0.74; 3.13]	0.0
	High income	6	407/752	1.55 [1.04; 2.31]	[0.88; 2.72]	0.0	5	1.54 [1.03; 2.32]	0.0
**Digital pillbox**	**All**	**16**	**11478/18439**	**1.06 [0.91; 1.23]**	**[0.68; 1.64]**	**70**	**15**	**1.04 [0.89; 1.21]**	**70.6**
	Real-time monitoring	12	1160/3723	1.06 [0.83; 1.36]	[0.49; 2.28]	70.3	11	1.02 [0.79; 1.33]	72.1
	No real-time monitoring	4	3406/3314	1.00 [0.94; 1.08]	[0.96; 1.04]	0.0	4	1.00 [0.94; 1.08]	3.1
	Lower middle income	8	6519/12978	1.05 [0.82; 1.68]	[0.37; 3.71]	70.3	7	1.13 [0.76; 1.67]	74.7
	Upper middle income	8	4959/5461	1.05 [0.89; 1.25]	[0.69; 1.61]	73.4	8	1.05 [0.89; 1.25]	70.3
**Feature-phone based**	**All**	**8**	**1926/2288**	**1.18 [0.64; 2.2]**	**[0.14; 9.78]**	**86**	**6**	**1.00 [0.46; 2.17]**	**89.5**
	Low income	2	339/379	1.98 [1.16; 3.39]	-	0.0	0	-	-
	Lower middle income	4	1067/1470	1.10 [0.31; 3.91]	[0.0; 438.18]	91.9	4	1.10 [0.31; 3.91]	91.9
	Upper middle income	2	520/439	0.84 [0.61; 1.14]	-	0.0	2	0.84 [0.61; 1.14]	0.0
**99DOTS**	**All**	**8**	**17792/21036**	0.91 [0.74; 1.11]	**[0.51; 1.63]**	**70**	**7**	**0.89 [0.73; 1.10]**	**73.4**
	Low income	3	3251/2629	1.03 [0.85; 1.25]	[0.30; 3.53]	0.0	1	1.04 [0.68; 1.59]	-
	Lower middle income	4	13328/16527	0.78 [0.61; 1.01]	[0.27; 2.24]	71.0	4	0.78 [0.61; 1.01]	71.0
	Upper middle income	1	1213 /1880	0.84 [0.59; 1.21]	-	-	1	0.84 [0.59; 1.21]	-
**Smartphone apps**	**All**	**6**	**1459/1862**	**1.74 [1.00; 3.03]**	**[0.41; 7.47]**	**49**	**5**	**2.09 [0.88; 4.98]**	**58.6**
**SMS+ Digital pillbox**	**All**	**1**	**1064/1104**	**1.00 [0.45; 2.21]**	**-**	**-**	**1**	**1.00 [0.45; 2.21]**	**-**

Primary analysis summarizes evidence from all studies that reported on the outcome of interest. Sensitivity analysis summarizes evidence from all included studies after excluding studies published as conference abstracts

Table S14 Pooled estimates for loss to follow up stratified by type of DAT, subtype of DAT and country-income-level Evidence is pooled from RCTs and observational studies

		Primary analysis					Sensitivity analysis		
	**Subgroup**	**Studies k**	**Participants N DAT/N SoC**	**OR [95% CI]**	**95% Prediction Interval**	**I² (%)**	**Studies k**	**OR [95% CI]**	**I² (%)**
**SMS**	**All**	**9**	**3181/3428**	**0.85 [0.57; 1.25]**	**[0.27; 2.64]**	**64.7**	**9**	**0.85 [0.57; 1.25]**	**64.7**
	One-way SMS	6	880/925	0.87 [0.46; 1.61]	[0.12; 6.07]	72.7	6	0.87 [0.46; 1.61]	72.7
	Two-way SMS	3	2301/2503	0.83 [0.49; 1.41]	[0.00; 199.76]	49.9	3	0.83 [0.49; 1.41]	49.9
	Low income	3	409/530	0.91 [0.49; 1.68]	[0.02; 50.54]	0.0	3	0.91 [0.49; 1.68]	0.0
	Lower middle income	3	1321/1313	1.01 [0.73; 1.39]	[0.06; 16.22]	21.0	3	1.01 [0.73; 1.39]	21.0
	Upper middle income	3	1451/1585	0.73 [0.25; 2.12]	[0.00; 344465.83]	87.3	3	0.73 [0.25; 2.12]	87.3
**VOT**	**All**	**4**	**419/716**	**1.16 [0.46; 2.95]**	**[0.15; 9.01]**	**0.0**	**4**	**1.16 [0.46; 2.95]**	**0.0**
	Asynchronous	2	155/185	0.59 [0.14; 2.54]	-	6.1	2	0.59 [0.14; 2.54]	6.1
	Synchronous	2	264/531	1.96 [0.56; 6.82]	-	0.0	2	1.96 [0.56; 6.82]	0.0
	Upper-middle income	1	203/202	1.73 [0.40; 7.41]	-	-	1	1.73 [0.40; 7.41]	-
	High income	3	216/514	0.88 [0.24; 3.18]	[0.00; 11577.02]	9.7	3	0.88 [0.24; 3.18]	9.7
**Digital pillbox**	**All**	**12**	**10081/17333**	**0.57 [0.28; 1.17]**	**[0.04; 7.31]**	**86.0**	**11**	**0.61 [0.29; 1.26]**	**87.1**
	Real-time monitoring	9	7729/14784	0.55 [0.20; 1.52]	[0.02; 19.72]	90.5	8	0.55 [0.20; 1.52]	90.5
	No real-time monitoring	3	2352/2549	0.87 [0.60; 1.24]	[0.39; 1.91]	0.0	3	0.86 [0.58; 1.28]	0.0
	Low income	1	57/57	2.02 [0.07; 61.37]	-	-	1	2.02 [0.07; 61.37]	-
	Lower middle income	6	6255/12719	0.44 [0.11; 1.80]	[0.00; 64.53]	91.7	5	0.50 [0.11; 2.30]	93.3
	Upper middle income	5	3769/4557	0.74 [0.44; 1.26]	[0.15; 3.63]	55.2	5	0.74 [0.44; 1.26]	55.2
**Feature-phone based**	**All**	**5**	**1228/1214**	**1.14 [0.31; 4.22]**	**[0.01; 159.92]**	**90.0**	**4**	**1.48 [0.26; 8.31]**	**92.2**
	low income	1	210/248	0.45 [0.19; 1.05]	-	-	**0**	-	-
	lower middle income	3	791/757	1.52 [0.12; 19.82]	[0.00; 197873844197014.]	94.3	**3**	1.52 [0.12; 19.82]	94.3
	upper middle income	1	227/209	1.51 [0.67; 3.40]	-	-	**1**	1.51 [0.67; 3.40]	-
**99DOTS**	**All**	**7**	**17483/20904**	**1.00 [0.73; 1.37]**	**[0.38; 2.63]**	**72.1**	**7**	**1.00 [0.73; 1.37]**	**72.1**
	Low income	2	2942/2497	0.77 [0.41; 1.44]	-	75.6	**2**	0.77 [0.41; 1.44]	75.6
	Lower middle income	4	13328/16527	1.15 [0.75; 1.75]	[0.21; 6.39]	67.6	**4**	1.15 [0.75; 1.75]	67.6
	Upper middle	1	1213/1880	1.09 [0.62; 1.90]	-	-	**1**	1.09 [0.62; 1.90]	-
**Smartphone apps**	**All**	**5**	**1377/1774**	**0.31 [0.13; 0.77]**	**[0.03; 3.42]**	**35.8**	**4**	**0.22 [0.08; 0.57]**	**10.3**
**SMS+ Digital pillbox**	**All**	**1**	**1064/1104**	**0.90 [0.38; 2.11]**	**-**	**-**	**1**	**0.90 [0.38; 2.11]**	**-**

Primary analysis summarizes evidence from all studies that reported on the outcome of interest. Sensitivity analysis summarizes evidence from all included studies after excluding studies published as conference abstracts

Table S15 Pooled estimates for treatment failure in TB disease stratified by type of DAT, subtype of DAT and country-income-level Evidence is pooled from RCTs and observational studies

		Primary analysis					Sensitivity analysis		
	**Subgroup**	**Studies k**	**Participants N DAT/N SoC**	**OR [95% CI]**	**95% Prediction Interval**	**I^2 (%)**	**Studies k**	**OR [95% CI]**	**I^2 (%)**
**SMS**	**All**	**6**	**2810/3022**	**1.09 [0.62; 1.91]**	**[0.37; 3.24]**	**12.2**	**6**	**1.09 [0.62; 1.91]**	**12.2**
	One-way SMS	3	509/519	0.51 [0.15; 1.74]	[0.00; 1417.81]	0.0	3	0.51 [0.15; 1.74]	0.0
	Two-way SMS	3	2301/2503	1.58 [0.64; 3.94]	[0.00; 19115.01]	48.9	3	1.58 [0.64; 3.94]	48.9
	Low income	2	335/456	1.01 [0.19; 5.28]	-	57.9	2	1.01 [0.19; 5.28]	57.9
	Lower middle income	2	1184/1171	0.92 [0.55; 1.55]	-	0.0	2	0.92 [0.55; 1.55]	0.0
	Upper middle income	2	1291/1395	1.76 [0.23; 13.66]	-	50.8	2	1.76 [0.23; 13.66]	50.8
**VOT**	**All**	**1**	**203/202**	**0.79 [0.21; 2.99]**	**-**	**-**	**1**	**0.79 [0.21; 2.99]**	**-**
**Digital pillbox**	**All**	**8**	**4575/7030**	**1.07 [0.67; 1.69]**	**[0.37; 3.05]**	**48.3**	**7**	**1.08 [0.66;1.78]**	**55.0**
	Real-time monitoring	6	2531/5163	1.06 [0.46; 2.43]	[0.13; 8.36]	41.6	5	1.12 [0.43; 2.90]	48.6
	No real-time monitoring	2	2044/1867	1.00 [0.98; 1.03]	-	0.0	2	1.00 [0.98; 1.03]	0.0
	Lower middle income	5	2441/5075	0.98 [0.39; 2.51]	[0.06; 15.03]	53.2	4	1.03 [0.34; 3.10]	61.4
	Upper middle income	3	2134/1955	1.00 [0.98; 1.03]	[0.85; 1.17]	0.0	3	1.00 [0.98; 1.03]	0.0
**Feature-phone based**	**All**	**4**	**1018/966**	**0.55 [0.11; 2.73]**	**[0.00; 461.23]**	**82.6**	**4**	**0.55 [0.11; 2.73]**	**82.6**
	lower middle income	3	791/757	0.19 [0.10; 0.36]	[0.00; 13.03]	0.0	3	0.19 [0.10; 0.36]	0.0
	upper middle income	1	227/209	1.51 [0.67; 3.40]	-	-	1	1.51 [0.67; 3.40]	-
**99DOTS**	**All**	**2**	**9192/8683**	**0.90 [0.24; 3.46]**	**-**	**27.9**	**2**	**0.90 [0.24; 3.46]**	**27.9**
**Smartphone apps**	**All**	**4**	**1394/1831**	**0.78 [0.38; 1.58]**	**[0.17; 3.65]**	**0.0**	3	**1.01 [0.29; 3.46]**	**0.0**
**SMS+ Digital pillbox**	**All**	**1**	**1064/1104**	**1.07 [0.22; 5.34]**	**-**	**-**	**1**	**1.07 [0.22; 5.34]**	**-**
**Digital pillbox or 99DOTS**	**All**	**3**	**10644/9724**	**2.12 [1.34; 3.37]**	**[0.11; 42.14]**	**0.0**	**3**	**2.12 [1.34; 3.37]**	**0.0**

Primary analysis summarizes evidence from all studies that reported on the outcome of interest. Sensitivity analysis summarizes evidence from all included studies after excluding studies published as conference abstracts

Table S16 Pooled estimates for death in TB disease stratified by type of DAT, subtype of DAT and country-income-level. Evidence is pooled from RCTs and observational studies

		Primary analysis					Sensitivity analysis		
	**Subgroup**	**Studies k**	**Participants N DAT/N SoC**	**OR [95% CI]**	**95% Prediction Interval**	**I^2 (%)**	**Studies k**	**OR [95% CI]**	**I^2 (%)**
**SMS**	**All**	**6**	**2873/3090**	**1.19 [0.78; 1.83]**	**[0.44; 3.23]**	**30.9**	**6**	**1.19 [0.78; 1.83]**	**30.9**
	One-way SMS	3	572/587	0.84 [0.32; 2.22]	[0.00; 5842.54]	29.0	3	0.84 [0.32; 2.22]	29.0
	Two-way SMS	3	2301/2503	1.31 [0.76; 2.27]	[0.01; 342.77]	49.8	3	1.31 [0.76; 2.27]	49.8
	Low income	2	335/456	0.91 [0.55; 1.51]	-	0.0	2	0.91 [0.55; 1.51]	0.0
	Lower middle income	2	1247/1239	0.97 [0.17; 5.46]	-	76.3	2	0.97 [0.17; 5.46]	76.3
	Upper middle income	2	1291/1395	1.32 [0.69; 2.52]	-	0.0	2	1.32 [0.69; 2.52]	0.0
**VOT**	**All**	**4**	**470/714**	**0.79 [0.22; 2.84]**	**[0.05; 13.05]**	**0.0**	**4**	**0.79 [0.22; 2.84]**	**0.0**
	Asynchronous	2	206/183	0.55 [0.11; 2.84]	-	0.0	2	0.55 [0.11; 2.84]	0.0
	Synchronous	2	264/531	1.38 [0.18; 10.36]	-	0.0	2	1.38 [0.18; 10.36]	0.0
	Upper-middle income	1	203/202	0.67 [0.01; 33.71]	-	-	1	0.67 [0.01; 33.71]	-
	High income	3	267/512	0.81 [0.21; 3.16]	[0.00; 5119.16]	0.0	3	0.81 [0.21; 3.16]	0.0
**Digital pillbox**	**All**	**13**	**9977/16861**	**1.17 [0.94; 1.44]**	**[0.76; 1.79]**	**20.**	**12**	**1.22 [1.00; 1.48]**	**11.5**
	Real-time monitoring	10	7876/14937	1.23 [0.98; 1.54]	[0.78; 1.94]	21.2	9	1.29 [1.07; 1.56]	4.8
	No real-time monitoring	3	2101/1924	0.85 [0.53; 1.36]	[0.04; 17.73]	0.0	3	0.85 [0.53; 1.36]	0.0
	Low income	1	57/57	1.00 [0.02; 51.28]	-	-	1	1.00 [0.02; 51.28]	-
	Lower middle income	7	6455 12919	1.24 [0.92; 1.67]	[0.6171; 2.5001]	0.0%	6	1.35 [1.05; 1.74]	20.8
	Upper middle income	5	3465 3885	0.96 [0.69; 1.32]	[0.5670; 1.6189]	37.5%	5	0.96 [0.69; 1.32]	0.0
**Feature-phone based**	**All**	**4**	**1018/966**	**1.05 [0.37; 2.95]**	**[0.03; 31.89]**	**29.9**	**4**	**1.05 [0.37; 2.95]**	**29.9**
	lower middle income	3	791/757	1.69 [0.27; 10.69]	[0.00; 485620478.87]	52.9	3	1.69 [0.27; 10.69]	52.9
	upper middle income	1	227/209	0.92 [0.18; 4.61]	-	-	1	0.92 [0.18; 4.61]	-
**99DOTS**	**All**	**5**	**14541/18407**	**1.35 [1.13; 1.61]**	**[0.84; 2.18]**	**37.8**	**5**	**1.35 [1.13; 1.61]**	**37.8**
**Smartphone apps**	**All**	**5**	**1415/1852**	**1.29 [0.80; 2.08]**	**[0.59; 2.80]**	**0.0**	**4**	**1.47 [0.86; 2.51]**	**0.0**
**SMS+ Digital pillbox**	**All**	**1**	**1064/1104**	**1.27 [0.42; 3.79]**	**-**	**-**	**1**	**1.27 [0.42; 3.79]**	**-**

Primary analysis summarizes evidence from all studies that reported on the outcome of interest. Sensitivity analysis summarizes evidence from all included studies after excluding studies published as conference abstracts

Table S17 Pooled estimates for reporting of adverse events in TB disease stratified by type of DAT, subtype of DAT and country-income-level. Evidence is pooled from RCTs and observational studies

		Primary analysis					Sensitivity analysis		
	Subgroup	Studies k	Participants N DAT/N SoC	OR [95% CI]	95% Prediction Interval	I² (%)	Studies k	OR [95% CI]	I² (%)
**SMS**	**All**	**2**	**1068/1164**	**1.23 [0.84; 1.80]**	**-**	**0.0**	**2**	**1.23 [0.84; 1.80]**	**0.0**
	One-way SMS	1	60/60	1.22 [0.60; 2.50]	-	-	1	1.22 [0.60; 2.50]	-
	Two-way SMS	1	1008/1104	1.24 [0.79; 1.93]	-	-	1	1.24 [0.79; 1.93]	-
	Lower middle income	1	60/60	1.22 [0.60; 2.50]	-	-	1	1.22 [0.60; 2.50]	-
	Upper middle income	1	1008/1104	1.24 [0.79; 1.93]	-	-	1	1.24 [0.79; 1.93]	-
**VOT**	**All**	**5**	**606/797**	**1.72 [1.26; 2.34]**	**[1.04; 2.84]**	**0.0**	**5**	**1.72 [1.26; 2.34]**	**0.0**
	Asynchronous	3	432/365	1.78 [1.26; 2.51]	[0.19; 16.64]	0.0	3	1.78 [1.26; 2.51]	0.0
	Synchronous	1	61/329	2.74 [0.09; 82.87]	-	-	1	2.74 [0.09; 82.87]	-
	Mixed synchronous and asynchronous	1	113/103	1.42 [0.68; 2.97]	-	-	1	1.42 [0.68; 2.97]	-
	Upper-middle income	2	320/251	1.55 [1.04; 2.32]	-	0.0	2	1.55 [1.04; 2.32]	0.0
	High income	3	286<br>546	2.00 [1.22; 3.28]	[0.0809; 49.3887]	0.0	3	2.00 [1.22; 3.28]	0.0
**Digital pillbox**	**All**	**1**	**997/1104**	**1.22 [0.78; 1.91]**	**-**	**-**	**1**	**1.22 [0.78; 1.91]**	**-**
**SMS+ Digital pillbox**	**All**	**1**	**1064/1104**	**1.91 [1.27; 2.88]**	**-**	**-**	**1**	**1.91 [1.27; 2.88]**	**-**

Primary analysis summarizes evidence from all studies that reported on the outcome of interest. Sensitivity analysis summarizes evidence from all included studies after excluding studies published as conference abstracts

Table S18 Pooled estimates for microbiologic conversion stratified by type of DAT. Evidence is pooled from RCTs and observational studies

		Primary analysis					Sensitivity analysis		
	Subgroup	Studies k	Participants N DAT/N SoC	OR [95% CI]	95% Prediction Interval	I² (%)	Studies k	OR [95% CI]	I² (%)
**SMS**	**All**	**1**	**283/291**	**1.16 [0.78; 1.73]**	**-**	**-**	**1**	**1.16 [0.78; 1.73]**	**-**
**VOT**	**All**	**2**	**347/272**	**1.85 [0.37; 9.18]**	**-**	**46.5**	**2**	**1.85 [0.37; 9.18]**	**46.5**
**Digital pillbox**	**All**	**4**	**1522/1676**	**1.17 [0.65; 2.09]**	**[0.12; 11.24]**	**63.9**	**4**	**1.17 [0.65; 2.09]**	**63.9**
**Smartphone apps**	**All**	**1**	**112/112**	**5.06 [2.57; 9.96]**	**-**	**-**	**0**	**-**	**-**

Primary analysis summarizes evidence from all studies that reported on the outcome of interest. Sensitivity analysis summarizes evidence from all included studies after excluding studies published as conference abstracts

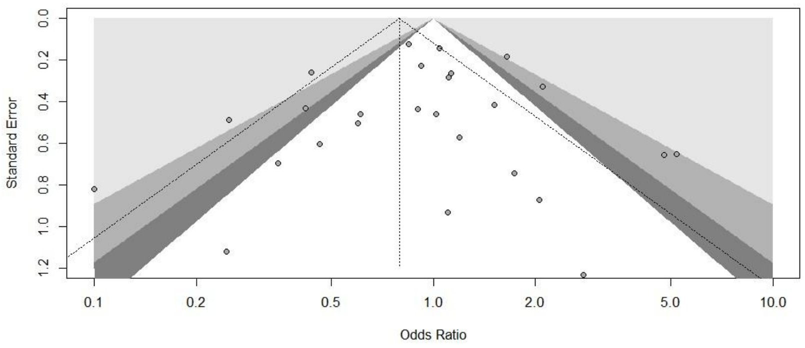


```
Eggers' test of the intercept
=============================

 intercept      95% CI       t          p
    -0.394 -1.49 - 0.7 -0.705 0.4846207

Eggers' test does not indicate the prese
nce of funnel plot asymmetry.
```

Figure S29 Funnel plot of included studies for the qualitative assessment of publication bias using Egger's test. It shows studies comparing LTFU in patients using DATs versus standard care. Each point represents an individual study

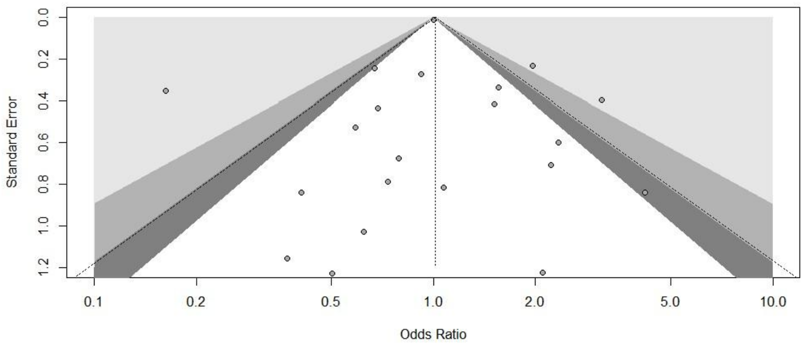


```
Eggers' test of the intercept
=============================

 intercept       95% CI     t          p
     0.029 -0.56 - 0.62 0.098 0.9229182

Eggers' test does not indicate the
presence of funnel plot asymme
```

Figure S30 Funnel plot of included studies for the qualitative assessment of publication bias using Egger's test. It shows studies comparing treatment failure in patients using DATs versus standard care. Each point represents an individual study

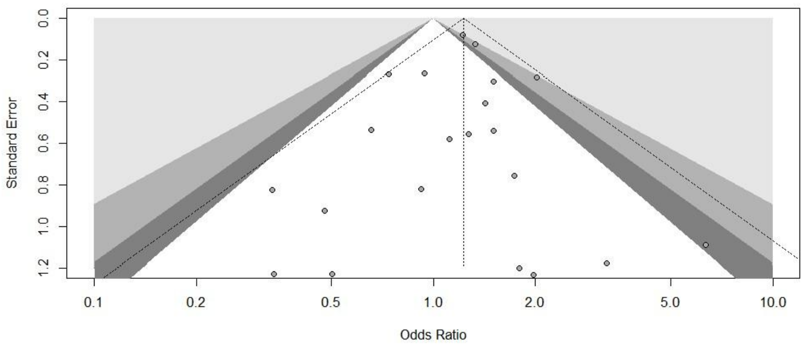


```
Eggers' test of the intercept
=============================

 intercept       95% CI       t          p
    -0.141 -0.61 - 0.33 -0.591 0.5580443

Eggers' test does not indicate the
presence of funnel plot asymmetry.
```

Figure S31 Funnel plot of included studies for the qualitative assessment of publication bias using Egger's test. It shows studies comparing death during treatment in patients using DATs versus standard care. Each point represents an individual study

Quantitative synthesis of evidence- TB infection

Table S19 Pooled estimates for treatment completion in l LTBI

	Studies N	Participants N	OR [95% CI]	95% Prediction Interval	Heterogeneity I^2 (%)
All	4	1615	1.77 [0.85; 3.66]	[0.08; 37.30]	77
SMS	2	1023	1.05 [0.78; 1.40)	-	0
VOT	2	592	4.69 [2.08; 10.55]	-	0

Subgroup analysis based on country income level

Study	DAT	N DAT	N Control	OR [95% CI]	Weight
country_income_level = Low					
Cattamanchi et al	99DOTS	891	1022	1.04 [0.68; 1.59]	4.2%
Hope et al	Feature-phone	129	131	2.29 [0.56; 9.34]	0.8%
Gashu et al	SMS	152	154	1.49 [0.75; 2.95]	2.6%
Total (95% CI)				**1.20 [0.85; 1.70]**	**7.6%**
Heterogeneity: Tau^2 = 0; Chi^2 = 1.64, df = 2 (P = 0.44); I^2 = 0% [0%; 90%]					
country_income_level = Lower middle					
Jerene et al - Philippines	99DOTS	2368	4188	0.87 [0.51; 1.48]	3.4%
Jerene et al - Tanzania	99DOTS	1768	3656	0.55 [0.34; 0.91]	3.7%
Yoeli et al	Feature-phone	569	535	3.42 [2.12; 5.52]	3.8%
Jerene et al - Philippines	Digital pillbox	2236	4188	0.90 [0.53; 1.52]	3.5%
Jerene et al - Tanzania	Digital pillbox	1778	3656	0.79 [0.50; 1.25]	3.9%
Jerene et al - Ukraine	Digital pillbox	1526	1589	0.87 [0.63; 1.20]	5.1%
Moulding & Caymittes	Digital pillbox	64	59	2.41 [0.77; 7.52]	1.2%
Velen et al	Digital pillbox	124	126	1.11 [0.63; 1.97]	3.2%
Bediang et al	SMS	137	142	1.45 [0.82; 2.56]	3.2%
Farooqi et al	SMS	74	74	1.27 [0.33; 4.92]	0.9%
Mohammed et al	SMS	1110	1097	1.02 [0.81; 1.28]	6.0%
Total (95% CI)				**1.10 [0.82; 1.46]**	**37.6%**
Heterogeneity: Tau^2 = 0.1494; Chi^2 = 36.74, df = 10 (P < 0.01); I^2 = 73% [50%; 85%]					
country_income_level = Upper middle					
Jerene et al - South Africa	99DOTS	1213	1880	0.84 [0.59; 1.21]	4.7%
Bassett et al	Feature-phone	293	230	0.87 [0.62; 1.24]	4.8%
Khachadourian et al	Feature-phone	227	209	0.70 [0.35; 1.42]	2.5%
Acosta et al	Digital pillbox	53	53	1.71 [0.52; 5.60]	1.1%
Jerene et al - South Africa	Digital pillbox	1281	1880	0.82 [0.57; 1.18]	4.8%
Liu et al (2015)	Digital pillbox	997	1104	1.41 [0.66; 3.01]	2.2%
Liu et al (2023)	Digital pillbox	1298	1388	0.97 [0.69; 1.36]	5.0%
Ratchakit-Nedsuwan et al	Digital pillbox	50	50	0.46 [0.08; 2.67]	0.6%
Wei et al	Digital pillbox	143	135	1.29 [1.15; 1.44]	6.8%
Haslinda & Juni	Smartphone app	55	55	4.14 [1.15; 14.89]	1.0%
Iribarren et al	Smartphone app	21	21	4.71 [0.48; 46.22]	0.3%
Zhou et al	Smartphone app	112	112	1.53 [0.83; 2.81]	2.9%
Fang et al	SMS	160	190	3.89 [1.55; 9.74]	1.7%
Liu et al	SMS	1008	1104	2.27 [0.88; 5.86]	1.6%
Louwagie et al	SMS	283	291	0.86 [0.60; 1.24]	4.7%
Peng et al	SMS	234	229	1.10 [0.52; 2.34]	2.2%
Liu et al	SMS+ Digital pillbox	1064	1104	1.00 [0.45; 2.21]	2.1%
Guo et al (b)	VOT	203	202	1.40 [0.55; 3.57]	1.6%
Ravenscroft et al	VOT	85	93	1.71 [0.55; 5.33]	1.2%
Total (95% CI)				**1.13 [0.94; 1.34]**	**51.9%**
Heterogeneity: Tau^2 = 0.0521; Chi^2 = 35.28, df = 18 (P < 0.01); I^2 = 49% [13%; 70%]					
country_income_level = High					
Story et al	VOT	112	114	1.53 [0.82; 2.85]	2.9%
**Total (95% CI)**				**1.14 [0.99; 1.30]**	**100.0%**
Heterogeneity: Tau^2 = 0.0679; Chi^2 = 76.68, df = 33 (P < 0.01); I^2 = 57% [37%; 71%]					
Test for subgroup differences: Chi^2 = 1.02, df = 3 (P = 0.80)					

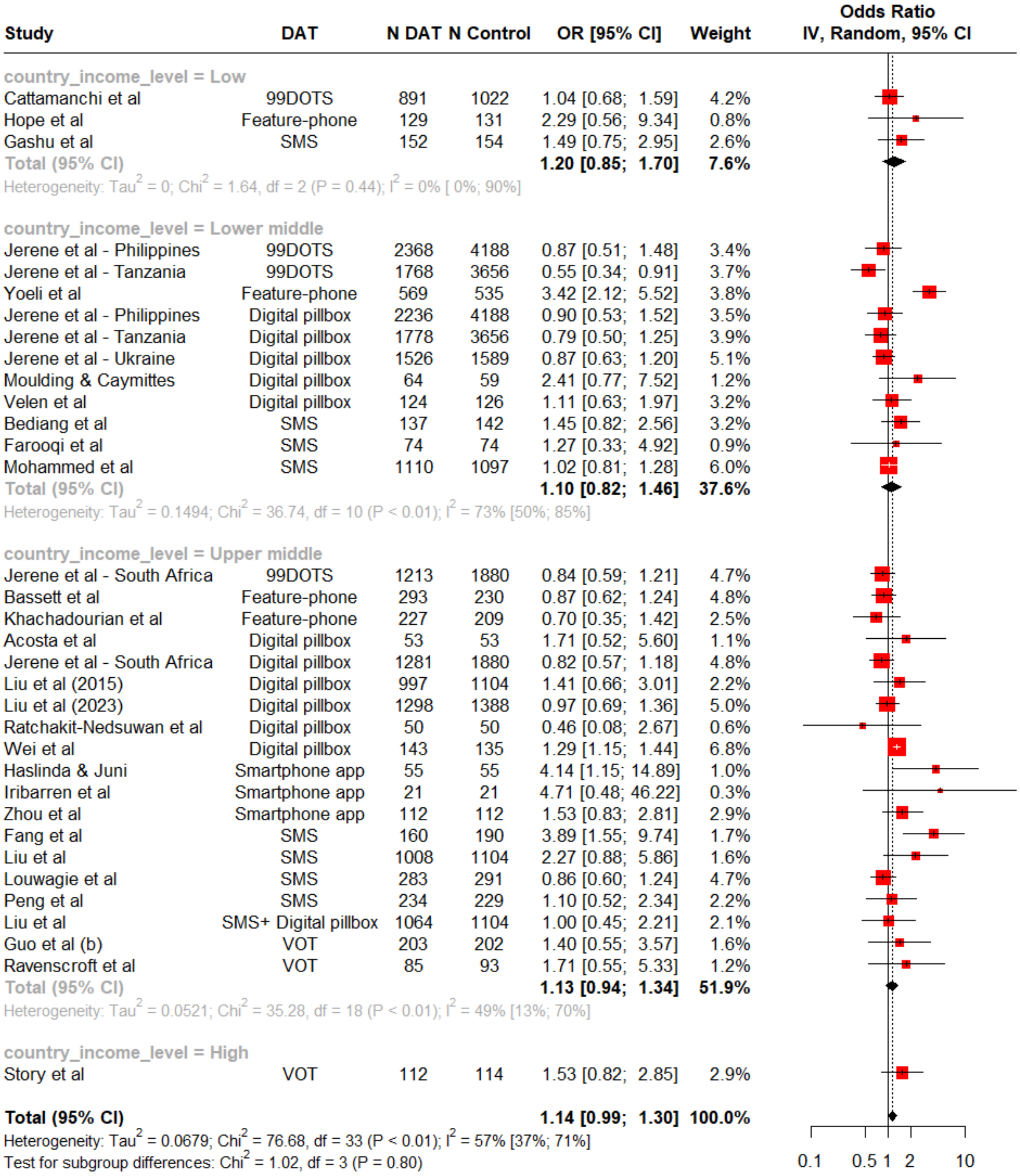

Figure S32a Forest plot showing treatment success with DATs compared to standard of care stratified by country income level (RCTs)

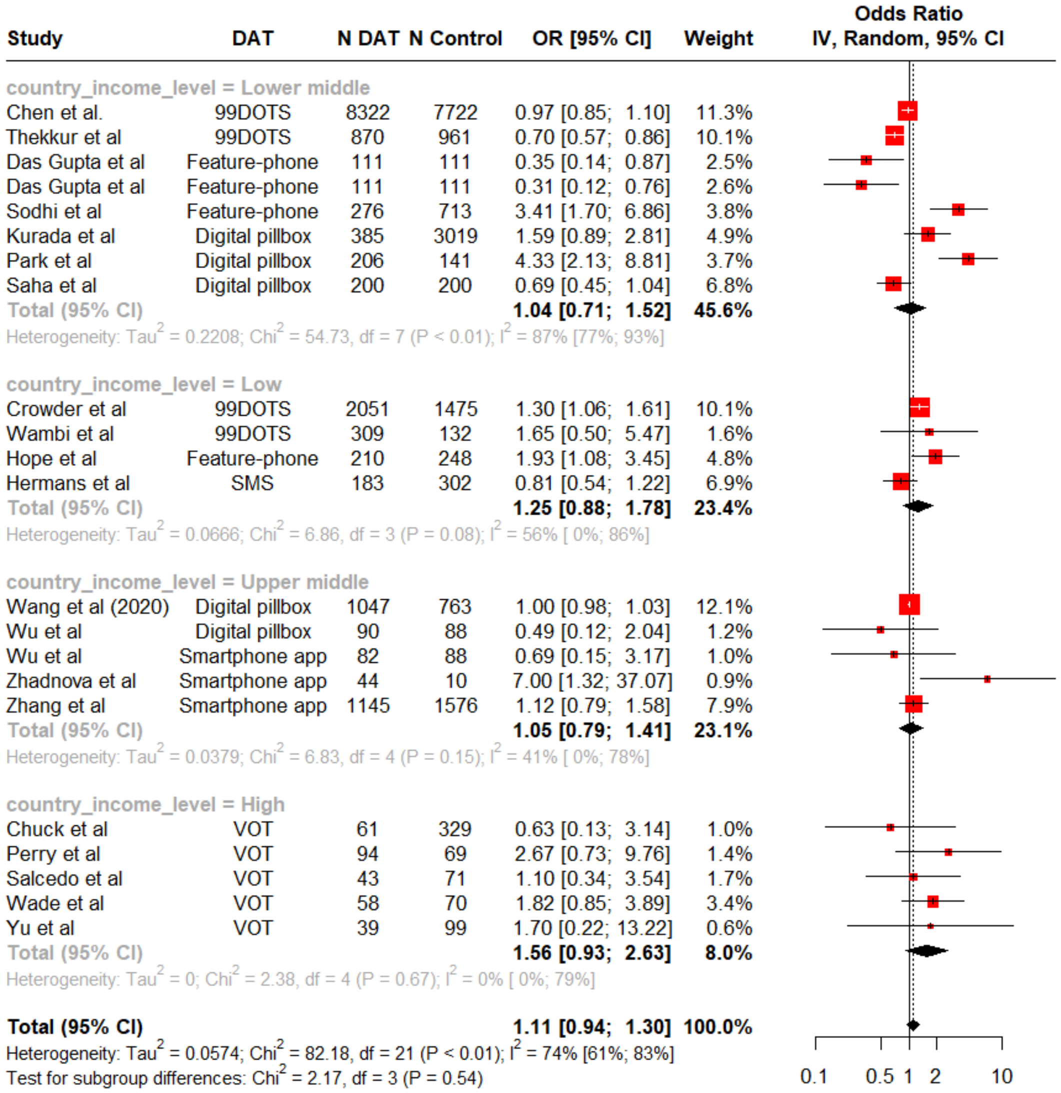

Figure S33 Forest plot showing treatment success with DATs compared to standard of care stratified by country income level (observational studies)

Study	DAT	N DAT	N Control	OR [95% CI]	Weight
country_income_level = Low					
Cattamanchi et al	99DOTS	891	1022	1.11 [0.64; 1.93]	5.2%
Manyazewal et al	Digital pillbox	57	57	2.02 [0.07; 61.37]	0.4%
Gashu et al	SMS	152	154	2.05 [0.37; 11.38]	1.5%
Gashu et al	SMS	152	154	2.05 [0.37; 11.38]	1.5%
Total (95% CI)				**1.25 [0.76; 2.05]**	**8.6%**
Heterogeneity: Tau^2 = 0; Chi^2 = 0.9, df = 3 (P = 0.83); I^2 = 0% [0%; 85%]					
country_income_level = Lower middle					
Jerene et al - Philippines	99DOTS	2368	4188	1.02 [0.42; 2.51]	3.5%
Jerene et al - Tanzania	99DOTS	1768	3656	1.23 [0.53; 2.87]	3.7%
Yoeli et al	Feature-phone	569	535	0.16 [0.08; 0.32]	4.5%
Velen et al	Digital pillbox	124	126	1.20 [0.39; 3.67]	2.7%
Jerene et al - Philippines	Digital pillbox	2236	4188	0.88 [0.37; 2.10]	3.6%
Jerene et al - Tanzania	Digital pillbox	1778	3656	0.65 [0.29; 1.47]	3.9%
Jerene et al - Ukraine	Digital pillbox	1526	1589	1.65 [0.68; 4.00]	3.5%
Bediang et al	SMS	137	142	1.13 [0.67; 1.89]	5.4%
Mohammed et al	SMS	1110	1097	1.04 [0.78; 1.38]	6.5%
Farooqi et al	SMS	74	74	0.35 [0.09; 1.37]	2.1%
Total (95% CI)				**0.81 [0.53; 1.23]**	**39.4%**
Heterogeneity: Tau^2 = 0.2903; Chi^2 = 30.68, df = 9 (P < 0.01); I^2 = 71% [44%; 85%]					
country_income_level = Upper middle					
Jerene et al - South Africa	99DOTS	1213	1880	1.09 [0.62; 1.90]	5.1%
Khachadourian et al	Feature-phone	227	209	1.51 [0.67; 3.40]	3.9%
Liu et al	Digital pillbox	997	1104	0.61 [0.25; 1.50]	3.5%
Ratchakit-Nedsuwan et al	Digital pillbox	50	50	1.03 [0.06; 16.98]	0.6%
Liu et al	Digital pillbox	1298	1388	0.92 [0.59; 1.44]	5.7%
Wei et al	Digital pillbox	143	135	0.25 [0.10; 0.65]	3.3%
Jerene et al - South Africa	Digital pillbox	1281	1880	1.25 [0.73; 2.14]	5.2%
Haslinda & Juni	Smartphone app	55	55	0.05 [0.00; 0.96]	0.6%
Zhou et al	Smartphone app	112	112	0.71 [0.27; 1.83]	3.3%
Iribarren et al	Smartphone app	21	21	0.23 [0.01; 5.47]	0.5%
Louwagie et al	SMS	283	291	2.10 [1.10; 4.01]	4.7%
Liu et al	SMS	1008	1104	0.42 [0.18; 0.98]	3.7%
Fang et al	SMS	160	190	0.44 [0.26; 0.73]	5.4%
Liu et al	SMS+ Digital pillbox	1064	1104	0.90 [0.38; 2.11]	3.7%
Guo et al (b)	VOT	203	202	1.73 [0.40; 7.41]	1.9%
Total (95% CI)				**0.81 [0.58; 1.13]**	**51.1%**
Heterogeneity: Tau^2 = 0.2163; Chi^2 = 33.57, df = 14 (P < 0.01); I^2 = 58% [26%; 76%]					
country_income_level = High					
Story et al	VOT	112	114	0.25 [0.03; 2.21]	1.0%
**Total (95% CI)**				**0.84 [0.66; 1.06]**	**100.0%**
Heterogeneity: Tau^2 = 0.1961; Chi^2 = 68.45, df = 29 (P < 0.01); I^2 = 58% [36%; 72%]					
Test for subgroup differences: Chi^2 = 3.61, df = 3 (P = 0.31)					

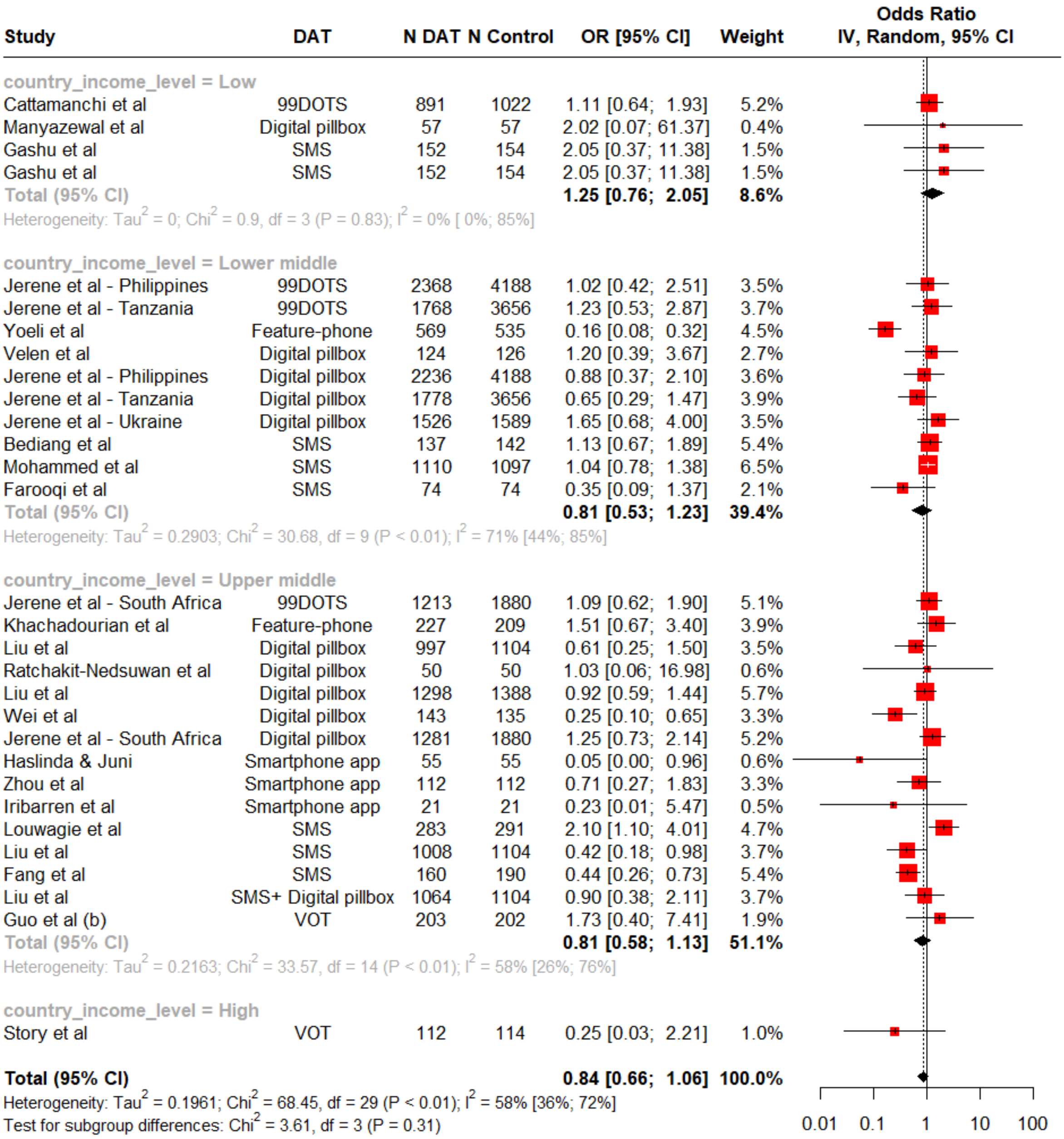

Figure S34a Forest plot showing losses to follow-up with DATs compared to standard of care stratified by country income level (RCTs)

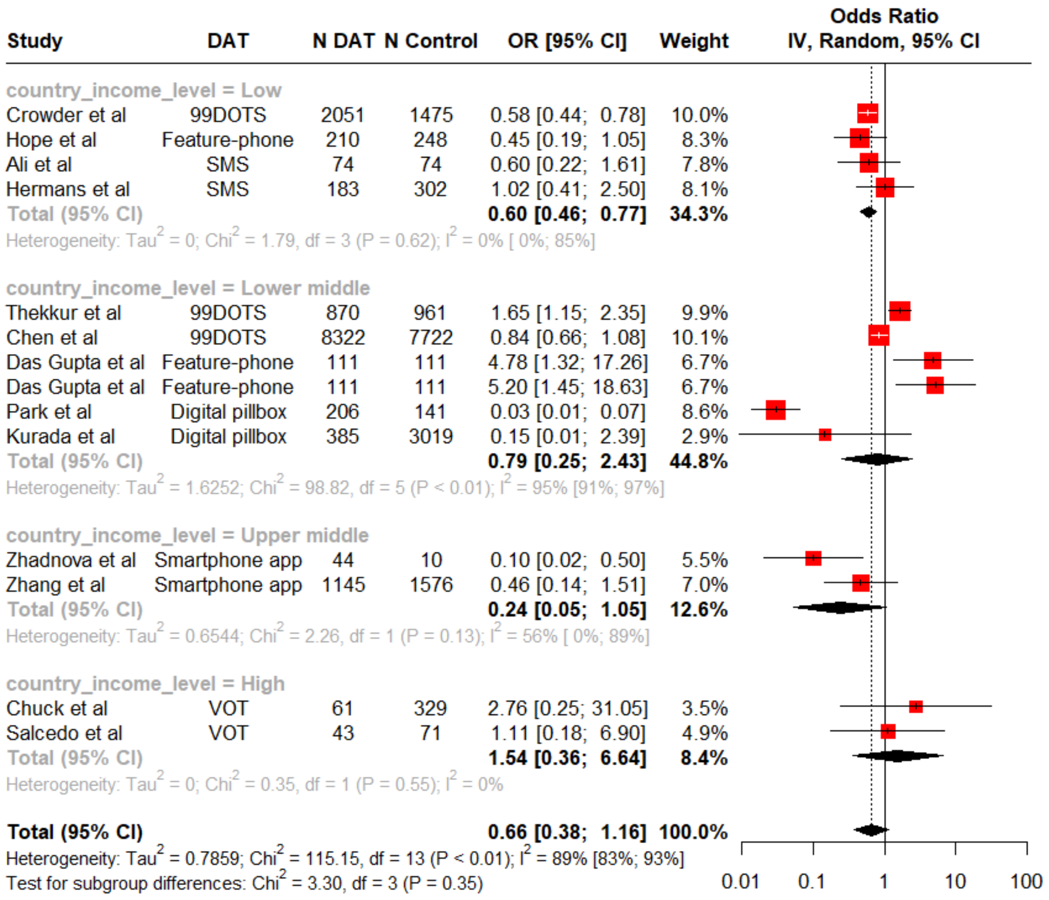

Figure S35b Forest plot showing losses to follow-up with DATs compared to standard of care stratified by country income level (observational studies)

Study	DAT	N DAT	N Control	OR [95% CI]	Weight
country_income_level = Low					
Gashu et al	SMS	152	154	0.50 [0.05; 5.61]	2.7%
country_income_level = Upper middle					
Khachadourian et al	Feature-phone	227	209	1.51 [0.67; 3.40]	7.9%
Liu et al	Digital pillbox	997	1104	0.37 [0.04; 3.58]	2.9%
Jerene et al - South Africa	Digital pillbox or 99DOTS	2494	1880	3.13 [1.44; 6.80]	8.1%
Haslinda & Juni	Smartphone app	55	55	0.74 [0.16; 3.45]	4.8%
Zhou et al	Smartphone app	112	112	0.69 [0.29; 1.62]	7.7%
Louwagie et al	SMS	283	291	0.41 [0.08; 2.12]	4.4%
Liu et al	SMS	1008	1104	2.21 [0.55; 8.88]	5.3%
Liu et al	SMS+Digital pillbox	1064	1104	1.07 [0.22; 5.34]	4.6%
Guo et al (b)	VOT	203	202	0.79 [0.21; 2.99]	5.6%
Total (95% CI)				**1.17 [0.71; 1.91]**	**51.3%**
Heterogeneity: $Tau^2 = 0.1756$; $Chi^2 = 11.95$, df = 8 (P = 0.15); $I^2 = 33\%$ [0%; 69%]					
country_income_level = Lower middle					
Yoeli et al	Feature-phone	569	535	0.16 [0.08; 0.32]	8.5%
Velen et al	Digital pillbox	124	126	4.11 [0.18; 92.16]	1.8%
Jerene et al - Ukraine	Digital pillbox	1526	1589	1.96 [1.25; 3.08]	9.5%
Jerene et al - Philippines	Digital pillbox or 99DOTS	4604	4188	1.56 [0.81; 3.01]	8.6%
Jerene et al - Tanzania	Digital pillbox or 99DOTS	3546	3656	2.34 [0.72; 7.61]	6.2%
Mohammed et al	SMS	1110	1097	0.92 [0.54; 1.56]	9.2%
Farooqi et al	SMS	74	74	1.00 [0.06; 16.29]	2.1%
Total (95% CI)				**1.07 [0.47; 2.41]**	**46.0%**
Heterogeneity: $Tau^2 = 0.8431$; $Chi^2 = 39.87$, df = 6 (P < 0.01); $I^2 = 85\%$ [71%; 92%]					
**Total (95% CI)**				**1.05 [0.66; 1.65]**	**100.0%**
Heterogeneity: $Tau^2 = 0.5200$; $Chi^2 = 52.76$, df = 16 (P < 0.01); $I^2 = 70\%$ [50%; 82%]					
Test for subgroup differences: $Chi^2 = 0.46$, df = 2 (P = 0.79)					

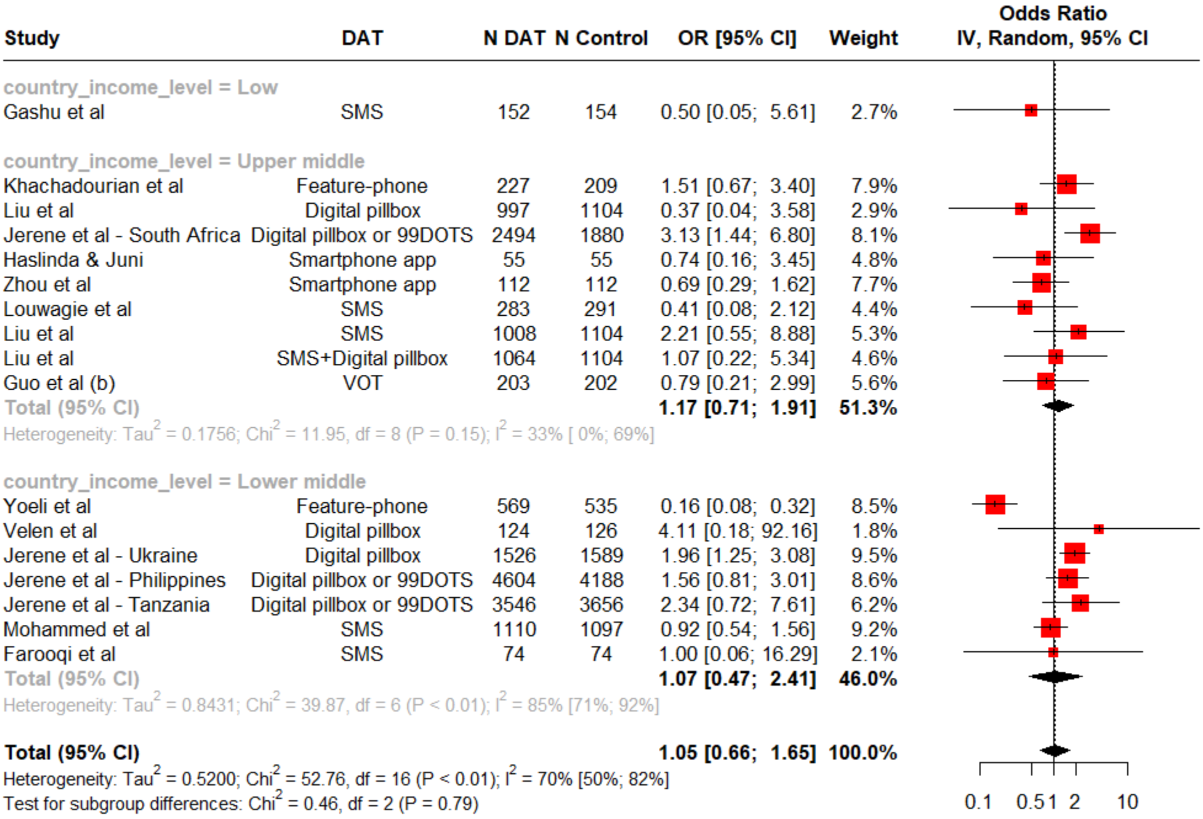

Figure S36a Forest plot showing treatment failure with DATs compared to standard of care stratified by country income level (RCTs)

Study	DAT	N DAT	N Control	OR [95% CI]	Weight
country_income_level = Lower middle					
Thekkur et al	99DOTS	870	961	4.43 [0.20; 98.29]	0.0%
Chen et al	99DOTS	8322	7722	0.67 [0.42; 1.08]	0.3%
Das Gupta et al	Feature-phone	111	111	1.00 [0.06; 16.19]	0.0%
Das Gupta et al	Feature-phone	111	111	0.50 [0.02; 14.99]	0.0%
Park et al	Digital pillbox	206	141	0.11 [0.01; 2.25]	0.0%
Kurada et al	Digital pillbox	385	3019	0.62 [0.08; 4.68]	0.0%
Saha et al	Digital pillbox	200	200	0.59 [0.21; 1.65]	0.1%
Total (95% CI)				**0.66 [0.44; 0.99]**	**0.4%**
Heterogeneity: Tau^2 = 0; Chi^2 = 2.96, df = 6 (P = 0.81); I^2 = 0% [0%; 71%]					
country_income_level = Upper middle					
Wang et al	Digital pillbox	1047	763	1.00 [0.98; 1.03]	99.6%
Wu et al	Digital pillbox	90	88	1.97 [0.07; 59.37]	0.0%
Zhang et al	Smartphone app	1145	1576	2.09 [0.19; 23.14]	0.0%
Wu et al	Smartphone app	82	88	1.07 [0.02; 54.74]	0.0%
Total (95% CI)				**1.00 [0.98; 1.03]**	**99.6%**
Heterogeneity: Tau^2 = 0; Chi^2 = 0.52, df = 3 (P = 0.92); I^2 = 0% [0%; 85%]					
country_income_level = Low					
Hermans et al	SMS	183	302	4.21 [0.81; 21.95]	0.0%
**Total (95% CI)**				**1.00 [0.97; 1.02]**	**100.0%**

Heterogeneity: Tau^2 = 0; Chi^2 = 10.40, df = 11 (P = 0.49); I^2 = 0% [0%; 58%]
Test for subgroup differences: Chi^2 = 6.93, df = 2 (P = 0.03)

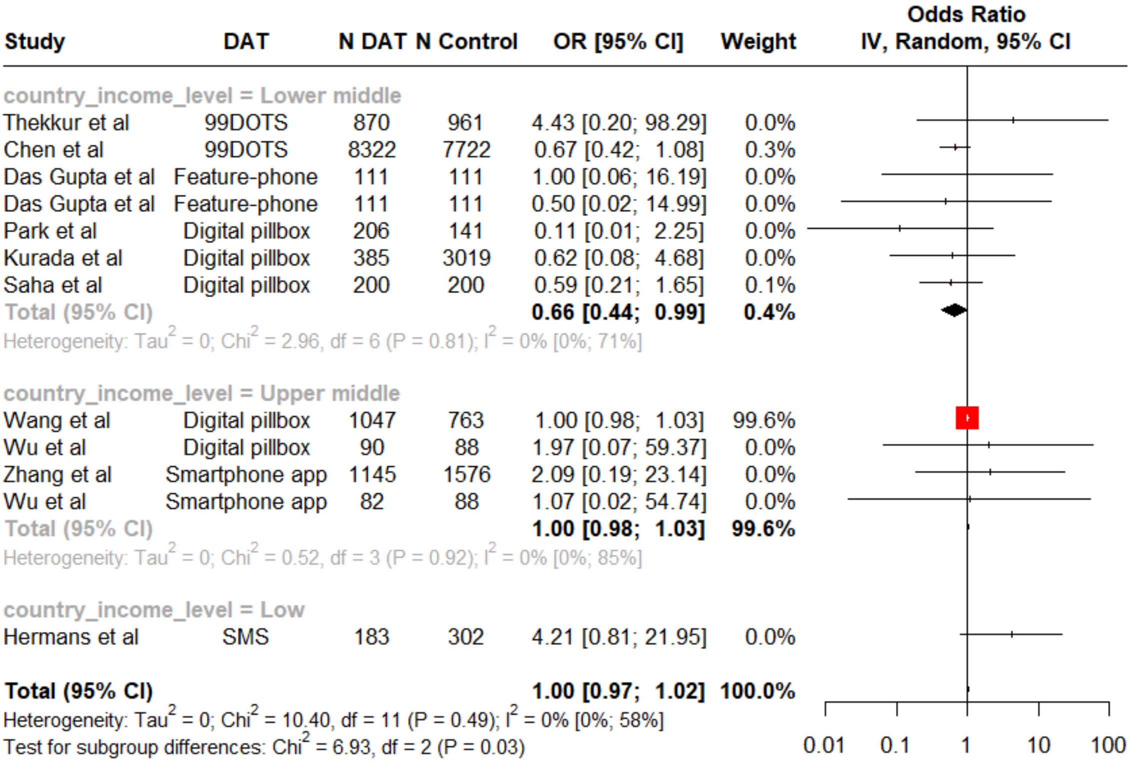

Figure S37b Forest plot showing treatment failure with DATs compared to standard of care stratified by country income level (observational studies)

Study	DAT	N DAT	N Control	OR [95% CI]	Weight
**country_income_level = Low**					
Manyazewal et al	Digital pillbox	57	57	1.00 [0.02; 51.28]	0.1%
Gashu et al	SMS	152	154	0.50 [0.05; 5.61]	0.3%
**Total (95% CI)**				**0.61 [0.08; 4.74]**	**0.4%**
Heterogeneity: $Tau^2 = 0$; $Chi^2 = 0.08$, df = 1 (P = 0.77); $I^2 = 0\%$					
**country_income_level = Lower middle**					
Jerene et al - Philippines	99DOTS	2368	4188	1.74 [1.03; 2.94]	6.3%
Jerene et al - Tanzania	99DOTS	1768	3656	1.99 [1.29; 3.07]	9.4%
Yoeli et al	Feature-phone	569	535	0.56 [0.24; 1.28]	2.5%
Velen et al	Digital pillbox	124	126	6.36 [0.75; 53.59]	0.4%
Jerene et al - Philippines	Digital pillbox	2236	4188	1.57 [0.92; 2.69]	6.1%
Jerene et al - Tanzania	Digital pillbox	1778	3656	1.57 [1.06; 2.33]	11.3%
Jerene et al - Ukraine	Digital pillbox	1526	1589	1.26 [0.99; 1.60]	30.3%
Bediang et al	SMS	137	142	0.34 [0.07; 1.69]	0.7%
Mohammed et al	SMS	1110	1097	2.02 [1.15; 3.52]	5.6%
**Total (95% CI)**				**1.48 [1.15; 1.91]**	**72.6%**
Heterogeneity: $Tau^2 = 0.0619$; $Chi^2 = 15.53$, df = 8 (P = 0.05); $I^2 = 48\%$ [0%; 76%]					
**country_income_level = Upper middle**					
Jerene et al - South Africa	99DOTS	1213	1880	1.05 [0.66; 1.68]	8.0%
Khachadourian et al	Feature-phone	227	209	0.92 [0.18; 4.61]	0.7%
Liu et al	Digital pillbox	997	1104	1.51 [0.52; 4.36]	1.5%
Ratchakit-Nedsuwan et al	Digital pillbox	50	50	3.24 [0.32; 32.57]	0.3%
Jerene et al - South Africa	Digital pillbox	1281	1880	1.00 [0.63; 1.58]	8.3%
Haslinda & Juni	Smartphone app	55	55	1.73 [0.39; 7.64]	0.8%
Zhou et al	Smartphone app	112	112	0.74 [0.25; 2.19]	1.5%
Iribarren et al	Smartphone app	21	21	0.49 [0.02; 15.37]	0.1%
Louwagie et al	SMS	283	291	1.42 [0.64; 3.16]	2.8%
Liu et al	SMS	1008	1104	1.12 [0.36; 3.48]	1.4%
Liu et al	SMS+Digital pillbox	1064	1104	1.27 [0.42; 3.79]	1.5%
Guo et al (b)	VOT	203	202	0.67 [0.01; 33.71]	0.1%
**Total (95% CI)**				**1.10 [0.86; 1.42]**	**26.9%**
Heterogeneity: $Tau^2 = 0$; $Chi^2 = 3.07$, df = 11 (P = 0.99); $I^2 = 0\%$ [0%; 58%]					
**country_income_level = High**					
Story et al	VOT	112	114	1.02 [0.02; 51.75]	0.1%
**Total (95% CI)**				**1.34 [1.18; 1.53]**	**100.0%**

Heterogeneity: $Tau^2 = 0$; $Chi^2 = 22.56$, df = 23 (P = 0.49); $I^2 = 0\%$ [0%; 45%]
Test for subgroup differences: $Chi^2 = 3.10$, df = 3 (P = 0.38)

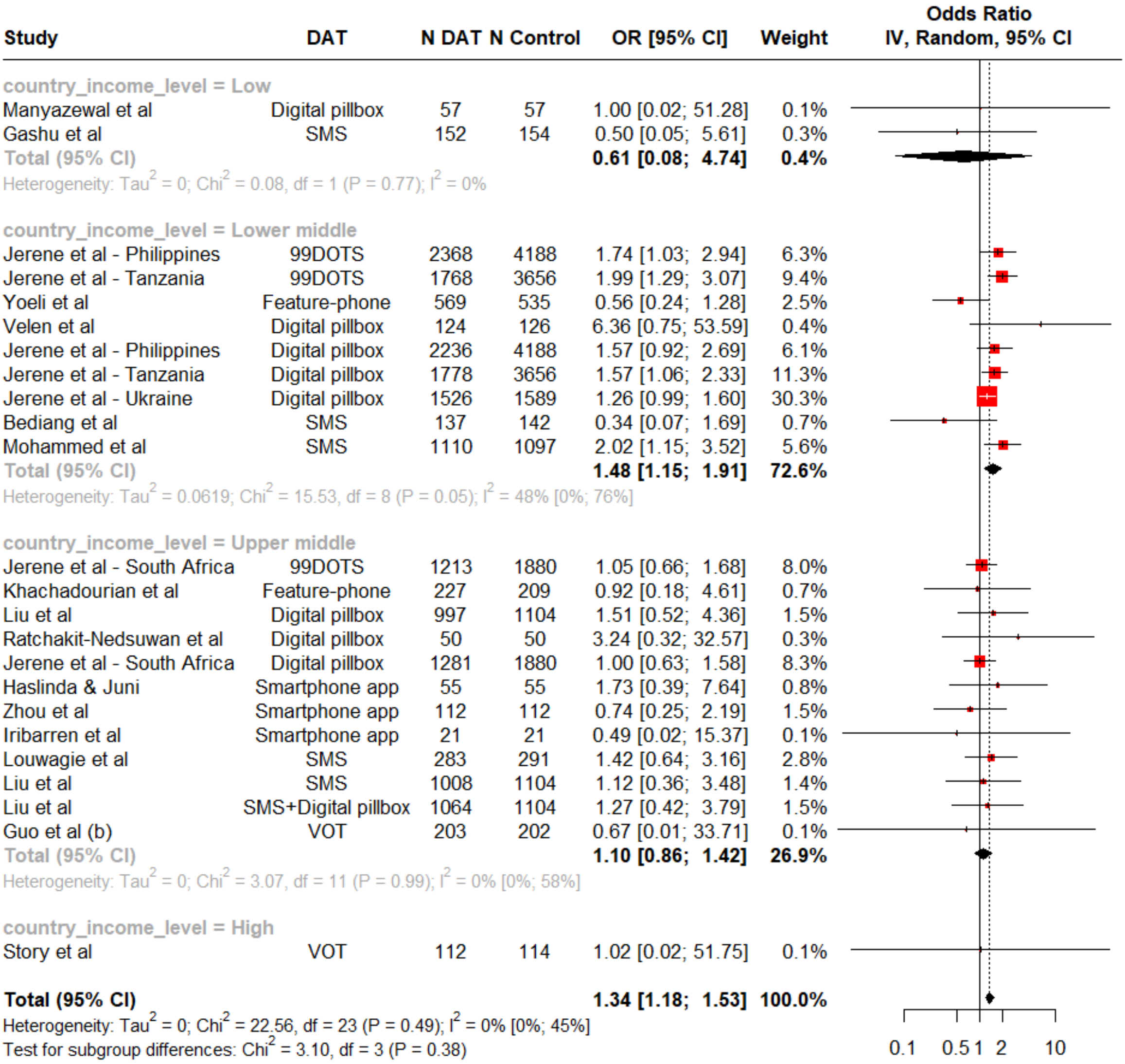

Figure S38a Forest plot showing death during treatment with DATs compared to standard of care stratified by country income level (RCTs)

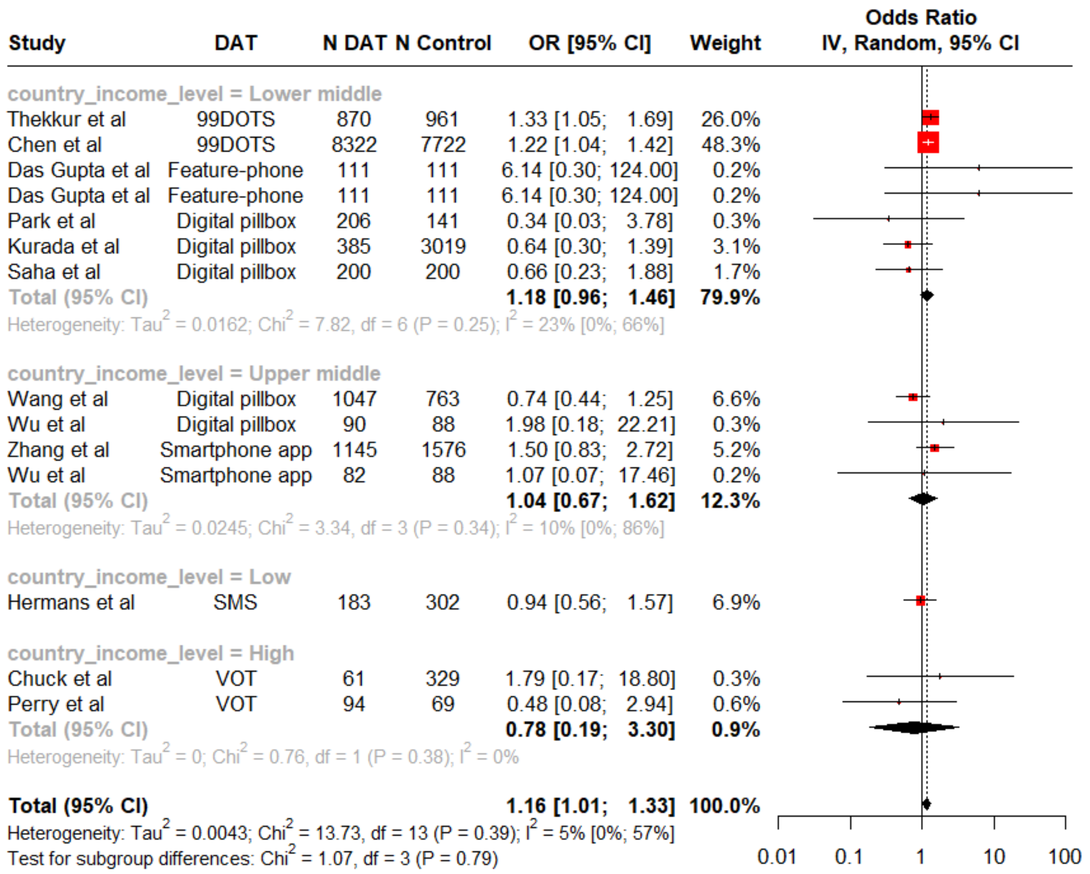

Figure S39b Forest plot showing death during treatment with DATs compared to standard of care stratified by country income level (observational studies)

S1 Meta-regression (country income level)

Digital pillboxes

```
Mixed-Effects Model (k = 16; tau^2 estimator: DL)

tau^2 (estimated amount of residual heterogeneity):     0.0430 (SE = 0.048
3)
tau (square root of estimated tau^2 value):             0.2074
I^2 (residual heterogeneity / unaccounted variability): 71.97%
H^2 (unaccounted variability / sampling variability):   3.57
R^2 (amount of heterogeneity accounted for):            0.00%

Test for Residual Heterogeneity:
QE(df = 14) = 49.9519, p-val < .0001

Test of Moderators (coefficient 2):
QM(df = 1) = 0.0539, p-val = 0.8165

Model Results:

                estimate      se     zval    pval    ci.lb   ci.ub
intrcpt           0.0802  0.1209   0.6635  0.5070  -0.1567  0.3172
CILUMIC or HIC   -0.0380  0.1637  -0.2321  0.8165  -0.3587  0.2828
```

SMS

```
Mixed-Effects Model (k = 9; tau^2 estimator: DL)

tau^2 (estimated amount of residual heterogeneity):     0.0894 (SE = 0.098
8)
tau (square root of estimated tau^2 value):             0.2990
I^2 (residual heterogeneity / unaccounted variability): 53.84%
H^2 (unaccounted variability / sampling variability):   2.17
R^2 (amount of heterogeneity accounted for):            0.00%

Test for Residual Heterogeneity:
QE(df = 7) = 15.1661, p-val = 0.0339

Test of Moderators (coefficient 2):
QM(df = 1) = 0.5816, p-val = 0.4457

Model Results:

                estimate      se    zval    pval    ci.lb   ci.ub
intrcpt           0.1033  0.1840  0.5615  0.5744  -0.2573  0.4639
CILUMIC or HIC    0.2256  0.2958  0.7626  0.4457  -0.3541  0.8052

---
```

S2 Meta-regression (Study design)

Digital pillbox

```
Mixed-Effects Model (k = 16; tau^2 estimator: DL)

tau^2 (estimated amount of residual heterogeneity):     0.0794 (SE = 0.059
9)
tau (square root of estimated tau^2 value):             0.2818
I^2 (residual heterogeneity / unaccounted variability): 65.85%
H^2 (unaccounted variability / sampling variability):   2.93
R^2 (amount of heterogeneity accounted for):            0.00%

Test for Residual Heterogeneity:
QE(df = 14) = 40.9910, p-val = 0.0002

Test of Moderators (coefficient 2):
QM(df = 1) = 0.4467, p-val = 0.5039

Model Results:

                          estimate      se     zval    pval    ci.lb   ci.u
b
intrcpt                     0.1677  0.1738   0.9647  0.3347  -0.1730  0.508
4
Study_design_metaregRCT    -0.1411  0.2111  -0.6684  0.5039  -0.5549  0.272
7
```

SMS

```
Mixed-Effects Model (k = 9; tau^2 estimator: DL)

tau^2 (estimated amount of residual heterogeneity):     0.0680 (SE = 0.085
4)
tau (square root of estimated tau^2 value):             0.2609
I^2 (residual heterogeneity / unaccounted variability): 47.87%
H^2 (unaccounted variability / sampling variability):   1.92
R^2 (amount of heterogeneity accounted for):            0.00%

Test for Residual Heterogeneity:
QE(df = 7) = 13.4273, p-val = 0.0624

Test of Moderators (coefficient 2):
QM(df = 1) = 1.5754, p-val = 0.2094

Model Results:

                         estimate      se     zval    pval    ci.lb   ci.u
b
intrcpt                   -0.2102  0.3337  -0.6301  0.5286  -0.8642  0.443
7
Study_design_metaregRCT    0.4569  0.3640   1.2551  0.2094  -0.2566  1.170
4

---
```

Evidence on adherence to treatment

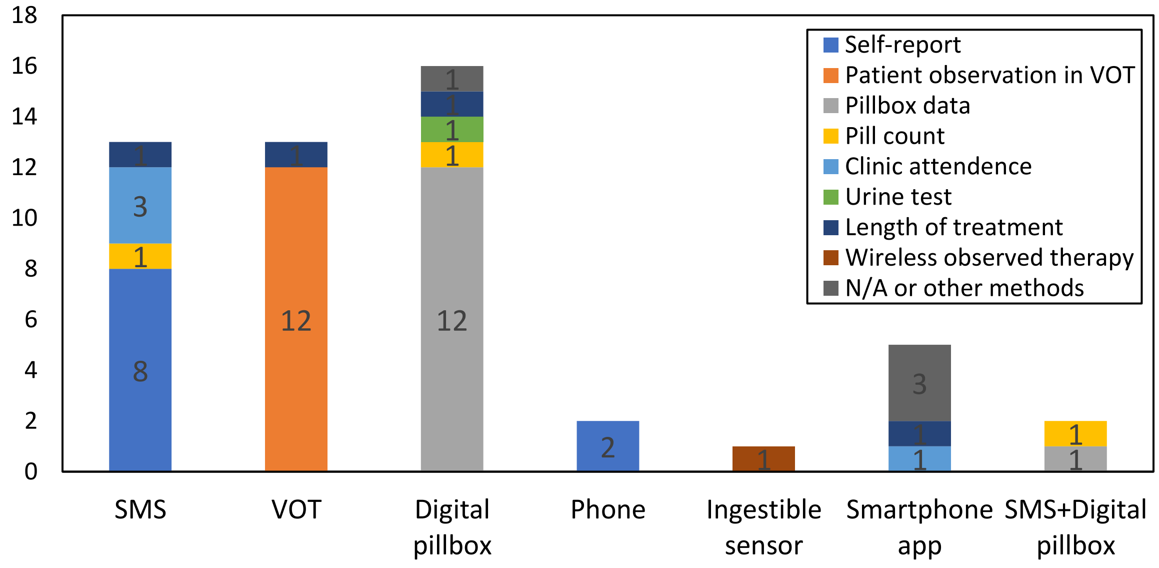

Figure S40 Measure of assessment of adherence to TB treatment stratified by DAT-type

Table S20 Qualitative summary of measures of adherence and estimated adherence in individual studies

	Study	Adherence assessed	Measure of assessment	Duration of adherence	Method of assessment	Unit	DAT	Standard care	Reported adherence
SMS- based intervention	Bediang et al	Optimal	Self-report	30 days	A 0 to 100% scale (Visual analog scale) at 2, 5 and 6 months)	P	Mean (SD) 99.7% (1.8)	Mean (SD) 99.5% (1.7)	MD 0.2 [−0.4, 0.7]
	Dewi et al	Optimal	Length of treatment	56 days	Adherence is identified if the length of treatment is 56 days	P	-	-	aOR 10.73 [3.64, 31.66][a]
	Fang et al	Suboptimal	Self-report	2 months Intensive phase	A questionnaire to assess the number of patients who missed a dose	P	30/160	87/190	p <0.001
	Gashu et al	Optimal	Self-report	4 months Continuation phase	11-item Adherence to Refill and Medication Scale (ARMS)	P	-	-	aRR 1.63 [1.09, 2.45][b]
	Hirsch-Moverman et al	Optimal	Self-report	30 days	Monthly follow up interviews about drug intake (cut-off point: 100%)	P	89.1%	79.5%	OR 2.10 [1.15, 3.82]
	Kibu et al	Optimal	Self-report -one-way SMS	Assessment after 3 months (interventio n)	A composite score for adherence was derived from: • visual analogue scale (VAS) and • Center for Adherence Support Evaluation (CASE) adherence index.	P	13/23	16/20	RR 0.46 [0.17, 1.24]
		Optimal	Self-report -two-way SMS			P	12/24	16/20	RR 0.4 [0.15, 1.05]
	Kumboyono	Optimal	Self-report	Not clear	observation sheet including three subjects. - the number of drugs consumed, - the type of drugs, - the time of drug consumption. patients who satisfy all three subjects are considered adherent	P	42/45	36/45	p= 0.059
	Liu et al (2015)	Suboptimal	Pill count, missing SMS Or medication monitor data	1 month	At each monthly follow-up visit missed doses were defined as the larger of (1) missed doses based on pill count or (2) missed doses from missing SMS reply (in the text messaging only arm) or (3) missed doses from failure to open the medication monitor box (in the combined intervention arm)				
					% patient-months with at least 3/15 doses missed	P	27.3%	29.9%	aMR 0.94 [0.71, 1.24][c]

					% patient-months with at least 7/15 doses missed	P	17.8%	18.9%	aMR 0.96 [0.67, 1.38][c]
					% total doses missed	P	20.7%	22.6%	aMR 0.94 [0.70, 1.26][c]
					% of patients with at least 10% of total doses missed	P	54.7%	57.4%	aMR 0.97 [0.77, 1.23][c]
					% patient-months with at least 3/15 doses missed (using pill count only)	P	3.8%	9.2%	aMR 0.39 [0.18, 0.83][c]
	Louwagie et al	Suboptimal	Self-report	4 days (assuming)	Modified versions of the AIDS Clinical Trials Group Adherence Questionnaire (assessed at 3 and 6 months) using an adherence index calculated by the formula (using the 4-day recall):<br>[total number of doses taken/ total number of doses prescribed] ×100;<br>(Cut-off point: 95%)	P	-	-	at 6 months:<br>OR 0.89 [0.26, 3.07]
	Mohammed et al	Optimal	Self-report validated by random samples using IsoScreen tests	Last 24 hours	A question: whether the patient took medication in the last 24 hours during unannounced visits during treatment. *Random sample of self-reported adherence was compared to the results of IsoScreen tests during the same study visit. IsoScreen tests indicated that 17% of those who said they had taken their drugs in the past 24 hours had not, indicating over-reporting.*	P	-	-	p= 0.772[d]
	Nguyen et al	Optimal	Timely attendance to clinic visits	6 months	On-time visit to the TB unit for sputum smear test (1st visit)	P	122/136	225/270	Calculated<br>OR 1.74 [0.92, 3.30]
					On-time visit to the TB unit for sputum smear test (2nd visit)	P	78/93	205/261	Calculated<br>OR 1.42 [0.76, 2.66]
					On-time visit to the TB unit for sputum smear test (3rd visit)	P	47/54	146/202	Calculated<br>OR [1.10, 6.04]
	Owiti et al	Optimal	Attendance to clinic visits	N/A	Adherence to scheduled clinic visits	P	101/150	16/37	RR 1.6 [1.06, 2.29]
	Peng et al	Optimal	Timely attendance to clinic visits	N/A	Timely attendance to clinic appointment for follow up examination	P	98.6%	92.5%	p<0.05
Vide	Bachina	Optimal	Patient's observation in VOT	6 months	Verified adherence (%)<br>[doses observed/ total number of prescribed doses]	D	81% (17.4)	54.5% (10.9)	-

	Burzynski et al	Optimal	Patient's observation in VOT	4 weeks	[Doses staff observed patients completely ingest/total doses]	D	2914/3244	2800/3190	89.8% [87.5, 92.1] vs 87.2% [84.6, 89.9] [k]
	Chen et al	Optimal	Patient's observation in VOT	9 months	[observed doses/ the total doses prescribed]	D	66.60%	61.42%	p = 0.001
	Doltu et al	Optimal	Patient's observation in VOT	3 months	[doses-days taken/ prescribed doses] Cut-off 80%	P	75/83	16/86	p<0.001
	Garfein et al	Optimal	Patient's observation in VOT#	6 months	the fraction of expected doses observed (FEDO) [the number of observed doses/ the sum of observed doses, missed doses, and self-administered doses]	D	93.0% (IQR 83.4%–97.1%)	66.4% (IQR 55.1%–89.3%)	Median FEDO
	Guo et al (a)	Optimal	Patient's observation in VOT	6 months	[The number of observed doses/ the total number of doses expected to be taken] § cut-off point 95%	P	63.0%	4.4%	p <0.001 148/ 235 vs 7/ 158
		Optimal			medication not discontinued for >= 3days	P	230/235	147/158	Calculated OR 3.44 [1.17, 10.11]
		Suboptimal			<100% adherence	P	21/235	41/158	Calculated OR 0.28 [0.16, 0.50]
	Lippincott	Optimal	Patient's observation in VOT	Overall	[the proportion of taken doses-days/ prescribed doses] Prescribed doses that could not be confirmed as ingested were recorded to be 'missed' or 'self-administered' based on chart documentation.	D	0.86 [0.7, 0.98]	0.59 [0.55, 0.64]	-
				Pre-covid			0.98 [0.78, 0.99]	0.58 [0.53, 0.61]	-
				After covid			0.8 [0.6, 0.93]	0.62 [0.55, 0.66]	-
		Suboptimal		Overall	Missed doses	D	0.05 [0, 0.16]	0.01 [0, 0.05]	-
				Pre-covid			0.02 [0, 0.17]	0.02 [0, 0.07]	-
				After covid			0.02 [0, 0.07]	0 [0, 0.02]	-
	Perry et al	Optimal	Patient's observation in VOT	≥ 2 months	[doses observed/ total number of doses]	D	Mean (SD) 68.4 (10.6)	Mean (SD) 53.9 (25.6)	p<0.001
					[doses that were observed/ total number of doses] §§	D	Mean (SD) 90 (9.9)	Mean (SD) 98.7 (3.1)	p<0.001
					[doses that were observed/ total number of doses] §§§	D	Mean (SD) 95.9 (5.9)	-	-
		Suboptimal			Proportion prescribed doses 'missed', %	D	3.4%	5.5%	p <0.001
	Ravenscroft et al	Suboptimal	Patient's observation in VOT	2 weeks	the number of days over each 2-week period (10 working days, excluding weekends and public holidays) that a	D	1.29 days per 2-week period	5.29 days per 2-week period	(Reduction per 2 weeks period) MD 4 days, [3.35, 4.67 days]

					patient was not observed adhering to their medication §§§§				
		Optimal		6 months	successful completion of scheduled treatment observations<br>Cut-off point 80%	P	74.5%	19.5%	OR 12.8 [10.06, 14.74]
	Salcedo et al	Optimal	Length of treatment	continuation phase	Number of days on treatment for TB	P	Mean (SD)<br>252.5 (69.4)	Mean (SD)<br>247.1 (89.07)	p=0.729
	Siddiqui	Optimal	Patient's observation in VOT	N/A	average adherence rate = proportion of observations that demonstrated successful receipt of TB treatment	D	2126/2303	2799/2879	p<0.05
	Story et al	Optimal	Patient's observation in VOT ##	2 months following randomizati on	successful completion of scheduled treatment observations<br>cut-off point 80%	P	78/112	35/114	aOR 5.48 [3.10, 9.68][e]
		Optimal		2 months	Proportion of total doses observed over 2 months	D	5091/ 6474	1774/3922	Calculated<br>OR 4.46 [4.09, 4.86]
		Optimal		Up to 6 months	Proportion of scheduled observations successfully completed over the full follow-up period	D	12422/16230	3884/9882	p<0.0001
	Wade et al	Suboptimal	Patient's observation in VOT	Not clear	Observation days lost	Not clear	5.3	3.3	MD 2 [-5.4, 4.8]
**Digital pillbox**	Acosta et al	Suboptimal	Pillbox data	4 months	proportion of patients who missed at least one of the total doses scheduled §§§§§<br>Cut-off point 0%	P	11/49	15/53	RR 0.79 [0.4, 1.56]<br>p=0.498
		Suboptimal			proportion of patients who missed 10% of doses<br>cut off point 10%	P	1/49	7/53	RR 0.15 [0.02, 1.21]<br>p=0.0613
	Charalambous	Suboptimal	Pillbox data	168 days	Poor adherence §§§§§§<br>Cut-off point <80%	P	18.8%	48.7%	aRR 0.4 [0.30, 0.54] [f]
		Optimal		94 weeks	overall adherence<br>1839 episodes amongst participants required a phone call and 1433 (78%) were successful.	D	88.5%	69.70%	aMD 17.20% [13.7, 20.8] [f]
	Guo et al	Optimal	Pillbox data	Weekly for 6 months	a percentage of the patient's prescribed number of medications per week. (village)	P	47% (20.5)	26.7 (21.1)	
	Liu et al 2015	Suboptimal	Pill count or pillbox data	1 month	At each monthly follow-up visit, missed doses were defined as the larger of<br>(1) missed doses based on pill count or				

					(2) missed doses from failure to open the medication monitor box (in the other two intervention arms)				
					percentage of patient-months with at least 3/15 doses missed	P	17.0%	29.9%	aMR 0.58 [0.42; 0.79][c]
					Percentage of months with at least 7/15 doses missed	P	11.1%	18.9%	aMR 0.6 [0.40, 0.89] [c]
					Percentage of total doses missed	P	13.9%	22.6%	aMR 0.62 [0.46, 0.84] [c]
					At least 10% of total doses missed	P	3.7%	57.4%	aMR 0.68 [0.52, 0.89] [c]
					Percentage of patient-months with at least 3/15 doses missed (using pill count only)	P	5.5%	9.2%	aMR 0.58 [0.35, 0.96] [c]
	Liu et al 2023	Suboptimal	Pillbox data	6 months	Months in which patient missed >20% of doses per person per months of treatment	P	0.9/6	2.7/6	aMD 0.36 [0.27, 0.50] [g]
					Doses missed per person per doses expected	P	16/160	42/160	aMD 0.43 [0.34, 0.53] [g]
			Clinic visits		Late or missed clinic visits per person per scheduled visits	P	2.5/5	2.6/5	aMD 0.97[0.87, 1.08] [g]
	Manyazewal et al	Optimal	Pillbox data; assuming all take-home doses in the control group were ingested ###	Four 15-day appointments (intensive phase)	the proportion of participants who achieved adherence threshold ≥ 90%	P	57/57	55/57	p=0.496
					the proportion of participants who achieved adherence threshold ≥ 80%	P	57/57	57/57	p=0.189
					Geometric mean of doses taken (individual-level % adherence averaged over the 2-month intensive phase measured)	P	99.01%	98.97%	aMR 1.00 [0.99, 1.01]
			Pillbox data; assuming all take-home doses in the control group were NOT ingested ###	Four 15-day appointments (intensive phase)	Geometric mean of doses taken (individual-level % adherence averaged over the 2-month intensive phase measured)	D	3386/3420	2658/3420	MR 1.27 [1.33, 1.43]
		Suboptimal	IsoScreenTM urine isoniazid test	Four 15-day appointments	Non-Adherence (=negative urine isoniazid test defined as not having taken a dose within last 24-36h at AT LEAST 1 of the 4, 15-day appointments Missed samples do not count.	P	2/57	11/57	RR 5.5 [1.28, 23.71]
			self-report	At the end of the intensive phase	Questionnaire about often forgetting taking the medication	P	0.019	0.089	p=0.21

	Moulding and Caymittes	Optimal	Pillbox data	9 months	Patients who took at least 80% of their doses	P	50/58	38/48	Calculated OR 1.64 [0.59, 4.57]
				9 months	Patients who took at least 90% of their doses	P	34/58	28/48	Calculated OR 1.01 [0.47, 2.20]
	Park et al	Optimal	Pillbox data	1 month (for 6 months)	monthly drug adherence rate for TB medication	P	81.35 SD= 6.8	80.77 SD= 9.2	p = 0.525
	Pima et al	Optimal	self-report	6 months	Mean adherence (no further details)	Not clear	99%	99%	p=0.86
			Pill count	6 months	Mean adherence (no further details)	Not clear	94%	83%	p<0.01
	Ratchakit-Nedsuwan et al	Optimal	Pillbox data	6 months or until completion	Proportion of adherence in the 2 groups >=80%	P	39/40	37/40	HR 3.2 [0.2, 170.2]
					Proportion of adherence in the 2 groups >=90%	P	37/40	29/40	HR 4.7 [1.1, 28.0]
					Proportion of adherence in the 2 groups >=100%	P	14/40	4/40	HR 4.8 [1.3, 22.1]
	Saha	Optimal	Not clear; assessed during follow up visits and further validated using urine test for 20% of patients	6 months	Calculated for each patient [the number of days for which the prescribed number of doses were taken/ total number of days] cut off point 80%	P	99% [98.3; 99.7]	90% [89.3; 90.7]	-
			Not clear; assessed during follow up visits	More than 8 weeks	Point adherence for those who were on treatment after the study duration and had completed more than 8 weeks of treatment	P	98.7% [97.9; 99.5]	95.2% [94.5; 95.9]	-
			urine test ####	During intensive phase	Positive rifampicin (>100micrograms)-ROUND 1	P	26/29	24/30	Calculated OR 2.17 [0.49, 9.46]
				Within 1 month of round 1	Positive rifampicin (>100micrograms)-ROUND 2	P	25/28	20/25	Calculated OR 2.08 [0.44, 9.79]
				Within 1 month of round 2	Positive rifampicin (>100micrograms)-ROUND 3	P	16/19	13/17	Calculated OR 1.64 [0.31, 8.68]
	Velen	Suboptimal	Pillbox data	1 month	percentage of patient-months with at least 6/30 doses were missed	P	25.8% [19.2; 31.8]	35.8% [29.8; 41.2]	MR 0.72 [0.67, 0.77]
					percentage of patient-months with at least 14/30 doses were missed	P	12.4% [7.6; 17.0]	20.2% [15.1; 25.0]	MR 0.61 [0.54, 0.68]

					the proportion of total expected doses missed over the treatment period	P	15.8% [12.9; 18.7]	21.2% [18.3; 23.9]	MR 0.75 [0.68, 0.80]
		Optimal			Patient months with at least 24/30 doses taken	P	-	-	aOR 2.2 [1.3, 2.9] [h]
					Patient months with at least 16/30 doses taken	P	-	-	aOR 1.9 [1.01, 3.4] [h]
	Wei et al	Suboptimal	Pillbox data	1 month	Patient months with at least 20% missed planned doses	P	84/854	287/798	aRR 0.34 [0.23, 0.42] [l]
				6 months	Percentage of all missed doses	D	1976/25594	6937/23872	aRR 0.25 [0.18, 0.35] [l]
				1 month	missing ≥10% of all planned doses §§§§§§§	P	32/142	72/134	aRR 0.44 [0.26, 0.68] [l]
	Wang	Optimal	Pillbox data through the background management system	6 months	Medication adherence rate = actual medication doses/number of prescribed doses * 100%,	D	95.7% (85.5, 98.7)	83.8 (47.2, 95.7)	
					High medication adherence, cut-off point 90%	P	64.5%	39.60%	OR=2.73 [1.84,4.04]
		Suboptimal			Number of missed doses	D	3 (1, 9)	9 (3, 25)	
	Wu	Optimal	extracted from the mHealth reminder system	Not clear	Median treatment days	P	280, IQR 198-365	360, IQR 283-369	
**Feature phone-based**	Khachadourian et al	Optimal	Self-report	Not clear	% of patients who received their drugs as scheduled	P	187/187	173/198	Calculated OR 55.12 [3.33, 912.22]
		Optimal	self-report (family member)	Not clear	% of patients who adhered to treatment as reported by family supporters	P	185/187	158/198	Calculated OR 23.42 [5.57, 98.44]
	Santra et al	Optimal	Self- report	Previous 15 days (Assessed at baseline and after 90 days of the intervention)	4-item Morisky-Green-Levine Adherence Scale (MGLS) Nonadherence to anti-tuberculosis medications was defined as a score <4 as per the MGLS	P	Baseline 94/110 Endline 106/110	Baseline 94/110 Endline 97/110	- Calculated OR 3.55 [1.12, 11.26]
**Ingestibl**	Brown et al	Optimal	Data from the WOT system	All observation days	percentage of IS-Rifamate ingestions detected by WOT when administered under direct observation (proportion confirmed)	D	92.9% [88.7; 96]	63.1% [58.3; 66.9]	OR 7.69 [4.51, 14.48]

					daily adherence ≥ 90% in individual participants over the entire period of follow-up (≥ 90% confirmed doses)	P	78%	0%	MD 0.78 [0.65, 0.87]
**Smartphone apps**	Haslinda & Juni	Optimal	gathered from the record book in health clinics	6 months	Not clear	P	45/55	38/55	aOR 2.16 [0.71, 6.58] [i]
	Wang et al	Optimal	through the background management system	6 months	Medication adherence rate = actual medication doses/number of prescribed doses * 100%,	D	95.3% (85.0, 98.5)	83.8 (47.2, 95.7)	
					High medication adherence, cut-off point 90%	P	64.7%	39.60%	OR 2.92 [2.18, 3.91]
		Suboptimal			Number of missed doses	D	3 (1, 10)	9 (3, 25)	
	Wu et al	Optimal	extracted from the mHealth reminder system	Not clear	Median treatment days	P	296, IQR 204-365	360, IQR 283-369	
	Zhang et al	Optimal	N/A	6 months (done monthly)	medication adherence defined as 80% or more of prescribed monthly doses taken	D	965/1145	1266/1576	aOR 1.33 [1.08, 1.63] [j]
	Zhou et al	Suboptimal	N/A	2 years	poor adherence over the period of 2 years	P	6/88	37/79	Calculated OR 0.08 [0.03, 0.21]
	Liu et al 2015	Suboptimal	Pill count or pillbox data	1 month	At each monthly follow-up visit, missed doses were defined as the larger of (1) missed doses based on pill count or (2) missed doses from failure to open the medication monitor box (in the other two intervention arms)				
					percentage of patient-months with at least 3/15 doses missed	P	13.9%	29.9%	aMR 0.49 [0.27, 0.88][c]
					Percentage of months with at least 7/15 doses missed	P	9.4%	18.9%	aMR 0.52 [0.28; 0.97][c]
					Percentage of total doses missed	P	11.4%	22.6%	aMR 0.53 [0.29; 0.95][c]
					At least 10% of total doses missed	P	31.0%	57.4%	aMR 0.56 [0.33, 0.97] [c]
					Percentage of patient-months with at least 3/15 doses missed (using pill count only)	P	6.4%	9.2%	aMR 0.67 [0.31, 1.47][c]
	Musiimenta et al	Optimal	Pillbox data	6 months	the total number of device openings divided by the total number of study follow-up days	P	96.1%, (IQR 84.8–98.0)	92.2% (IQR 56.3–97.8)	
						P	92.5% (IQR 80.6–96.3)	92.2% (IQR 56.3–97.8)	

P Patient; D Dose

a- Adjusted for age, sex, education, occupation, and income), accessibility (available mode of transportation and time to reach a health facility), and adverse drug reactions.

b- Adjusted for sex, literacy, residence, wealth index, health facility type, and randomisation.
c- Adjusted for individual-level variables of gender, age group, occupation (farmer or not), local resident or not, distance to nearest TB clinic, education level, income category, and smear result at start of treatment, and for the cluster-level variable of pre-randomisation stratum (rural/urban).
d- Controlling for the length of the regimen, days in the study, and days in the study-squared
e- Adjusted for time since start of treatment, age, sex, and treatment, current social risk factor (homelessness, imprisonment, drug use, alcohol problems, immigration concern), ever lost to follow-up, no recourse to public funds and mental health problems.
f- Adjusted for sex, age group, bacteriologically confirmed TB, HIV/ART status, ethnic group
g- Adjusted for age, sex, occupation, migrant status, distance to clinic, education level, household expenditure, and smear result at treatment initiation, using the two stage approach.
h- Adjusted for education level and patient distance from healthcare facility.
i- Adjusted for sociodemographic variables (age, gender, marital status, employment status, income status, educational status, ethnicity, smoking status), type of PTB (smear+ or smear-)
j- Adjusted for age, gender, occupation, category of TB report and type of TB
k- Adjusted for fixed-effect explanatory variables representing DOT method at each dose, participant randomization group, crossover period, the dose outcome during each of the 2 preceding scheduled and observable doses (representing carryover effects), season (represented as calendar quarter), and the interaction between DOT method and season
l- Adjusted for county (stratum/centre), age, sex, job, marriage status, treatment month, treatment month

For this analysis, doses were only considered observed if all pills were taken. If no video was received or ingestion of fewer than all pills was observed, the dose was considered missed, as were self-administered doses. Because weekend doses are not ordinarily observed in DOT, they were excluded from this calculation.

only videos for which ingestion of all medicines was observed classified as successfully.

Missed doses were verified using pill count and discussion. Any participant in the intervention arm who delayed (>15 days) for a follow-up was considered non-adherent for each day the patient did not refill medications. Any patient who missed more than five tablets in any 15-day refill period was subject to reassignment to DOT throughout the remaining days of the intensive phase.

A 24-h recall of drug consumption was elicited, and those who had consumed the tablets were requested to give their urine samples. A total of 104 samples were collected in the intervention arm over three cycles. However, in the control arm, 108 samples were collected. However, the number of samples processed was less.

§ The numerator includes either directly observed or video-observed doses, excluding those obtained by self-report. The denominator includes the self-administered medications; it does not include periods when the treatment was suspended based on medical advice because of side effects.

§§ Calculated based on the assumption that patients take every dose that is not observed (including prescribed, dispensed, self-administered doses such as on weekends and holidays).

§§§ Calculated based on crediting videos that were submitted by rejected videos (such as in cases where the observer could not visualize the pill).

§§§§ For DOT patients, this was based on whether they electronically signed the tablet at their clinic to indicate their attendance. For VOT patients, this was based on whether they sent a video showing them taking their medication. Across the monitoring period, each patient contributed approximately eight 2-week periods.

§§§§§ A missed dose was considered when the patient did not attend the PHC and could not be reached by staff on the day of treatment in the control group, or when the pillbox was not opened on the day of treatment and the treatment monitor had excluded connectivity problems in the MERM group.

§§§§§§ % adherence was calculated as days monitor was opened (proxy for daily-dose taken)/ total expected treatment days. 4 from the MERM group were withdrawn: 2 withdrew voluntarily, 1 switched to a different treatment, and the TB programme staff withdrew 1 for suspected misuse of the pillbox.

§§§§§§§ The total % of planned doses missed during treatment outcome was recorded at the dose level (with 30 planned doses per patient treatment month) but analysed at the patient treatment month level with intervention effect estimates calculated as per the primary outcome approach.

Evidence on patient-reported outcomes

Table S21 Qualitative summary of patient-reported outcomes in DAT-groups and standard of care groups

	Study	Time of assessment	Measure of assessment	Method of assessment	DAT	Standard care	Comments
	**Satisfaction**						
SMS-based intervention	**Bediang et al**	At the end of month 6	Self-report	(i) general management of patients- (Likert scale)	99.5%	99.2%	-
		At the end of month 6	Self-report	(ii) support provided for adherence to drug prescriptions (Likert scale)	99.6%	99.1%	-
	Gashu et al	Phone call-based endline assessment	Self-report	Good provider-patient relationship rate between intervention and control groups (a 7-item provider–patient relationship questionnaire adapted from previous studies). The items include:<br>• patients' satisfaction with the care provided,<br>• patient's trust in care provider,<br>• frequency of phone call to the patient,<br>• the convenience of appointments,<br>• frequency of calls by the patient to care provider,<br>• patient feeling shame to question, and<br>• patients' perceived compassionate and caring provider.	73.3%<br>102/139	52.4%<br>75/143	AD 20.9% (95% lower confidence level 10.0)
Video-observed therapy	Chen et al	Assessed were subjects who completed or quit their 9-month isoniazid LTBI treatment regimen during January 2014 to December 2017	Self-report	The satisfaction questionnaire was composed of five elements. Satisfaction to each element was graded into a four-point scale.			
				time scheduling and	81.3%		p<0.001
				location arrangement.	96.7%		p=0.039
				efficacy of monitoring and managing adverse events	98.3		-
				ensuring treatment adherence; and	88.8%		p=0.027
				efficacy of alleviating privacy concerns	92.5%		P= 0.005
	Story et al	At 2 and 6 months	Self-report via telephone interviews	"How much do you agree or disagree with the following statement: "I am satisfied with the way my treatment is observed?" (Likert scale- 5 scales)	Satisfaction is presented as frequencies in a Likert scale		
	Ravenscroft et al	After 4 months	Self-report endline questionnaire	satisfaction of being in the treatment group (5-point scale)	4.9	4.2	aOR 3.29 [1.66; 4.92]
				Did the way (VDOT/DOT) help you not to miss doses?	93% (185/199)	87.3% (171/196)	p = 0.057

	Guo et al 2020 (b)		Self-report (Likert scale (yes-no-unsure))	Was the way (VDOT/DOT) convenient and comfortable?	96% (191/199)	56.7% (111/196)	p < 0.001
				If a further treatment needed, would you choose the original way or a new way?	96% (191/199)	63.3% (124/196)	p < 0.001
				Would you recommend it (VDOT/DOT) to 6 OTHER TB patients?	96% (191/199)	57.7% (113/196)	p < 0.001
**Digital pillboxes**	Manyazewal et al 2023		Self-report	Treatment Satisfaction Questionnaire for Medication (TSQM) for Effectiveness (satisfaction with treatment, satisfaction with symptom relief, satisfaction with time to start working) *	85.78 (1.19)	63.43 (1.23)	MR 1.35 [1.26; 1.45]
				TSQM for Convenience (treatment easy to use, easy planning of use, intake convenience)	85.41 (1.17)	48.18 (1.33)	MR 1.77 [1.63; 1.93]
				TSQM for Global satisfaction (confidence in benefits, balance between good and bad things, overall satisfaction)	90.19 (1.13)	67.11 (1.22)	MR 1.34 [1.26; 1.43]
	HRQoL						
**SMS**	Johnston et al	At study exit	Self-report	Score SF-12 physical	54.9 [47.6; 57.0]	54.1 [46.3; 57.1]	p= 0.823
				Score SF-12 mental	53.7 [48.6; 58.6]	53.5 [48.0; 57.7]	p= 0.471
**Video-observe**	Story et al	At 2 months	Self-report	time trade-off (TTO) derived from EQ5D-3L using UK value set	0.75 [0.66; 0.83]	0.76 [0.65; 0.86]	-
		At 6 months			0.73 [0.58; 0.88]	0.7 [0.51; 0.89]	-
		At 2 months		EQ-5D-3L visual analogue scale	0.71 [0.64; 0.79]	0.73 [0.63; 0.83]	-
		At 6 months			0.74 [0.63; 0.84]	0.74 [0.62; 0.86]	-
**Digital pillbox**	Manyazewal et al (b)	At the end of the intensive phase	Self-report	the EuroQoL 5-Dimension 5-Level**<br>EQ-5D-5L median (IQR) index value	1 [0.97; 1]	0.91 [0.89; 0.96]	-
				• mobility	51/52	35/57	
				• self-care	50/52	38/57	
				• ability to do usual activity	45/52	22/57	
				• pain/discomfort	46/52	20/57	
				• anxiety/ depression	44/52	25/57	
	Saha et al	at baseline and the first follow-up	Self-report	EuroQol's Crosswalk value sets for Thailand using the EQ5D5L profile**<br>EQ-5D-5L index value	0.626	0.666	-
**Feature-Phone**	Khachadourian et al	Follow-up	Self-report	EQ-5D index utility score<br>- TB Patient - Difference (estimate)	5.01 [-0.64; 10.66]	7.29 [1.77; 12.81]	-
				EQ-5D index utility score<br>- TB Patient - according to PP analysis	78.8 (25.9)	81.6 (24.5)	-
	**Stigma**						
**Fe at**		At the follow up	Self-report	Modified Van Rie scale (mean (SD)) ***	0.7 (3.1)	0.6 (2.7)	-

	Khachadourian et al			TB patient – Difference in stigma ****	-	-	MD 0.52 [−0.27; 1.31]
				TB family - Difference in stigma****	-	-	MD 0.26 [− 0.53; 1.05]

*Patient treatment satisfaction was assessed using the Treatment Satisfaction Questionnaire for Medication (TSQM v1.4©). TSQM© is a PRO instrument designed to evaluate treatment satisfaction with a wide variety of medications. It comprises 14 questions subdivided into four domains: Effectiveness, Convenience, Side Effects, and Global Satisfaction. Each of the four domains have at least three questions: Effectiveness (Qs 1–3), Side Effects (Qs 4–8), Convenience (Qs 9–11), and Global Satisfaction (Qs 12–14). Of the 14 questions; 13 were designed as a 5- or 7-point Likert scale to assess the level of satisfaction or dissatisfaction a participant had with the medication last used in the clinical trial. The sole remaining question contains a binary (yes/no) score. Each domain was computed independently by adding the TSQM items from each domain and the composite score transformed into a value ranging from 0 to 100, with a higher score indicating greater satisfaction. The questionnaire was administered by independent study staff using a paper format at the end of the two-month intensive treatment phase.

**Questionnaire (EQ-5D-5L) The tool measures health across 5 domains: mobility, self-care, ability to do usual activities, pain or discomfort, and anxiety or depression. Each domain has 5 levels of response—no problems, slight problems, moderate problems, severe problems, and extreme problems or inability—that provide a descriptive profile used to generate a health state utility value. Health state index scores generally range from less than 0 (where zero is the value of a health state equivalent to death; negative values representing values worse than death based on patient perception) to 1 (the value of full health), with higher scores indicating higher health utility states. Patient responses were converted into a health state, a 5-digit number generated by concatenating each of the 5 responses to provide a single index value score that determines the health state (e g, full health would be coded 11111). Health state index scores were calculated from individual health profiles using the Ethiopia value set

***Van Rie scale measures stigma of tuberculosis from the community perspectives. The questions in the scale were modified to measure the behaviour of family members toward the tuberculosis patient from patients’ perspective. Its score ranged from 0 to 27

****Change is the difference in the outcome between intervention and control group

References

[1] N. N. Linh *et al.*, "World Health Organization treatment outcome definitions for tuberculosis: 2021 update," *Eur Respir J*, vol. 58, no. 2, Aug. 2021, doi: 10.1183/13993003.00804-2021.

[2] "The Cochrane Collaboration tool for assessing risk of bias." Accessed: Sep. 08, 2023. [Online]. Available: https://handbook-5-1.cochrane.org/chapter_8

[3] A. O. A. Ali and M. H. Prins, "Mobile health to improve adherence to tuberculosis treatment in khartoum state, sudan," *J Public Health Afr*, vol. 10, no. 2, 2019, doi: 10.4081/jphia.2019.1101.

[4] G. Bediang, B. Stoll, N. Elia, J. L. Abena, and A. Geissbuhler, "SMS reminders to improve adherence and cure of tuberculosis patients in Cameroon (TB-SMS Cameroon): A randomised controlled trial," *BMC Public Health*, vol. 18, no. 1, May 2018, doi: 10.1186/s12889-018-5502-x.

[5] R. Belknap *et al.*, "Self-administered versus directly observed once-weekly isoniazid and rifapentine treatment of latent tuberculosis infection," *Ann Intern Med*, vol. 167, no. 10, pp. 689–697, Nov. 2017, doi: 10.7326/M17-1150.

[6] F. S. T. Dewi, S. Sudiya, Supriyati Supriyati, and A. Utarini, "Preparing Short Message Service Reminders to Improve Treatment Adherence among Tuberculosis Patients in Sleman District, Indonesia," *Indian J Community Med*, vol. 44, no. 2, pp. 81–87, Apr. 2019, doi: 10.4103/IJCM.IJCM_207_18.

[7] X. H. Fang *et al.*, "Effect of short message service on management of pulmonary tuberculosis patients in Anhui Province, China: A prospective, randomized, controlled study," *Medical Science Monitor*, vol. 23, pp. 2465–2469, May 2017, doi: 10.12659/MSM.904957.

[8] R. Farooqi, S. Ashraf, and M. Zaman, "THE ROLE OF MOBILE SMS-REMINDERS IN IMPROVING DRUGS COMPLIANCE IN PATIENTS RECEIVING ANTI-TB TREATMENT FROM DOTS PROGRAM," *Journal of Postgraduate Medical Institute*, 2017.

[9] K. D. Gashu, K. A. Gelaye, R. Lester, and B. Tilahun, "Effect of a phone reminder system on patient-centered tuberculosis treatment adherence among adults in northwest ethiopia: A randomised controlled trial," *BMJ Health Care Inform*, vol. 28, no. 1, Jun. 2021, doi: 10.1136/bmjhci-2020-100268.

[10] S. M. Hermans, S. Elbireer, H. Tibakabikoba, B. J. Hoefman, and Y. C. Manabe, "Text messaging to decrease tuberculosis treatment attrition in TB-HIV coinfection in Uganda," *Patient Prefer Adherence*, vol. 11, pp. 1479–1487, Aug. 2017, doi: 10.2147/PPA.S135540.

[11] Y. Hirsch-Moverman *et al.*, "Using mHealth for HIV/TB Treatment Support in Lesotho: Enhancing Patient–Provider Communication in the START Study," *J Acquir Immune Defic Syndr*, vol. 74, no. Suppl 1, p. S37, Jan. 2017, doi: 10.1097/QAI.0000000000001202.

[12] J. C. Johnston *et al.*, "The effect of text messaging on latent tuberculosis treatment adherence: A randomised controlled trial," *European Respiratory Journal*, vol. 51, no. 2, 2018, doi: 10.1183/13993003.01488-2017.

[13] O. D. Kibu, V. V. Siysi, S. E. Albert Legrand, E. Asangbeng Tanue, and D. S. Nsagha, "Treatment Adherence among HIV and TB Patients Using Single and Double Way Mobile Phone Text Messages: A Randomized Controlled Trial," *J Trop Med*, vol. 2022, 2022, doi: 10.1155/2022/2980141.

[14] Kumboyono, “Short message service as an alternative in the drug consumption evaluation of persons with tuberculosis in Malang, Indonesia,” *Japan Journal of Nursing Science*, vol. 14, no. 2, pp. 112–116, Apr. 2017, doi: 10.1111/jjns.12140.

[15] X. Liu *et al.*, “Effectiveness of Electronic Reminders to Improve Medication Adherence in Tuberculosis Patients: A Cluster-Randomised Trial,” *PLoS Med*, vol. 12, no. 9, Sep. 2015, doi: 10.1371/journal.pmed.1001876.

[16] G. Louwagie *et al.*, “Effect of a brief motivational interview and text message intervention targeting tobacco smoking, alcohol use and medication adherence to improve tuberculosis treatment outcomes in adult patients with tuberculosis: A multicentre, randomised controlled trial of the ProLife programme in South Africa,” *BMJ Open*, vol. 12, no. 2, Feb. 2022, doi: 10.1136/bmjopen-2021-056496.

[17] S. Mohammed, R. Glennerster, and A. J. Khan, “Impact of a daily SMS medication reminder system on tuberculosis treatment outcomes: A randomized controlled trial,” *PLoS One*, vol. 11, no. 11, Nov. 2016, doi: 10.1371/journal.pone.0162944.

[18] Nguyen TS, Nguyen NT, Bao B, Byrkit R, and Dao S, “Mobile health and tuberculosis treatment adherence: a way forward?,” in *45th Union World Conference on Lung Health*, Barcelona, Spain: International Union Against Tuberculosis and Lung Disease (The Union), 2014, pp. S214-215.

[19] Owiti P, Szkwarko D, Diero L, Carter EJ, and Gardner A, “Mobile phone text mesaging reminders to aid adherence to tuberculosis care in Eldoret, Kenya,” in *43rd Union World Conference on Lung Health*, Kuala Lumpur, Malaysia: International Union Against Tuberculosis and Lung Disease (The Union), 2012, pp. S200-201.

[20] Peng H, Xu WG, and Lu WEI, “Mobile phone text messaging forpromoting adherence to anti-tuberculosis treatment: a community-randomised trial in Jiangsu, China,” in *45th Union World Conference on Lung Health*, Barcelona, Spain: International Union Against Tuberculosis and Lung Disease (The Union), 2014, pp. S213-214.

[21] P. Bachina *et al.*, “Programmatic Adoption and Implementation of Video-Observed Therapy in Minnesota: Prospective Observational Cohort Study,” *JMIR Form Res*, vol. 6, no. 8, Aug. 2022, doi: 10.2196/38247.

[22] J. Burzynski *et al.*, “In-Person vs Electronic Directly Observed Therapy for Tuberculosis Treatment Adherence: A Randomized Noninferiority Trial,” *JAMA Netw Open*, vol. 5, no. 1, Jan. 2022, doi: 10.1001/jamanetworkopen.2021.44210.

[23] S. H. Chen *et al.*, “Advantage in privacy protection by using synchronous video observed treatment enhances treatment adherence among patients with latent tuberculosis infection,” *J Infect Public Health*, vol. 13, no. 9, pp. 1354–1359, Sep. 2020, doi: 10.1016/j.jiph.2020.03.013.

[24] C. Chuck, E. Robinson, M. Macaraig, M. Alexander, and J. Burzynski, “Enhancing management of tuberculosis treatment with video directly observed therapy in New York City,” *International Journal of Tuberculosis and Lung Disease*, vol. 20, no. 5, pp. 588–593, May 2016, doi: 10.5588/ijtld.15.0738.

[25] S. Doltu *et al.*, “Short and long-term outcomes of video observed treatment in tuberculosis patients, the Republic of Moldova,” *J Infect Dev Ctries*, vol. 15, no. 91, pp. 17S-24S, Sep. 2021, doi: 10.3855/jidc.14601.

[26] R. S. Garfein *et al.*, “Tuberculosis treatment monitoring by video directly observed therapy in 5 health districts, California, USA,” *Emerg Infect Dis*, vol. 24, no. 10, pp. 1806–1815, Oct. 2018, doi: 10.3201/eid2410.180459.

[27] X. Guo *et al.*, “A comprehensive app that improves tuberculosis treatment management through video-observed therapy: Usability study,” *JMIR Mhealth Uhealth*, vol. 8, no. 7, 2020, doi: 10.2196/17658.

[28] P. Guo, W. Qiao, Y. Sun, F. Liu, and C. Wang, "Telemedicine technologies and tuberculosis management: A randomized controlled trial," *Telemedicine and e-Health*, vol. 26, no. 9, pp. 1150–1156, Sep. 2020, doi: 10.1089/tmj.2019.0190.
[29] C. K. Lam, K. M. G. Pilote, A. Haque, J. Burzynski, C. Chuck, and M. Macaraig, "Using video technology to increase treatment completion for patients with latent tuberculosis infection on 3-month isoniazid and rifapentine: An implementation study," *J Med Internet Res*, vol. 20, no. 11, 2018, doi: 10.2196/jmir.9825.
[30] C. K. Lippincott, A. Perry, E. Munk, G. Maltas, and M. Shah, "Tuberculosis treatment adherence in the era of COVID-19," *BMC Infect Dis*, vol. 22, no. 1, Dec. 2022, doi: 10.1186/s12879-022-07787-4.
[31] A. Perry *et al.*, "Real-world implementation of video-observed therapy in an urban TB program in the United States," *International Journal of Tuberculosis and Lung Disease*, vol. 25, no. 8, pp. 655–661, Aug. 2021, doi: 10.5588/ijtld.21.0170.
[32] L. Ravenscroft *et al.*, "Video-observed therapy and medication adherence for tuberculosis patients: Randomised controlled trial in Moldova," *European Respiratory Journal*, vol. 56, no. 2, Aug. 2020, doi: 10.1183/13993003.00493-2020.
[33] J. Salcedo, M. Rosales, J. S. Kim, D. Nuno, S. Suen, and A. H. Chang, "Cost-effectiveness of artificial intelligence monitoring for active tuberculosis treatment: A modeling study," *PLoS One*, vol. 16, no. July, Jul. 2021, doi: 10.1371/journal.pone.0254950.
[34] M. M. Salerno *et al.*, "Adverse events among persons with TB using in-person vs. electronic directly observed therapy," *International Journal of Tuberculosis and Lung Disease*, vol. 27, no. 11, pp. 833–840, Nov. 2023, doi: 10.5588/ijtld.22.0594.
[35] S. Siddiqui *et al.*, "Video Directly Observed Therapy for Tuberculosis Treatment at Harris County Public Health: A Cost Analysis and Adherence Assessment," 2019.
[36] A. Story *et al.*, "Smartphone-enabled video-observed versus directly observed treatment for tuberculosis: a multicentre, analyst-blinded, randomised, controlled superiority trial," *The Lancet*, vol. 393, no. 10177, pp. 1216–1224, Mar. 2019, doi: 10.1016/S0140-6736(18)32993-3.
[37] V. A. Wade, J. Karnon, J. A. Eliott, and J. E. Hiller, "Home Videophones Improve Direct Observation in Tuberculosis Treatment: A Mixed Methods Evaluation," *PLoS One*, vol. 7, no. 11, Nov. 2012, doi: 10.1371/journal.pone.0050155.
[38] Yu MC, Feng WH, Shia TF, Hsu KM, and Lin H C, "Application of mobile videophone in the DOTS-Plus program of northern Taiwan," in *44th Union World Conference on Lung Health*, Paris, France: International Union Against Tuberculosis and Lung Disease (The Union), 2013, pp. S559-560.
[39] J. Acosta, P. Flores, M. Alarcón, M. Grande-Ortiz, L. Moreno-Exebio, and Z. M. Puyen, "A randomised controlled trial to evaluate a medication monitoring system for TB treatment," *International Journal of Tuberculosis and Lung Disease*, vol. 26, no. 1, pp. 44–49, Jan. 2022, doi: 10.5588/ijtld.21.0373.
[40] S. Broomhead and M. Mars, "Retrospective return on investment analysis of an electronic treatment adherence device piloted in the Northern Cape Province," *Telemedicine and e-Health*, vol. 18, no. 1, pp. 24–31, Jan. 2012, doi: 10.1089/tmj.2011.0143.
[41] Charalambous S *et al.*, "TB treatment adherence amongst drug-susceptible TB persons using medication monitor and differentiated care approach versus standard of care in South Africa," in *53rd Union World Conference on Lung Health*, Virtual: International Union Against Tuberculosis and Lung Disease (The Union), 2022, p. S302.

[42] G. Guo *et al.*, "eDOTS: Improving the Treatment of Pulmonary Tuberculosis in Xinjiang, China," *Infect Drug Resist*, vol. 16, pp. 7497–7505, 2023, doi: 10.2147/IDR.S438962.

[43] Kurada J *et al.*, "Treatment adherence by real-time medication event reminder monitor device in Hyderabad, Telangana State: a cohort study," in *50th Union World Conference on Lung Health*, Hyderabad, India: International Union Against Tuberculosis and Lung Disease (The Union), 2019, pp. S234-235.

[44] D. Jerene *et al.*, "Effectiveness of digital adherence technologies in improving treatment outcomes in persons with drug-susceptible tuberculosis: results from pragmatic, cluster randomized trials in four countries." [Online]. Available: https://ssrn.com/abstract=4720744

[45] X. Liu *et al.*, "Digital adherence technologies to improve tuberculosis treatment outcomes in China: a cluster-randomised superiority trial," *Lancet Glob Health*, vol. 11, no. 5, pp. e693–e703, May 2023, doi: 10.1016/S2214-109X(23)00068-2.

[46] T. Manyazewal, Y. Woldeamanuel, D. P. Holland, A. Fekadu, and V. C. Marconi, "Effectiveness of a digital medication event reminder and monitor device for patients with tuberculosis (SELFTB): a multicenter randomized controlled trial," *BMC Med*, vol. 20, no. 1, Dec. 2022, doi: 10.1186/s12916-022-02521-y.

[47] T. Manyazewal *et al.*, "Patient-reported usability and satisfaction with electronic medication event reminder and monitor device for tuberculosis: a multicentre, randomised controlled trial," *EClinicalMedicine*, vol. 56, Feb. 2023, doi: 10.1016/j.eclinm.2022.101820.

[48] T. Manyazewal, Y. Woldeamanuel, A. Fekadu, D. P. Holland, and V. C. Marconi, "Effect of Digital Medication Event Reminder and Monitor-Observed Therapy vs Standard Directly Observed Therapy on Health-Related Quality of Life and Catastrophic Costs in Patients with Tuberculosis: A Secondary Analysis of a Randomized Clinical Trial," *JAMA Netw Open*, vol. 5, no. 9, p. E2230509, Sep. 2022, doi: 10.1001/jamanetworkopen.2022.30509.

[49] T. S. Moulding, M. Caymittes, and T. Moulding, "Managing medication compliance of tuberculosis patients in Haiti with medication monitors Petion-Ville, Haiti," 2002.

[50] A. Musiimenta *et al.*, "The feasibility, acceptability, and preliminary impact of real-time monitors and SMS on tuberculosis medication adherence in southwestern Uganda: Findings from a mixed methods pilot randomized controlled trial," *PLOS Global Public Health*, vol. 3, no. 12, p. e0001813, Dec. 2023, doi: 10.1371/journal.pgph.0001813.

[51] S. Park *et al.*, "Medication event monitoring system for infectious tuberculosis treatment in Morocco: A retrospective cohort study," *Int J Environ Res Public Health*, vol. 16, no. 3, Jan. 2019, doi: 10.3390/ijerph16030412.

[52] Pima FM *et al.*, "Effectiveness of evriMED in ensuring TB treatment adherence among TB patients in the Kilimanjaro Region: a cluster-randomised controlled two-armed trial," in *52nd Union World Conference on Lung Health*, Virtual: International Union Against Tuberculosis and Lung Disease (The Union), 2021, p. S131.

[53] R. Ratchakit-Nedsuwan *et al.*, "Ensuring tuberculosis treatment adherence with a mobile-based CARE-call system in Thailand: a pilot study," *Infect Dis*, vol. 52, no. 2, pp. 121–129, Feb. 2020, doi: 10.1080/23744235.2019.1688862.

[54] S. Saha *et al.*, "Tuberculosis Monitoring Encouragement Adherence Drive (TMEAD): Toward improving the adherence of the patients with drug-sensitive tuberculosis in Nashik, Maharashtra," *Front Public Health*, vol. 10, Dec. 2022, doi: 10.3389/FPUBH.2022.1021427/FULL.

[55] K. Velen *et al.*, "The effect of medication event reminder monitoring on treatment adherence of TB patients," *Int J Tuberc Lung Dis*, vol. 27, no. 4, pp. 322–328, Apr. 2023, doi: 10.5588/ijtld.22.0500.
[56] N. Wang *et al.*, "Do electronic medication monitors improve tuberculosis treatment outcomes? Programmatic experience from China," *PLoS One*, vol. 15, no. 11 November, Nov. 2020, doi: 10.1371/journal.pone.0242112.
[57] X. Wang, Q. Fu, M. Zhou, and Y. Li, "How Integrated Digital Tools Can Improve Tuberculosis Medication Adherence: A Longitudinal Study in China," *Telemedicine and e-Health*, vol. 30, no. 2, pp. 490–498, Feb. 2024, doi: 10.1089/tmj.2023.0084.
[58] X. Wei *et al.*, "Effectiveness of a comprehensive package based on electronic medication monitors at improving treatment outcomes among tuberculosis patients in Tibet: a multicentre randomised controlled trial," *The Lancet*, vol. 403, no. 10430, pp. 913–923, Mar. 2024, doi: 10.1016/S0140-6736(23)02270-5.
[59] Z. Wu *et al.*, "Effect of mobile health reminders on tuberculosis treatment outcomes in Shanghai, China: A prospective cohort study," *Front Public Health*, vol. 11, 2023, doi: 10.3389/fpubh.2023.923319.
[60] I. V. Bassett *et al.*, "Sizanani: A Randomized Trial of Health System Navigators to Improve Linkage to HIV and TB Care in South Africa," *J Acquir Immune Defic Syndr*, vol. 73, no. 2, p. 154, Oct. 2016, doi: 10.1097/QAI.0000000000001025.
[61] D. Das Gupta *et al.*, "Choice-Based Reminder Cues: Findings From an mHealth Study to Improve Tuberculosis (TB) Treatment Adherence Among the Urban Poor in India," *World Med Health Policy*, vol. 12, no. 2, pp. 163–181, Jun. 2020, doi: 10.1002/wmh3.337.
[62] Hope M *et al.*, "Impact of a digital adherence support intervention on TB treatment outcomes in a nomadic population: a case study of five facilities in Karamoja subregion, Uganda," in *53rd Union World Conference on Lung Health*, Virtual: International Union Against Tuberculosis and Lung Disease (The Union), 2022, p. S120.
[63] Hope M *et al.*, "Impact of a mobile phone-based interactive voice response software on tuberculosis treatment outcomes at public facilities in Uganda," in *53rd Union World Conference on Lung Health*, Virtual: International Union Against Tuberculosis and Lung Disease (The Union), 2022, pp. S209-210.
[64] V. Khachadourian *et al.*, "People-centred care versus clinic-based DOT for continuation phase TB treatment in Armenia: A cluster randomized trial," *BMC Pulm Med*, vol. 20, no. 1, Apr. 2020, doi: 10.1186/s12890-020-1141-y.
[65] S. Santra, S. Garg, S. Basu, N. Sharma, M. M. Singh, and A. Khanna, "The effect of a mhealth intervention on anti-tuberculosis medication adherence in Delhi, India: A quasi-experimental study," *Indian J Public Health*, vol. 65, no. 1, pp. 34–38, Jan. 2021, doi: 10.4103/ijph.IJPH_879_20.
[66] R. Sodhi *et al.*, "Impact of a pilot mHealth intervention on treatment outcomes of TB patients seeking care in the private sector using Propensity Scores Matching – Evidence collated from New Delhi, India," *medRxiv*, p. 2023.12.05.23299517, Dec. 2023, doi: 10.1101/2023.12.05.23299517.
[67] E. Yoeli *et al.*, "Digital Health Support in Treatment for Tuberculosis," *New England Journal of Medicine*, vol. 381, no. 10, pp. 986–987, Sep. 2019, doi: 10.1056/nejmc1806550.
[68] A. Cattamanchi *et al.*, "Digital adherence technology for tuberculosis treatment supervision: A stepped-wedge cluster-randomized trial in Uganda," *PLoS Med*, vol. 18, no. 5, May 2021, doi: 10.1371/journal.pmed.1003628.

[69] A. Z. Chen, R. Kumar, R. K. Baria, P. K. Shridhar, R. Subbaraman, and W. Thies, "Impact of the 99DOTS digital adherence technology on tuberculosis treatment outcomes in North India: a pre-post study," *BMC Infect Dis*, vol. 23, no. 1, Dec. 2023, doi: 10.1186/s12879-023-08418-2.
[70] R. Crowder *et al.*, "Implementation of enhanced 99DOTS for TB treatment supervision in Uganda: An interrupted time series analysis," *medRxiv*, p. 2024.01.22.24300949, Jan. 2024, doi: 10.1101/2024.01.22.24300949.
[71] P. Thekkur *et al.*, "Outcomes and implementation challenges of using daily treatment regimens with an innovative adherence support tool among HIV-infected tuberculosis patients in Karnataka, India: a mixed-methods study," *Glob Health Action*, vol. 12, no. 1, Jan. 2019, doi: 10.1080/16549716.2019.1568826.
[72] Wambi P *et al.*, "The role of 99DOTS digital adhenrance technology to support tuberculosis treatment adherance among adolescents," in *53rd Union World Conference on Lung Health*, Virtual: International Union Against Tuberculosis and Lung Disease (The Union), 2022, pp. S305-306.
[73] S. H. Browne *et al.*, "Wirelessly observed therapy compared to directly observed therapy to confirm and support tuberculosis treatment adherence: A randomized controlled trial," *PLoS Med*, vol. 16, no. 10, 2019, doi: 10.1371/journal.pmed.1002891.
[74] J. M. H. Haslinda Noor I., "EFFECTIVENESS OF HEALTH EDUCATION MODULE DELIVERED THROUGH WHATSAPP TO ENHANCE TREATMENT ADHERENCE AND SUCCESSFUL OUTCOME OF TUBERCULOSIS IN SEREMBAN DISTRICT, NEGERI SEMBILAN, MALAYSIA," *International Journal of Public Health and Clinical Sciences*, vol. 6, no. 4, Sep. 2019, doi: 10.32827/ijphcs.6.4.145.
[75] S. J. Iribarren *et al.*, "Patient-centered mobile tuberculosis treatment support tools (TB-TSTs) to improve treatment adherence: A pilot randomized controlled trial exploring feasibility, acceptability and refinement needs," *The Lancet Regional Health - Americas*, vol. 13, p. 100291, 2022, doi: 10.1016/j.
[76] S. N. Zhdanova, O. B. Ogarkov, and S. K. Heysell, "Mobile health intervention for outpatient treatment of tuberculosis and HIV infection," *Acta Biomed Sci*, vol. 5, no. 3, pp. 46–53, 2020, doi: 10.29413/ABS.2020-5.3.7.
[77] M. Zhang *et al.*, "OPEN ACCESS EDITED BY Digitizing tuberculosis treatment monitoring in Wuhan city, China, Impact on medication adherence," 2023.
[78] Zhou X, Zuo T, Zhao X, Cheng T, and Zhao J, "Effect of mobile app-based nursing health education on patients with pulmonary multidrug-resistant tuberculosis," in *49th Union World Conference on Lung Health*, The Hague, The Netherlands: International Union Against Tuberculosis and Lung Disease (The Union), 2018, p. S288.
